# Donor–Acceptor Pentacene Analogues With Near‐Infrared Emission and Tunable Aromaticity

**DOI:** 10.1002/anie.202523436

**Published:** 2026-03-30

**Authors:** Krzysztof Nowak, Olaf Morawski, Muhammad Yasir Mehboob, Maja Morawiak, Krzysztof Noworyta, Cina Foroutan‐Nejad, Marek Grzybowski

**Affiliations:** ^1^ Institute of Organic Chemistry Polish Academy of Sciences Warsaw Poland; ^2^ Institute of Physics Polish Academy of Sciences Warsaw Poland; ^3^ Institute of Physical Chemistry Polish Academy of Sciences Warsaw Poland

**Keywords:** heteroacenes, π‐systems, aromaticity, near‐infrared dyes, π‐conjugated materials

## Abstract

Strategic substitution of the 5 and 12 positions in pentacene with an electron‐donating nitrogen and electron‐accepting phosphine oxide, sulfone and carbonyl groups results in highly polarized *para*‐quinodimethane (pQDM) derivatives with large dipole moments exceeding 10 D. These push‐pull systems feature narrow HOMO‐LUMO gaps (1.0–1.7 eV), reversible redox properties and near‐infrared (NIR) absorption and emission with maxima between 800 and 1100 nm. Remarkably, the polarization induces aromaticity switching: unlike their non‐aromatic double–donor and double–acceptor counterparts, the donor–acceptor analogues develop weak aromatic character in the central quinoidal ring, which is further enhanced in polar solvents, as confirmed by theoretical calculations, NMR spectroscopy and X‐ray crystallography. These findings demonstrate how molecular polarization can modulate aromaticity and electronic structure, providing a design platform for functional quinoidal π‐systems with tunable optical and redox properties.

## Graphical abstract

Air‐ and photostable heterocyclic pentacene analogues with donor and acceptor groups flanking a *para*‐quinodimethane core exhibit large dipole moments, narrow bandgaps, reversible redox behavior, and NIR‐I to NIR‐II absorption and emission with pronounced solvatochromism. Combined theoretical and experimental studies reveal that partial zwitterionic character induces polarity‐dependent aromaticity across the central quinoidal framework.

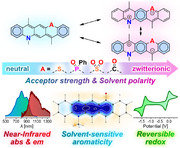

## 1 Introduction

Acenes, linearly fused polycyclic aromatic hydrocarbons (PAHs), are promising small‐molecule semiconducting materials for organic electronics [1, 2]. However, pentacene and higher acenes suffer from extreme air‐ and photosensitivity, making their applications in commercial devices highly challenging [3, 4]. Among many strategies used to stabilize acenes [5], substitution of carbon atoms with heteroatoms (sometimes referred to as “doping”) not only leads to more stable analogues but also unlocks new advantageous properties by tuning their energy levels and photophysics [6, 7, 8]. Nitrogen is by far the most frequently used heteroatom with numerous examples of azaacenes characterized [6, 9], but other elements such as sulfur [10, 11], boron [12, 13, 14], phosphorus [15, 16, 17, 18], as well as boron–nitrogen pairs [19, 20, 21, 22, 23] were also used to that end.

**SCHEME 1:**
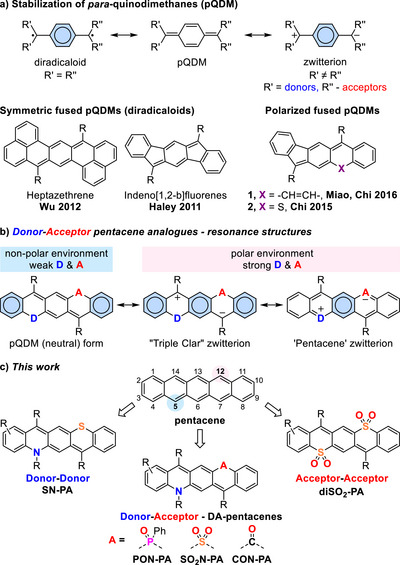
a) Resonance structures of *para*‐quinodimethanes and examples of polycyclic arenes based on the pQDM core, b) polarization‐dependent aromaticity of donor–acceptor pentacene analogues, c) DD, AA, and DA‐pentacenes described herein. R = aryl, ─C≡C─TIPS, alkyl, etc.

Some heteroacenes incorporate *para*‐quinodimethane (pQDM) cores, which become isoelectronic to acenes only upon two‐electron oxidation [9, 10, 11, 24], or reduction [12, 25]. While most studies have focused on symmetric pQDM‐based polycyclic arenes with diradical character [5, 26, 27, 28], such as zethrenes [29] and indenofluorenes [30, 31, 32, 33], for example heptazethrene and indeno[1,2‐*b*]fluorene (Scheme 1a), unsymmetrical derivatives remain largely unexplored.

In non‐fused pQDM derivatives, strong polarization induced by electron‐donating and ‐accepting groups leads to a fully zwitterionic structure containing a Clar sextet in the central ring, rendering it Hückel aromatic (Scheme 1a), as demonstrated for diaminodicyanoquinones and related molecules [34, 35, 36].

In contrast, only weakly polarized analogues have been described for acene‐like PAHs containing a fused pQDM core. In 2016 Miao, Chi and co‐workers reported pentacene isomers incorporating a motif of consecutively fused five‐, six‐, and seven‐membered rings (**1**, Scheme 1a) [37]. As in azulene, the five‐ and seven‐membered rings act as electron acceptor and donor, respectively, leading to a dipole moment of 2.6 D. A related system **2**, bearing a sulfur‐containing six‐membered ring as the donor, shows a slightly higher dipole moment of 3.1 D [38, 39]. In both cases, polarization enhanced stability, narrowed the HOMO‐LUMO gap, and shifted absorption to the far‐red region. More recently, Haley and co‐workers reported pronounced charge‐transfer effects in extended push‐pull derivatives of antiaromatic *s*‐indacene, which also feature embedded pQDM motifs [40].

Inspired by these seminal works, we wondered whether it might be possible to bridge the two intriguing realms of π‐conjugated systems—acenes and fused *para*‐quinodimethanes—by strategically replacing the carbon atoms at positions 5 and 12 of pentacene with electron‐donating and electron‐accepting groups, respectively (Scheme 1b).

In the ideal case where the donor and acceptor form a Lewis pair (e.g., a nitrogen‐boron or nitrogen‐carbonyl group), the resulting π‐system with 22 π‐electrons would be isoelectronic with the parent pentacene and could retain some of its optoelectronic properties. Simultaneously, the molecule would become strongly polarized due to the presence of the push–pull system with a pQDM core. To explore this concept, we considered the Lewis resonance structures of such donor–acceptor‐substituted pentacenes (DA‐pentacenes, Scheme 1b). A neutral pQDM mesomer would dominate when weak donor and acceptor groups are present or in non‐polar media. Conversely, stronger donor–acceptor pairs and a polar environment would increase the contribution of zwitterionic resonance forms (referred to as the “Triple Clar” and “Pentacene” forms in Scheme 1b). In these zwitterions, positive and negative charges are stabilized within the donor‐ and acceptor‐fused rings, respectively, and the pQDM ring gains aromatic character.

Hence, we hypothesized that the electronic structure—and particularly the aromaticity—of these molecules would be highly sensitive to the environment, shifting from locally aromatic in non‐polar solvents to globally aromatic in polar ones, which should manifest as strong solvatochromism. The large dipole moment and partial zwitterionic structure were also expected to enhance stability by suppressing diradical and diene character, while the push–pull nature should narrow the HOMO‐LUMO gap, leading to near‐infrared absorption and emission.

To test this concept, we designed a series of donor–acceptor systems with nitrogen as the donor and functional groups of increasing electron‐withdrawing strength—phosphine oxide, sulfone, and carbonyl groups—as acceptors. All analogues were successfully synthesized, together with donor–donor and acceptor–acceptor references for comparison (Scheme 1c). Herein, we report air‐ and photostable donor–acceptor pentacene analogues with a polarized *para*‐quinodimethane core, exhibiting near‐infrared absorption and fluorescence and polarity‐dependent aromaticity.

## 2 Results and Discussion

### 2.1 Synthesis

**SCHEME 2:**
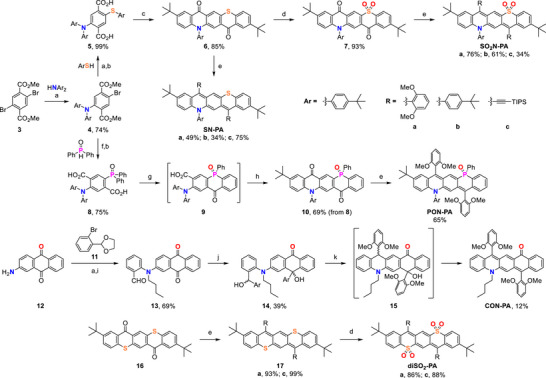
Synthesis of donor–acceptor, donor–donor, and acceptor–acceptor pentacene analogues. Reaction conditions: a) Pd(OAc)_2_, XantPhos, K_3_PO_4_ or Cs_2_CO_3_, toluene, 120°C; b) NaOH, THF, H_2_O, EtOH, reflux (**5**: quant., **8**: 82%); c) i. (COCl)_2_, DCM, 0°C to rt, ii. TiCl_4_, DCM, rt; d) mCPBA, DCM, rt; e) i. RLi, THF, 0°C to rt, ii. SnCl_2_; f) Pd(OAc)_2_, dppp, DIPEA, toluene, 120°C; g) LDA, THF, 0°C to rt; h) Eaton's reagent (10% P_4_O_10_ in MsOH), 60°C; i) i. *n*‐BuBr, KOH, DMF, 50°C, ii. *p*TsOH (20 mol%), MeCN, H_2_O, 65°C; j) 2,6‐dimethoxyphenyllithium, THF, 0°C to rt; k) Sc(OTf)_3_ (30 mol%), DCE, 90°C.

Our approach to synthesizing donor–acceptor pentacene analogues consisted of two general stages. The initial step involved the preparation of unsymmetric quinacridone analogues—diketones **7** and **10**—in which one of the nitrogen atoms is substituted with an electron‐withdrawing sulfone or phosphine oxide group. In the subsequent step, these diketones were reacted with organolithium reagents followed by reduction to yield the final products (Scheme 2).

Both diketones were synthesized starting from dimethyl 2,5‐dibromoterephthalate (**3**) through different palladium‐catalyzed coupling reactions. First, the Buchwald–Hartwig reaction of bis(4‐*tert*‐butylphenyl)amine with a slight excess of diester **3** furnished the monoaminated product **4** in 74% yield. To obtain the sulfur/nitrogen unsymmetric diketones **6** and **7**, compound **4** was subjected to a carbon–sulfur coupling reaction [41] with 4‐*tert*‐butylthiophenol in the presence of palladium(II) acetate / XantPhos as catalyst. Subsequent hydrolysis of the resulting diester afforded the unsymmetric terephthalic acid **5**, bearing amino and thioether groups, in quantitative yield (Scheme 2). The carboxylic groups in **5** were converted to acid chlorides using oxalyl chloride, and the resulting intermediate was treated with titanium(IV) chloride to promote an intramolecular Friedel–Crafts reaction, affording S,N quinacridone analogue **6** in 85% yield. Finally, diketone **6** was oxidized with *m*CPBA to the corresponding sulfone **7** in 93% yield.

For the synthesis of the phosphorus/nitrogen diketone **10**, a carbon–phosphorus coupling reaction [42] was performed on intermediate **4** with diphenylphosphine oxide in the presence of palladium(II) acetate and dppp as ligand. Subsequent hydrolysis afforded the phosphine oxide‐substituted terephthalic acid **8** in 75% yield. Attempts to cyclize diacid **8** to diketone **10** under conventional Friedel–Crafts conditions were unsuccessful, likely due to the electron‐withdrawing nature of the phosphine oxide group. Instead, the phosphinine ring was constructed using an *ortho*‐lithiation protocol developed by Snieckus and co‐workers, employing the phosphine oxide as a directing group and LDA as the base [43]. The resulting phospha–xanthone **9** was then subjected to an intramolecular Friedel–Crafts reaction with Eaton's reagent, affording diketone **10** in 69% yield (Scheme 2).

The unsymmetrical diketones **6**, **7,** and **10** were transformed into fully conjugated pentacene analogues *via* the addition of organolithium reagents, followed by reduction of the resulting diols with tin(II) chloride (Scheme 2). Aryl‐ or (triisopropylsilyl)ethynyl‐substituted donor–acceptor analogues **SO_2_N‐PA** and **PON‐PA**, as well as donor–donor derivatives **SN‐PA** (for reference), were obtained in generally good yields. The aryl‐substituted derivatives required harsher reduction conditions, involving overnight stirring with SnCl_2_ at 80°C in toluene with trifluoroacetic acid [32].

For the nitrogen‐carbonyl analogue **CON‐PA**, a route analogous to those used for **PON‐PA** and **SO_2_N‐PA** was initially explored but proved highly inefficient due to difficulties with the late‐stage oxidation of the methylene group to a carbonyl (see Scheme S1). Consequently, we designed an alternative synthetic pathway, starting from carbonyl‐preinstalled 2‐aminoanthraquinone (**12**) (Scheme 2). Buchwald‐Hartwig amination and *N*‐alkylation, followed by acetal deprotection, afforded aldehyde **13**. Since one of the carbonyl groups in the anthraquinone moiety is deactivated by the electron‐donating amine in the *para* position, we anticipated that the addition of aryllithium would occur predominantly at the remaining two carbonyl groups. Indeed, the desired adduct **14** was obtained in 39% yield, although other regioisomers were also present in the reaction mixture. We hypothesized that under acidic conditions, diol **14** could undergo Friedel‐Crafts cyclization followed by dehydration of the intermediate **15** to yield **CON‐PA**. While boron trifluoride as the Lewis acid promoted only the initial cyclization, prolonged heating of diol **14** in the presence of scandium triflate afforded the desired **CON‐PA** in 12% yield.

In addition to the unsymmetrical analogues, we also synthesized the symmetric bis‐sulfone derivatives to serve as acceptor‐acceptor reference compounds. The corresponding dithia precursors **17** were prepared according to the procedures reported by Chi and co‐workers, [24] and oxidized with *m*CPBA to the desired disulfones **diSO_2_‐PA**‐**a** and ‐**c** (Scheme 2).

All the obtained acene analogues are strongly colored in solid and in solution. **SN‐PA** derivatives are violet or deep‐blue, while, surprisingly, the disulfones form bright fluorescent orange (**diSO_2_‐PA‐a**) or red (**diSO_2_‐PA‐c**) solutions. On the other hand, all DA‐pentacenes are green both in solid and in solution, suggesting absorption in far‐red or NIR regions. Due to aggregation tendencies, in some cases, recording NMR spectra required elevated temperatures or protonation with TFA.

### 2.2 Crystal Structures

**FIGURE 1:**
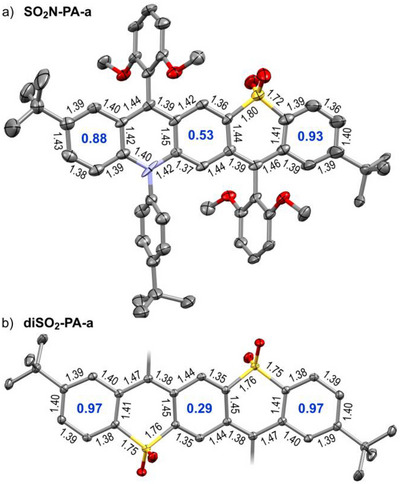
Crystal structures of a) **SO_2_N‐PA‐a** and b) **diSO_2_‐PA‐a**. Bond lengths (black, in Å) and HOMA values (blue) of carbocyclic rings are given. Ellipsoids are drawn at the 50% probability level; hydrogen atoms were omitted for clarity. In b) aryl groups are not shown. CCDC 2492475 and 2451462 [44].

Due to their unsymmetrical and dipolar structures, DA‐pentacenes exhibit good solubility in most organic solvents, which makes the growth of suitable single crystals for X‐ray analysis particularly challenging. Nevertheless, we successfully obtained crystal structures of **SO_2_N‐PA‐a** and **CON‐PA**, as well as the symmetric disulfones **diSO_2_‐PA**‐**a** and ‐**c** (Figures S1–S6), [44] whereas **PON‐PA** yielded crystals of insufficient quality. Figure 1 presents a structural comparison of **SO_2_N‐PA‐a** and **diSO_2_‐PA**‐**a**, both bearing 2,6‐dimethoxyphenyl substituents.

**SO_2_N‐PA‐a** crystallizes as a toluene solvate in the *P*2_1_ space group, rendering the crystal non‐centrosymmetric and thus chiral, despite its achiral components. The π‐system is nearly planar, with the largest deviations (0.24 Å) at the peripheral rings (Figures 1 and S1). The aryl groups adopt similar rotational orientation, tilted by 70°–80° relative to the π‐system plane. Interatomic distances reveal a quinoidal pattern in the pQDM core with lower bond length alternation (BLA) in the peripheral benzene rings.

Comparison with the symmetric acceptor–acceptor reference compound **diSO_2_‐PA**‐**a** (space group: *P*2_1_/*c*, Figures 1b and S3) clearly shows that in the donor–acceptor analogue, BLA decreases in the central ring and increases in the peripheral rings, suggesting enhanced aromatic character in the *p*‐quinodimethane unit (see also Figure S6). The harmonic oscillator model of aromaticity (HOMA), [45, 46] a well‐established and reliable empirical method for quantifying aromaticity based purely on molecular geometry, supports this observation. The HOMA value of the central benzene ring increases from 0.29 (disulfone) to 0.53 (azasulfone), while it slightly decreases in the peripheral rings. As anticipated, the pQDM core gains some aromatic character due to polarization.

### 2.3 Photophysical Properties

**FIGURE 2:**
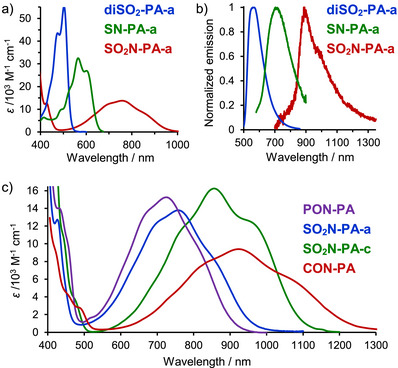
Comparison of UV–vis‐NIR absorption a) and normalized emission b) spectra of acceptor–acceptor (**diSO_2_‐PA‐a**), donor–donor (**SN‐PA‐a**) and donor–acceptor (**SO_2_N‐PA‐a**) pentacene analogues in dichloromethane. c) Comparison of absorption spectra of DA‐PA derivatives with different acceptors or pendant groups in dichloromethane.

UV–vis absorption and fluorescence measurement results are summarized in Table 1 and Figure 2 (see ESI for more details). The most illustrative comparison involves azasulfones (**SO_2_N‐PA**) alongside their double‐acceptor (**diSO_2_‐PA**) and double–donor (**SN‐PA**) counterparts, where the strong polarization effects on photophysical properties become evident (Figures 2a and S8–S15).

**TABLE 1:** Photophysical properties of the obtained compounds.

Compound	Solvent	*λ*_abs_ ^a^ [nm]	*ε*_max_ [M^−1^ cm^−1^]	*λ*_em_ [nm]	*Φ*_fl_ ^b^ [%]
**diSO_2_‐PA‐a**	*n*‐hexane CH_2_Cl_2_ MeOH	496 503 498	59 600 55 300 60 100	528 560 568	92 93 1.9
**SN‐PA‐a**	*n*‐hexane CH_2_Cl_2_ MeOH	557, 596 565, 602 560, 594	32 900 32 400 30 700	693 708 nd	3.6 2.1 Nd
**PON‐PA**	*n*‐hexane CH_2_Cl_2_ MeOH	700 (782) 725 (823) 740 (841)	15 100 15 300 13 700	864 909 947	0.075 0.19 0.014
**SO_2_N‐PA‐a**	*n*‐hexane CH_2_Cl_2_ MeOH	718 (808) 757 (862) 758 (869)	16 300 13 800 15 300	899 896 966	0.023 0.028 0.006
**CON‐PA**	*n*‐hexane CH_2_Cl_2_ MeOH	852 (1014) 924 (1082) 978 (1159)	9 900 9 400 8 100	nd	Nd
**diSO_2_‐PA‐c**	*n*‐hexane CH_2_Cl_2_ MeOH	550 559 551	104 900 92 800 85 700	567 622 648	81 71 25
**SN‐PA‐c**	CH_2_Cl_2_	669	40 100	nd	Nd
**SO_2_N‐PA‐c**	*n*‐hexane CH_2_Cl_2_ MeOH	805 (911) 855 (975) 856 (978)	15 300 16 200 16 100	1002 1066 1068	0.013 0.005 0.003

^a^ Values in round brackets correspond to the wavelengths of the 0─0 vibronic transitions obtained by non‐linear curve fitting, see Table S2 for details.

^b^ Fluorescence quantum yields measured using IR140 in DMSO (*Φ*_fl_  =  15%), fluorescein in 0.1 M NaOH_aq_ (*Φ*_fl_  =  91%) and sulforhodamine 101 in ethanol (*Φ*_fl_  =  90%) as references.

In dichloromethane (DCM), the 2,6‐dimethoxyphenyl‐substituted disulfone **diSO_2_‐PA‐a** exhibits sharp, intense absorption and fluorescence bands at relatively short wavelengths (*λ*_abs_  =  503 nm, *λ*_em_  =  560 nm) with a remarkably high fluorescence quantum yield (*Φ*_fl_  =  93%). Changing the system to donor–donor in **SN‐PA‐a** results in a pronounced bathochromic shift of both absorption (∼100 nm) and emission (∼145 nm), accompanied by a drastic drop in fluorescence quantum yield to 2%. **SN‐PA‐a** absorbs at wavelengths comparable to those of its symmetric S,S (*λ*_abs_  =  558 nm) [10, 24] and N,N (*λ*_abs_  =  574 nm) [9] analogues.

However, when both donor and acceptor flank the pQDM core, much larger bathochromic shifts occur. Unlike the DD and AA analogues, the absorption band of **SO_2_N‐PA‐a** is broad and weakly structured, with a global maximum at 757 nm (0‐1 vibronic band). The lowest energy 0─0 band is obscured but can be estimated at 862 nm (11,600 cm^−1^) *via* nonlinear Gaussian fitting (Table 1 and S2). This represents a dramatic redshift of 260–360 nm (5000–8300 cm^−1^) compared to the DD and AA counterparts, highlighting significant bandgap narrowing in this polarized molecule.

**SO_2_N‐PA‐a** also exhibits weak but detectable NIR fluorescence (*λ*_em_  =  897 nm, *Φ*_fl_  =  0.03%), aligning with the NIR‐I biological window. Substituting the 2,6‐dimethoxyphenyl groups with TIPS‐ethynyl in **diSO_2_‐PA‐c, SN‐PA‐c, and SO_2_N‐PA‐c** induces additional bathochromic shifts (60–100 nm) due to π‐system expansion. However, the general trends remain consistent, with the azasulfone derivative exhibiting the longest‐wavelength absorption and emission maxima (Figure S11).

The remaining donor–acceptor pentacene analogues also exhibit absorption (Figure 2b) and emission (Figure S9, except of **CON‐PA** for which emission could not be detected) in the NIR region. The absorption and emission maxima strongly depend on the acceptor strength: the stronger the acceptor, the longer the wavelengths. Among DA‐pentacenes with identical 2,6‐dimethoxyphenyl substituents, azaphosphone **PON‐PA**, featuring a phosphine oxide acceptor, has the shortest‐wavelength absorption maximum (*λ*_abs_  =  725 nm in DCM), followed by **SO_2_N‐PA‐a** (*λ*_abs_  =  757 nm), while **CON‐PA** exhibits the longest‐wavelength absorption (*λ*_abs_  =  924 nm). The corresponding 0‐0 absorption bands range from 823 nm (**PON‐PA**) to 1082 nm (**CON‐PA**) (12,150 cm^−1^ to 9,240 cm^−1^), highlighting the profound effect of increasing polarization on the photophysical properties of these push‐pull systems.

The dipolar nature of DA‐pentacenes is also reflected in significant solvatochromism, confirmed by measurements in solvents of varying polarity—from non‐polar *n*‐hexane to moderately polar dichloromethane and highly polar acetonitrile and methanol (Figure S8). In all cases, increasing solvent polarity induces noticeable redshifts in both absorption and emission spectra, with solvatochromic shifts of 750–1300 cm^−1^ from *n*‐hexane to methanol (Table 1), showing positive solvatochromism and a clear dependence on the *E*_T_(30) polarity scale (Figure S16) [47]. This contrasts with the acceptor–acceptor **diSO_2_‐PA**, which shows typical positive solvatochromism with larger emission than absorption shifts (redshifts of 30–80 cm^−1^ and 1600–2500 cm^−1^ for absorption and emission maxima, respectively, Figures S12 and S13). Comparable solvatochromism in the absorption and fluorescence spectra of DA‐pentacenes is reminiscent of that reported for rhodol analogues based on phospha‐xanthene [48] and fluorene, [49] which likewise contain quinoidal fragments in push–pull π‐systems, and suggests unusual solvent‐dipole interactions in both the ground and excited states.

Crucially, except for **CON‐PA**, DA‐pentacenes exhibit detectable fluorescence in the NIR region. Weakly polarized **PON‐PA** and **SO_2_N‐PA‐a** emit near 900 nm, while π‐expanded **SO_2_N‐PA‐c** fluoresces between 1000–1070 nm, corresponding to biological NIR‐I and NIR‐II windows. However, fluorescence quantum yields remain very low (*Φ*_fl_ < 0.1%), likely due to the energy gap law [50, 51]. Although modest, these efficiencies are comparable to those reported for several state‐of‐the‐art NIR‐II dyes in solution prior to interaction with biological targets (Table S4), [52, 53] even though the DA‐pentacenes were not optimized for emissive applications. Notably, azaphosphone **PON‐PA** exhibits the highest emission efficiency (*Φ*_fl_  =  0.2% in DCM), suggesting that targeted structural modifications could further enhance its quantum yield toward application‐relevant levels.

The DA‐pentacenes exhibit exceptional photostability: even prolonged irradiation with a 777 nm laser (1.5 W, 60 min) caused no detectable loss of absorption, confirming their high stability under intense excitation (Figure S17).

DA‐pentacenes display high stability both in the solid state and in solution. No decomposition was observed upon prolonged storage of the solid samples under ambient conditions, including exposure to air and moisture (see Supporting Information for details). Their chemical stability was further evaluated in solution: the compounds are stable in neutral methanol under air for at least several days, showing no decrease in absorption intensity (Figures S18 and S19), while protonation under acidic conditions—leading to the formation of acridinium‐type species (Scheme S2)—is reversible for derivatives bearing bulky 2,6‐dimethoxyphenyl substituents (Figures S20–S22).

### 2.4 Computational Studies

To gain a better understanding of the electronic structures and photophysics of the DA‐pentacenes, density‐functional theory (DFT) calculations were performed first on the simplified molecules (R  =  H or TMS‐ethynyl, Scheme S3) to identify the appropriate DFT method. Among several tested DFT functionals, the range‐separated functional LC‐ωHPBE with range separation parameter optimized to *ω*  =  0.2 performed the best, that is, well correlated with experimental excitation energies and solvatochromic shifts (see ESI for details). Consequently, all the following results were obtained at the LC‐ωHPBE(*ω*  =  0.2) [54, 55] level of theory with the triple‐zeta def2‐TZVPP [56] basis set using SMD [57] solvation model. Simplified structures were used for calculating both ground and excited state geometries and their properties. To evaluate the effects of substituents, we also calculated the complete structures with all the substituents present in the physically synthesized samples.

**FIGURE 3:**
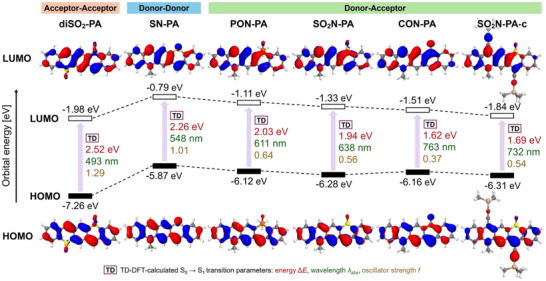
Summary of DFT and TD‐DFT results obtained at the LC‐ ωHPBE (*ω* = 0.2)/def2‐TZVPP level of theory with the SMD solvation model (dichloromethane) for simplified DA‐pentacenes and their double‐donor and double‐acceptor analogues. Canonical HOMO and LUMO orbitals are shown at the bottom and top of the figure, respectively. Orbital energies are displayed in the central energy diagram. TD‐DFT‐predicted parameters of the lowest vertical singlet excitation (S_0_ → S_1_) are indicated: energy gap (Δ*E*, red), absorption wavelength (*λ*_abs_, green), and oscillator strength (*f*, gold).

The results of the computational investigation in dichloromethane are summarized in Figure 3 (simplified structures) and Figure S23 (complete structures). In the case of donor–donor and acceptor‐acceptor references, the orbital energies are expectedly highest, and lowest, respectively. The introduction of the donor–acceptor motif leads to strong narrowing of the HOMO‐LUMO gaps in the order from **PON‐PA** to **CON‐PA**. At the same time, strong ground‐state dipole moments are induced by DA‐polarization, µ_gs_ = 9.3–11.4 D in DCM (Table S7, Figures S24–S29), much larger than in the DD analogue **SN‐PA** (3.9 D) or previously described fused pQDMs [37, 38, 39]. The excitation energies computed using the TD‐DFT approach, like in the experimental data, show substantial redshifts and reduced oscillator strengths when going from the DD/AA to DA derivatives. In all cases S_0_‐S_1_ transitions correspond to HOMO‐LUMO (π‐π*) transitions (Table S7).

While the DD and AA compounds have symmetric or nearly symmetric (**SN‐PA**) distribution of the frontier orbitals, in the DA analogues the distribution is much less uniform (Figure 3). The HOMO is primarily localized along the molecular edge containing the nitrogen donor, whereas the LUMO resides on the opposite edge bearing the acceptor groups (Figure 3, S23). This transverse spatial separation of frontier orbitals reduces their overlap, leading to lower oscillator strengths in the DA systems compared to the DD and AA analogues.

The calculations confirm pronounced bathochromic shifts in absorption and emission of DA‐pentacenes with increasing solvent polarity, narrowing the optical bandgap by ∼0.1–0.2 eV from *n*‐hexane to methanol (Tables S7 and S8). Since solvatochromism usually originates from differences in dipole stabilization between ground and excited states, we examined the dipole moments of the DA‐pentacenes.

Increasing solvent polarity markedly enhances the ground‐state dipole moments, for example, from 7.4 to 10.6 D for **PON‐PA** and from 8.0 to 13.9 D for **CON‐PA**, reflecting the stabilization of zwitterionic resonance forms in polar media (Table S7). This trend is consistent with the bond‐length alternations observed in the optimized geometries: increasing both solvent polarity and acceptor strength leads to elongation of double bonds and shortening of single bonds within the pQDM fragment. The ground‐state dipoles are oriented approximately along the donor–acceptor axis (see Figs. S24–S29 and S35–S38 for bond lengths and dipole orientations). Upon excitation, dipole magnitudes change only slightly and tilt by 20–30° across the acene axis, consistent with the transverse distribution of the orbitals involved in the transition. Excited‐state relaxation has only minor effects on dipole magnitude and orientation. Overall, DA‐pentacenes possess large (∼9–13 D) and comparable dipole moments in both ground and excited states (µ_gs_ ≈ *µ*_es_). This, combined with their rotation, accounts for the similar solvatochromic shifts observed in absorption and emission. In contrast, typical positively solvatochromic dyes show much larger excited‐state dipoles (µ_gs_ < *µ*_es_) leading to stronger ES dipole stabilization and generally larger emission shifts [58].

**FIGURE 4:**
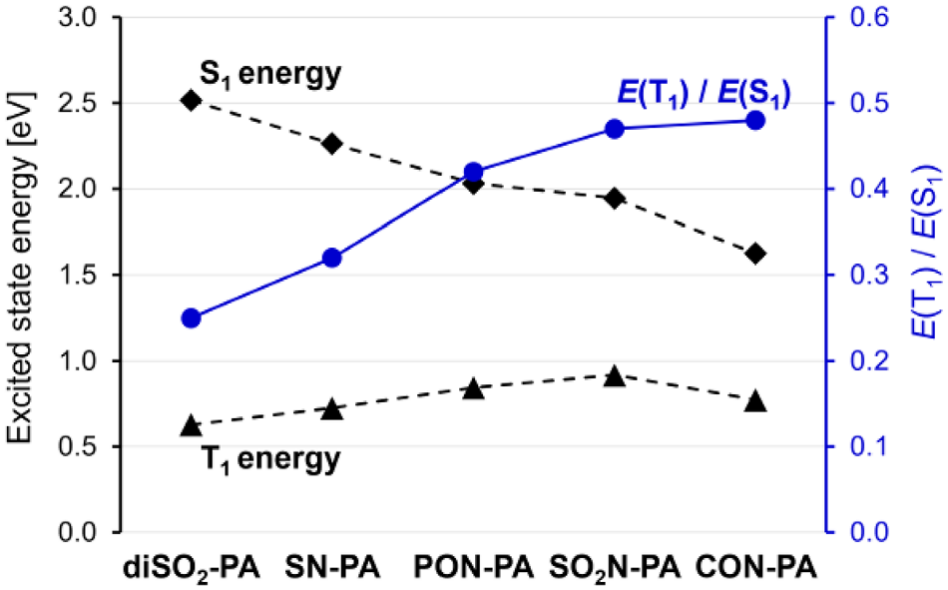
Comparison of the singlet (S_1_, diamonds) and triplet (T_1_, triangles) vertical excited‐state energies, along with their energy ratio *E*(T_1_)/*E*(S_1_) (blue circles), calculated by TD‐DFT at the LC‐ ωHPBE (*ω* = 0.2)/def2‐TZVPP level with the SMD solvation model (dichloromethane).

A comparison of the vertical triplet and singlet excited‐state energies reveals a clear trend: the T_1_ triplet state becomes increasingly destabilized with growing molecular polarization and decreasing S_1_ energy. In DCM, the *E*(T_1_)/*E*(S_1_) ratio increases from 0.25 for the symmetric double‐acceptor derivative **diSO_2_‐PA** to 0.48 for the donor–acceptor analogue **CON‐PA** (Figure 4). Notably, solvent polarity enhances this effect in DA‐pentacenes, for example, in **CON‐PA** the ratio rises from 0.39 in *n*‐hexane to 0.53 in methanol—while differences remain negligible for the AA and DD counterparts (**diSO_2_‐PA**: 0.24–0.25) (Table S9). These results support our reasoning that increasing zwitterionic character of the quinodimethane core leads to destabilization of the corresponding diradical states.

### 2.5 Aromaticity

**FIGURE 5:**
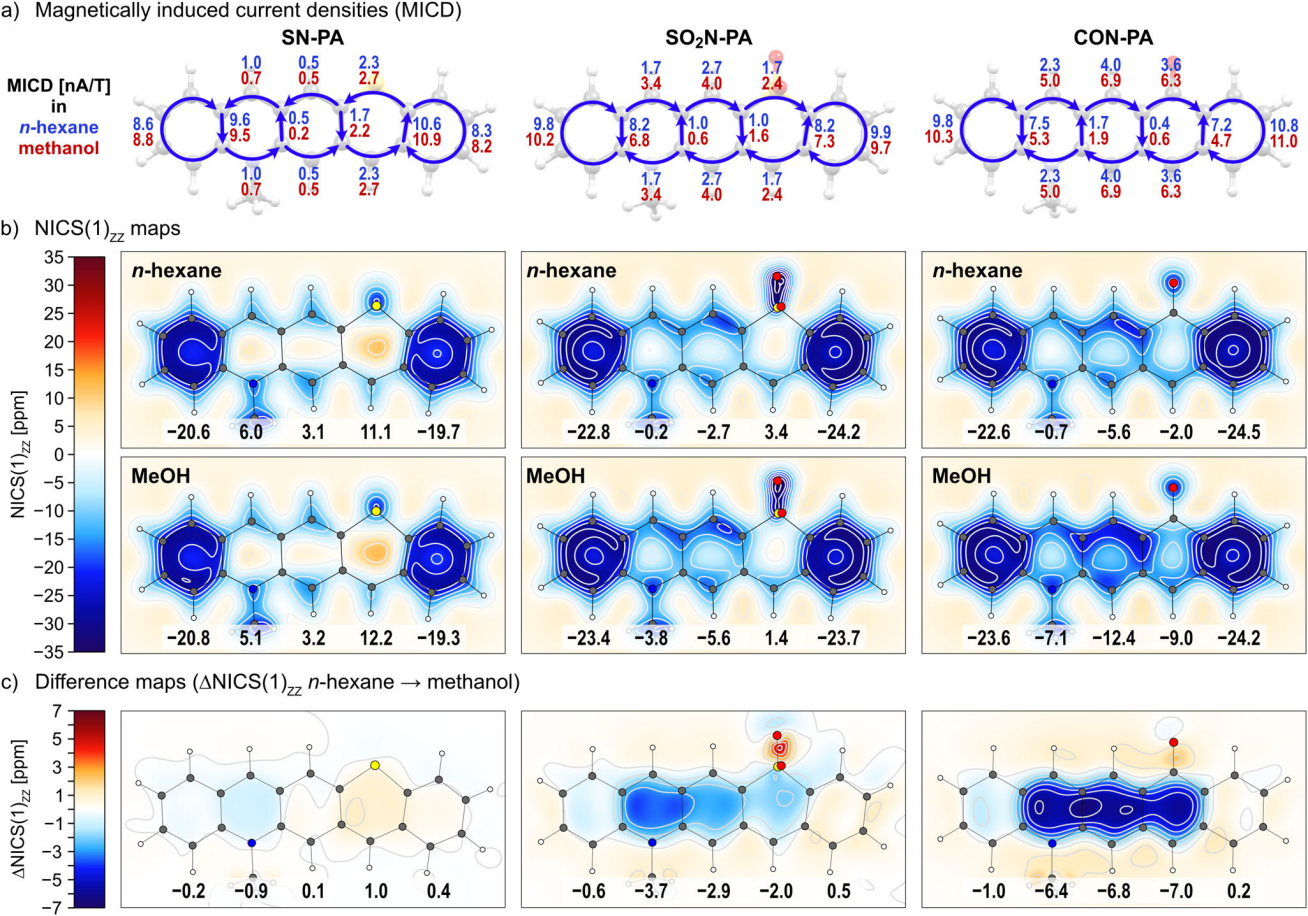
a) Magnetically induced current densities (MICD) calculated in *n*‐hexane and methanol for **SN‐PA**, **SO_2_N‐PA** and **CON‐PA**; b) their 2D NICS(1)_ZZ_ maps; c) differential maps showing changes in NICS(1)_ZZ_ parameter between *n*‐hexane and methanol. In b) and c), NICS(1)_ZZ_ and ΔNICS(1)_ZZ_ values at the ring centers are shown at the bottom of each plot. Results for the remaining analogues, as well as for the NICS(1.7)_ZZ_ parameter, are provided in the Supporting Information.

The resonance structures presented in Scheme 1 suggest a strong relationship between the polarization of the pQDM moiety and the local/global aromatic character of the DA‐pentacenes. To explore this aspect, we examined the ring current strength of the molecules both indirectly via the nucleus independent chemical shifts (NICS(1)_ZZ_ and NICS(1.7)_ZZ_) [59] and directly via the magnetically induced current density (MICD) of these molecules [60]. The NICS values were calculated at rings centres (Table 2 and S10 and S11) and visualized as 2D maps (Figures 5b,c and S44–S51). Since NICS(1)_ZZ_ and NICS(1.7)_ZZ_ results exhibit the same trends, the discussion focuses on the former.

**TABLE 2:** Comparison of NICS(1)_ZZ_ values calculated at ring centers for various heteroatom‐substituted pentacene analogues. The data are color‐coded to emphasize trends in aromaticity and changes in dipole moment.

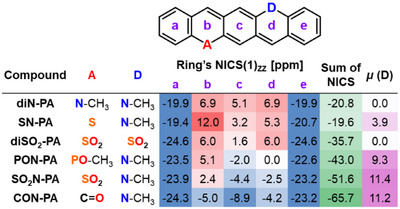

**FIGURE 6:**
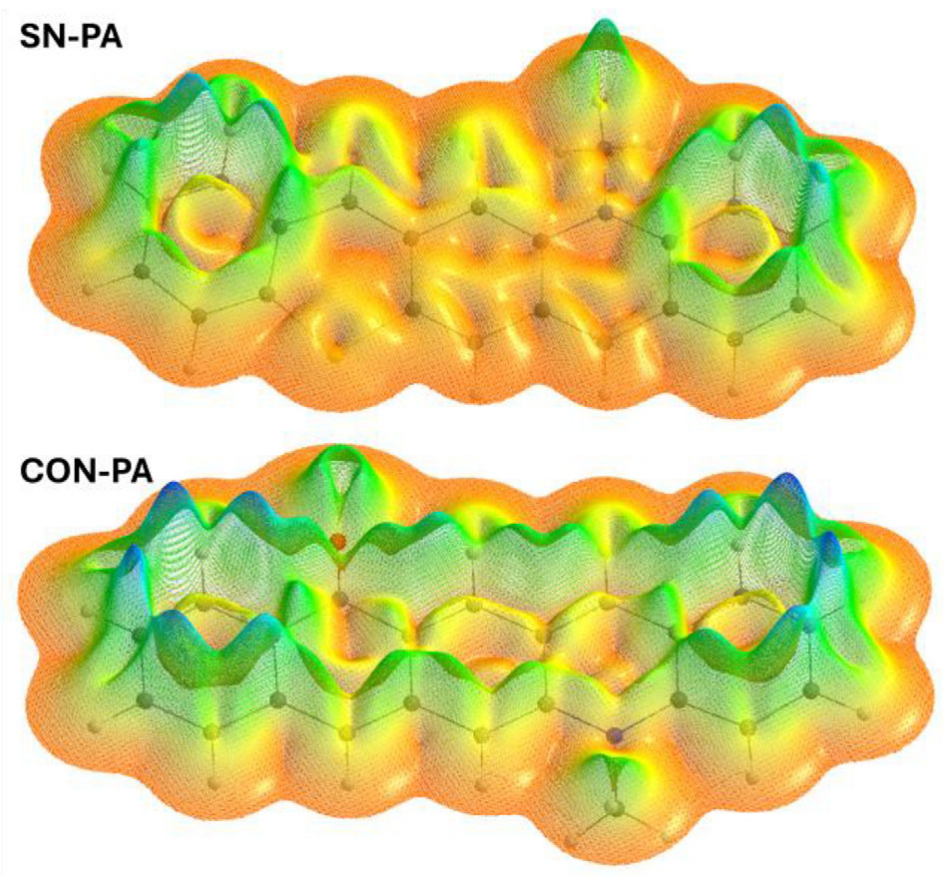
Magnetically induced current densities (MICD) visualized for **SN‐PA** and **CON‐PA** optimized in methanol, showing enhanced global diatropic current of the latter.

The MICD and NICS parameters indicate increasing diatropicity in the central pQDM units of pentacene analogues as the polarization increases (Figures 5 and 6, Table 2). The DD and AA reference compounds exhibit a predominantly non‐aromatic character, with weak induced ring current and NICS values close to 0 ppm (NICS(1)_ZZ_  =  +3.2 ppm and +1.6 ppm at ring centers for **SN‐PA** and **diSO_2_‐PA** in DCM, respectively). However, the introduction of a donor–acceptor motif leads to increasingly negative NICS parameters, with diatropic ring currents strengthening as the electron‐accepting group becomes more potent. This trend is reflected in NICS(1)_ZZ_ values: −2.0 ppm for **PON‐PA**, −4.4 ppm for **SO_2_N‐PA**, and −8.9 ppm for **CON‐PA** in dichloromethane (Table 2). The ring current magnitudes also increase correspondingly (Figures 5a and S42). Interestingly, for **CON‐PA**, diatropic ring currents are observed across all rings, indicating the emergence of global aromaticity.

Even more intriguingly, polarity exerts a strong influence on magnetically induced currents. This effect becomes evident when comparing MICD and NICS values for molecules in non‐polar *n*‐hexane and polar methanol (Figures 5 and 6, S42–S50). While polarity has a negligible impact on ring currents in the bis‐acceptor (**diSO2‐PA**) and bis‐donor (**SN‐PA**) analogues (ΔNICS(1)_ZZ_  =  +0.1–0.3 ppm in the central ring), a significant enhancement of diatropic ring currents is observed in all DA‐pentacenes. This effect is moderate for **PON‐PA** and **SO_2_N‐PA** (ΔNICS(1)_ZZ_  =  −0.9 ppm and −2.9 ppm, respectively) but pronounced for **CON‐PA**, where the NICS(1)_ZZ_ value at the central ring decreases from −5.6 ppm in *n*‐hexane to −12.4 ppm in methanol (ΔNICS(1)_ZZ_  =  −6.8 ppm, Table S10, Figure 5b). This corresponds to an increase in the aromatic ring current from 4.0 to 6.9 nA/T (integrated at the zigzag edges of the pQDM core, Figure 5a). This effect is especially evident in the differential 2D NICS maps (Figure 5c, S42–S50), which clearly illustrate the enhanced aromatic character of DA‐pentacenes in response to higher polarity – showing negligible change for **SN‐PA** or **diSO_2_‐PA** but a strong enhancement of global aromaticity for **CON‐PA**. In line with that, HOMA values also increase significantly with both increasing polarization (from **PON‐PA** to **CON‐PA**) and solvent polarity (from *n*‐hexane to methanol) for all DA‐pentacenes, based on their DFT‐optimized geometries (Table S12). It is worth noting that the increase in the magnetic aromaticity measures correlates with HOMO‐LUMO gap narrowing, reflecting a more efficient virtual transition leading to a stronger magnetic response, as discussed elsewhere [61, 62].

**FIGURE 7:**
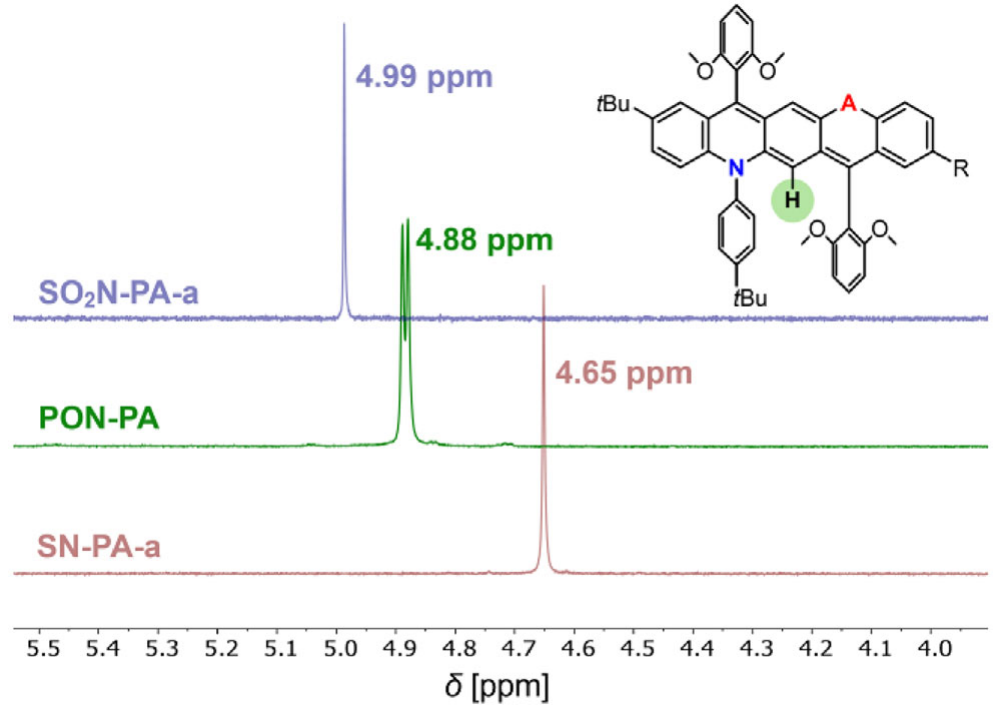
Comparison of the ^1^H NMR chemical shifts of the hydrogen atom at the C6 position (highlighted in green) in **SO_2_N‐PA‐a**, **PON‐PA**, and **SN‐PA‐a**, recorded in C_6_D_6_.

Ring currents can also be probed experimentally via NMR spectroscopy. For meaningful assessment, the selected nuclei should have similar chemical environments. In DA‐pentacenes, the central proton adjacent to the nitrogen atom is the most suitable probe for the pQDM ring as its other proton is more affected by shielding from varying acceptor groups. **SN‐PA‐a**, **SO_2_N‐PA‐a** and **PON‐PA** share two identical aryl substituents flanking this proton, and their ^1^H NMR spectra were recorded in the same solvent (C_6_D_6_), allowing reliable chemical shift comparison. As shown in Figure 7, the chemical shift increases from *δ*  =  4.65 ppm in the double‐donor compound **SN‐PA‐a** to *δ*  =  4.88 ppm in **PON‐PA** and *δ*  =  4.99 ppm in **SO_2_N‐PA‐a**, reflecting progressive deshielding and an enhanced diatropic ring current with increasing polarization of the pQDM core.

### 2.6 Electrochemical Characterization

**FIGURE 8:**
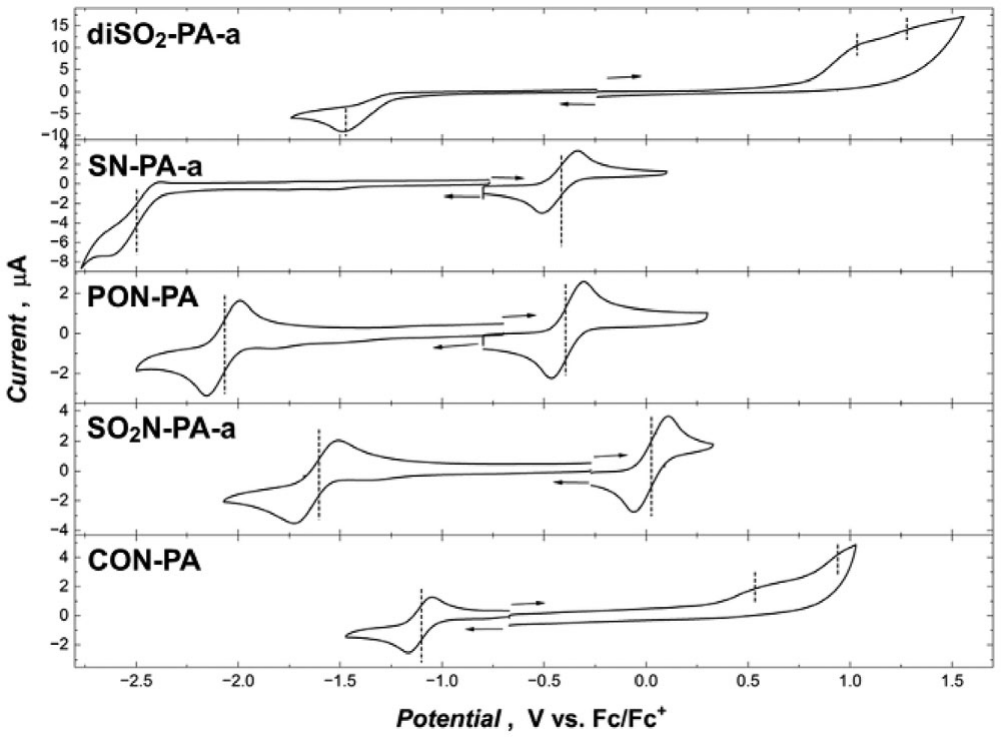
Cyclic voltammograms of pentacene analogues in 0.1 M (TBA)PF_6_ / o‐dichlorobenzene, limited to the first redox events.

The redox properties of the pentacene analogues were examined using cyclic and square‐wave voltammetry (Figure 8 and S52–S54). Most derivatives display reversible or quasi‐reversible first oxidation and reduction waves, whereas higher‐potential processes are typically irreversible and accompanied by product adsorption on the electrode surface.

In line with photophysical and theoretical predictions, the cyclic voltammograms reveal a pronounced narrowing of the HOMO‐LUMO gap from the double‐acceptor and double‐donor systems (*E*_g(CV)_ > 2 eV) to the donor‐acceptor derivatives (*E*_g(CV)_ = 1.4–1.7 eV, Figure 8 and Table 3). A similar trend is observed for the TIPS‐ethynyl‐substituted series, with the smallest bandgap of 1.32 eV found for **SO_2_N‐PA‐c** (Figure S53 and Table S13).

**TABLE 3:** Formal potentials of the first reversible anodic and cathodic redox processes of pentacene analogues measured in o‐dichlorobenzene.

Compound	*E* ^0^_a1_ ^a^ [V]	*E*°_c2_ ^a^ [V]	*E*_HOMO_ ^b^ [eV]	*E*_LUMO_ ^b^ [eV]	*E*_g(CV)_ ^c^ [eV]
**diSO_2_‐PA‐a**	0.826^d^	−1.234^d^	−5.54	−3.48	2.06
**SN‐PA‐a**	−0.419	−2.503^d^	−4.29	−2.21	2.08
**PON‐PA**	−0.388	−2.081	−4.32	−2.63	1.69
**SO_2_N‐PA‐a**	0.022	−1.616	−4.73	−3.09	1.64
**CON‐PA**	0.395^d^	−1.108	−5.01	−3.60	1.40
**SO_2_N‐PA‐c**	−0.093	−1.413	−4.62	−3.30	1.32

^a^ vs. Fc/Fc^+^.

^b^ Calculated using the energy of an electron in vacuum (−4.71 eV).

^c^ Electrochemical bandgap.

^d^ Irreversible process; onset potential is reported.

As expected, the presence of both donor and acceptor groups stabilizes the radical cations/anions, resulting in reversible oxidation and reduction processes for **PON‐PA** and **SO_2_N‐PA‐a** / **‐c**. In contrast, **CON‐PA** shows irreversible oxidation, likely due to its less bulky *N*‐alkyl substituent. Double‐donor analogues **SN‐PA‐a** (*E*_ox1,2_ = −0.42 V; +0.28 V) and—**SN‐PA‐c** (*E*_ox1,2_ = −0.13 V; +0.52 V) exhibit two‐step reversible oxidations, indicating the formation of stable radical cations and dications (Figures S52–S53, Table S13), similar to their symmetric N,N and S,S‐counterparts [9, 10, 24]. Noteworthy, the dications are isoelectronic with the parent pentacene.

The expected mirror behaviour—two step reversible reduction to dianions—was observed only for **diSO_2_‐PA‐c** (*E*_red1,2_ = −0.91 V; −1.42 V), while **diSO_2_‐PA‐a** shows no reversibility, illustrating the stabilizing effect of the silyl‐ethynyl substituents. Overall, the electrochemical data agree well with DFT‐predicted energy levels and optical bandgaps (*E*_g(opt)_ = 1.0–1.4 eV for DA‐PAs, Table S13).

## 3 Conclusion

In summary, we demonstrate that replacing the carbon atoms at positions 5 and 12 in pentacene with donor and acceptor groups yields air‐ and photostable derivatives featuring a highly polarized *para*‐quinodimethane (pQDM) core. With nitrogen as the donor and phosphine oxide, sulfone, or carbonyl as acceptors, the resulting DA‐pentacenes exhibit large dipole moments, markedly narrow HOMO‐LUMO gaps, and absorption and emission redshifted to the near‐infrared region, extending into the NIR‐II window.

Crucially, the donor–acceptor motif introduces partial zwitterionic character that *triggers* aromaticity in the central quinoidal ring in a polarization‐dependent manner, as confirmed by combined experimental (X‐ray, NMR) and theoretical (NICS, MIC, HOMA) analyses. The effect intensifies with increasing acceptor strength (P(O)Ph → SO_2_ → CO) and solvent polarity, but is absent in the double‐donor and double‐acceptor analogues.

Among the studied systems, the aza‐ketone **CON‐PA**—formally isoelectronic with pentacene—shows the most pronounced photophysical and electronic effects. Its diatropic ring current extends beyond the central ring, giving rise to weak global aromaticity that is strongly solvent‐dependent.

Altogether, these findings establish a molecular design principle in which controlled donor–acceptor polarization modulates aromaticity and electronic structure, providing a foundation for developing new functional acene analogues and tunable quinoidal π‐systems.

## Conflicts of Interest

The authors declare no conflicts of interest.

## Supporting information



The authors have cited additional references within the Supporting Information [24, 32, 45, 46, 47, 52, 53, 54, 55, 56, 57, 59, 63, 64, 65, 66, 67, 68, 69, 70, 71, 72, 73, 74, 75, 76, 77, 78, 79, 80, 81, 82, 83, 84, 85, 86, 87, 88].

**Supporting File (CIF files)**: anie71917‐sup‐0002‐Data.zip.

**Supporting File (DFT_Cartesian_coordinates)**: anie71917‐sup‐0003‐DFT_Cartesian_coordinates.zip.

## Data Availability

The data that support the findings of this study are available in the supporting information of this article.
