# Supplementary material for: Donor–Acceptor Pentacene Analogues With Near‐Infrared Emission and Tunable Aromaticity
Source: Angew Chem Int Ed Engl. 2026 Mar 30;65(20):e23436. doi: 10.1002/anie.202523436 (PMC13159433; doi:10.1002/anie.202523436)
Supplement: Supplementary file 1 — The authors have cited additional references within the Supporting Information [24, 32, 45, 46, 47, 52, 53, 54, 55, 56, 57, 59, 63, 64, 65, 66, 67, 68, 69, 70, 71, 72, 73, 74, 75, 76, 77, 78, 79, 80, 81, 82, 83, 84, 85, 86, 87, 88]. [file ANIE-65-e23436-s001.pdf]

Supporting Information

Donor–Acceptor Pentacene Analogues With Near-Infrared Emission and Tunable Aromaticity

Krzysztof Nowak, Olaf Morawski, Muhammad Yasir Mehboob, Maja Morawiak, Krzysztof Noworyta, Cina Foroutan-Nejad, Marek Grzybowski*

doi.org/10.1002/anie.202523436

Table of Contents

1. Experimental procedures 3
2. X-ray data 23
3. Photophysical properties 30
4. DFT calculations 48
5. Aromaticity 71
6. Cyclic voltammetry 86
7. Bibliography 89
8. NMR spectra 92
9. Mass spectra.. 115

1. Experimental procedures

General. NMR spectra were recorded on 500 MHz (BRUKER or Varian) or 600 MHz (Varian) spectrometers. Chemical shifts in the ^{1}H and ^{13}C NMR spectra are reported in *δ* (ppm) using residual solvents signals as internal standards. Mass spectra were recorded on a Waters Synapt GS-2 Mass Spectrometer using the APCI ionization method. Flash column chromatography was performed on a Teledyne Isco CombiFlash NextGen 300+ apparatus using gel (20–40 µm, pore size 60 Å, 400–632 mesh; or 40–60 µm, pore size 55–60 Å, 230–400 mesh) as the stationary phase. Manual column chromatography was carried out on silica gel (pore size 60 Å, 230-400 mesh).

Single crystal X-ray diffraction experiments were performed on an Agilent SuperNova, Dual (Cu source) or XtaLAB Synergy, Dualflex, HyPix-Arc 150 diffractometer. Structures were solved using *Olex2*,[1] with the SHELXS[2] structure solution program *via* Direct Methods and refined using the *olex2.refine*[3] package with Gauss-Newton minimization or using ShelXL 2018/3[4] with Least Squares minimization.

UV-Vis-NIR absorption spectra were recorded on a Shimadzu UV-3600i Plus UV-Vis-NIR spectrophotometer or a Perkin Elmer Lambda 35 UV/Vis spectrophotometer. Fluorescence spectra of the NIR-emissive compounds were collected on an Edinburgh Instruments FLS1000 spectrofluorometer using a 658.7 nm diode laser (30.0 mW) as an external excitation source, while spectra of the remaining derivatives were collected on an Edinburgh Instruments FS5 spectrofluorometer. All emission spectra and fluorescence quantum yields were measured in air-equilibrated solvents. Control experiments confirmed that removal of dissolved oxygen by bubbling nitrogen for 30 min did not affect the emission intensities of representative samples.

All electrochemical measurements were carried out using a Gamry Reference 600 potentiostat (Gamry Instruments, USA) operated with the Gamry Framework software (v7.8.2), with the ferrocene/ferrocenium (Fc/Fc^{+}) redox couple as an internal standard.

3D molecular models based on X-ray crystal structures and DFT-optimized geometries were visualized using *Mercury* software.[5] Two-dimensional projections of the π-systems including bond lengths and/or dipole moments, were generated using custom Python scripts (*xyz2BL.py* and *DipoleVisBL.py*) available at: https://github.com/mrkgrb/DFT-visualizer

Unless otherwise noted, all reactions were performed under an argon atmosphere in anhydrous solvents. Schlenk vessels were dried prior to use by heating with a heat gun under vacuum. All commercial reagents were used as received. Compounds **3**,[6] **S3**,[7] **16**,[8] **17c**[8] and **11**[9] were synthesized according to literature procedures.

List of abbreviations and common names used:

ACN, MeCN	–	acetonitrile
cHex	–	cyclohexane
DCE	–	1,2-dichloroethane
DCM	–	dichloromethane
DDQ	–	2,3-dichloro-5,6-dicyano-1,4-benzoquinone
DIPA	–	diisopropylamine
DIPEA	–	diisopropylethylamine
DMF	–	dimethylformamide
DMSO	–	dimethyl sulfoxide
Eaton's reagent	–	10 wt% phosphorus pentoxide solution in methanesulfonic acid
EtOH	–	ethanol
Hex	–	*n*-hexane
iPrOH	–	isopropanol, 2-propanol
mCPBA	–	*meta*-chloroperoxybenzoic acid
MeOH	–	methanol
pQDM	–	*para*-quinodimethane
rt	–	room temperature
TFA	–	trifluoroacetic acid
THF	–	tetrahydrofuran

Synthesis

1st synthesis of CON-PA. To synthesize the nitrogen-carbonyl analogue **CON-PA**, we initially attempted a route analogous to those used for azasulfone and azaphosphone analogues, starting from terephthalic diacetal **S3** instead of diester **3** (Scheme S1). Buchwald-Hartwig amination of **S3** with bis(4-tert-butylphenyl)amine failed, likely due to steric hindrance. However, the same reaction with simple aniline proceeded efficiently, and subsequent *N*-butylation afforded the unsymmetrical terephthalic diacetal **S4** in 77% yield. Suzuki coupling of **S4** with benzylboronic acid pinacol ester (BnBpin), followed by acetal deprotection, gave dialdehyde **S6** in good overall yield. Addition of an organolithium reagent to **S6**, followed by electrophilic cyclization of the resulting diol in the presence of boron trifluoride, successfully furnished precursor **S7**, which contains the desired polycyclic scaffold. Conversion of **S7** into the target acene analogue required a two-stage oxidation: (a) oxidation of the methylene group to a carbonyl, and (b) dehydrogenation of the fused para-xylene core to the fully conjugated pQDM backbone. After screening a range of oxidants, we found that **CON-PA** could be obtained in very low yield (4%) using DDQ in dichloromethane/methanol. The major product under these conditions was the more favored 5-azapentacene cation **S8** (Scheme S1).

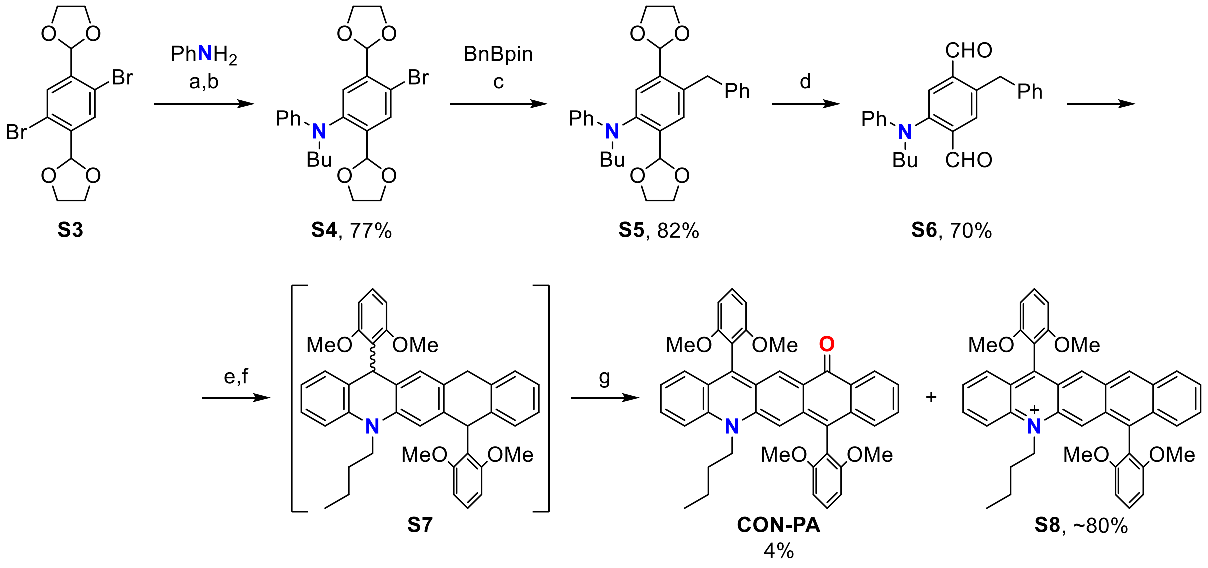

Scheme S1. Reaction conditions: a) $Pd(OAc)_2$, XantPhos, K_3PO_4, toluene, 120 °C; b) *n*-BuBr, KOH, DMF, 50 °C; c) $Pd(dppf)Cl_2$, Na_2CO_3 ($2M_{aq}$), 1,4-dioxane, H_2O, 100 °C; d) p-toluenesulfonic acid (10 mol%), MeCN, H_2O, 65 °C; e) RLi, THF, 0 °C to rt; f) $BF_3{\cdot}Et_2O$, DCM, 0 °C to rt; g) DDQ, DCM, MeOH, rt.

Dimethyl 2,5-dibromo terephthalate (3)

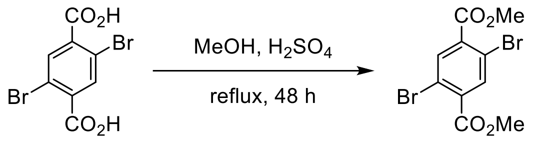

3, 90%

Following a literature procedure,[6] 2,5-dibromoterephthalic acid (20.74 g, 64.0 mmol) was suspended in methanol (400 mL) with sulfuric acid (4.0 mL) and refluxed (oil bath temperature: 90 °C) for 48 h. After cooling the reaction mixture in a refrigerator, the precipitate was filtered off, washed with methanol, and dried to afford ester **3** (20.27 g, 57.6 mmol, 90%) as an off-white crystalline solid. ^{1}H NMR (500 MHz, $CDCl_3$) δ 8.05 (s, 2H), 3.95 (s, 6H). ^{13}C NMR (126 MHz, $CDCl_3$) δ 164.7, 136.8, 135.6, 120.3, 53.1. HRMS (APCI): m/z calcd for $C_{10}H_8O_4Br_2+H^+$: 350.8867 $[M+H]^+$, found: 350.8867.

Dimethyl 2-(bis(4-(*tert*-butyl)phenyl)amino)-5-bromoterephthalate (4)

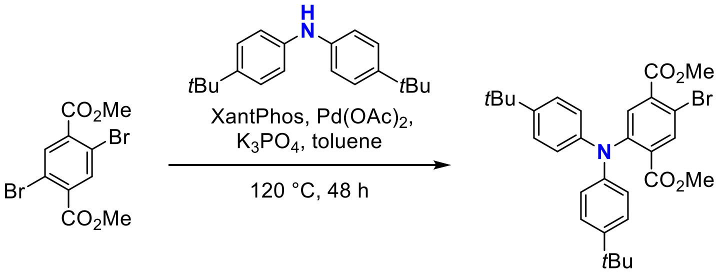

3 **4**, 74%

Ester **3**[6] (5.28 g, 15.0 mmol, 1.5 eq), bis(4-(*tert*-butyl)phenyl)amine (2.81 g, 10.0 mmol, 1.0 eq), palladium(II) acetate (168 mg, 0.75 mmol, 7.5 mol%), XantPhos (651 mg, 1.13 mmol, 11.3 mol%) and anhydrous tripotassium phosphate (4.25 g, 20.0 mmol, 2.0 eq) were placed in a Schlenk flask. The flask was evacuated and backfilled with argon three times, followed by the addition of anhydrous toluene (100 mL). The flask was again carefully evacuated and backfilled with argon three times. The flask was sealed and the reaction mixture was stirred at 120 °C for 48 h. After cooling, the reaction mixture was diluted with water and extracted three times with chloroform. The combined organic layers were washed with brine, dried over anhydrous Na_2SO_4 and filtered. Celite (ca. 10 g) was added to the filtrate, and the solvents were evaporated to dryness. The resulting powder was subjected to flash column chromatography (silica gel, hexanes/ethyl acetate 99/1 to 95/5) to give crude **4** as an orange oil. Dilution with methanol (20 mL) followed by cooling in a refrigerator induced precipitation of the product. Filtration afforded product **4** (4.09 g, 7.40 mmol, 74%) as a yellow powder with an orange-yellow fluorescence. ^{1}H NMR (500 MHz, CD_2Cl_2) δ 7.82 (s, 1H), 7.48 (s, 1H), 7.29 – 7.22 (m, 4H), 6.93 – 6.86 (m, 4H), 3.84 (s, 3H), 3.34 (s, 3H), 1.30 (s, 18H). ^{13}C NMR (126 MHz, CD_2Cl_2) δ 166.2 (2 signals), 146.5, 146.4, 145.0, 136.5, 136.3, 132.1, 130.6, 126.5, 123.2, 114.2, 53.0, 52.3, 34.6, 31.5. HRMS (APCI): *m*/*z* calcd for $C_{30}H_{34}NO_4Br+H^+$: 552.1749 $[M+H]^+$, found: 552.1753.

Dimethyl 2-(bis(4-(*tert*-butyl)phenyl)amino)-5-((4-(*tert*-butyl)phenyl)thio)terephthalate (S1)

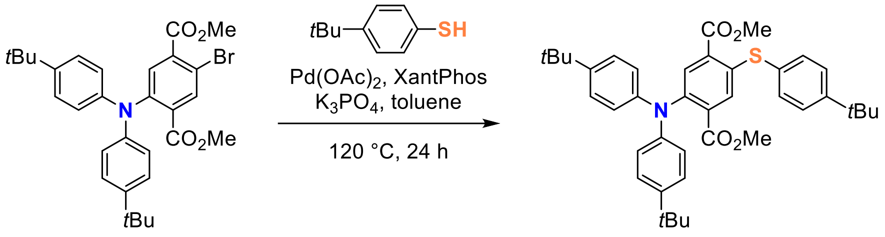

Compound **4** (4.09 g, 7.40 mmol, 1.0 eq), palladium (II) acetate (166 mg, 0.740 mmol, 10 mol%), XantPhos (482 mg, 0.833 mmol, 11.3 mol%) and anhydrous tripotassium phosphate (980 g, 4.62 mmol, 0.62 eq) were placed in a Schlenk flask. The flask was evacuated and backfilled with argon three times, followed by the addition dry toluene (80 mL). The flask was again carefully evacuated and backfilled with argon three times. 4-*tert*-Butylbenzenethiol (1.60 mL, 9.25 mmol, 1.25 eq) was then added, and the flask was sealed. The reaction mixture was stirred at 120 °C for 24. After cooling, the mixture was diluted with water and extracted three times with chloroform. The combined organic layers were washed with brine, and dried over anhydrous Na_2SO_4. The drying agent was filtered off, Celite (ca. 10 g) was added to the filtrate, and the solvents were evaporated to dryness. The resulting residue was subjected to column chromatography (silica, toluene as eluent) to afford crude **S1** as orange oil. The product was recrystallized by slow addition of methanol (20 mL) to a hot solution of **S1** in chloroform (8 mL) and iPrOH (1 mL), yielding **S1** (4.65 g, 7.29 mmol, 99%) as a yellow solid.

Note: 4 and **S1** exhibit the same color and have similar R_f in various eluents; however, they differ in fluorescence intensity under UV light, with **S1** showing significantly stronger emission.

^1H NMR (500 MHz, CD_2Cl_2) δ 7.69 (s, 1H), 7.47 (s, 1H), 7.26 – 7.20 (m, 1H), 7.06 (s, 1H), 6.90 – 6.85 (m, 1H), 3.84 (s, 1H), 3.24 (s, 1H), 1.35 (s, 3H), 1.29 (s, 6H). ^{13}C NMR (126 MHz, CD_2Cl_2) δ 167.0, 166.4, 153.0, 145.9, 145.2, 143.7, 137.3, 134.9, 132.9, 131.7, 131.1, 130.8, 129.4, 127.3, 126.3, 122.7, 52.7, 52.1, 35.1, 34.5, 31.6, 31.4. HRMS (APCI): *m/z* calcd for $C_{40}H_{47}NO_4S+H^+$: 638.3304 $[M+H]^+$, found: 638.3309.

2-(bis(4-(*tert*-butyl)phenyl)amino)-5-((4-(*tert*-butyl)phenyl)thio)terephthalic acid (5)

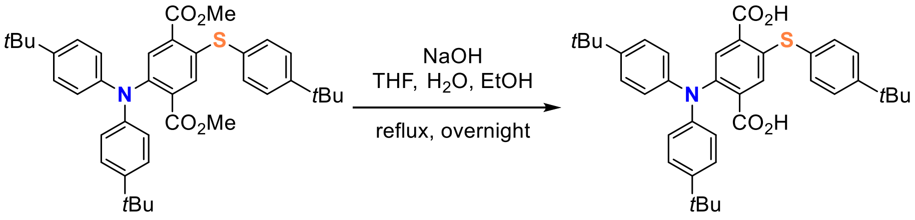

In a 250 mL round-bottom flask, compound **S1** (2.05 g, 3.22 mmol) was dissolved in a mixture of tetrahydrofuran (20 mL) and ethanol (12 mL), followed by the addition of 8M aqueous sodium hydroxide (12 mL). The flask was sealed with a septum fitted with an air-filled balloon and the mixture was gently refluxed overnight. After cooling to room temperature, the reaction mixture was concentrated under reduced

pressure. The remaining aqueous phase was diluted with water and acidified to approximately pH 2 with 2M hydrochloric acid. The resulting orange precipitate was filtered off, washed with water (3x), and dried under vacuum at 110 °C for 4 h. Crude acid **5** was obtained quantitatively as an orange powder and used in subsequent steps without further purification.

Note: During prolonged heating under strongly basic conditions, the joint between the flask and the reflux condenser may become fused, making the glassware inseparable. To prevent this, it is strongly recommended to use a large round-bottom flask without a reflux condenser and to apply mild reflux conditions.

Spectral data for crude product **5**: ^{1}H NMR (500 MHz, DMSO-*d*$_6$) δ 7.60 (s, 1H), 7.56 – 7.49 (m, 4H), 7.26 – 7.19 (m, 4H), 6.99 (s, 1H), 6.80 – 6.75 (m, 4H), 1.29 (s, 9H), 1.22 (s, 18H). HRMS (APCI): *m/z* calcd for $C_{38}H_{43}NO_4S+H^+$: 610.2991 [M+H]$^+$, found: 610.2994.

2,9-di-*tert*-butyl-12-(4-(*tert*-butyl)phenyl)-7H-thiochromeno[2,3-b]acridine-7,14(12H)-dione (6)

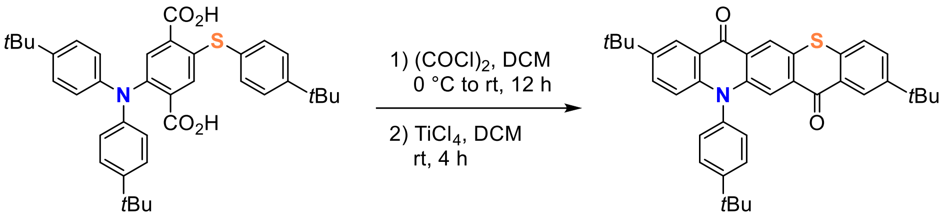

5 **6**, 85%

To a stirred suspension of acid **5** (2.14 g, 3.50 mmol, 1.0 eq) in dichloromethane (20 mL) at 0 °C (ice bath) under an argon atmosphere, oxalyl chloride (1.80 mL, 21.0 mmol, 6.0 eq) was slowly added *via* a syringe, followed by a few drops of DMF to initiate the reaction. The reaction was stirred at 0 °C for 30 min, after which the ice bath was removed and stirring was continued at room temperature overnight. All volatiles were removed under reduced pressure, and the resulting red residue was dried under vacuum for 30 min. The residue was then dissolved in dichloromethane (30 mL) and stirred under argon. Titanium(IV) chloride (3.9 mL, 35.0 mmol, 10.0 eq) was added dropwise, and the mixture was stirred vigorously for 3 h at room temperature. The reaction was quenched by the dropwise addition of water (30 mL) and dichloromethane. The layers were separated, and the aqueous layer was extracted with dichloromethane (5x). The combined organic layers were washed with brine, dried over anhydrous Na_2SO_4, and filtered. Celite (ca. 5 g) was added to the filtrate, and the solvents were evaporated to dryness. The residue was subjected to flash column chromatography (hexanes/ethyl acetate 99/1 to 95/5). The crude product was recrystallized from methanol to give **6** (1.70 g, 2.96 mmol, 85%) as an orange powder with a bright yellow fluorescence. ^{1}H NMR (500 MHz, CD_2Cl_2) δ 8.78 (d, J = 0.7 Hz, 1H), 8.49 (d, J = 2.6 Hz, 1H), 8.48 (d, J = 2.3 Hz, 1H), 8.05 (d, J = 0.6 Hz, 1H), 7.82 – 7.76 (m, 2H), 7.71 (dd, J = 8.5, 2.3 Hz, 1H), 7.64 (dd, J = 9.0, 2.5 Hz, 1H), 7.56 (d, J = 8.4 Hz, 1H), 7.38 – 7.33 (m, 2H), 6.80 (d, J = 9.0 Hz, 1H), 1.51 (s, 9H) 1.40 (s, 9H), 1.37 (s, 9H). ^{13}C NMR (126 MHz, CD_2Cl_2) δ 180.2, 177.6, 153.6, 149.9, 145.2, 142.6, 141.4, 136.2, 135.4, 132.7, 132.3, 131.2,

129.7, 128.6, 128.4 (2 signals), 126.3, 126.2, 125.5, 124.6, 122.9, 121.5, 118.5, 117.5, 35.4, 35.2, 34.9, 31.6, 31.4, 31.3. HRMS (APCI): *m/z* calcd for $C_{38}H_{39}NO_2S$+H$^+$: 574.2780 [M+H]$^+$, found: 574.2782.

2,9-di-*tert*-butyl-12-(4-(*tert*-butyl)phenyl)-7H-thiochromeno[2,3-b]acridine-7,14(12H)-dione 5,5-dioxide (7)

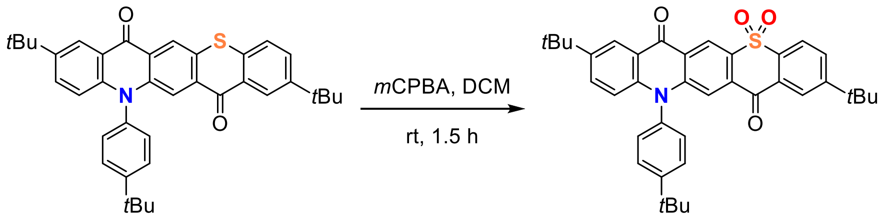

6 **7**, 93%

In a round-bottom flask, compound **6** (230 mg, 0.400 mmol) was dissolved in dichloromethane (50 mL). *meta*-chloroperoxybenzoic acid (*m*CPBA, 197 mg, 77%$_{wt}$, 0.880 mmol, 2.2 eq) was added under an argon flow, and the flask was sealed with a septum fitted with an argon-filled rubber balloon. The reaction mixture was stirred at room temperature for 1.5 h, then quenched with water (50 mL) and extracted three times with dichloromethane. The combined organic layers were washed with brine, dried over anhydrous Na_2SO_4 and filtered. The solvents were removed under reduced pressure, and the residue was recrystallized from methanol to afford pure product **7** (226 mg, 0.373 mmol, 93%) as a bright yellow powder. ^{1}H NMR (500 MHz, CD_2Cl_2) δ 9.25 (s, 1H), 8.53 (d, J = 2.4 Hz, 1H), 8.22 (d, J = 2.0 Hz, 1H), 8.11 (d, J = 8.3 Hz, 1H), 7.92 (ddd, J = 8.3, 2.1, 0.7 Hz, 1H), 7.83 – 7.76 (m, 2H), 7.71 – 7.67 (m, 2H), 7.37 – 7.30 (m, 2H), 6.82 (d, J = 9.0 Hz, 1H), 1.50 (s, 9H), 1.40 (s, 9H), 1.37 (s, 9H). ^{13}C NMR (126 MHz, CD_2Cl_2) δ 179.7, 176.9, 157.8, 154.2, 147.1, 145.3, 142.2, 139.1, 135.5, 134.3, 133.0, 132.6, 132.5, 131.6, 129.4, 128.8, 126.5, 125.2, 123.9, 123.7, 123.1, 122.6, 119.1, 118.1, 35.9, 35.4, 35.0, 31.5, 31.4, 31.0. HRMS (APCI): *m/z* calcd for $C_{38}H_{39}NO_4S$+H$^+$: 606.2678 [M+H]$^+$, found: 606.2684.

Dimethyl 2-(bis(4-(*tert*-butyl)phenyl)amino)-5-(diphenylphosphoryl)terephthalate (S2)

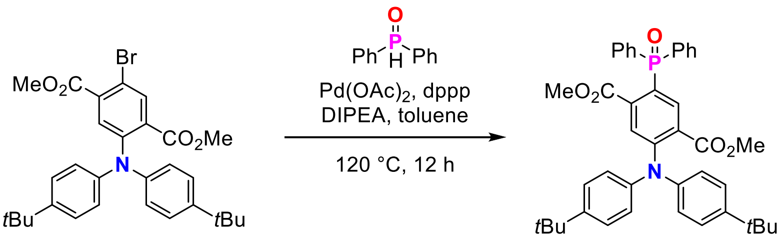

4 **S2**, 91%

Compound **4** (551 mg, 0.997 mmol, 1.2 eq), palladium(II) acetate (19 mg, 0.083 mmol, 10 mol%), 1,3-bis(diphenylphosphino)propane (dppp, 42 mg, 0.100 mmol, 12 mol%) and diphenylphosphine oxide (168 mg, 0.831 mmol, 1.0 eq) were placed in a Schlenk flask. The flask was evacuated and backfilled with argon three times, followed by the addition of dry toluene (15 mL) and diisopropylethylamine (DIPEA, 580 µL,

3.3 mmol, 4.0 eq). The flask was sealed, and the reaction mixture was stirred at 120 °C for 12 h. After cooling, the reaction was quenched with water (50 mL) and extracted three times with chloroform. The combined organic layers were washed with brine, dried over anhydrous Na_2SO_4 and filtered. Celite (ca. 2 g) was added to the filtrate, and the solvents were removed under reduced pressure. The resulting residue was subjected to flash column chromatography (dichloromethane/methanol 99/1 to 9/1). The crude product was recrystallized from n-hexane to afford compound **S2** (510 mg, 0.757 mmol, 91%) as an orange powder. ^{1}H NMR (500 MHz, CD_2Cl_2) δ 7.70 – 7.65 (m, 4H), 7.58 – 7.47 (m, 7H), 7.45 (d, J = 3.1 Hz, 1H), 7.31 – 7.27 (m, 4H), 6.97 – 6.93 (m, 4H), 3.39 (s, 3H), 3.20 (s, 3H), 1.30 (s, 18H). ^{13}C NMR (126 MHz, CD_2Cl_2) δ 167.7 (d, J_{CP} = 2.6 Hz), 167.1, 150.1 (d, J_{CP} = 2.7 Hz), 147.6, 144.8, 139.8 (d, J_{CP} = 7.0 Hz), 137.8 (d, J_{CP} = 12.3 Hz), 133.9 (d, J_{CP} = 107.6 Hz), 132.1 (d, J_{CP} = 6.7 Hz), 132.0, 128.8 (d, J_{CP} = 12.4 Hz), 127.8 (d, J_{CP} = 12.2 Hz), 127.7 (d, J_{CP} = 8.8 Hz), 126.6, 124.7 (d, J_{CP} = 103.5 Hz), 124.2, 52.5, 52.0, 34.7, 31.5. ^{31}P{^{1}H} NMR (202 MHz, $CDCl_3$) δ 29.7. HRMS (APCI): *m/z* calcd for $C_{42}H_{44}NO_5P+H^+$: 674.3035 [M+H]$^+$, found: 674.3038.

Dimethyl 2-(bis(4-(*tert*-butyl)phenyl)amino)-5-(diphenylphosphoryl)terephthalate (8)

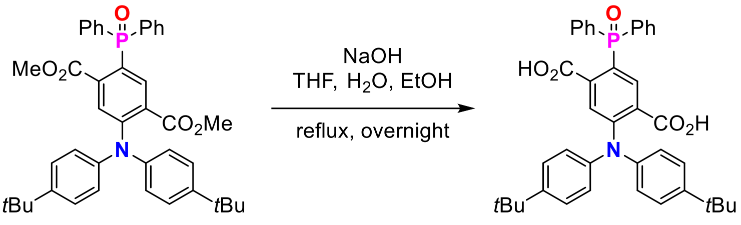

In a 100 mL round-bottom flask, compound **S2** (716 mg, 1.06 mmol) was dissolved in a mixture of tetrahydrofuran (8 mL) and ethanol (4 mL), then of 8M aqueous sodium hydroxide (4 mL). The flask was sealed with a septum fitted with an air-filled balloon and the mixture was gently refluxed overnight. After cooling to room temperature, the reaction mixture was concentrated under reduced pressure. The remaining aqueous phase was diluted with water and acidified to approximately pH 2 with 2M hydrochloric acid. The resulting orange precipitate was filtered off, washed with water (3x), and dried under vacuum at 110 °C for 4 h. Crude acid **8** (561 mg, 0.869 mmol, 82%) was obtained as a brown powder and used in subsequent steps without further purification.

Note: During prolonged heating under strongly basic conditions, the joint between the flask and the reflux condenser may become fused, making the glassware inseparable. To prevent this, it is strongly recommended to use a large round-bottom flask without a reflux condenser and to apply mild reflux conditions.

Spectral data for crude product **8**: ^{1}H NMR (500 MHz, DMSO-d_6) δ 7.63 (d, J = 13.6 Hz, 1H), 7.61 – 7.55 (m, 4H), 7.54 – 7.42 (m, 7H), 7.31 – 7.24 (m, 4H), 6.86 – 6.80 (m, 4H), 1.25 (s, 18H). HRMS (APCI): *m/z* calcd for $C_{40}H_{40}NO_5P+H^+$: 646.2722 [M+H]$^+$, found: 646.2726.

2-(*tert*-Butyl)-5-(4-(*tert*-butyl)phenyl)-12-phenyl-5-hydrophosphinolino[2,3-*b*]acridine-7,14-dione 12-oxide (10)

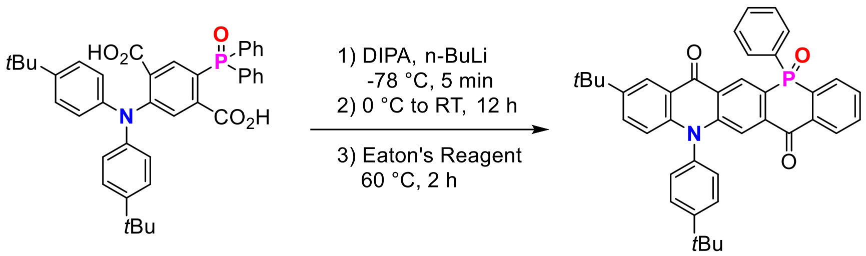

8 **10**, 69%

Under an argon atmosphere, a solution of diisopropylamine (DIPA, 190 µL, 1.36 mmol, 6.8 eq) in tetrahydrofuran (8 mL) was cooled to −78 °C using a dry ice/acetone bath. *n*-Butyllithium (2.5M in hexanes, 550 µL, 1.36 mmol, 6.8 eq) was slowly added. The mixture was stirred at −78 °C for 5 min, then the cooling bath was removed, allowing the reaction to gradually warm to 0 °C (judged by the moment when frost on the bottom of the flask began to melt). The flask was then placed in an ice bath and stirring was continued for 20 min. Diacid **8** (129 mg, 0.200 mmol, 1.0 eq) was added under an argon flow, and the reaction mixture was stirred for 12 h. During this time, the ice in the bath melted, and the reaction mixture gradually reached room temperature. The reaction was quenched with saturated aqueous ammonium chloride and extracted three times with chloroform. The combined organic layers were washed with brine, dried over anhydrous Na_2SO_4. The drying agent was removed by filtration and the solvents were evaporated to dryness.

To the resulting residue, Eaton's reagent (2 mL) was added, and the solution was stirred at 60 °C for 2 h. The reaction was quenched by careful addition of of 1M aqueous NaOH (5 mL) and water (5 mL), followed by extraction with chloroform (3x). The combined organic layers were washed with brine, dried over anhydrous Na_2SO_4 and filtered. The drying agent was filtered off, and the product was purified by column chromatography (dichloromethane/methanol 19/1) and recrystallized from chloroform/*n*-hexane to afford compound **10** (84 mg, 0.138 mmol, 69%) as a yellow powder with green fluorescence. ^{1}H NMR (500 MHz, CD_2Cl_2) δ 9.09 (d, J = 13.5 Hz, 1H), 8.49 (d, J = 2.5 Hz, 1H), 8.31 (ddd, J = 8.0, 4.7, 1.4 Hz, 1H), 8.03 (ddd, J = 12.7, 7.3, 1.5 Hz, 1H), 7.87 (d, J = 4.0 Hz, 1H), 7.82 – 7.75 (m, 3H), 7.72 (tt, J = 7.4, 1.3 Hz, 1H), 7.66 (dd, J = 9.0, 2.5 Hz, 1H), 7.59 (ddd, J = 12.8, 8.4, 1.4 Hz, 2H), 7.48 – 7.43 (m, 1H), 7.41 – 7.36 (m, 2H), 7.36 – 7.33 (m, 2H), 6.79 (d, J = 9.0 Hz, 1H), 1.51 (s, 9H), 1.38 (s, 9H). ^{13}C NMR (126 MHz, cd_2cl_2) δ 183.1 (2 signals), 177.2, 154.0, 146.5, 145.4, 142.3, 138.5 (2 signals), 136.4 (2 signals), 135.8, 135.2, 134.8, 134.5, 134.4, 133.9, 133.0 (2 signals), 132.9, 132.7, 132.2 (2 signals), 131.6, 131.5, 131.3, 131.2, 129.5 (2 signals), 129.2, 129.1, 128.7, 128.6, 124.8, 124.3, 124.2, 124.0, 123.0, 122.6, 118.9, 118.8, 118.0, 35.4, 34.9, 31.6, 31.4. ^{31}P{^{1}H} NMR (202 MHz, CD_2Cl_2) δ 2.39. HRMS (APCI): *m/z* calcd for $C_{40}H_{36}NO_3P+H^+$: 610.2511 $[M+H]^+$, found: 610.2516. ^{31}P–^{13}C coupling complicates the ^{13}C NMR spectrum and hinders full signal assignment. Therefore, only a list of all observed peaks is provided.

General procedure for the synthesis of acene analogues with aryl side groups

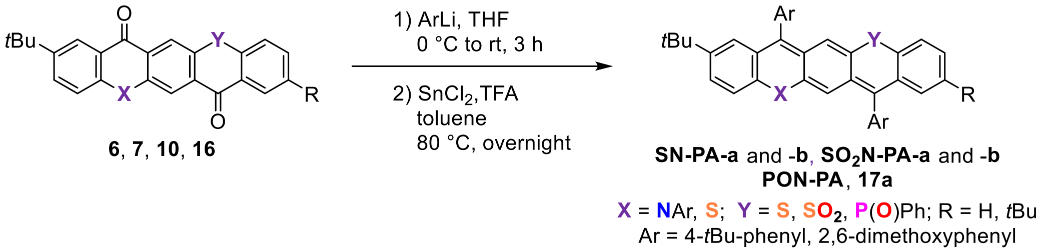

Under an argon atmosphere, in a heat-dried Schlenk flask (heat gun/vacuum), bromoarene (1.00 mmol, 10.0 eq) was dissolved in tetrahydrofuran (3.0 mL) and the solution was cooled to −78 °C. *tert*-Butyllithium (*t*BuLi, 1.86M in *n*-pentane, 1.1 mL, 2.0 mmol, 20.0 eq) was added dropwise *via* syringe. The reaction mixture was stirred at −78 °C for 10 min, after which the cooling bath was removed and the mixture was allowed to warm to 0 °C. Stirring was continued at this temperature for 30 min. Diketone (0.100 mmol, 1.0 eq) was added under an argon flow, and the reaction was stirred at room temperature for 3 h. The reaction was quenched with water (5 mL) and extracted three times with chloroform. The combined organic layers were washed with brine, dried over anhydrous Na_2SO_4 and filtered. The solvents were evaporated to dryness under reduced pressure.

The resulting crude diol was transferred to another Schlenk flask and suspended in anhydrous toluene (10 mL). Anhydrous tin(II) chloride (379 mg, 2.00 mmol, 20.0 eq) and trifluoroacetic acid (0.42 mL) were added, the flask was sealed with a septum fitted with an argon-filled balloon and stirred overnight at 80 °C. The reaction was quenched by the addition of water (20 mL) and 2M hydrochloric acid (5 mL), and the mixture was extracted three times with chloroform. The combined organic layers were washed with acidic brine (ca. 20 mL $2M_{aq}$ HCl per 30 mL of brine), followed by saturated aqueous sodium bicarbonate to neutralize the acid. The organic phase was dried over anhydrous Na_2SO_4 and filtered. Unless otherwise noted, Celite (ca. 1 g) was added to the filtrate, and the solvents were evaporated to dryness under reduced pressure. The resulting residue was subjected to flash column chromatography. Purification conditions for each compound are specified below.

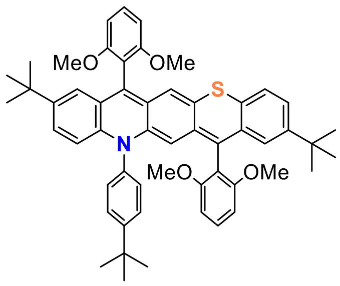

SN-PA-a. Synthesized following the general procedure from 1-bromo-2,6-dimethoxybenzene (217 mg, 1.00 mmol) and diketone **6** (57 mg, 0.100 mmol). The product was purified by flash column chromatography (silica gel, hexanes/ethyl acetate 4/1), then recrystallized from chloroform/methanol to afford **SN-PA-a** (40 mg, 0.040 mmol, 49%) as a dark purple powder. ^{1}H NMR (500 MHz, C_6D_6, 323 K) δ 7.21 (t, *J* = 8.3 Hz, 1H), 7.12 – 7.09 (m, 2H), 7.05 (t, *J* = 8.2 Hz, 1H), 6.91 (d, *J* = 2.2 Hz, 1H), 6.75 (d, *J* = 1.4 Hz, 1H), 6.69 –

6.65 (m, 2H), 6.65 – 6.60 (m, 3H), 6.47 (d, *J* = 8.3 Hz, 2H), 6.40 (s, 1H), 6.32 (d, *J* = 8.3 Hz, 2H), 5.96 (d, *J* = 8.7 Hz, 1H), 4.65 (s, 1H), 3.31 (s, 6H), 3.27 (s, 6H), 1.24 (s, 9H), 1.08 (s, 9H), 1.03 (s, 9H). ^{13}C NMR (126 MHz, C_6D_6, 323 K) δ 159.5, 159.4, 150.8, 148.1, 143.6, 143.2, 140.8, 137.7, 135.1, 133.9, 133.3, 130.5, 129.8, 129.7, 128.7, 127.5, 127.4, 125.8, 124.7, 124.3, 123.3, 123.2, 122.2, 122.0, 119.7, 117.4, 114.8, 114.4, 113.8, 104.7, 104.6, 99.6, 55.7, 55.6, 34.6, 34.3, 34.0, 31.6, 31.4, 31.2. HRMS (APCI): *m/z* calcd for $C_{54}H_{57}NO_4S+H^+$: 816.4087 $[M+H]^+$, found: 816.4073.

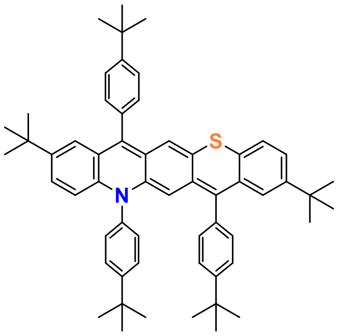

SN-PA-b. Synthesized following the general procedure from 1-bromo-4-*tert*-butylbenzene (173 µL, 1.00 mmol) and diketone **6** (57 mg, 0.100 mmol). The product was purified by flash column chromatography (silica gel, hexanes/ethyl acetate 4/1), then recrystallized from chloroform/methanol to afford **SN-PA-b** (28 mg, 0.034 mmol, 34%) as a dark purple powder. ^{1}H NMR (500 MHz, C_6D_6, 323 K) δ 7.40 – 7.36 (m, 2H), 7.34 – 7.30 (m, 2H), 7.21 – 7.18 (m, 4H), 7.01 – 6.98 (m, 2H), 6.97 (d, *J* = 2.2 Hz, 1H), 6.96 – 6.93 (m, 2H), 6.70 – 6.61 (m, 3H), 6.53 (s, 1H), 6.43 (d, *J* = 2.0 Hz, 1H), 5.92 (d, *J* = 8.7 Hz, 1H), 5.20 (s, 1H), 1.30 (s, 9H), 1.26 (s, 9H), 1.22 (s, 9H), 1.05 (s, 9H), 0.98 (s, 9H). ^{13}C NMR spectrum could not be recorded due to aggregation. HRMS (APCI): *m/z* calcd for $C_{58}H_{65}NS+H^+$: 808.4916 $[M+H]^+$, found: 808.4901.

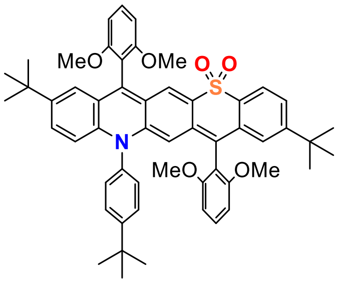

SO_2N-PA-a. Synthesized following the general procedure from 1-bromo-2,6-dimethoxybenzene (217 mg, 1.00 mmol) and diketone **7** (61 mg, 0.100 mmol). After extraction, drying with Na_2SO_4, filtration and evaporation of the solvents, the crude product was precipitated by the addition of methanol to the obtained residue. The solid was collected by filtration and recrystallized from toluene/n-hexane (0.5 mL / 5 mL) to afford **SO_2N-PA-a** (41 mg, 0.048 mmol, 48%) as a green crystalline solid.

The reaction was also performed on a larger scale, following the same procedure with the following quantities of reagents: diketone **7** (212 mg, 0.350 mmol), 1-bromo-2,6-dimethoxybenzene (760 mg, 3.50 mmol), *t*BuLi (1.7M in *n*-pentane, 2.1 mL, 3.6 mmol, 10 eq), THF (10 mL). For the reduction step: $SnCl_2$ (1.33 g, 7.01 mmol), TFA (1.5 mL), toluene (30 mL). After recrystallization from toluene/n-hexane, **SO_2N-PA-a** was

obtained (245 mg, 0.267 mmol, 76%) as a green crystalline solid being a solvate with toluene in a 4:3 molecular ratio, as estimated from ^{1}H NMR spectrum.

^{1}H NMR (500 MHz, C_6D_6, 323 K) δ 8.11 (s, 1H), 8.08 (d, J = 8.2 Hz, 1H), 7.22 (d, J = 2.3 Hz, 1H), 7.19 (t, J = 8.4 Hz, 1H), 7.09 (d, J = 1.9 Hz, 1H), 7.06 (t, J = 8.3 Hz, 1H), 6.86 – 6.78 (2×dd, J = ~8.5, 2.1 Hz, 2×1H), 6.71 – 6.66 (m, AA'BB', 2H), 6.42 (d, J = 8.4 Hz, 2H), 6.32 (d, J = 8.3 Hz, 2H), 6.17 (d, J = 8.8 Hz, 1H), 4.99 (s, 1H), 3.28 (s, 6H), 3.20 (s, 6H), 1.25 (s, 9H), 1.04 (s, 9H), 1.00 (s, 9H) (one AA'BB' signal (2H) is obscured by the residual C_6D_6 peak).^{13}C NMR (126 MHz, C_6D_6, 323 K) δ 159.8, 159.1, 154.5, 151.6, 143.6, 141.8, 141.3, 138.7, 137.5, 137.3, 135.9, 131.8, 131.7, 131.2, 129.5, 129.1, 127.7, 127.6, 126.7, 124.2, 123.9, 123.6, 123.5, 121.7, 116.7, 114.3, 113.9, 112.6, 104.9, 104.8, 100.0, 55.8, 55.7, 34.9, 34.7, 34.1, 31.6, 31.2, 31.1 (one aromatic signal is missing, probably obscured by the residual C_6D_6 peak). HRMS (APCI): *m/z* calcd for $C_{54}H_{57}NO_6S+H^+$: 848.3985 [M+H]$^+$, found: 848.3981.

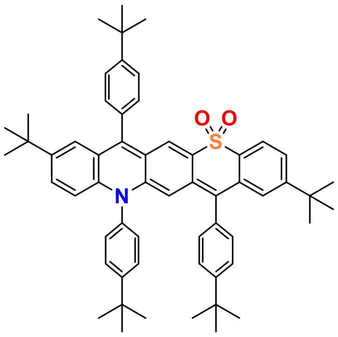

SO$_2$N-PA-b: Synthesized following the general procedure from 1-bromo-4-*tert*-butylbenzene (173 µL, 1.00 mmol) and diketone **7** (61 mg, 0.100 mmol). The product was purified by flash column chromatography (silica gel, hexanes/ethyl acetate 4/1), then recrystallized from methanol to afford **SO$_2$N-PA-b** (31 mg, 0.037 mmol, 37%) as a dark green powder.

The reaction was also performed on a larger scale, following the general procedure with the following quantities of reagents: diketone **7** (212 mg, 0.350 mmol), 1-bromo-4-*tert*-butylbenzene (600 µL, 3.50 mmol), *t*BuLi (1.7M in *n*-pentane, 2.1 mL, 3.6 mmol, 10eq), THF (10 mL). For the reduction step: $SnCl_2$ (1.33 g, 7.01 mmol), TFA (1.5 mL), toluene (30 mL). After extraction, drying with Na_2SO_4, filtration and evaporation of the solvents, the crude product was recrystallized from methanol and dried under vacuum at 100 °C for 4 h to remove residual *tert*-butylbenzene. **SO$_2$N-PA-b** (180 mg, 0.214 mmol, 61%) was obtained as a dark green powder.

^{1}H NMR (500 MHz, C_6D_6, 323 K) δ 8.23 (s, 1H), 8.12 (d, J = 8.2 Hz, 1H), 7.37 – 7.33 (m, 2H), 7.29 – 7.24 (m, 4H), 7.23 – 7.19 (m, 3H), 7.07 – 7.03 (m, 2H), 6.97 – 6.93 (m, 2H), 6.87 (dd, J = 8.2, 1.8 Hz, 1H), 6.83 (d, J = 1.8 Hz, 1H), 6.80 (dd, J = 8.8, 2.3 Hz, 1H), 6.13 (d, J = 8.8 Hz, 1H), 5.62 (s, 1H), 1.30 (s, 9H), 1.26 (s, 9H), 1.22 (s, 9H), 1.02 (s, 9H), 0.96 (s, 9H). ^{13}C NMR (126 MHz, Benzene-d_6, 323 K) δ 154.7, 152.1 (2 signals), 149.8, 144.0, 143.6, 142.0, 140.8, 138.3, 137.2, 137.1, 136.4, 132.3, 131.8, 131.2, 130.8, 130.4, 129.5, 129.2, 127.1, 126.5, 126.1, 125.4, 125.0, 124.7, 124.0, 123.7, 122.2, 120.6, 114.7, 99.9, 35.0, 34.8 (2 signals), 34.7, 34.1, 31.6 (2 signals), 31.3, 31.1, 30.9 (one aromatic signal is missing, probably obscured by the residual C_6D_6 peak). HRMS (APCI): *m/z* calcd for $C_{58}H_{65}NO_2S+H^+$: 840.4814 [M+H]$^+$, found: 840.4802.

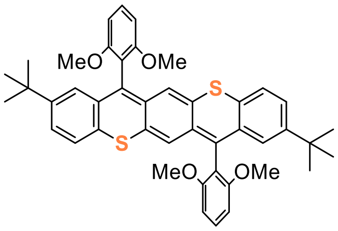

17a. The first step – addition of organolithium reagent to diketone **16** – was performed according to the general procedure on a 0.350 mmol scale, using the following quantities of reagents: 1-bromo-2,6-dimethoxybenzene (760 mg, 3.50 mmol), diketone **16**[8] (161 mg, 0.350 mmol), *t*BuLi (1.7M in *n*-pentane, 4.1 mL, 7.0 mmol), THF (11 mL).

The reduction step was carried out under milder conditions than in the general procedure. The reaction mixture from the first step was diluted with water (2 mL), followed by the addition of tin(II) chloride (1.33 g, 7.01 mmol, 20 eq) and 2M aqueous hydrochloric acid (2 mL). The resulting mixture was stirred at room temperature for 30 min. Water (20 mL) and 2M hydrochloric acid (5 mL) were then added, and the mixture was extracted three times with chloroform. The combined organic layers were washed with acidic brine (ca. 20 mL $2M_{aq}$ HCl per 30 mL of brine), followed by saturated aqueous sodium bicarbonate to neutralize residual acid. The organic phase was dried over anhydrous Na_2SO_4 and filtered. Solvents were removed under reduced pressure. The resulting crude product was recrystallized from toluene/n-heptane (ca. 1:20), affording compound **17a** (229 mg, 0.327 mmol, 93%) as a purple crystalline solid.

Due to significant aggregation, no signals could be detected in the ^1H and ^{13}C NMR spectra, even at elevated temperatures (up to 70 °C in C_6D_6). HRMS (APCI): *m/z* calcd for $C_{44}H_{44}S_2O_4$+H^+: 701.2759 $[M+H]^+$, found: 701.2751.

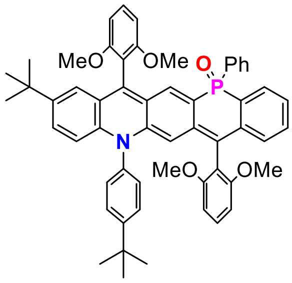

PON-PA. Synthesized following the general procedure from 1-bromo-2,6-dimethoxybenzene (217 mg, 1.00 mmol) and diketone **10** (61 mg, 0.100 mmol). The product was purified by column chromatography (dichloromethane/methanol 98/2) using *deactivated* silica gel (1 mL of water per 20 g of silica)* as the stationary phase. Recrystallization from toluene/n-hexane (0.5 mL / 5 mL) afforded **PON-PA** (55 mg, 0.065 mmol, 65%) as a green crystalline solid. ^1H NMR (500 MHz, C_6D_6, 323 K) δ 8.14 (dd, *J* = 11.4, 7.6 Hz,

* Silica gel deactivation: Water (1 g per 20 g of silica gel) was added to a suspension of silica gel in dichloromethane in a round-bottom flask. The flask was attached to a rotary evaporator and rotated until the suspension became uniform. Dichloromethane was then evaporated to dryness, yielding free-flowing deactivated silica gel, which was used imediately for chromatography.

2H), 8.07 (dd, J = 13.4, 7.4 Hz, 1H), 7.83 (d, J = 16.4 Hz, 1H), 7.20 (t, J = 8.4 Hz, 1H), 7.19 – 7.14 (m, 2H), 7.12 (d, J = 1.7 Hz, 1H), 7.09 – 6.98 (m, 4H), 6.99 – 6.89 (m, 2H), 6.78 – 6.72 (m, 2H), 6.61 (br s, 2H), 6.49 (d, J = 8.3 Hz, 1H), 6.42 (d, J = 8.3 Hz, 1H), 6.37 – 6.30 (m, 2H), 6.07 (d, J = 8.8 Hz, 1H), 4.88 (d, J = 4.6 Hz, 1H), 3.33 (s, 3H), 3.29 (s, 3H), 3.27 (s, 3H), 3.10 (s, 3H), 1.28 (s, 9H), 1.04 (s, 9H).
^{13}C NMR (126 MHz, C_6D_6, 323 K) δ 159.7, 159.4 (2 signals), 159.0, 151.2, 143.2, 141.8 (2 signals), 141.7 (2 signals), 140.2, 139.4, 137.4, 134.6, 134.5, 134.2, 132.9, 132.1, 131.7, 131.5, 131.4, 130.8, 130.6 (2 signals), 130.4 (2 signals), 129.7, 128.7, 127.6, 127.5, 126.7, 126.1, 126.0, 124.5, 124.4, 124.1, 123.8, 118.4, 117.8, 117.7, 114.0, 113.1, 105.2, 104.9, 104.8, 104.6, 99.9 (2 signals), 55.9, 55.8, 55.7, 55.4, 34.7, 34.0, 31.6, 31.3. ^{31}P{^{1}H} NMR (202 MHz, C_6D_6) δ 7.80. HRMS (APCI): *m/z* calcd for $C_{56}H_{54}NO_5P+H^+$: 852.3818 $[M+H]^+$, found: 852.3817. ^{31}P–^{13}C coupling complicates the ^{13}C NMR spectrum and hinders full signal assignment. Therefore, only a list of all observed peaks is provided.

General procedure for the synthesis of acene analogues with TIPS-acetylene side groups

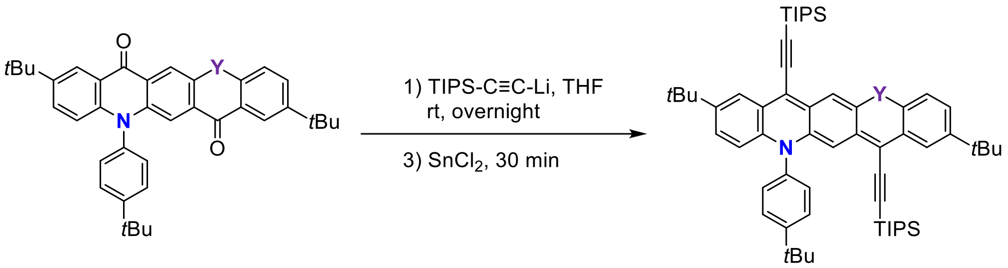

6, Y = S
7, Y = SO_2

SN-PA, Y = S
SO_2N-PA, Y = SO_2

Under an argon atmosphere, in a heat-dried Schlenk flask (heat gun/vacuum), (triisopropylsilyl)acetylene (0.34 mL, 1.52 mmol, 15 eq) was dissolved in tetrahydrofuran (2 mL), then the mixture was cooled to 0 °C and *n*-butyllithium (*n*BuLi, 1.52M in hexanes, 0.65 mL, 0.99 mmol, 10.0 eq) was added dropwise via syringe. The reaction mixture was stirred at 0 °C for 30 min, after which diketone **6** or **7** (0.100 mmol, 1.0 eq) was added under an argon flow, and the stirring was continued overnight at room temperature. The reaction was quenched with water (1 mL), followed by the addition of anhydrous tin(II) chloride (379 mg, 2.00 mmol, 20.0 eq). The resulting mixture was stirred at room temperature for 30 min. The reaction mixture was then diluted with water (10 mL) and 2M hydrochloric acid (2 mL), and extracted three times with chloroform. The combined organic layers were washed with acidic brine (ca. 20 mL $2M_{aq}$ HCl per 30 mL of brine), followed by saturated aqueous sodium bicarbonate. The organic phase was dried over anhydrous Na_2SO_4 and filtered. Celite (ca. 1 g) was added to the resulting mixture, and the solvents were evaporated to dryness under reduced pressure. The resulting residue was subjected to flash column chromatography (silica gel, conditions specified below).

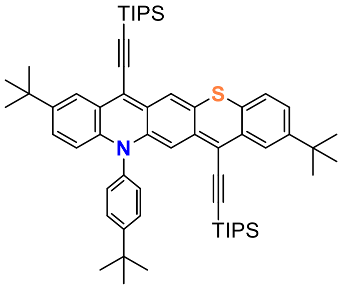

SN-PA-c. Synthesized following the general procedure using the following quantities of reagents: diketone **6** (172 mg, 0.300 mmol), (triisopropylsilyl)acetylene (1.0 mL, 4.5 mmol, 15 eq) *n*-butyllithium (1.52M in hexanes, 2.0 mL, 3.00 mmol, 10.0 eq), tin(II) chloride (1.14 g, 6.0 mmol, 20.0 eq). The product was purified by flash column chromatography (silica gel, hexanes/ethyl acetate 1/0 to 4/1), then recrystallized from chloroform/methanol to afford **SN-PA-c** (203 mg, 0.224 mmol, 75%) as a dark blue crystalline powder. ^{1}H NMR (500 MHz, C_6D_6, 323 K) δ 8.10 – 8.06 (m, 1H), 8.05 (d, *J* = 2.3 Hz, 1H), 7.62 (s, 1H), 7.42 – 7.35 (m, 2H), 6.98 – 6.92 (m, 2H), 6.80 – 6.73 (m, 2H), 6.62 (dd, *J* = 8.8, 2.3 Hz, 1H), 6.31 (s, 1H), 5.69 (d, *J* = 8.8 Hz, 1H), 1.33 – 1.20 (m, 48H), 1.19 – 1.14 (m, 18H), 1.08 – 1.01 (m, 3H). *Due to significant aggregation, no signals could be detected in the ^{13}C NMR spectra.* HRMS (APCI): *m/z* calcd for $C_{60}H_{81}NSSi_2+H^+$: 904.5707 $[M+H]^+$, found: 904.5703.

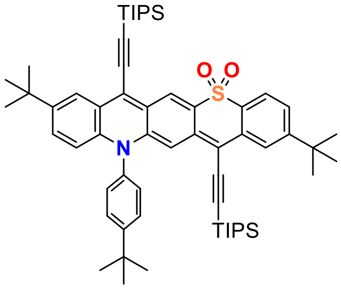

SO_2N-PA-c. Synthesized following the general procedure from diketone **7** (61 mg, 0.100 mmol). The product was purified by flash column chromatography (silica gel, hexanes/ethyl acetate 1/0 to 4/1), then recrystallized from methanol to afford **SO_2N-PA-c** (32 mg, 0.034 mmol, 34%) as a bright green powder. ^{1}H NMR (600 MHz, C_6D_6, 323 K) δ 9.03 (s, 1H), 8.25 (d, J = 1.8 Hz, 1H), 8.15 (d, J = 2.3 Hz, 1H), 8.13 (d, J = 8.4 Hz, 1H), 7.50 – 7.45 (m, 2H), 6.99 – 6.96 (m, 2H), 6.95 (dd, J = 8.2, 1.8 Hz, 1H), 6.79 (dd, J = 8.9, 2.3 Hz, 1H), 6.44 (s, 1H), 5.85 (d, J = 8.8 Hz, 1H), 1.36 (s, 9H), 1.34 (d, J = 7.0 Hz, 18H), 1.30 – 1.26 (m, 3H), 1.25 (s, 9H), 1.21 (s, 9H), 1.17 (d, J = 7.2 Hz, 18H), 1.10 – 1.04 (m, 3H). ^{13}C NMR (126 MHz, C_6D_6 + TFA) δ 158.3, 156.6, 153.5, 143.7, 143.0, 142.4, 141.8, 140.2, 136.2, 134.0, 133.1, 130.3, 129.9, 127.5, 126.9, 126.7, 126.1, 125.4, 125.0, 124.3, 124.0, 120.1, 117.0, 99.6, 96.5, 35.5 (2 signals), 35.4, 31.2, 30.7, 30.3, 18.8, 18.5, 11.6 (2 signals) (3 aromatic signals are missing, probably obscured by the residual C_6D_6 peak). HRMS (APCI): *m/z calcd* for $C_{60}H_{81}NSO_2Si_2+H^+$: 936.5605 $[M+H]^+$, found: 936.5615.

General procedure for the oxidation of dithia-acene analogues to disulfones

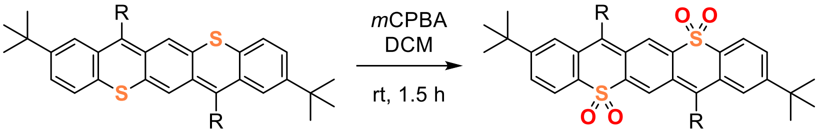

17
a, R = 2,6-(OMe)$_2$Ph
c, R = -C≡C-TIPS

diSO$_2$-PA
a, R = 2,6-(OMe)$_2$Ph, 86%
c, R = -C≡C-TIPS, 88%

In a round-bottom flask, compound **17a** or **17c**[8] was dissolved in dichloromethane, followed by the addition of *meta*-chloroperoxybenzoic acid (*m*CPBA, ≥77%$_{wt}$, 4.4 eq). The flask was sealed with a septum fitted with an argon-filled balloon, and the reaction mixture was stirred at room temperature for 1.5 h. During this time, the solution gradually changed color and began to exhibit bright fluorescence under ambient light. The solvent was then evaporated to dryness under reduced pressure, and the crude product was recrystallized from chloroform/methanol.

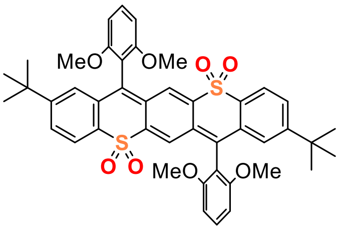

diSO$_2$-PA-a. Synthesized following the general procedure using the following quantities of reagents: **17a** (70 mg, 0.100 mmol), *m*CPBA (≥77%$_{wt}$, 99 mg, 0.44 mmol), and DCM (10 mL, not anhydrous). **diSO$_2$-PA-a** (66 mg, 0.086 mmol, 86%) was obtained as an orange powder. ^{1}H NMR (500 MHz, CD_2Cl_2) δ 7.96 (d, *J* = 8.2 Hz, 2H), 7.58 – 7.52 (m, 4H), 7.46 (s, 2H), 7.07 (d, *J* = 1.8 Hz, 2H), 6.81 (d, *J* = 8.5 Hz, 4H), 3.72 (s, 12H), 1.16 (s, 18H). ^{13}C NMR (126 MHz, CD_2Cl_2) δ 158.9, 156.9, 135.2, 133.7, 133.3, 133.2, 132.0, 130.2, 127.5, 127.3, 127.2, 123.8, 111.8, 104.6, 56.4, 35.4, 30.9. HRMS (APCI): *m/z* calcd for $C_{44}H_{44}O_8S_2+H^+$: 765.2556 $[M+H]^+$, found: 765.2550.

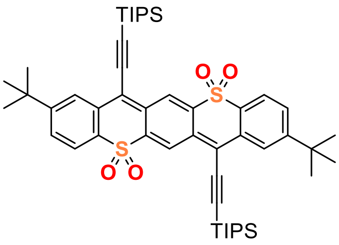

diSO$_2$-PA-c. Synthesized following the general procedure using the following quantities of reagents: **17c**[8] (60 mg, 0.076 mmol), *m*CPBA (≥77%$_{wt}$, 75 mg, 0.33 mmol), and DCM (10 mL, not anhydrous). **diSO$_2$-PA-a** (57 mg, 0.067 mmol, 88%) was obtained as a dark crystalline solid.

^{1}H NMR (500 MHz, CD_2Cl_2) δ 8.59 (s, 2H), 8.32 (d, *J* = 1.8 Hz, 2H), 8.03 (d, *J* = 8.2 Hz, 2H), 7.69 (dd, *J* = 8.2, 1.8 Hz, 2H), 1.39 (s, 18H), 1.36 – 1.28 (m, 6H), 1.26 (d, *J* = 6.4 Hz, 36H). ^{13}C NMR (126 MHz, CD_2Cl_2)

δ 157.6, 136.6, 133.4, 132.0, 131.6, 129.6, 128.8, 128.2, 124.0, 120.5, 114.4, 102.8, 35.9, 31.1, 19.0, 11.8. HRMS (APCI): *m/z* calcd for $C_{50}H_{68}O_4S_2Si_2+H^+$: 853.4176 $[M+H]^+$, found: 853.4171.

2-((2-(1,3-Dioxolan-2-yl)phenyl)amino)anthracene-9,10-dione (S9)

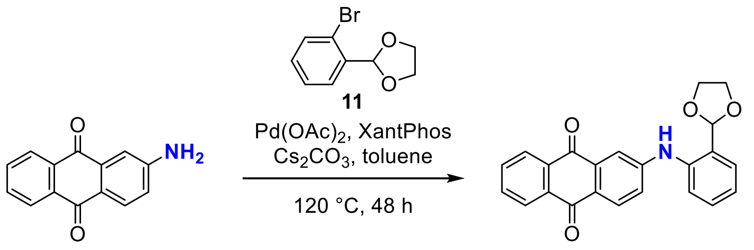

12 **S9**, 69%

2-Aminoanthraquinone (3.00 g, 13.4 mmol, 1.0 eq.), palladium(II) acetate (75 mg, 0.34 mmol, 2.5 mol%), XantPhos (292 mg, 0.504 mmol, 3.8 mol%), and cesium carbonate (8.76 g, 26.9 mmol, 2.0 eq) were placed in a Schlenk flask. The flask was evacuated and backfilled with argon three times, followed by the addition of anhydrous toluene (150 mL) and 2-(2-bromophenyl)-1,3-dioxolane (**11**, 3.69 g, 16.1 mmol, 1.2 eq). The mixture was again evacuated and backfilled with argon three times, then sealed, and the reaction mixture was stirred at 120 °C for 48 h. After cooling, the reaction mixture was passed through a Celite pad to remove inorganic salts. Celite (ca. 10 g) was added to the filtrate, and the solvents were evaporated to dryness. The resulting residue was subjected to flash column chromatography (hexanes / ethyl acetate 19/1 to 1/1; the product eluted at ~7/3), affording compound **S9** (3.45 g, 9.29 mmol, 69%) as a viscous orange oil. ^{1}H NMR (500 MHz, $CDCl_3$) δ 8.30 (dd, J = 7.6, 1.5 Hz, 1H), 8.28 – 8.23 (m, 1H), 8.19 (d, J = 8.6 Hz, 1H), 7.81 – 7.74 (m, 2H), 7.74 (td, J = 7.4, 1.6 Hz, 1H), 7.57 (dd, J = 7.7, 1.6 Hz, 1H), 7.50 (d, J = 8.0 Hz, 1H), 7.39 (td, J = 7.7, 1.6 Hz, 1H), 7.35 – 7.29 (m, 2H), 7.15 (td, J = 7.5, 1.1 Hz, 1H), 5.92 (s, 1H), 4.18 – 4.04 (m, 4H). ^{13}C NMR (126 MHz, $CDCl_3$) δ 183.8, 181.7, 149.3, 138.8, 135.6, 134.3, 134.2, 133.8, 133.5, 130.1, 130.0, 129.0, 127.6, 127.2, 127.1, 125.6, 123.8, 121.2, 119.7, 112.6, 102.3, 65.3. HRMS (APCI): *m/z* calcd for $C_{23}H_{17}NO_4+H^+$: 372.1236 $[M+H]^+$, found: 372.1237.

2-((2-(1,3-Dioxolan-2-yl)phenyl)(butyl)amino)anthracene-9,10-dione (S10)

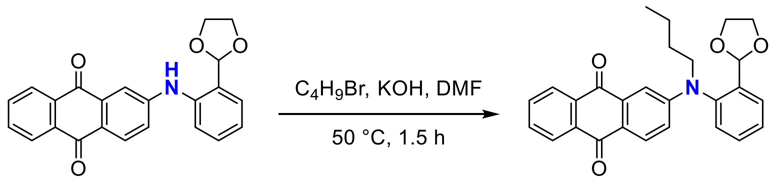

S9 **S10**, quant.

Compound **S9** (3.45 g, 9.29 mmol) was dissolved in dimethyformamide (100 mL) and heated to 50 °C. Potassium hydroxide (2.09 g, 37.2 mmol, 4.0 eq) was added and the resulting mixture was stirred at 50 °C for 10 min, during which nearly all solids dissolved. *n*-Butyl bromide (1.50 mL, 14.0 mmol, 1.5 eq) was then

added and the mixture was stirred at the same temperature for 1.5 h. After cooling to room temperature, the reaction mixture was diluted with water and extracted three times with dichloromethane. The combined organic layers were washed with brine, dried over anhydrous Na_2SO_4 and filtered. The solvents were removed under reduced pressure, and the residue was dried under vacuum at 100 °C for 4 h, affording compound **S10** (3.98 g, quantitative yield) as a red viscous oil. ^1H NMR (500 MHz, $CDCl_3$) δ 8.27 (d, *J* = 7.4 Hz, 1H), 8.23 (d, *J* = 7.3 Hz, 1H), 8.05 (d, *J* = 8.8 Hz, 1H), 7.80 – 7.67 (m, 3H), 7.54 – 7.42 (m, 3H), 7.16 (d, *J* = 7.5 Hz, 1H), 6.65 (dd, *J* = 8.9, 2.7 Hz, 1H), 5.69 (s, 1H), 4.14 – 4.04 (m, 2H), 3.96 – 3.88 (m, 2H), 3.87 – 3.44 (m, 2H), 1.75 (br s, 2H), 1.42 (h, *J* = 7.4 Hz, 2H), 0.98 (t, *J* = 7.4 Hz, 3H). ^{13}C NMR (126 MHz, $CDCl_3$) δ 184.3, 181.7, 153.3, 143.7, 136.6, 135.2, 134.5, 134.1, 133.9, 133.2, 131.4, 129.9, 129.6, 128.8, 128.6, 127.1, 127.0, 123.1, 118.0, 109.0, 100.1, 65.6, 52.8, 29.1, 20.4, 14.0. HRMS (APCI): *m/z* calcd for $C_{27}H_{25}NO_4+H^+$: 428.1862 $[M+H]^+$, found: 428.1864.

2-(Butyl(9,10-dioxo-9,10-dihydroanthracen-2-yl)amino)benzaldehyde (13)

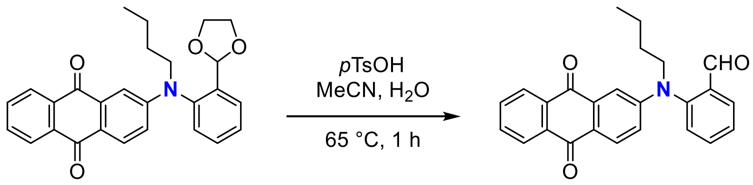

S10 **13**, quant.

To a stirred solution of compound **S10** (3.98 g, 9.31 mmol) in a mixture of acetonitrile (100 mL) and water (20 mL) was added a solution of *para*-toluenosulfonic acid (1.0 M in water, 1.9 mL, 1.86 mmol, 20 mol%). The reaction mixture was stirred at 65 °C for 1 h. The reaction was quenched with saturated aqueous sodium bicarbonate solution (10 mL) and extracted three times with dichloromethane. The combined organic layers were washed with brine, dried over anhydrous Na_2SO_4 and filtered. The solvents were removed under reduced pressure, and the resulting residue was dried under vacuum to afford compound **13** (3.57 g, quantitative yield) as a dark red viscous oil. ^1H NMR (500 MHz, $CDCl_3$) δ 10.04 (s, 1H), 8.27 (dd, J = 7.4, 1.5 Hz, 1H), 8.23 (dd, J = 7.5, 1.6 Hz, 1H), 8.10 (d, J = 8.8 Hz, 1H), 8.06 (dd, J = 7.7, 1.7 Hz, 1H), 7.83 – 7.68 (m, 3H), 7.56 (t, J = 7.6 Hz, 1H), 7.45 (d, J = 2.7 Hz, 1H), 7.32 (d, J = 7.9 Hz, 1H), 6.80 (dd, J = 8.8, 2.8 Hz, 1H), 3.95 – 3.76 (m, 2H), 1.75 (p, J = 7.6 Hz, 2H), 1.42 (h, J = 7.4 Hz, 2H), 0.97 (t, J = 7.4 Hz, 3H). ^{13}C NMR (126 MHz, $CDCl_3$) δ 190.2, 183.9, 181.7, 153.5, 147.7, 136.5, 135.4, 134.3, 134.2, 133.8, 133.7, 133.4, 130.6, 130.3, 129.9, 128.5, 127.1 (2 signals), 124.0, 118.0, 109.8, 53.8, 29.4, 20.3, 13.9. HRMS (APCI): *m/z* calcd for $C_{25}H_{21}NO_3+H^+$: 384.1600 $[M+H]^+$, found: 384.1601.

Synthesis of CON-PA

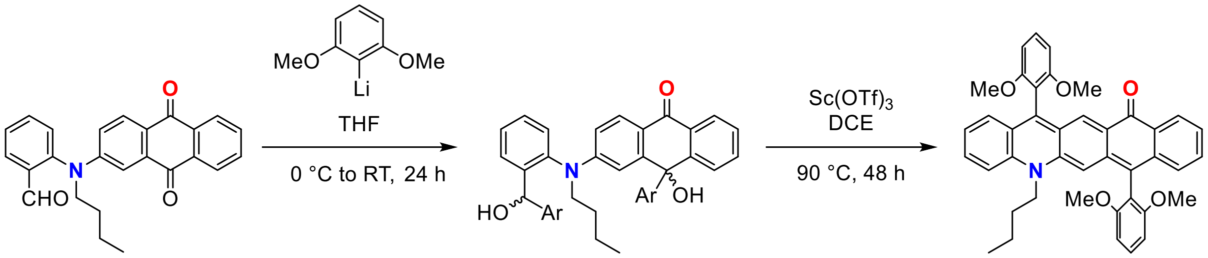

13 **14**, 39% **CON-PA**, 12%

Step 1. Arylation of compound 13. Under an argon atmosphere, 2-bromo-1,3-dimethoxybenzene (1.56 g, 7.19 mmol, 2.2 eq) was dissolved in tetrahydrofuran (50 mL) and cooled to −78 °C. *tert*-Butyllithium (1.7 M in *n*-pentane, 8.1 mL, 13.8 mmol, 4.2 eq) was added dropwise. The reaction mixture was stirred at −78 °C for 15 min, then allowed to gradually warm to ~0 °C. It was then submerged in an ice bath and stirred for an additional 30 min at 0 °C. The resulting aryllithium solution was transferred *via* cannula to a second Schlenk flask containing a solution of compound **13** (1.25 g, 3.26 mmol, 1.0 eq) in tetrahydrofuran (50 mL), pre-cooled and stirred at 0 °C. The ice bath was removed and the reaction mixture was stirred at room temperature for 20 h. In a separate Schlenk flask, an additional amount of the aryllithium reagent (1.0 eq) was prepared using the same procedure from 2-bromo-1,3-dimethoxybenzene (694 mg, 3.27 mmol, 1.0 eq), *t*-BuLi (1.7M in *n*-pentane, 3.9 mL, 2.0 eq) in THF (20 mL). This second batch was added to the main reaction mixture in two equal portions (~0.5 eq each), at 1 h intervals. The resulting mixture was stirred at room temperature for a further 1 h. The reaction was quenched by the addition of 10% aqueous citric acid (10 mL) and water, followed by extraction with dichloromethane (3×). The combined organic layers were washed with brine, dried over anhydrous Na_2SO_4 and filtered. The solvents were removed under reduced pressure. The products were separated by column chromatography (silica gel, toluene / ethyl acetate 4 /1). Fractions containing compound **14** were combined and concentrated to afford the product as a yellowish foam-like powder with bright blue fluorescence (843 mg, ~1.28 mmol, 39%, mixture of two diastereoisomers). The product was used in the next step without further purification. HRMS (APCI): *m/z* calcd for $C_{41}H_{41}NO_7+H^+$: 660.2961 $[M+H]^+$, found: 660.2963.

Step 2. Cyclization and dehydration. In a heat-dried Schlenk flask, intermediate **14** (413 mg, 0.626 mmol) was dissolved in anhydrous dichloroethane (DCE, 60 mL) under an argon atmosphere, and scandium trifluoromethanesulfonate (92 mg, 0.19 mmol, 30 mol%) was added. The flask was sealed and the reaction mixture was stirred at 90 °C for 48 h. The reaction was quenched by the addition of *N*,*N*-diisopropylethylamine (DIPEA, 0.1 mL), which caused an immediate color change from dark red to green. The mixture was passed through a Celite pad to remove inorganic residues. The filtrate was washed with brine, dried over anhydrous Na_2SO_4 and filtered. The solvents were removed under reduced pressure. The product was separated by column chromatography (*deactivated*[*] sillica gel, toluene / ethyl acetate 19/1).

[*] See the synthesis of **PON-PA** and the corresponding footnote for the deactivation procedure.

The product was further purified by trituration with a toluene/*n*-hexane mixture as follows:

The crude product was suspended in *n*-hexane (3 ml), then toluene (1 mL) was added. The mixture was heated to boiling, allowed to cool to room temperature, then diluted with *n*-hexane (30 mL) and reheated. After cooling to room temperature, the suspension was kept in a refrigerator overnight. The precipitated solid was filtered and washed with cold *n*-hexane. Compound **CON-PA** (48 mg, 77 µmol, 12%) was obtained as a green powder.

Important note: *Toluene must be added to the suspension in n-hexane, not to the dry solid, as it would cause complete dissolution, preventing reprecipitation*.

Warning: CON-PA *is susceptible to hot alcohols.*

^{1}H NMR (500 MHz, DMSO-d_6) δ 8.07 (dd, *J* = 7.9, 1.5 Hz, 1H), 7.73 (s, 1H), 7.60 (t, *J* = 8.4 Hz, 1H), 7.51 – 7.43 (m, 3H), 7.28 (d, *J* = 8.6 Hz, 1H), 7.17 (td, *J* = 7.4, 1.1 Hz, 1H), 6.95 (d, *J* = 8.5 Hz, 2H), 6.94 – 6.86 (m, 3H), 6.86 – 6.80 (m, 2H), 5.67 (s, 1H), 3.65 (s, 6H), 3.64 (s, 6H), 1.49 (br s, 2H), 1.23 – 1.14 (br m, 2H), 0.83 (t, *J* = 7.3 Hz, 3H). ^{13}C NMR (126 MHz, DMSO-d_6) δ 182.5, 158.5, 157.7, 141.3, 141.2, 139.7, 136.8, 133.2, 133.0, 132.4, 131.9, 131.2, 129.5, 129.2, 127.2, 126.6, 126.2, 126.2, 124.7, 123.0, 122.8, 121.1, 114.0, 113.2, 112.6, 110.9, 104.5, 97.5, 55.9, 55.7, 46.0, 26.5, 19.3, 13.5. HRMS (APCI): *m/z* calcd for $C_{41}H_{37}NO_5+H^+$: 624.2750 $[M+H]^+$, found: 624.2747.

2. X-ray data

Single crystals suitable for X-ray diffraction were obtained by slow vapor diffusion of a poor solvent into a solution of the compound in a good solvent (solvent combinations are specified in Table S1 below). CCDC 2451461, 2451462, 2492451, 2492452, and 2492475 contain the supplementary crystallographic data for this paper. These data can be obtained free of charge from The Cambridge Crystallographic Data Centre via www.ccdc.cam.ac.uk/structures.

Table S1. Summary of X-ray crystallographic data for the studied compounds.

Compound	SO_2N-PA-a [a]	SO_2N-PA-a [a]	CON-PA	diSO_2-PA-a	diSO_2-PA-c
CCDC Number	2492475	2492451	2492452	2451462	2451461
Recryst. solvents (good / poor)	toluene / cHex	benzene / cHex	DCE / cHex	toluene[b] / cHex	toluene / cHex
Formula	$C_{54}H_{57}NO_6S{\cdot}0.76C_7H_8$	$C_{54}H_{57}NO_6S$	$C_{42}H_{37}ClNO_5$	$C_{22}H_{22}O_4S$	$C_{25}H_{34}O_2SSi$, C_3H_6
$D_{calc.}$/ g cm^{-3}	1.184	1.081	1.295	1.319	1.172
μ/mm^{-1}	0.959	0.910	1.363	1.699	1.670
Formula Weight	918.216	848.06	671.17	382.48	468.78
Color	dark blue	green	green	dark red	dark red
Shape	plate	irregular	irregular	block	block
Size/mm^3	0.30×0.20×0.05	0.30×0.25×0.14	0.11×0.10×0.05	0.35×0.3×0.1	0.35×0.3×0.3
T/K	100.01(10)	150.00(10)	146(6)	100.00(10)	100.01(10)
Crystal System	monoclinic	monoclinic	orthorhombic	monoclinic	monoclinic
Space Group	$P2_1$	$P2_1$	$Fdd2$	$P2_1/c$	$P2_1/c$
a/Å	14.4204(3)	14.4996(3)	41.297(3)	10.32317(9)	12.91166(11)
b/Å	12.06864(16)	12.0635(2)	25.8938(13)	16.41393(11)	18.38492(11)
c/Å	16.3610(4)	16.4265(4)	12.8778(6)	12.07869(11)	11.93501(11)
α/°	90	90	90	90	90
β/°	115.245(3)	114.931(3)	90	109.7947(10)	110.2828(10)
γ/°	90	90	90	90	90
V/Å^3	2575.45(11)	2605.51(11)	13770.8(13)	1925.73(3)	2657.46(4)
Z	2	2	16	4	4
Z'	1	1	1	1	1
Wavelength/Å	1.54184	1.54184	1.54184	1.54184	1.54184
Radiation type	Cu K$_\alpha$	Cu K$_\alpha$	Cu K$_\alpha$	Cu K$_\alpha$	Cu K$_\alpha$
Θ_{min}/°	2.99	2.967	3.980	4.55	3.65
Θ_{max}/°	70.20	74.477	75.337	70.05	70.04
Measured Refl.	38720	22301	16006	35717	49403
Independent Refl.	8498	8300	4991	3653	5046
Reflections with I > 2(I)	7858	7386	3110	3636	4977
R_{int}	0.0451	0.0269	0.0540	0.0266	0.0284
Parameters	677	572	449	249	298
Restraints	14	1	1	0	0
Largest Peak	1.3339	1.175	0.322	0.3791	0.4398
Deepest Hole	-0.3903	-0.452	-0.483	-0.4911	-0.4263
GooF	1.0489	1.047	1.066	1.0527	1.0351
wR_2 (all data)	0.2401	0.2360	0.2933	0.0866	0.0869
wR_2	0.2319	0.2255	0.2569	0.0865	0.0866
R_1 (all data)	0.0914	0.0853	0.1192	0.0336	0.0334
R_1	0.0867	0.0794	0.0871	0.0335	0.0331
Bond precision C–C /Å	**0.0071**	**0.0091**	**0.0149**	**0.0019**	**0.0021**

[a] Two different crystal structures were measured for **SO_2N-PA-a**.

[b] a few drops of chloroform were added to toluene to facilitate dissolution of the compound.

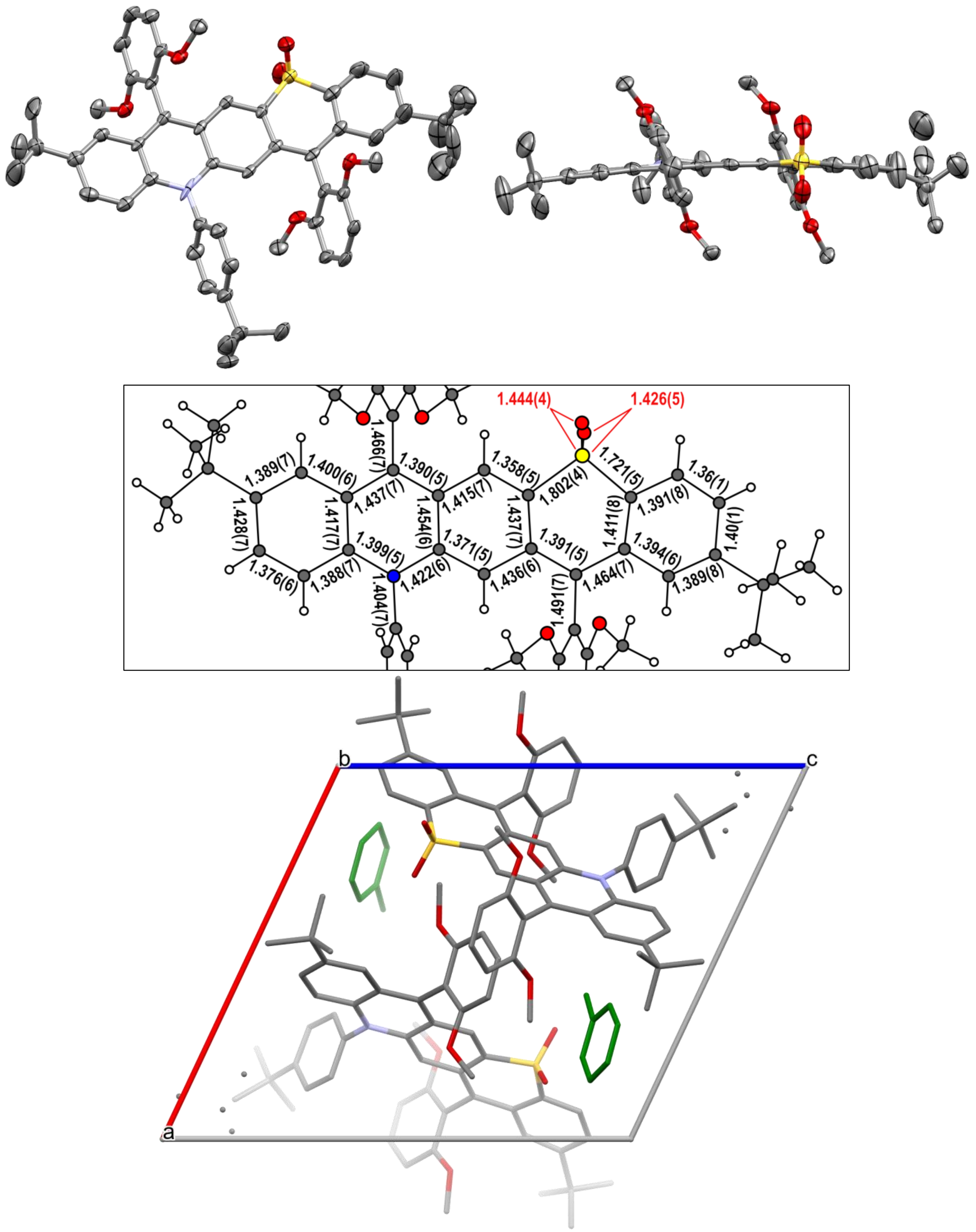

Figure S1. X-ray structure of **SO_2N-PA-a** (CCDC 2492475). (Top) Two projections of the molecule with thermal ellipsoids shown at the 50% probability level. (Middle) Bond lengths (Å) within the π-system. (Bottom) View of the unit cell along the *c* crystallographic axis. Hydrogen atoms are omitted for clarity. Solvent molecules are depicted in green.

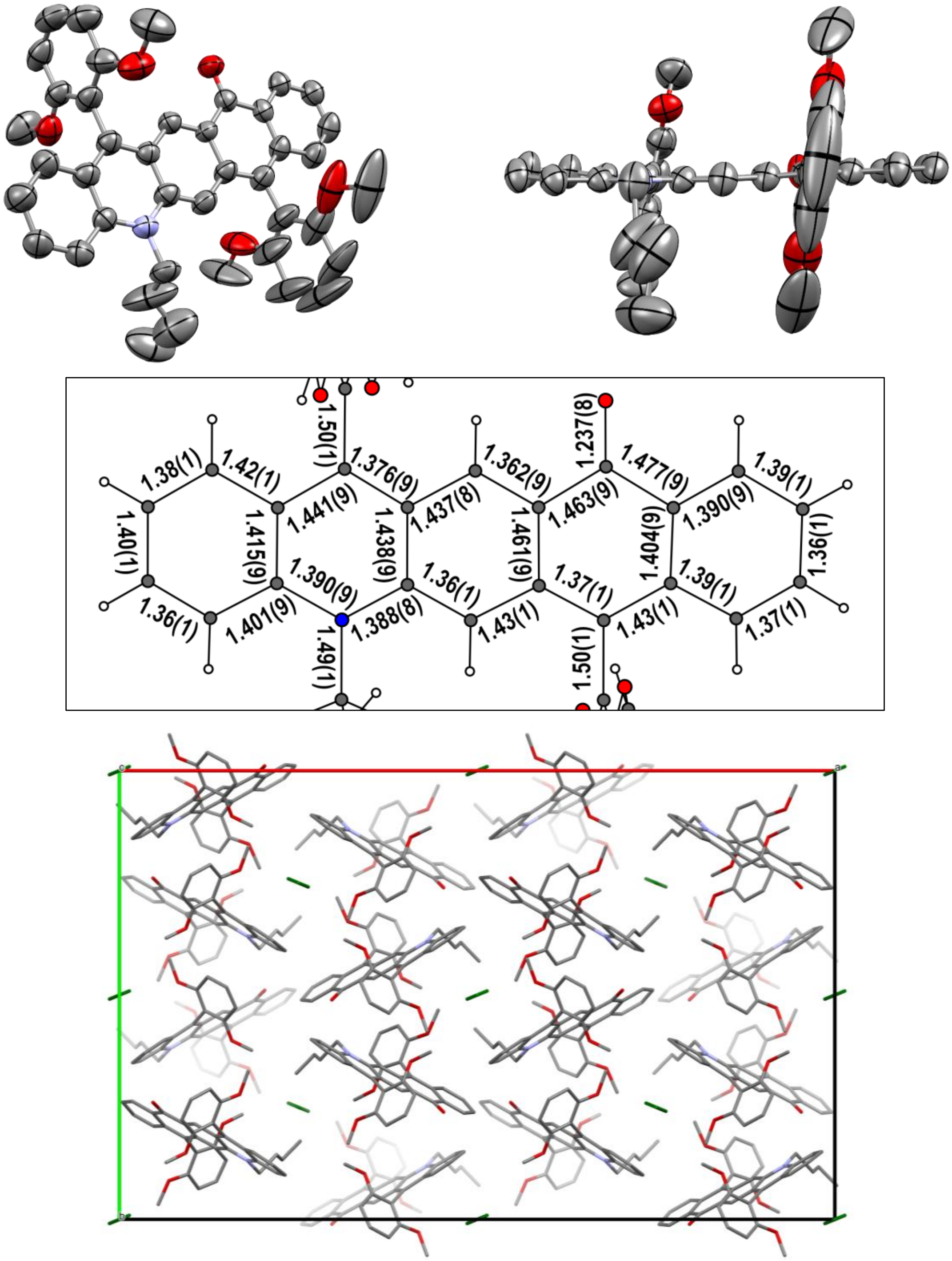

Figure S2. X-ray crystal structure of **CON-PA** (CCDC 2492452). (Top) Two projections of the molecule with thermal ellipsoids shown at the 50% probability level. (Middle) Bond lengths (Å) within the π-system. (Bottom) View of the unit cell along the *c* crystallographic axis. Hydrogen atoms are omitted for clarity. Solvent molecules are depicted in green.

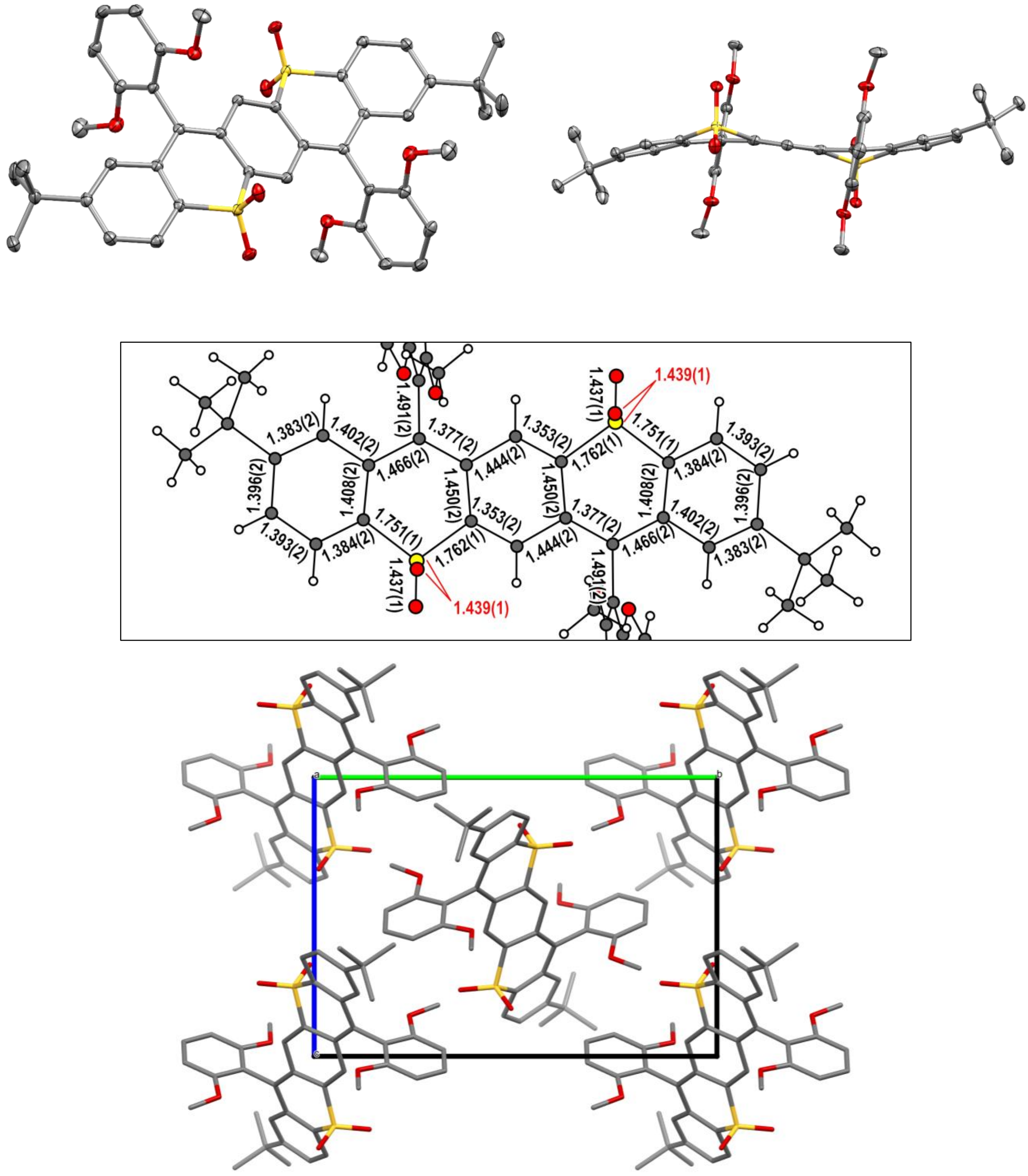

Figure S3. X-ray structure of **diSO$_2$-PA-a** (CCDC 2451462). (Top) Two projections of the molecule with thermal ellipsoids shown at the 50% probability level. (Middle) Bond lengths (Å) within the π-system. (Bottom) View of the unit cell along the *a* crystallographic axis. Hydrogen atoms are omitted for clarity.

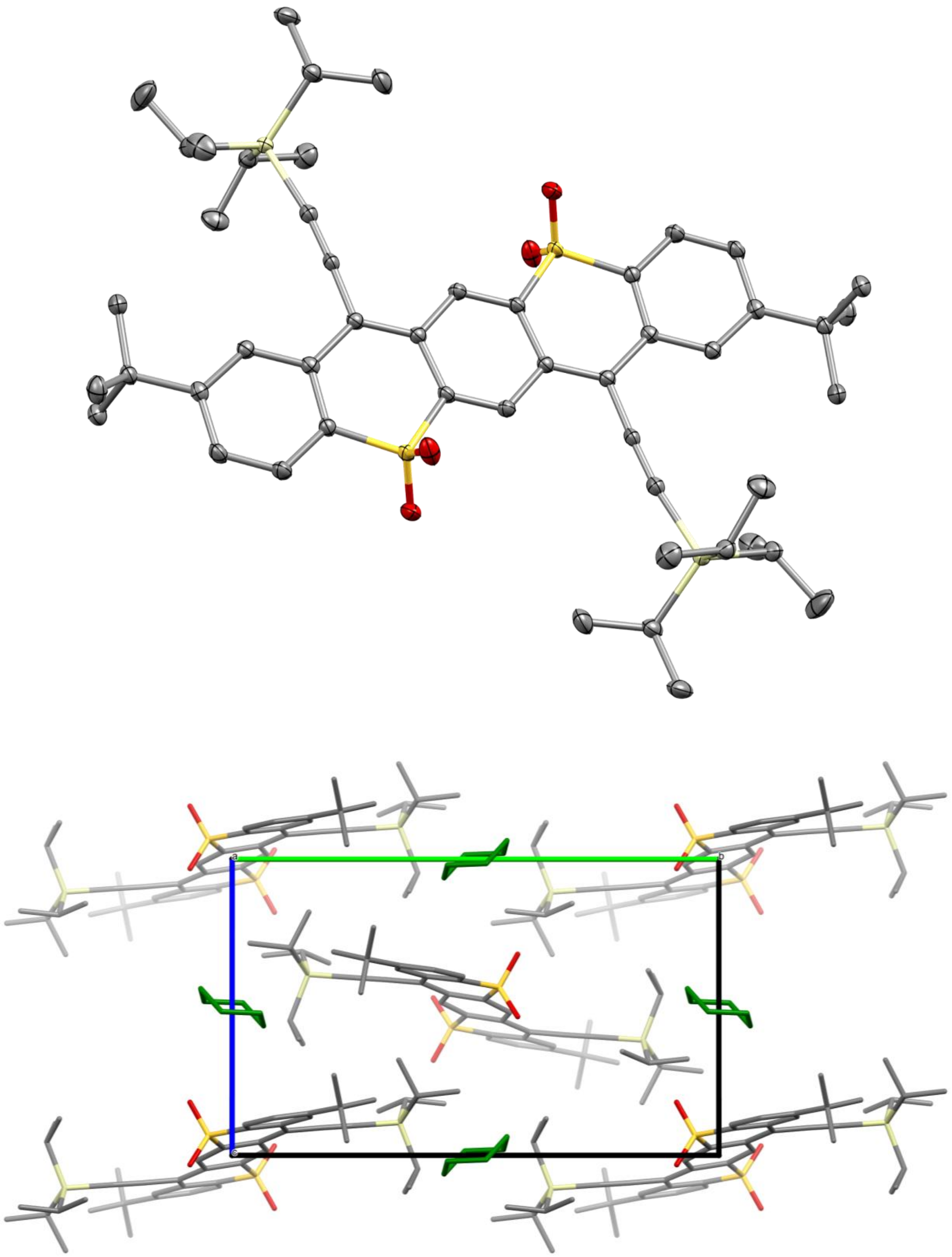

Figure S4. X-ray structure of **diSO$_2$-PA-c** (CCDC 2451461). (Top) Two projections of the molecule with thermal ellipsoids shown at the 50% probability level. (Bottom) View of the unit cell along the *a* crystallographic axis. Hydrogen atoms are omitted for clarity. Solvent molecules are depicted in green.

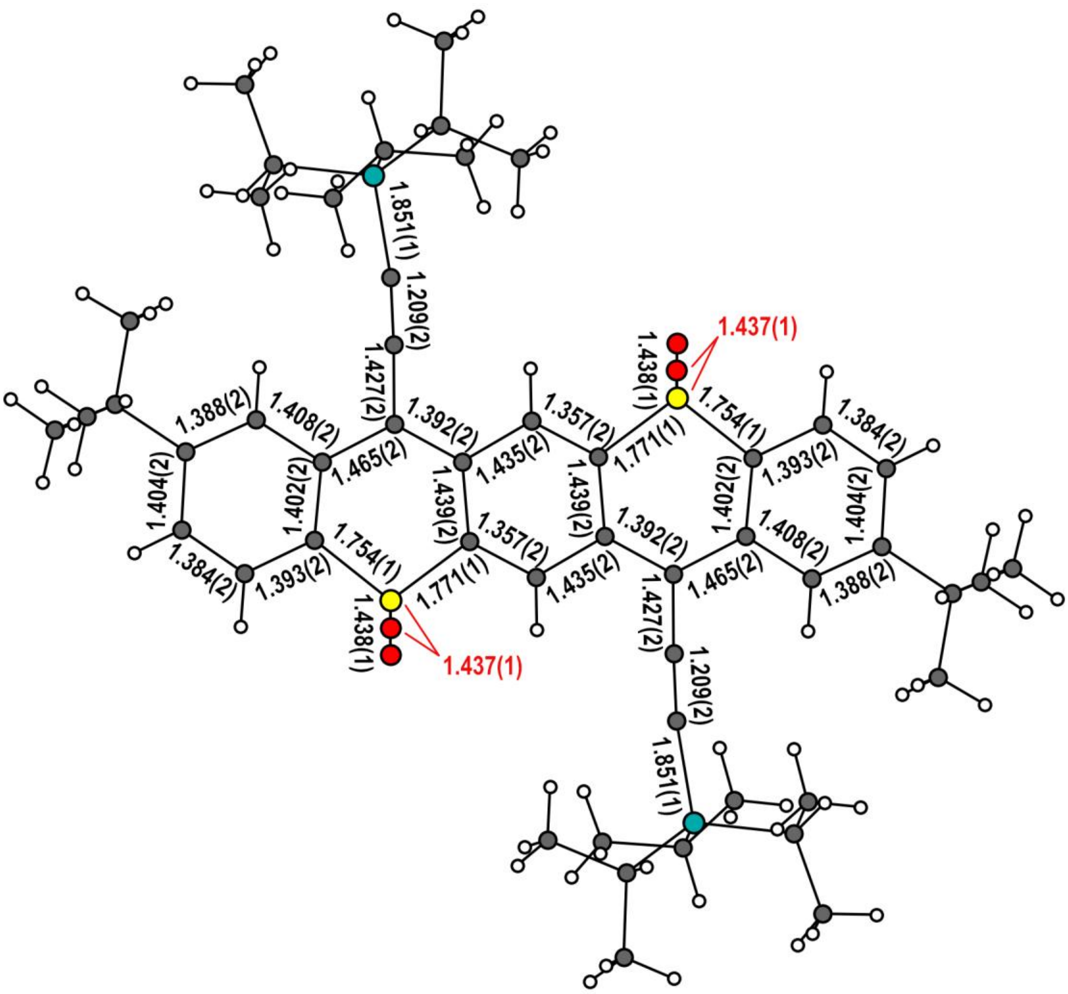

Figure S5. Bond lengths (Å) in the π-system of **diSO$_2$-PA-c** from the crystal structure (CCDC 2451461).

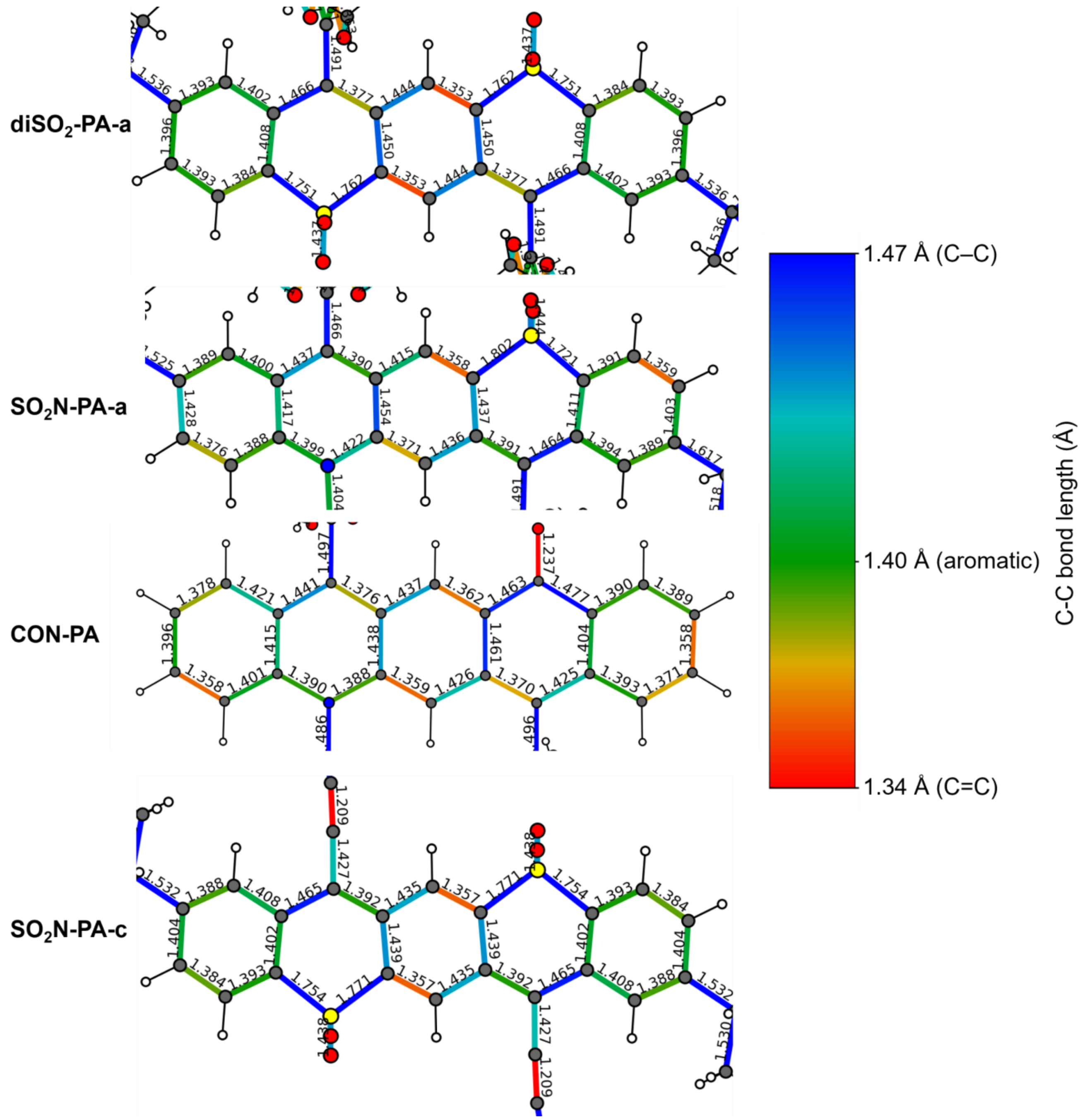

Figure S6. Bond-length analysis of the crystal geometries of pentacene analogues bearing different substituents. Bonds were color-coded to highlight discrete bond-length patterns; the corresponding color scale is shown on the right. Images were generated using the *xyz2BL.py* script.

3. Photophysical properties

Calculation of the positions of 0-0 vibronic bands in absorption spectra of DA-pentacenes

The positions of the 0-0 bands were estimated by deconvoluting the absorption spectra through non-linear fitting of four Gaussian curves to the lowest-energy absorption band, represented on an energy-proportional wavenumber scale. The following equation was used as the fitting model, assuming a uniform distribution of vibronic transitions with interval d, corresponding to the vibronic progression:

$$\varepsilon(\tilde{\nu}) = a_1 e^{-b_1(\tilde{\nu}-\tilde{\nu}_{00})^2} + a_2 e^{-b_2(\tilde{\nu}-\tilde{\nu}_{00}-d)^2} + a_3 e^{-b_3(\tilde{\nu}-\tilde{\nu}_{00}-2d)^2} + a_4 e^{-b_4(\tilde{\nu}-\tilde{\nu}_{00}-3d)^2} + C$$

Where: $\varepsilon(\tilde{\nu})$ - molar absorptivity [$M^{-1}\cdot cm^{-1}$] as a function of wavenumber $\tilde{\nu}$

a_i - intensities of the individual Gaussian bands

b_i - broadening factors

$\tilde{\nu}_{00}$ - wavenumber of the 0-0 transition

d - vibronic progression interval

C - constant accounting for baseline elevation (typically close to 0 $M^{-1}\cdot cm^{-1}$)

For simplicity, the fitting ranges of the experimental absorption spectra were selected to start at the flat, baseline-adjacent red end of the spectrum and to end approximately at the center of the 0-4 transition lobe. Extending the fitting range to include higher-energy features required more Gaussians but had minimal impact on the determined positions of the 0-0 bands and did not improve the quality of the fit.

Figure S7 presents a representative example of a spectrum deconvoluted using this method, and Table S2 summarizes the extracted 0-0 transition energies for the DA-pentacenes in various solvents.

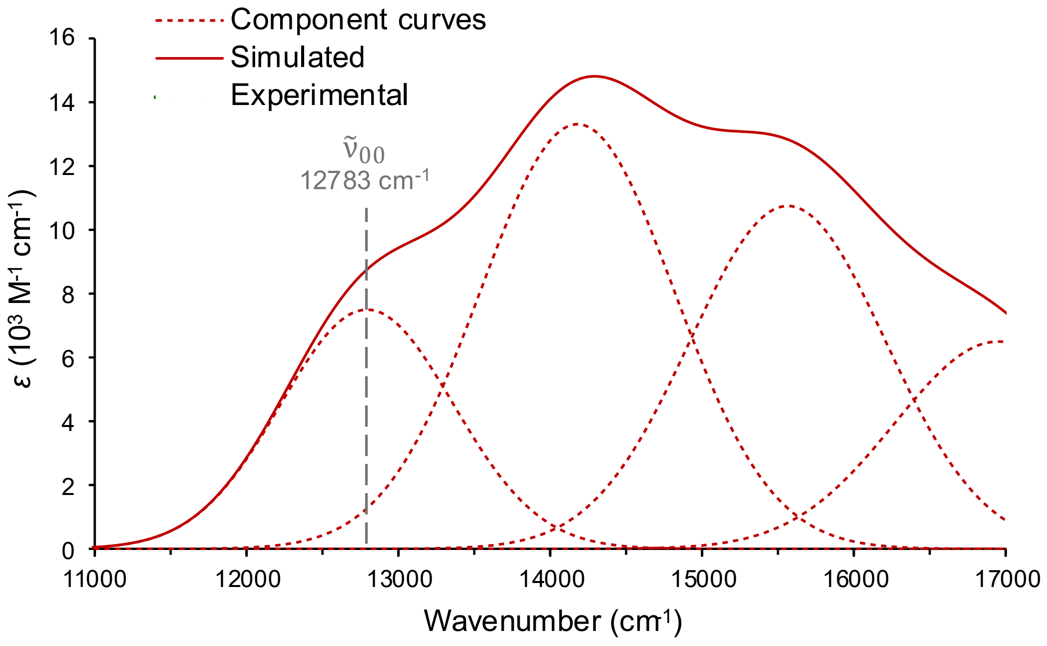

Figure S7. Example of a deconvoluted absorption spectrum of **PON-PA** in *n*-hexane, showing the experimental spectrum (green dotted line), the simulated (fitted) spectrum (ren solid line), which is the sum of four component Gaussian curves (red dshed lines). The calculated position of the 0-0 band is indicated in grey.

Table S2. Positions of the 0-0 bands $\tilde{\nu}_{00}$, vibronic progressions d and correlations coefficients *r* determined using non-linear fitting of the absorption spectra of DA-pentacene derivatives in different solvents.

Compound	Solvent	$\tilde{\nu}_{00}$ [cm^{-1}]	λ_{00} [nm] [a]	d [cm^{-1}]	r
**PON-PA**	*n*-hexane	12783.38	782.3	1387.59	0.999979
	CH_2Cl_2	12149.12	823.1	1449.34	0.999996
	methanol	11885.69	841.3	1322.93	0.999992
**SO$_2$N-PA-a**	*n*-hexane	12382.99	807.6	1430.06	0.999979
	CH_2Cl_2	11598.54	862.2	1266.95	0.999988
	methanol	11501.66	869.4	1248.94	0.999992
**CON-PA**	*n*-hexane	9858.90	1014.3	1504.98	0.999925
	CH_2Cl_2	9240.23	1082.2	1447.77	0.999986
	methanol	8629.74	1158.8	1375.44	0.999876
**SO$_2$N-PA-c**	*n*-hexane	10981.03	910.7	1394.17	0.999978
	CH_2Cl_2	10257.55	974.9	1305.35	0.999963
	methanol	10226.48	977.9	1280.04	0.999965

[a] Calculated using the formula: $\lambda_{00} = 10^7 / \tilde{\nu}_{00}$.

All

Table S3. Photophysical properties of compounds in different solvents.

Compound	Solvent	λ_{abs} [nm]	ε [$M^{-1}\cdot cm^{-1}$]	λ_{em} [nm]	Φ_{fl}[a] [%]	f [b]
**diSO$_2$-PA-a**	*n*-hexane	496	59 600	528	92	0.80
	DCM	503	55 300	560	93	0.77
	ACN	498	57 900	570	1.2	0.86
	MeOH	498	60 100	568	1.9	0.89
**SN-PA-a**	*n*-hexane	557	32 900	693	3.6	0.40
	DCM	565	32 400	708	2.1	0.42
	ACN	561	30 800	nd	nd	0.41
	MeOH	560	30 700	nd	nd	0.40
**PON-PA**	*n*-hexane	700	15 100	864	0.075	0.30
	DCM	725	15 300	909	0.190	0.30
	ACN[c]	719	-	936	0.024	-
	MeOH	740	13 700	947	0.014	0.27
**SO$_2$N-PA-a**	*n*-hexane	718	16 300	899	0.023	0.33
	DCM	757	13 800	896	0.028	0.28
	ACN	757	16 000	989	0.007	0.31
	MeOH	758	15 300	966	0.006	0.31
**CON-PA**	*n*-hexane	852	9 900	nd	nd	0.18
	DCM	924	9 400	nd	nd	0.18
	ACN	926	8 300	nd	nd	0.15
	MeOH	978	8 100	nd	nd	0.15
**diSO$_2$-PA-c**	*n*-hexane	550	104 900	567	81	0.85
	DCM	559	92 800	622	71	0.80
	ACN	553	84 300	660	36	0.89
	MeOH	551	85 700	648	25	0.85
**SN-PA-c**	DCM	669	40 100	nd	nd	0.56
**SO$_2$N-PA-c**	*n*-hexane	805	15 300	1002	0.013	0.27
	DCM	855	16 200	1066	0.005	0.30
	ACN	858	16 500	1102	0.003	0.30
	MeOH	856	16 100	1068	0.003	0.31
**SN-PA-b**	DCM	568	43 700	nd	nd	0.72
**SO$_2$N-PA-b**	*n*-hexane	717	16 800	900	0.016	0.33
	DCM	761	14 800	965	0.008	0.28
	ACN[c]	757	-	984	0.005	-
	MeOH	758	14 400	967	0.005	0.29

[a] Fluorescence quantum yields were determined using IR140 in DMSO (Φ_{fl} = 15%), fluorescein in 0.1M $NaOH_{aq}$ (Φ_{fl} = 91%) and sulforhodamine 101 in ethanol (Φ_{fl} = 90%) as references; [b] Experimental oscillator strengths calculated from the absorption spectra according to the equation: $f = 4.32 \cdot 10^{-9} \int \varepsilon(\tilde{\nu})\mathrm{d}\tilde{\nu}$; [c] Qualitative spectra were measured; nd – fluorescence not detected.

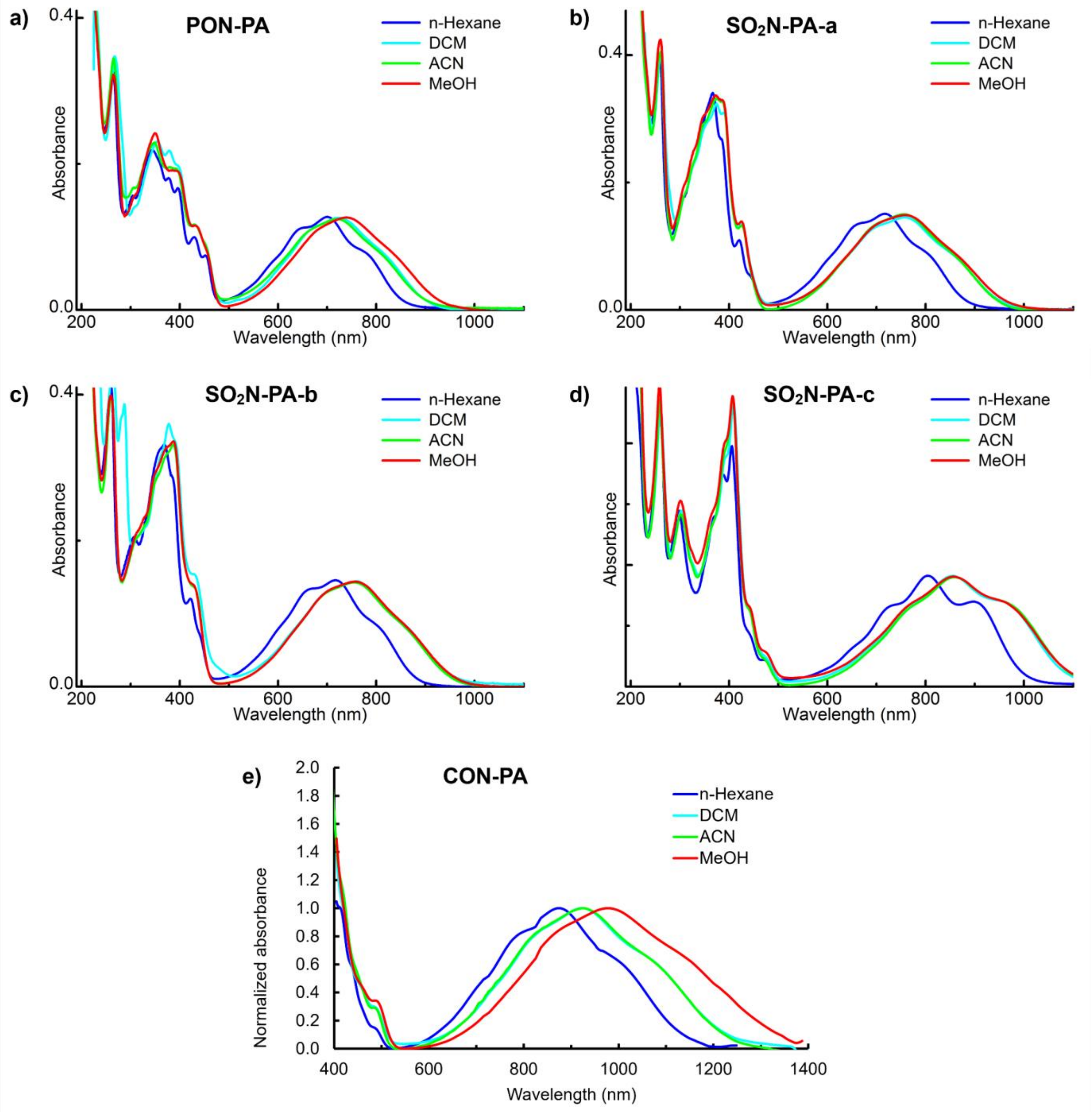

Figure S8. Absorption spectra of donor-acceptor pentacene analogues measured in four different solvents. a) **PON-PA**, b) **SO_2N-PA-a**, c) **SO_2N-PA-b**, d) **SO_2N-PA-c**, e) **CON-PA**.

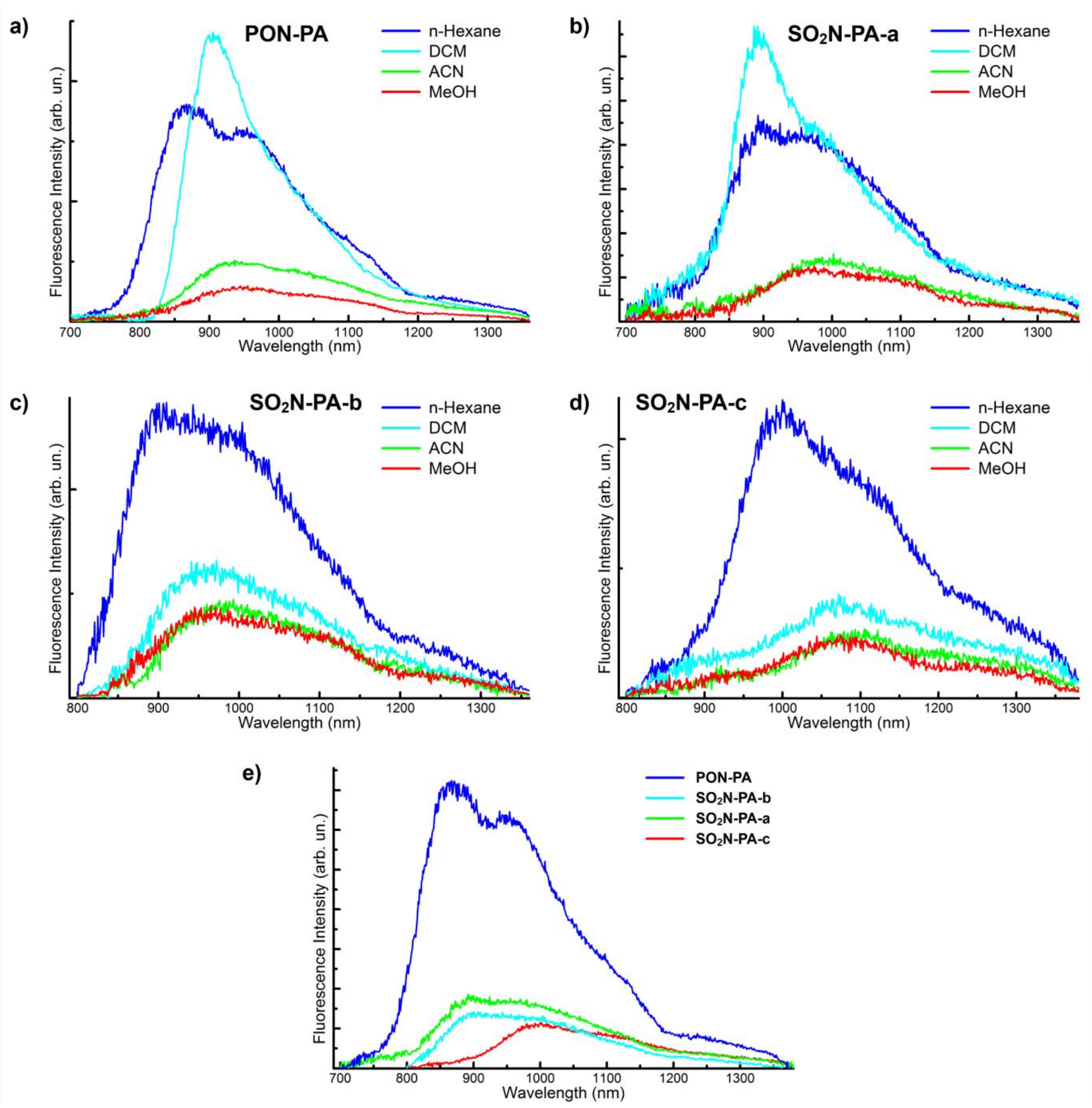

Figure S9. Fluorescence spectra of donor-acceptor pentacene analogues measured in four different solvents. a) **PON-PA**, b) **SO_2N-PA-a**, c) **SO_2N-PA-b**, d) **SO_2N-PA-c**. e) Comparison of the fluorescence spectra of all four compounds in *n*-hexane.

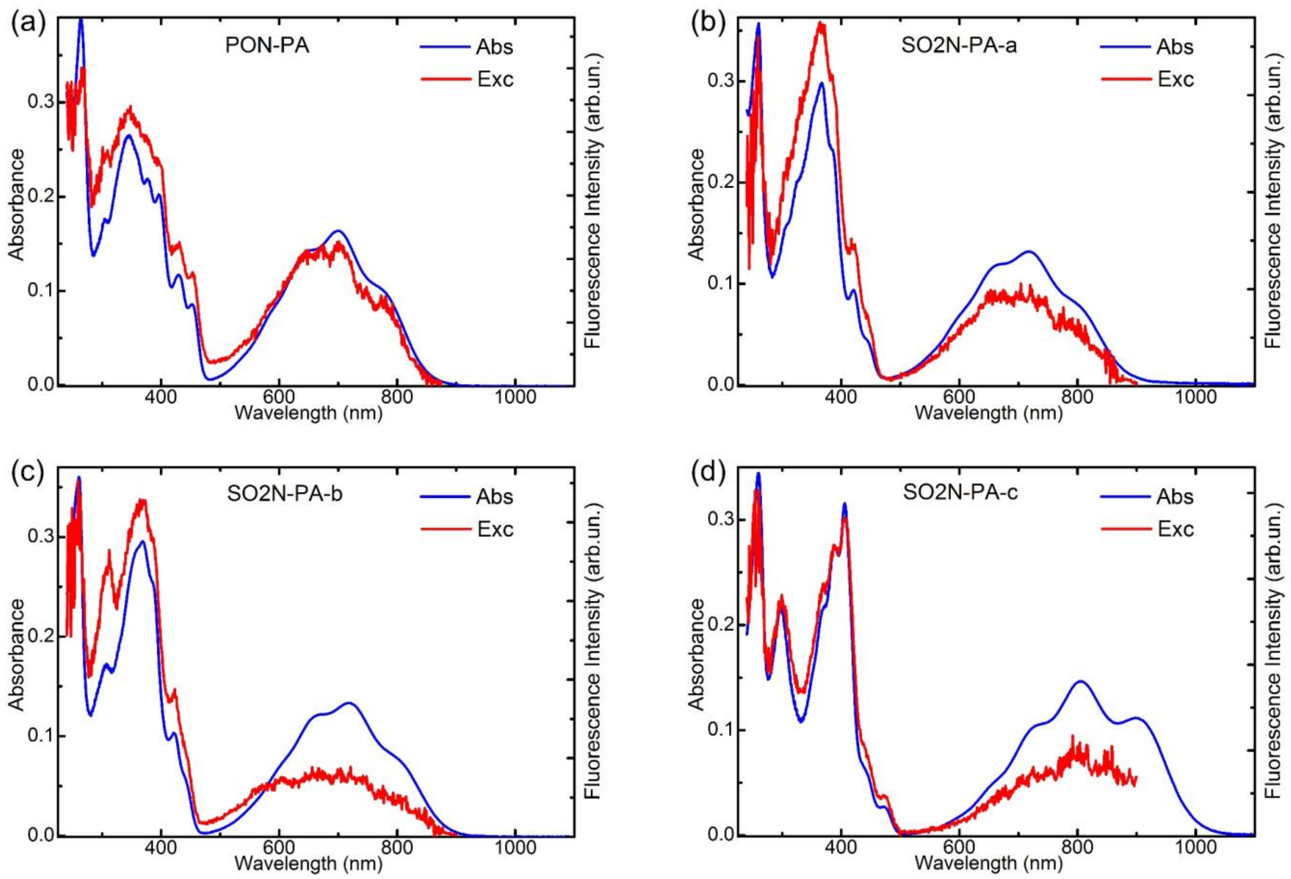

Figure S10. Comparison of the absorption spectra (blue lines) and fluorescence excitation spectra (red lines) of the compounds in *n*-hexane: (a) **PON-PA**, λ_{obs} = 900 nm; (b) **SO_2N-PA-a**; λ_{obs} = 950 nm; (c) **SO_2N -PA-b**; λ_{obs} = 950 nm, (d) **SO_2N -PA-c**, λ_{obs} = 1000 nm. Fluorescence excitation spectra were recorded using wide slits (5 nm for excitation and 20 nm for emission). Excitation spectra were collected over the full 240–900 nm range available on the FLS 1000 spectrophotometer (Edinburgh Instruments).

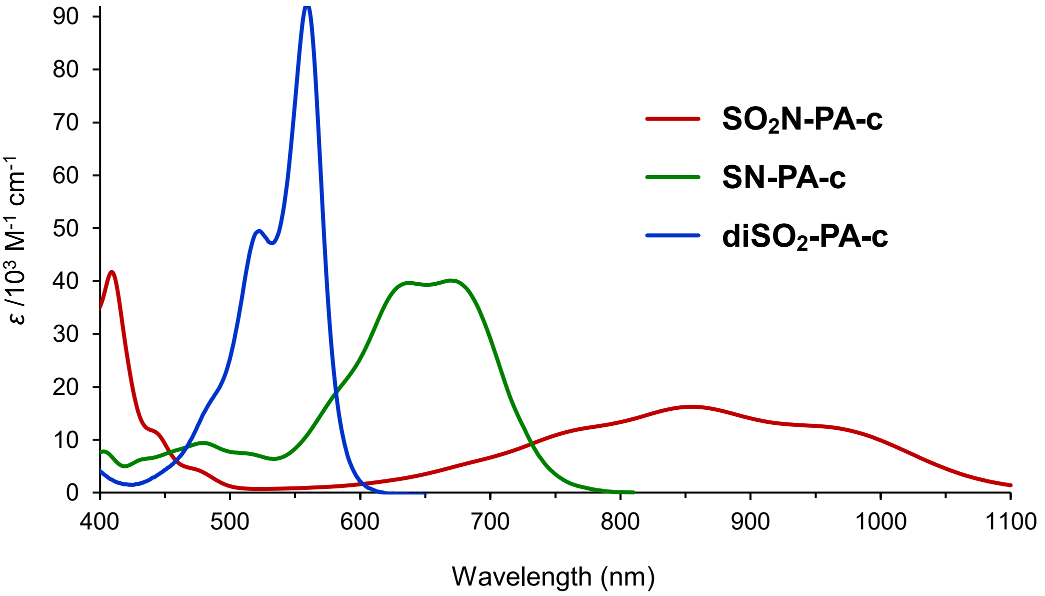

Figure S11. Comparison of absorption spectra of compounds **SO_2N-PA-c**, **SN-PA-c** and **diSO_2-PA-c** in dichloromethane.

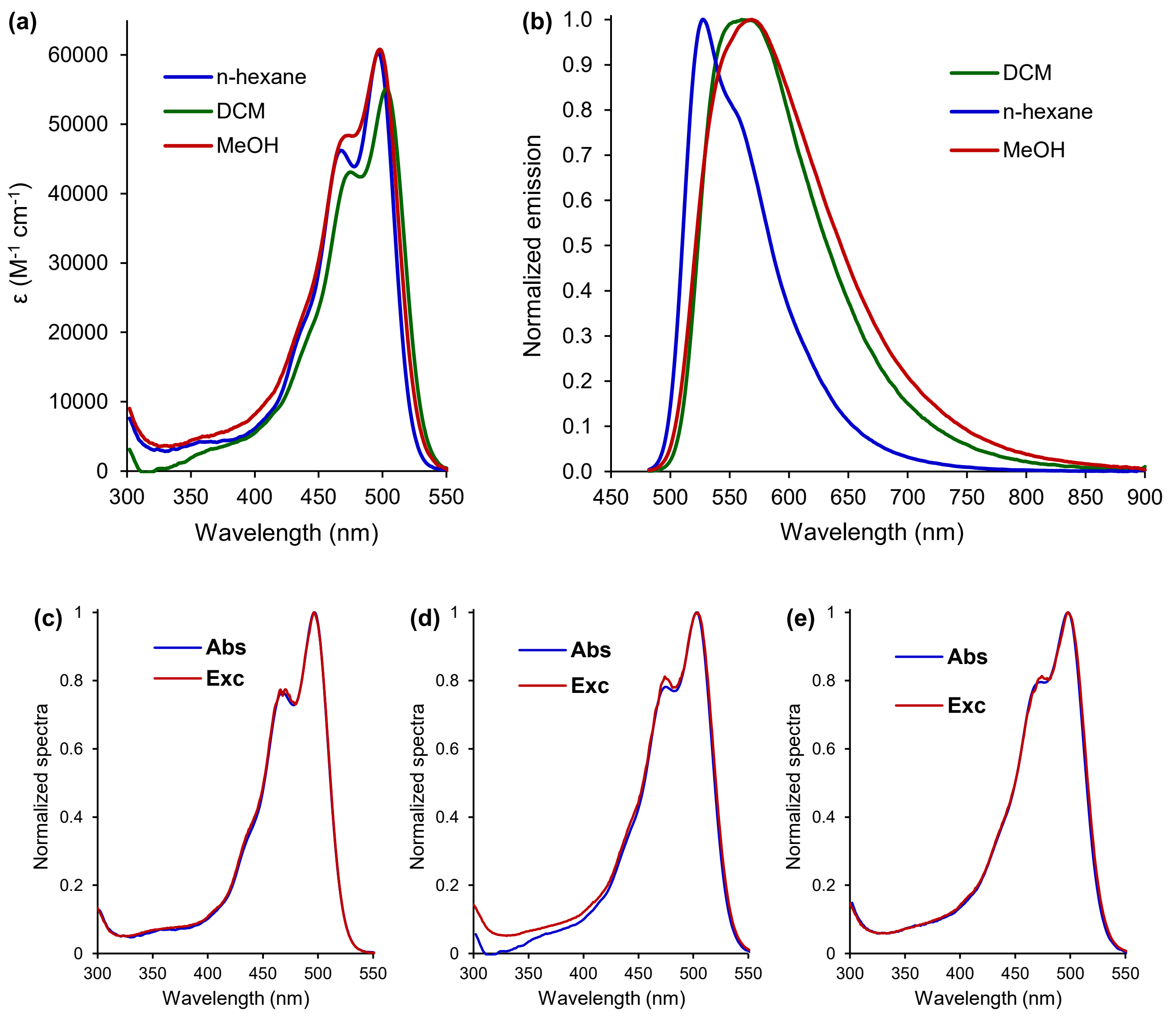

Figure S12. Absorption (a) and normalized fluorescence (b) spectra of **diSO$_2$-PA-a** in three different solvents. Comparison of the absorption spectra (blue lines) and fluorescence excitation spectra (λ_{obs} = 580 nm, red lines) of **diSO$_2$-PA-a** in (c) *n*-hexane, (d) dichloromethane, and (e) methanol.

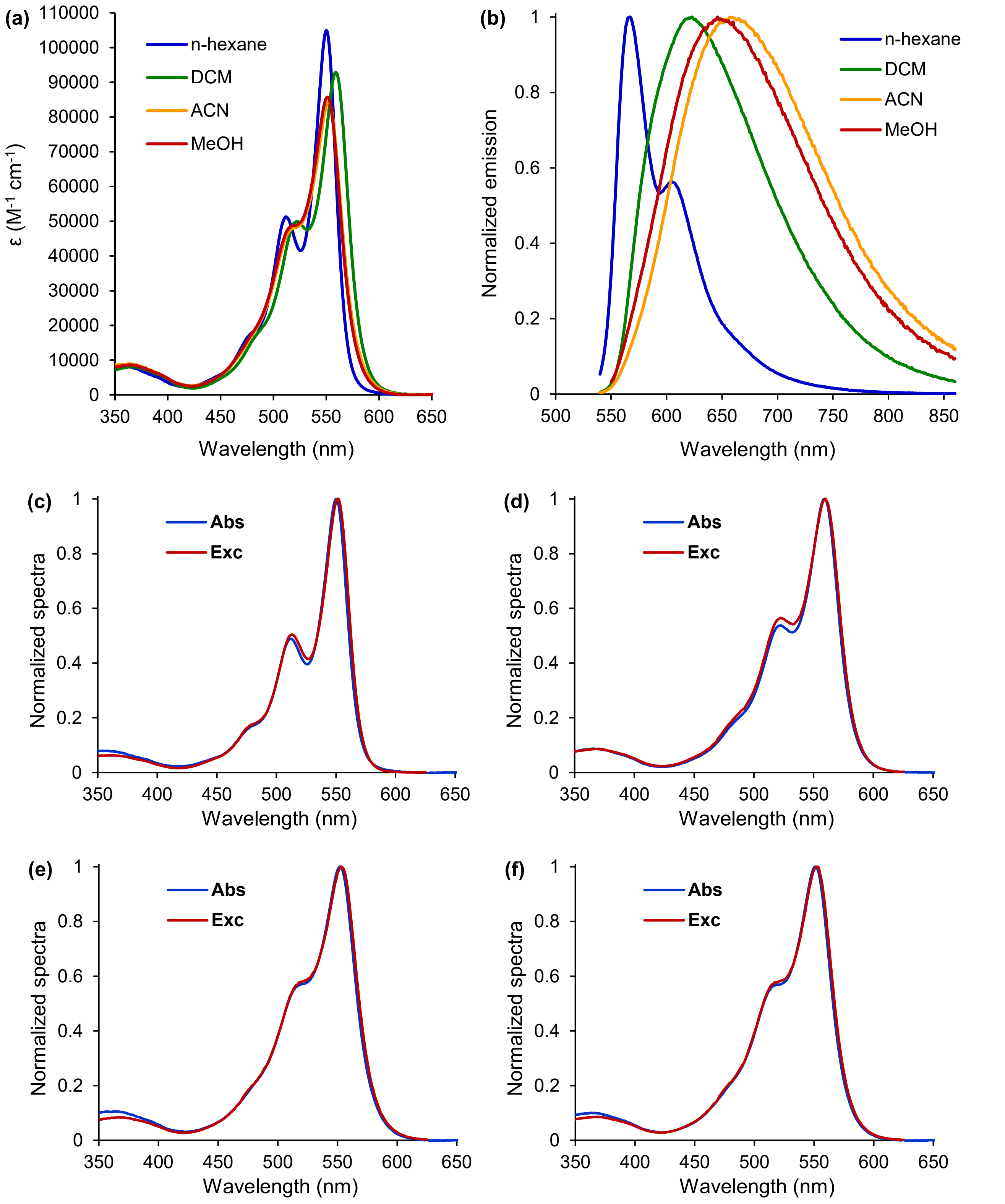

Figure S13. Absorption (a) and normalized fluorescence (b) spectra of **diSO$_2$-PA-c** in four different solvents. Comparison of the absorption spectra (blue lines) and fluorescence excitation spectra (λ_{obs} = 640 nm, red lines) of **diSO$_2$-PA-c** in (c) *n*-hexane, (d) dichloromethane, (e) acetonitrile, and (f) methanol.

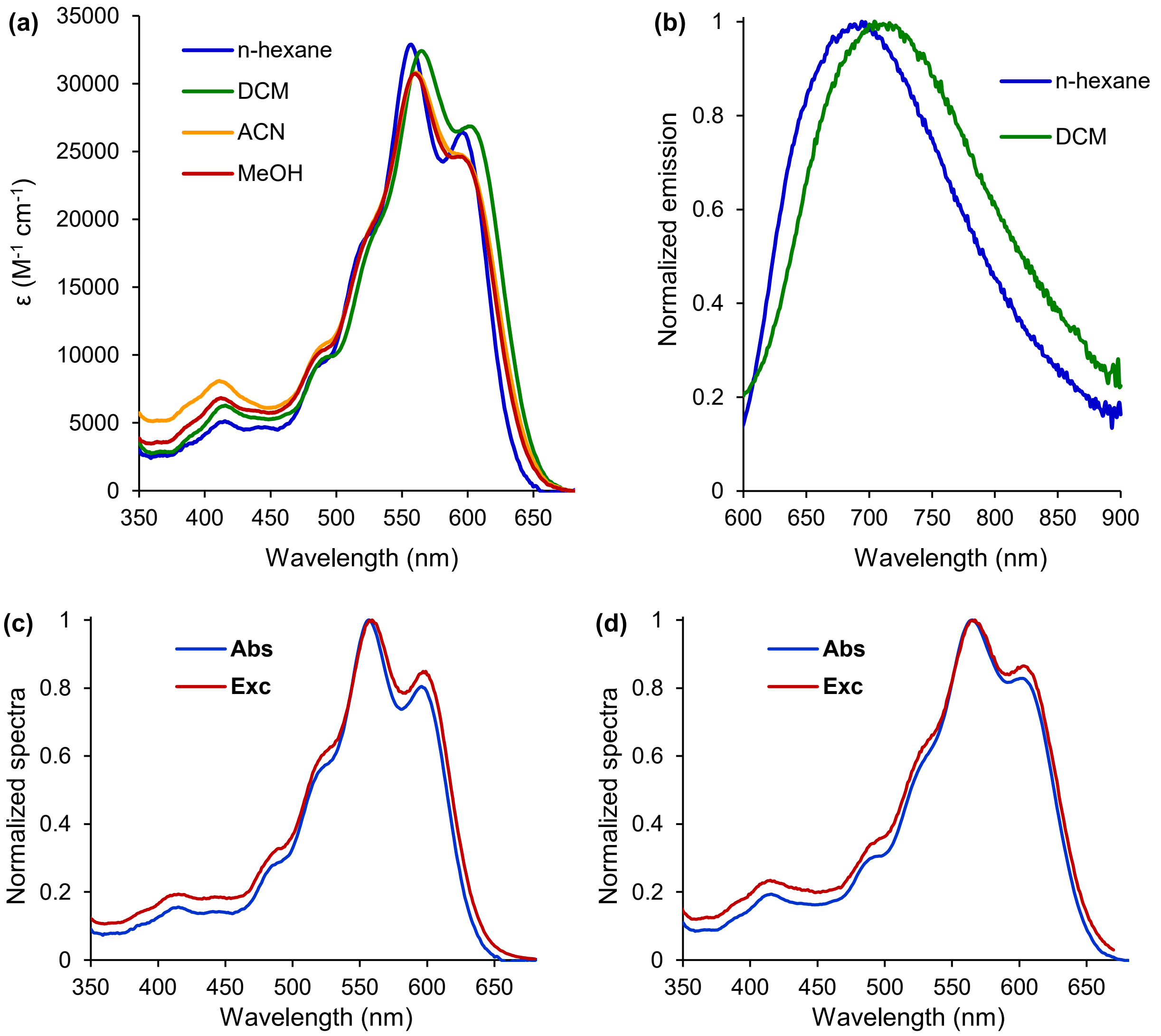

Figure S14. Absorption (a) and normalized fluorescence (b) spectra of **SN-PA-a** in different solvents. Comparison of the absorption spectra (blue lines) and fluorescence excitation spectra (λ_{obs} = 700 nm, red lines) of **SN-PA-a** in (c) *n*-hexane, and (d) dichloromethane.

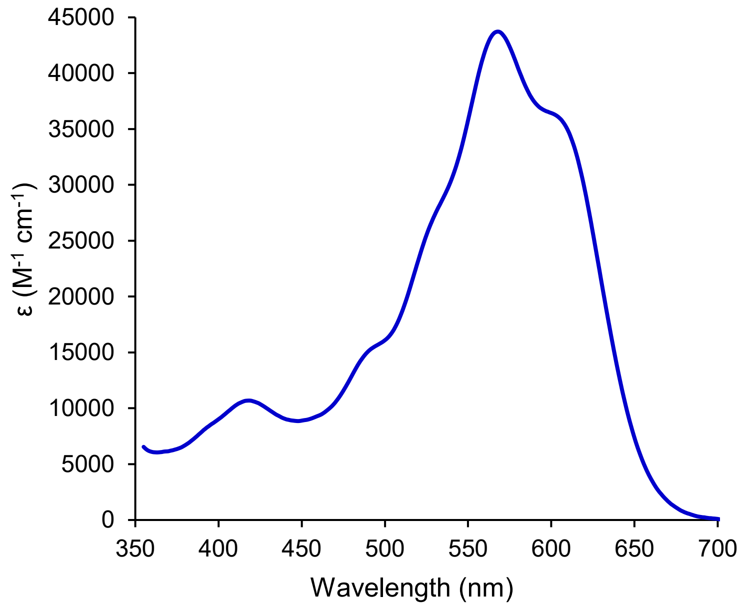

Figure S15. Absorption spectrum of **SN-PA-b** in dichloromethane.

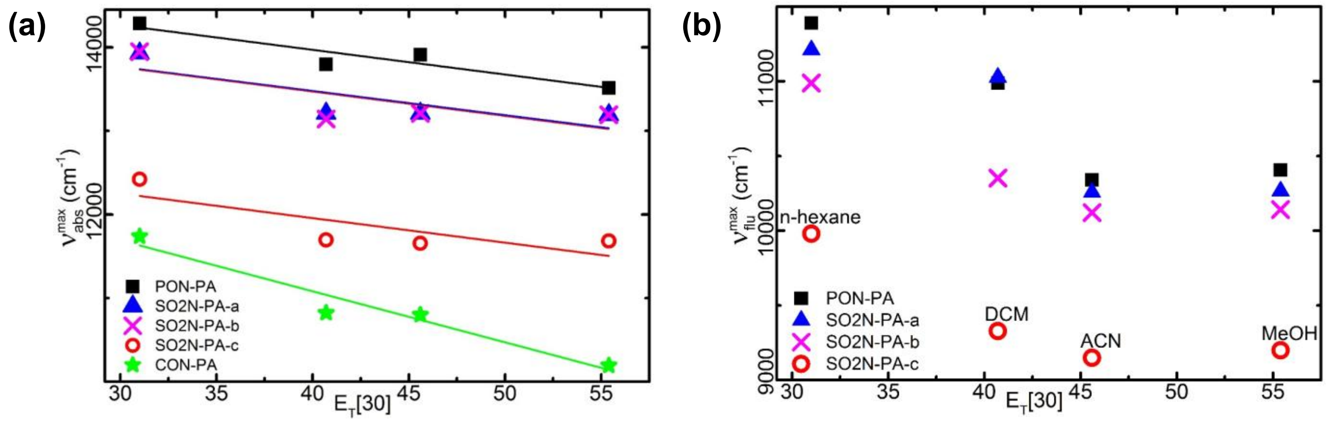

Figure S16. Wavenumber corresponding to the maximum of the (a) absorption and (b) emission spectrum, ν_{max}, plotted against Reichert's $E_T(30)$ solvent polarity values taken from the literature.[10] Solid lines represent linear fits to the solvatochromic data. The calculated slopes are -29.5±8, -28.9±14, 29.1±17, 29.4±15 and -60±10 for **PON-PA**, **SO_2N-PA-a**, **SO_2N-PA-b**, **SO_2N-PA-c**, and **CON-PA** respectively.

Comparison of fluorescence quantum yields with representative NIR/NIR-II dyes

The compounds reported in this study were not designed as optimized NIR fluorophores. The observed NIR absorption and emission result from strong push-pull electronic polarization, which is central to the present investigation of the electronic structure of donor-acceptor pentacene analogues. Molecular design was guided primarily by considerations of stability, solubility, and synthetic accessibility, rather than by maximization of fluorescence efficiency.

Nevertheless, given the very long emission wavelengths (~900–1100 nm), it is informative to place the measured fluorescence quantum yields in the context of representative NIR and NIR-II dyes reported in the literature.[11,12] In this spectral region, fluorescence quantum yields in homogeneous solution are commonly in the sub-percent range and are often enhanced only upon rigidification or binding to specific environments. Moreover, reported quantum yields beyond ~900 nm are subject to significant experimental uncertainty, as they are typically determined by relative methods using reference dyes such as IR-26, whose reported quantum yield varies widely in the literature (ca. 0.05–0.5%).[13]

Table S4. Representative small molecule NIR fluorophores (R = -$(CH_2)_4SO_3Na$ or -$(CH_2)_4SO_3^-$)

Name	Structure	λ_{em} [nm]	Φ_{fl} [%] (solvent)	Ref
**CH-4T**	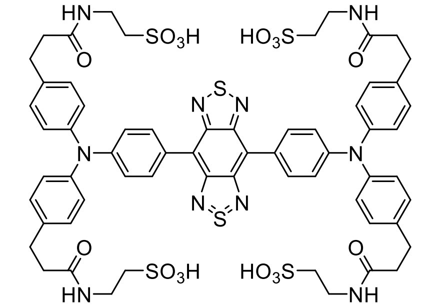	1055<br>1000	0.01–0.1 (PBS)<br>0.5–11 (FBS)	[13]
**Chrom7**	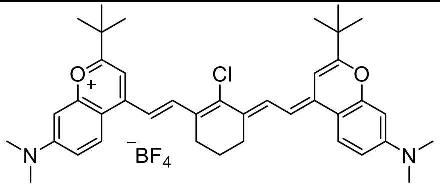	996	1.7 (DCM)	[14]
**FD-1080**	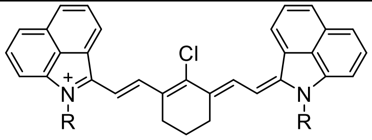	1053<br>1080	0.31 (EtOH)<br>5.9 (FBS)	[15]
**LZ-1105**	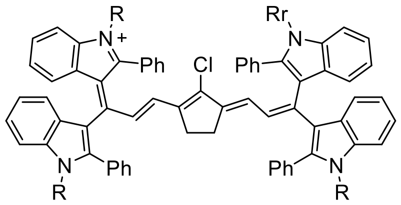	1075<br>1105<br>1105	1.2 (MeOH)<br>0.03 (PBS)<br>1.7 (mice blood)	[16]

Photostability

Diluted solutions of the compounds in methanol were irradiated using a continuous-wave (CW) 777 nm laser at a power of 1.5 W for 60 minutes in standard 1x1 cm quartz cuvettes (3.5 mL volume) without stirring. Absorption spectra were recorded immediately before and after irradiation. The comparison shown below demonstrates the exceptional photostability of all tested compounds. The negligible differences between the pre- and post-irradiation spectra suggest that the photostability exceeds several tens of watt-hours (Wh), significantly surpassing that of typical laser dyes.

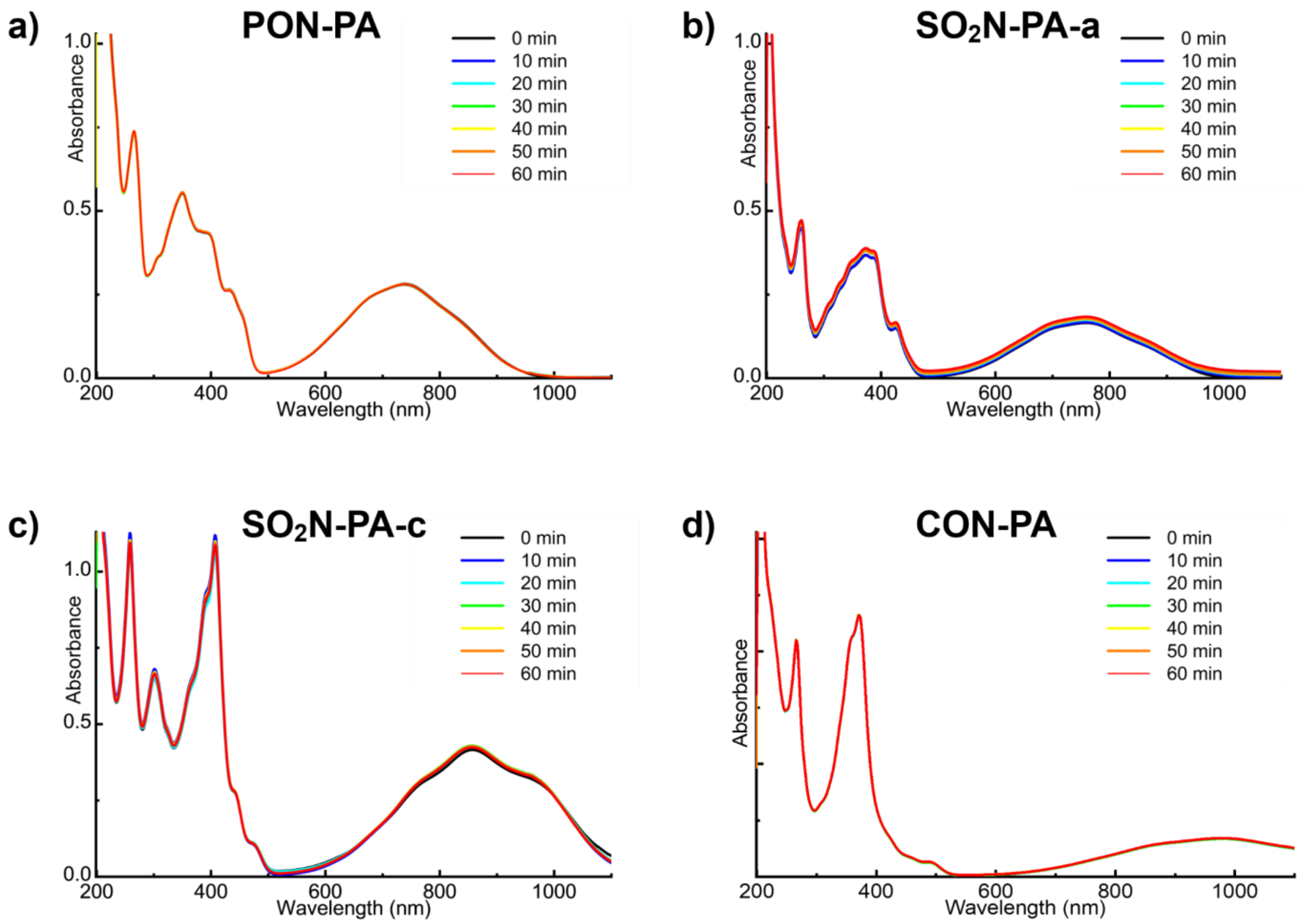

Figure S17. Absorption spectra of DA-pentacene solutions in methanol, recorded at 10-minute intervals during irradiation with a 777 nm laser (power: 1.5 W).

Chemical stability

Solid samples of all DA-pentacene analogues, as well as their donor-donor and acceptor-acceptor counterparts (**SN-PA** and **diSO$_2$-PA** derivatives) are in general exceptionally stable under ambient conditions. No deterioration has been observed upon prolonged exposure to ambient light (normal laboratory lighting, i.e. neutral white LED lamps), air, or atmospheric moisture (relative humidity in our laboratories: 20–60%) at room temperatures (20–25 °C). For long-term storage, vials containing the solid samples were kept in a refrigerator (4–8 °C) without any additional precautions (e.g., sealing with Parafilm or argon flushing), as is customary in our laboratory. The chemical stability tests described in this and the following sections were performed on samples that had already been stored for 1–2 years after preparation, further confirming their exceptional stability.

Given that DA-pentacenes contain strongly polarized *para*-quinodimethane cores, reduced stability in solution – particularly in polar protic solvents – might be anticipated. Among the DA-pentacenes studied, signs of decomposition (decrease in absorbance) were observed only for **SO$_2$N-PA-b** in protic or wet apolar solvents, which was attributed to insufficient steric protection of the nucleophilic and electrophilic sites by the 4-*tert*-butylphenyl substituents (Scheme S2). On this basis, solution-phase stability tests were performed in methanol.

Stability of DA-Pentacenes in methanol solutions

Solutions of DA-pentacenes in methanol (neither dried nor degassed) were prepared under air in standard 1×1 cm four-window fluorescence cuvettes (solutions volume: ~3.0 mL). The concentrations were adjusted such that the lowest-energy absorption band had an absorbance of 0.20–0.25. In the case of **SO$_2$N-PA-c**, which exhibits insufficient solubility in methanol, the compound was first dissolved in dichloromethane and subsequently diluted 100-fold with methanol. Absorption spectra in the 300–1400 nm range were recorded immediately after sample preparation and then after 3 h, 6 h, and 5 days (Figure S18, Figure S19). Between measurements, the cuvettes containing the solutions were stored at room temperature under ambient light.

Acid-base chemistry

The presence of both electron-donating and electron-accepting substituents flanking the *para*-quinodimethane moiety renders the DA-pentacenes amphoteric (ambipolar), and they can therefore be expected to be sensitive to both acids (electrophiles) and bases (nucleophiles).

To probe the stability of the DA-pentacene analogues under acidic and basic conditions, dye solutions were prepared in methanol as described in the previous section "**Stability of DA-Pentacenes in methanol solutions**" (solution volume: ca. 3.0 mL). After recording the initial absorption spectra of freshly prepared solutions in neutral methanol, trifluoroacetic acid (TFA, 1.0 M in methanol, 3.0 µL) was added to each cuvette using a micrometric pipette, resulting in an acid concentration of approximately 1.0 mM. As the observed color change from green to yellow was not instantaneous, the samples were equilibrated for 10 min prior to recording the absorption spectra of acidified DA-pentacenes. Subsequently, the acidified solutions were

neutralized and rendered basic by addition of 2 equiv. of triethylamine (TEA, 1.0 M in methanol, 6.0 µL), yielding basic solutions containing free TEA (c ≈ 1.0 mM) and the TFA·TEA salt (c ≈ 1.0 mM). Absorption spectra were recorded immediately after base addition and again after 2 h and 5 days (Figure S20, Figure S21). For the least stable compound, **SO_2N-PA-b**, stability towards bases was examined separately in the presence of TEA only (c ≈ 1.0 mM; Figure S22).

Stability measurements results and rationalization

Key observations can be summarized as follows:

- **Neutral methanol.** All DA-pentacenes are stable in neutral methanol under air, showing no spectral changes over 5 days (Figure S18, Figure S19), with the exception of **SO_2N-PA-b**, for which the NIR absorption band decreases by approximately 40% after 5 days (Figure S18c).
- **Acidic conditions.** In the presence of TFA (1.0 mM) NIR absorption band of the DA-pentacenes disappears almost completely, yielding yellow-colored solutions with a new absorption band centered at ~450 nm (Figure S20, Figure S21). **SO_2N-PA-c** represents a notable exception, as its NIR absorption maximum is reduced by only ~10% under the same conditions (Figure S20d).
- **Neutralization and basification.** Neutralization of the acid followed by basification with TEA leads to a slow but complete recovery of NIR absorption of all DA-pentacenes bearing 2,6-dimethoxyphenyl substituents (**PON-PA**, **SO_2N-PA-a**, **CON-PA**). In contrast, for **SO_2N-PA-b**, which contains less sterically protective 4-*tert*-butylphenyl substituents, the solution initially becomes nearly colorless and only partially recovers NIR absorption after 5 days (Figure S20c). For the TIPS-ethynyl-substituted derivative **SO_2N-PA-c,** the absorbance does not further change upon basification and remains at the same level as observed in the presence of TFA (Figure S20d).
- **Basic conditions only.** In the presence of TEA alone, **SO_2N-PA-b** also exhibits a decrease in NIR absorption (by ~30%), although to a slightly lesser extent than in neutral methanol.

Rationalization. These results demonstrate both the reversibility of protonation for DA-pentacenes bearing bulky aryl substituents (2,6-dimethoxyphenyl) and their high stability in neutral solvents as well as under basic conditions. The yellow coloration of the protonated species most likely arises from protonation within the acceptor-containing ring, leading to the formation of acridinium-type salts (Scheme S2). The corresponding absorption spectra closely resemble those reported in the literature (e.g.[17]).

For compounds containing bulky aryl substituents (2,6-dimethoxyphenyl in **PON-PA**, **SO_2N-PA-a**, **CON-PA**), neutralization of the acid fully restores the original green DA-pentacene chromophores. In contrast, when steric protection is insufficient – as in **SO_2N-PA-b** (R = 4-*tert*-butylphenyl) and **SO_2N-PA-c** (R = −C≡C-TIPS) – acid neutralization does not regenerate the parent DA-pentacenes. Instead, nucleophilic addition of water or methanol to the nitrogen-containing ring occurs, yielding colorless species composed of three non-conjugated aromatic benzene rings (Scheme S2). An analogous reaction pathway is operative in neutral protic solvents.

In conclusion, DA-pentacenes bearing bulky aryl substituents or TIPS-ethynyl groups **exhibit exceptional stability in neutral and basic solutions under air and ambient light**. Under acidic conditions, they undergo protonation to form acridinium-type salts, a process that is reversible when bulky aryl substituents are present.

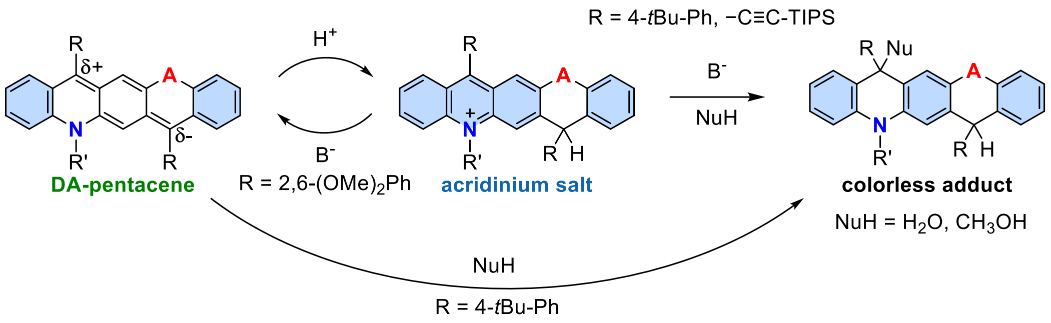

Scheme S2. Proposed rationale for the observed stability behavior. Protonation of DA-pentacenes leads to the formation of yellow acridinium-type salts. When sufficient steric protection of the reactive 5- and 12-positions is present, subsequent base addition results in slow deprotonation and regeneration of the parent DA-pentacenes. In contrast, for derivatives bearing less bulky aryl substituents, the acridinium salts undergo nucleophilic addition of methanol or water, yielding colorless adducts.

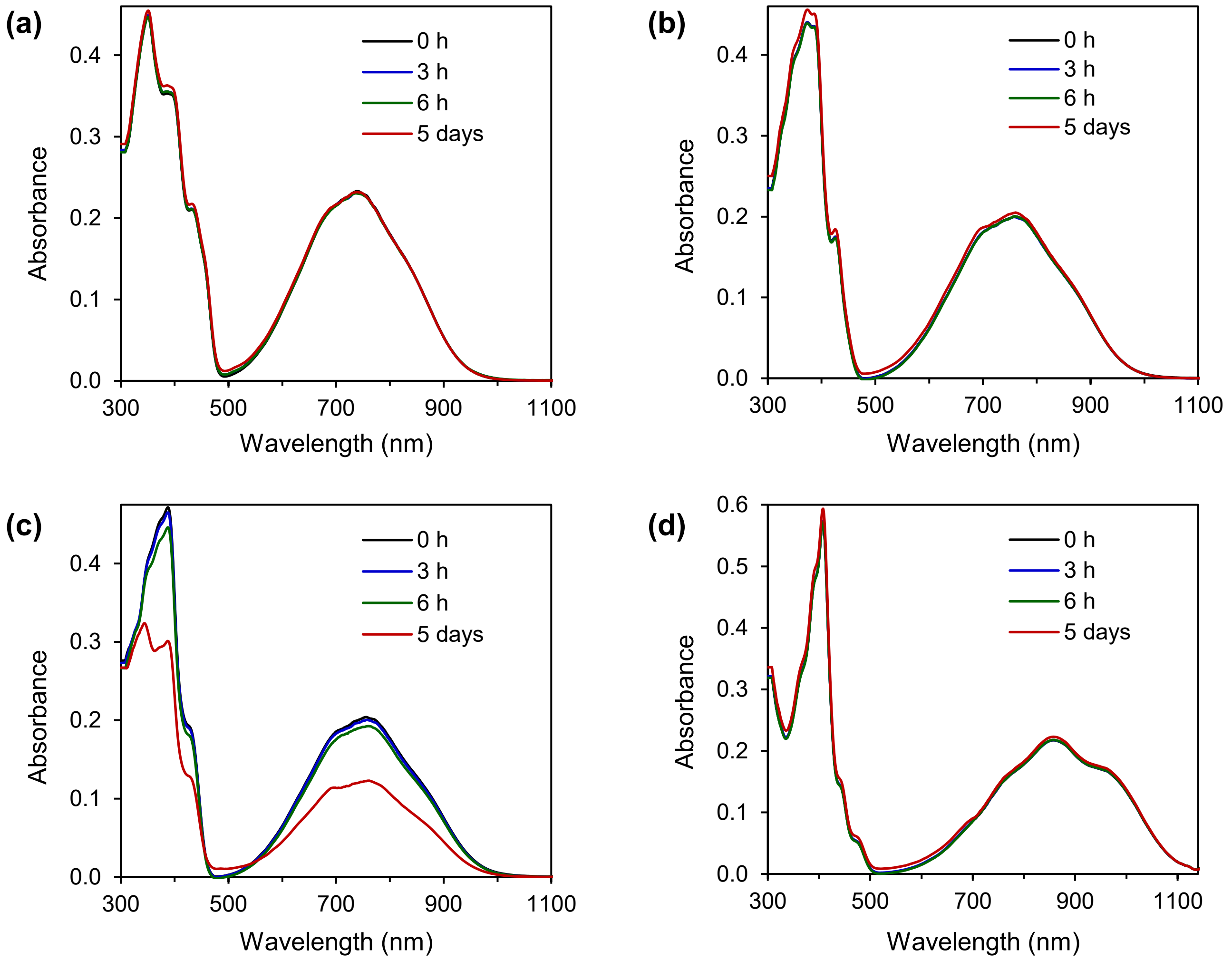

Figure S18. UV-Vis-NIR absorption spectra of (a) **PON-PA**, (b) **SO_2N-PA-a**, (c) **SO_2N-PA-b**, and (d) **SO_2N-PA-c**, in methanol recorded over time after dissolution.

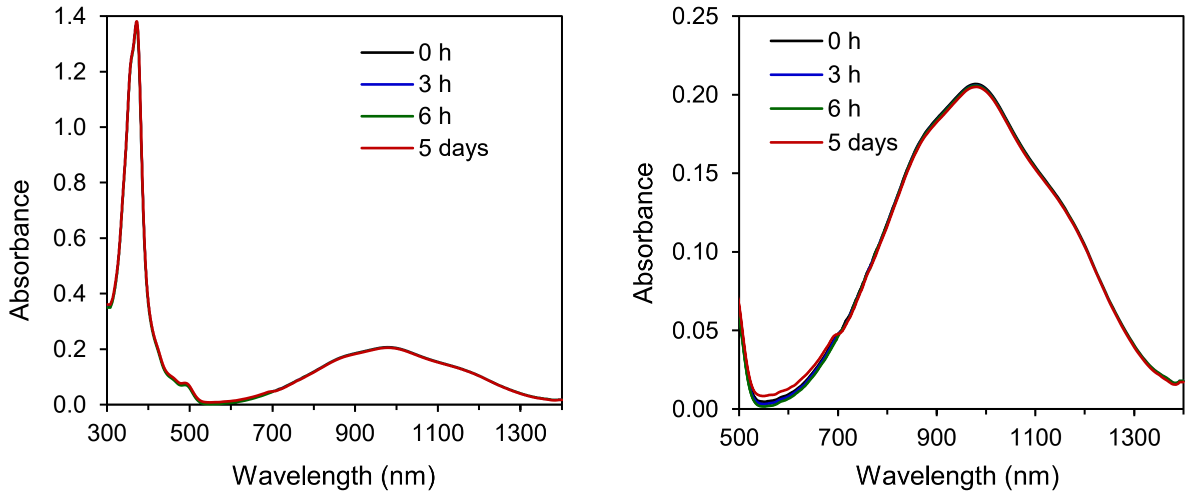

Figure S19. UV-Vis-NIR absorption spectra of **CON-PA** in methanol recorded over time after dissolution. (left) Full spectral range (300–1400 nm); (right) magnified lowest-energy absorption band.

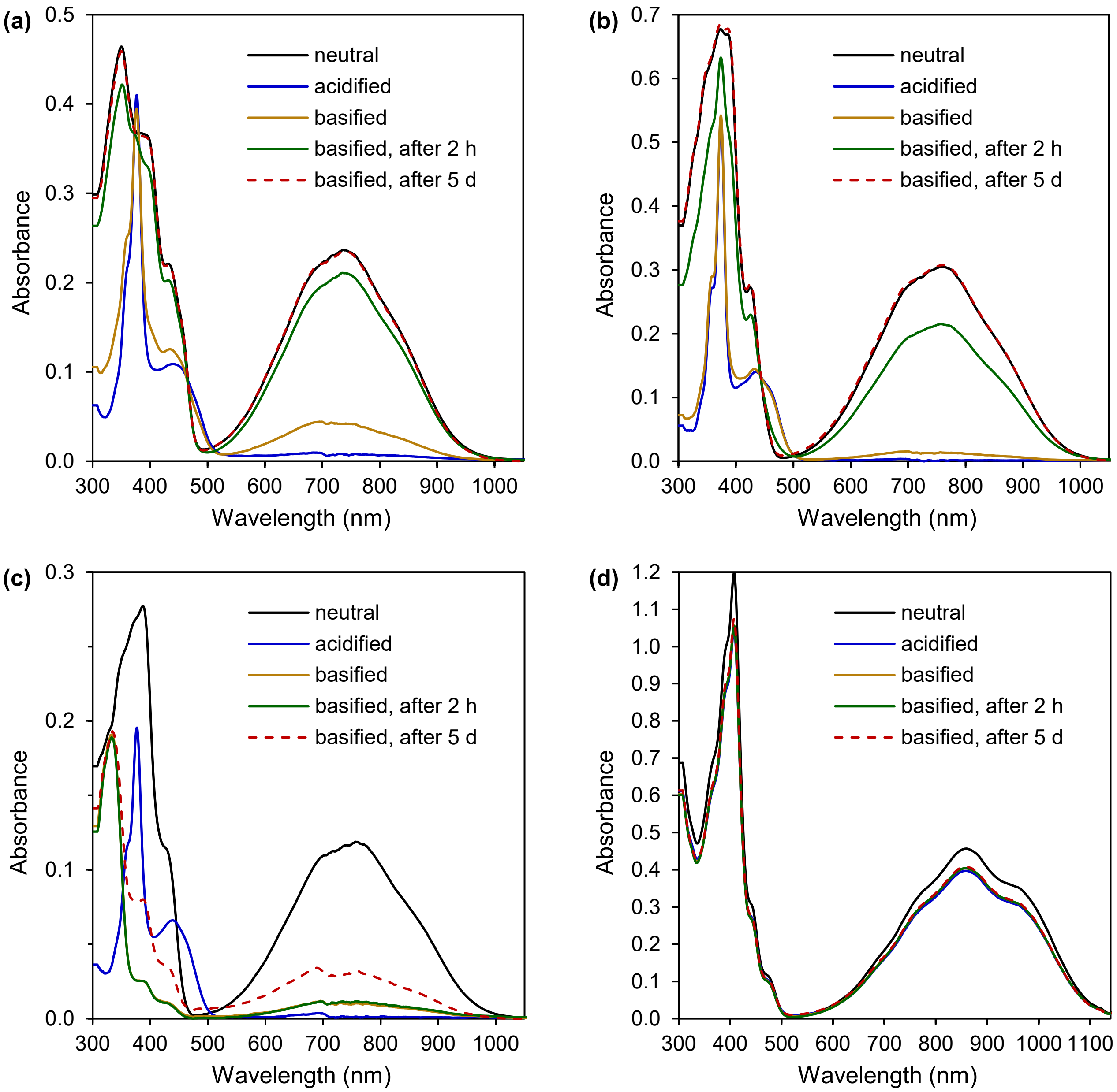

Figure S20. Changes in the UV-Vis-NIR absorption spectra of (a) **PON-PA**, (b) **SO_2N-PA-a**, (c) **SO_2N-PA-b**, and (d) **SO_2N-PA-c**, in methanol upon addition of trifluoroacetic acid and triethylamine: i. **neutral** (black) – freshly prepared solution, ii. **acidified** (blue) – after addition of 1.0 mM TFA and equilibration for 10 min; iii. **basified** (gold) – after addition of 2.0 mM of TEA and equilibration for 10 min; iv. **basified, after 2 h** (green); v. **basified, after 5 days** (red dashed line).

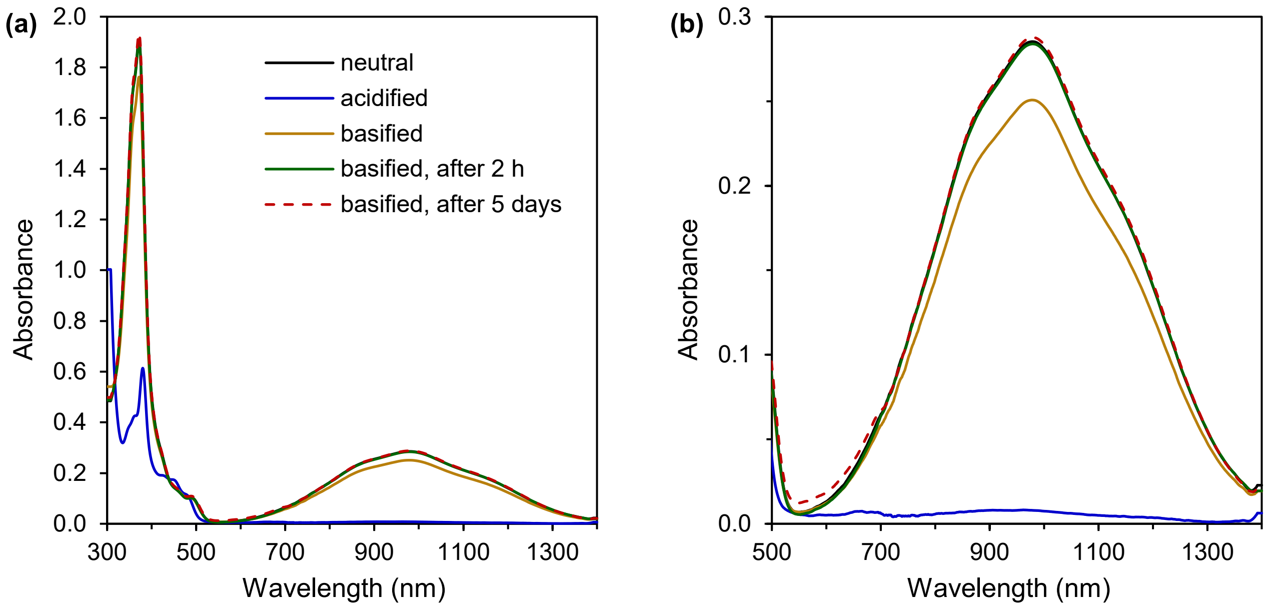

Figure S21. Changes in the UV-Vis-NIR absorption spectra of **CON-PA** in methanol upon addition of trifluoroacetic acid and triethylamine: i. **neutral** (black) – freshly prepared solution, ii. **acidified** (blue) – after addition of 1.0 mM TFA and equilibration for 10 min; iii. **basified** (gold) – after addition of 2.0 mM of TEA and equilibration for 10 min; iv. **basified, after 2 h** (green); v. **basified, after 5 days** (red dashed line). (a) Full spectral range (300–1400 nm); (b) magnified lowest-energy absorption band.

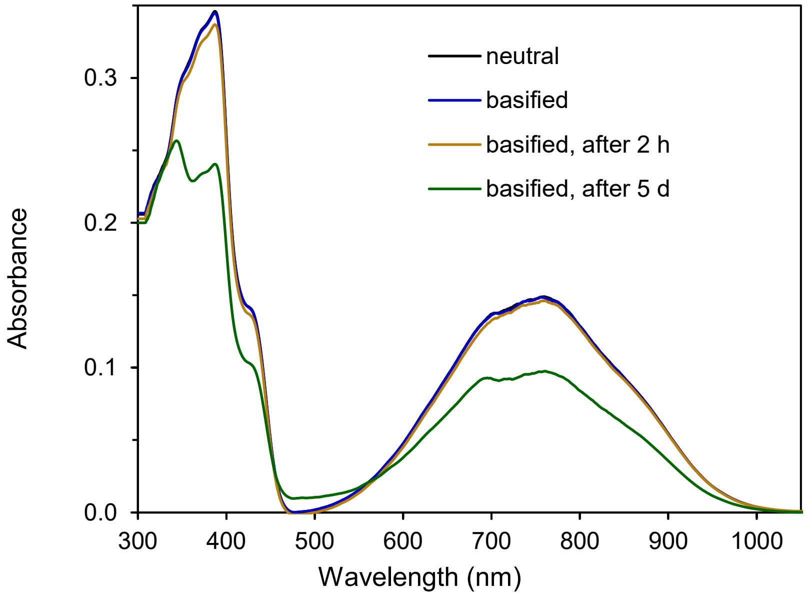

Figure S22. Changes in the UV-Vis-NIR absorption spectra of **SO_2N-PA-b** in methanol upon addition of triethylamine: i. **neutral** (black) – freshly prepared solution, ii. **basified** (blue) – after addition of 1.0 mM of TEA and equilibration for 10 min; iii. **basified, after 2 h** (gold); v. **basified, after 5 days** (green).

4. DFT calculations

Most of the theoretical studies were performed on simplified molecules, where aryl and alkyl substituents were replaced with hydrogen atoms or methyl groups (Scheme S3). All calculations were carried out in Gaussian 16[18] and started with the identification of the appropriate functional for the density-functional theory (DFT) and time-dependent DFT (TD-DFT) computations, to reliably model absorption spectra and excited state features. Several unsymmetrical pentacene analogues with known experimental absorption spectra in different solvents were selected as a benchmark set: **PON-PA**, **SO_2N-PA-a**, **SO_2N-PA-c** and **SN-PA-a**, along with two literature reference molecules: pentacene (**PA**, λ_{abs} = 590 nm)[19] and indeno[1,2-*b*]fluorene (**IndFlu**, λ_{abs} = 516 nm)[20]. In the latter case the experimental absorption peak corresponds to S_0-S_2 transition, since the S_0-S_1 transition is forbidden (f = 0.0000) according to our TD-DFT calculations. Simplified versions of these compounds (Scheme S3) were optimized at the M06-2X[21] / def2-TZVPP[22] level of theory using the SMD[23] solvation model: **PON-PA**, **SO_2N-PA-a** and **SO_2N-PA-c** were optimized in *n*-hexane and methanol, whereas **SN-PA-a**, **PA** and **IndFlu** – in dichloromethane as the solvents.
The geometries thus optimized were submitted to TD-DFT calculations with the same basis set (def2-TZVPP) and four DFT functionals: CAM-B3LYP, M06-2X, ωB97-XD, and LC-ωHPBE, selected due to their overall good performances in the description of π-conjugated molecules in benchmark studies.[24–26] For the range separated functionals, different values of range separation parameter ω were tested. The comparison of the experimental and calculated excitation energies, including mean absolute errors and correlation coefficients, are summarized in Table S5. Interestingly, only the range-separated functional LC-ωHPBE[27,28] correctly predicted that **PON-PA** absorption band is redshifted relative to pentacene, and this occurred only when the range separation was within a range of ω = 0.1–0.3, whereas a value of ω = 0.2 gave the best correlation with the experimental energies and reasonable mean deviations of 0.25 eV (Table S5, Table S6). Therefore, the range-separated functional LC-ωHPBE with range separation parameter set to ω = 0.2 was chosen as the optimal and used throughout the manuscript. Stationary points in the lowest singlet state (S_0) of the simplified molecules (Scheme S3) were optimized at the LC-ωHPBE(ω=0.2)/def2-TZVPP level in different solvents using the SMD solvation model and characterized by frequency anal ysis (the number of imaginary frequencies: 0).
Excitation energies and the optimized geometries of the excited states were calculated using TD-DFT at the same level of theory (Table S7 – Table S9). Three solvents of different polarity were used to study the solvatochromic properties: *n*-hexane, dichloromethane, and methanol. The complete structures of the synthesized molecules were also optimized using this method.
The route sections for the Gaussian calculations, including geometry optimizations, frequency analyses, and TD-DFT calculations, together with the keywords employed (here dichloromethane as the solvent), are listed below.

Geometry optimization:

```
#p LC-wHPBE/Def2TZVPP opt=(tight) scrf=(smd,solvent=dichloromethane) iop(3/107=0200000000) iop(3/108=0200000000)
```

```
Frequency calculations:
#p LC-wHPBE/Def2TZVPP freq scrf=(smd,solvent=dichloromethane) iop(3/107=0200000000) iop(3/108=0200000000)
TD-DFT calculations:
#p LC-wHPBE/Def2TZVPP td(nstates=10) scrf=(smd,solvent=dichloromethane) iop(3/107=0200000000) iop(3/108=0200000000)
```

The optimized geometries are shown in Figure S24 – Figure S34 (simplified molecules) and Figure S23, Figure S35 – Figure S40 (complete structures); their Cartesian coordinates are provided as easily accessible *.xyz files, compressed into a ZIP archive and included as a separate supporting information file. Orbitals were visualized with Jmol,[29] and the 3D models with Mercury software.[5]

Bond lengths and dipole moments in Figure S24 – Figure S32 and Figure S35 – Figure S40 were visualized using custom Python scripts based on the Matplotlib library (matplotlib.pyplot).[30] The scripts automatically extract data from .xyz geometry files (*xyz2BL.py*) or Gaussian 16 .log files (*DipoleVisBL.py*), reorient the molecules to project them onto the figure plane with the π-system aligned horizontally and dipole moments (if present) pointing toward the top right. Bond lengths and dipole vectors are then printed onto the structures, and the resulting images are saved as .png files. The Python scripts *xyz2BL.py* and *DipoleVisBL.py* used for visualization of bond lengths and dipole moments are available at:

https://github.com/mrkgrb/DFT-visualizer

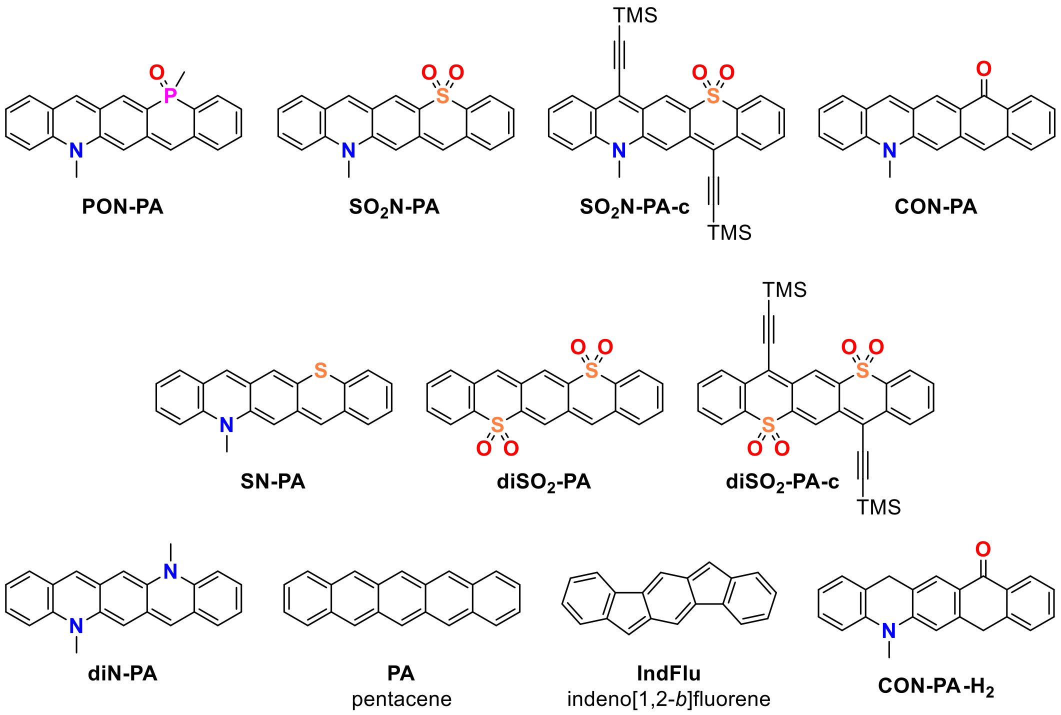

Scheme S3. Simplified structures of DA-pentacenes and reference compounds used for DFT calculations.

Table S5. Comparison of experimental and theoretical S_0-S_1 (except of **IndFlu**[a]) excitation energies calculated by TD-DFT using various functionals and different values of the range separation parameter (ω). Geometries of the simplified molecules were optimized using DFT at the M06-2X/def2-TZVPP level with the SMD solvation model. The same def2-TZVPP basis set and SMD model were used for the TD-DFT calculations. Default ω values for each functional are indicated in **bold**. Oscillator strengths for these excitations are listed in Table S6. The method highlighted in **bold green font** was selected as optimal and used throughout the manuscript.

Compound	Solvent	Experimental values		TD-DFT calculated excitation energies, E_{calc} [eV]																
				**Functional**	**cam-B3LYP**				**LC- ωHPBE**						**M06-2X**	**ωB97XD**				
		λ_{exp} [nm]	E_{exp} [eV]	**ω =**	0.11	0.22	**0.33**	0.44	0.05	0.10	**0.20**	0.30	**0.40**	0.50	–	0.05	0.10	0.15	**0.20**	0.25
**IndFlu**[a]	DCM	516[a]	2.403	Excitation energies E_{calc} [eV]	2.487	2.582	2.673	2.734	2.299	2.356	**2.543**	2.742	2.886	2.969	2.702	2.501	2.536	2.597	2.670	2.745
**PA**	DCM	590	2.101		2.000	2.152	2.237	2.282	1.707	1.886	**2.212**	2.392	2.483	2.518	2.237	1.988	2.090	2.206	2.301	2.371
**PON-PA**	Hex	709	1.749		2.073	2.199	2.320	2.405	1.820	1.881	**2.123**	2.384	2.575	2.694	2.318	2.093	2.136	2.216	2.316	2.417
**PON-PA**	MeOH	742	1.671		2.000	2.130	2.254	2.341	1.737	1.801	**2.051**	2.315	2.507	2.631	2.244	2.020	2.065	2.149	2.251	2.354
**SN-PA**	DCM	568	2.183		2.279	2.362	2.450	2.514	2.104	2.139	**2.300**	2.493	2.642	2.737	2.465	2.299	2.323	2.375	2.443	2.516
**SO_2N-PA**	Hex	733	1.691		1.997	2.126	2.250	2.337	1.740	1.802	**2.050**	2.315	2.509	2.634	2.246	2.018	2.062	2.145	2.248	2.351
**SO_2N-PA**	MeOH	763	1.625		1.895	2.031	2.157	2.245	1.626	1.695	**1.957**	2.220	2.412	2.541	2.140	1.915	1.965	2.055	2.161	2.265
**SO_2N-PA-c**	Hex	816	1.519		1.702	1.815	1.918	1.989	1.489	1.545	**1.763**	1.981	2.139	2.241	1.910	1.710	1.754	1.832	1.922	2.009
**SO_2N-PA-c**	MeOH	859	1.443		1.629	1.765	1.876	1.951	1.384	1.463	**1.730**	1.959	2.117	2.224	1.849	1.630	1.695	1.797	1.902	1.997
**Mean signed deviation**				**MSD [eV]**	0.17	0.30	0.40	0.48	-0.07	0.01	**0.25**	0.48	0.64	0.74	0.40	0.19	0.24	0.32	0.41	0.50
**Mean absolute deviation**				**MAD [eV]**	0.20	0.30	0.40	0.48	0.09	0.08	**0.25**	0.48	0.64	0.74	0.40	0.21	0.24	0.32	0.41	0.50
**Linear correlation coefficient**				**R^2**	0.83	0.84	0.79	0.74	0.82	0.90	**0.92**	0.84	0.74	0.63	0.80	0.81	0.84	0.86	0.83	0.79

[a] All data for **IndFlu** correspond to the S_0-S_2 transition; S_0-S_1 transition is forbidden (f = 0.0000).

Table S6. Oscillator strengths corresponding to the excitation energies listed in Table S5.

Compound	Solvent	Functional	cam-B3LYP				LC- ωHPBE						M06-2X	ωB97XD				
		ω	0.11	0.22	**0.33**	0.44	0.05	0.10	**0.20**	0.30	**0.40**	0.50	–	0.05	0.10	0.15	**0.20**	0.25
**IndFlu**[a]	DCM	Oscillator strengths *f*	0.699	0.768	0.828	0.872	0.547	0.595	0.725	0.848	0.945	1.016	0.828	0.712	0.740	0.785	0.835	0.885
**PA**	DCM		0.067	0.086	0.098	0.107	0.037	0.051	0.087	0.115	0.135	0.147	0.090	0.065	0.077	0.093	0.107	0.120
**PON-PA**	Hex		0.618	0.719	0.819	0.893	0.428	0.473	0.646	0.850	1.014	1.133	0.787	0.634	0.669	0.735	0.818	0.904
**PON-PA**	MeOH		0.548	0.650	0.751	0.826	0.365	0.409	0.585	0.791	0.957	1.076	0.720	0.561	0.598	0.667	0.753	0.841
**SN-PA**	DCM		1.000	1.086	1.168	1.228	0.788	0.836	1.003	1.173	1.304	1.399	1.144	1.020	1.047	1.100	1.164	1.229
**SO_2N-PA**	Hex		0.545	0.641	0.738	0.810	0.371	0.413	0.579	0.776	0.938	1.056	0.711	0.560	0.594	0.659	0.740	0.825
**SO_2N-PA**	MeOH		0.455	0.551	0.647	0.717	0.296	0.337	0.501	0.698	0.860	0.977	0.624	0.466	0.503	0.570	0.653	0.740
**SO_2N-PA-c**	Hex		0.543	0.634	0.718	0.779	0.383	0.427	0.589	0.766	0.908	1.011	0.689	0.549	0.587	0.652	0.729	0.806
**SO_2N-PA-c**	MeOH		0.428	0.512	0.592	0.651	0.293	0.330	0.479	0.650	0.789	0.888	0.568	0.432	0.468	0.530	0.605	0.681

[a] All data for **IndFlu** correspond to the S_0-S_2 transition; S_0-S_1 transition is forbidden (*f* = 0.0000).

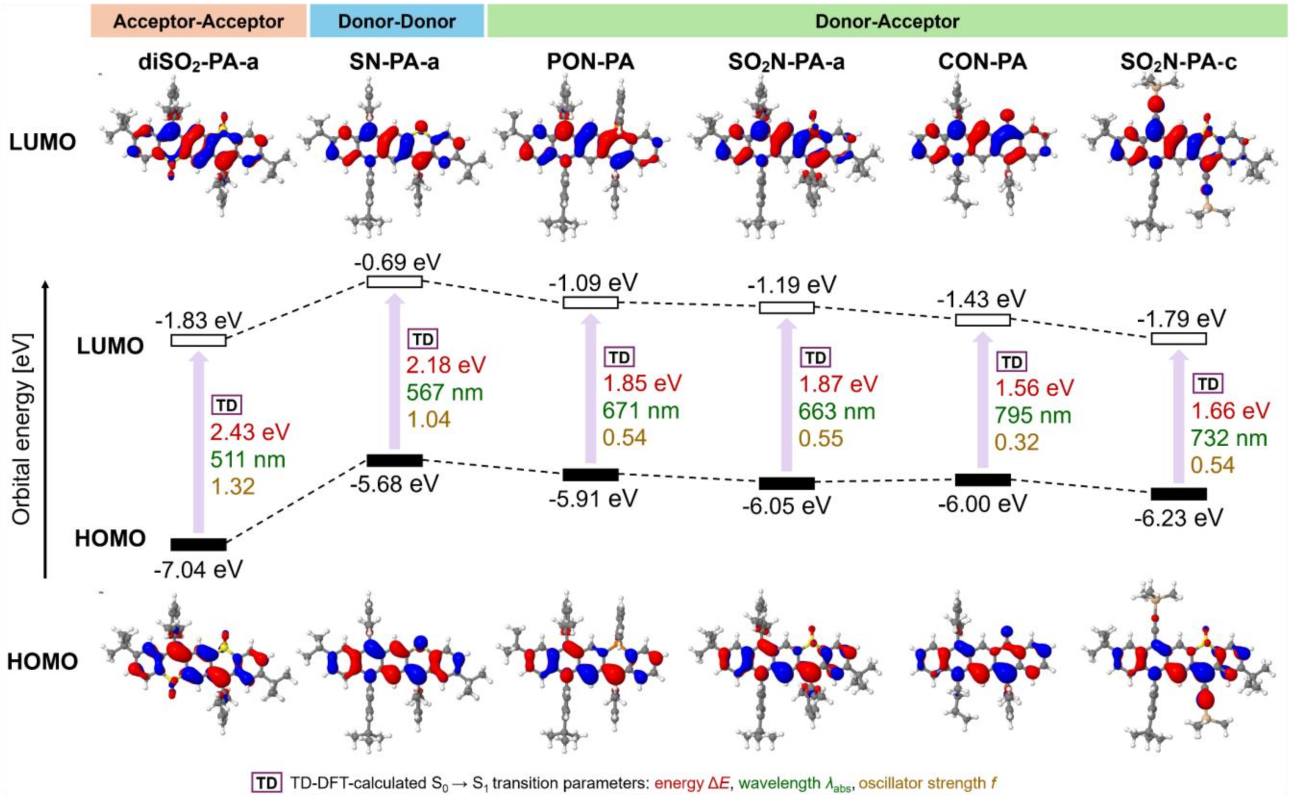

Figure S23. Summary of DFT and TD-DFT results obtained at the LC- ωHPBE (ω=0.2)/def2-TZVPP level of theory with the SMD solvation model (dichloromethane) for complete structures of DA-pentacenes and their double-donor and double-acceptor analogues. Canonical HOMO and LUMO orbitals are shown at the bottom and top of the figure, respectively. Orbital energies are displayed in the central energy diagram. TD-DFT-predicted parameters of the lowest vertical singlet excitation ($S_0 \rightarrow S_1$) are indicated: energy gap (ΔE, red), absorption wavelength (λ_{abs}, green), and oscillator strength (f, gold).

Table S7. Ground and lowest vertical excited state properties of the simplified molecules calculated at the LC- ωHPBE (ω=0.2)/def2-TZVPP level of theory with the SMD solvation model in different solvents.

Compound	Solvent	Ground state (DFT)			Vertical excited state (TD-DFT)					
		HOMO [eV]	LUMO [eV]	Dipole moment [D]	State	Orbital contributions	Energy *E* [eV]	λ [nm]	Oscillator strength	Dipole moment [D]
**PA**	DCM	−6.40	−1.33	0.00	S_1	73 -> 74 (0.70515)	2.1484	577.11	0.0867	
**IndFlu**	DCM	−7.00	−1.61	0.00	S_1<br>S_2	65 -> 67 (0.69948)<br>66 -> 67 (0.68767)	2.2868<br>2.4909	542.16<br>497.75	0.0000<br>0.7245	
**SN-PA**	Hex	−5.85	−0.76	2.96	S_1	82 -> 83 (0.69163)	2.2886	541.75	1.0056	3.62
	DCM	−5.87	−0.79	3.94	S_1	82 -> 83 (0.69142)	2.2628	547.92	1.0059	4.70
	MeOH	−5.89	−0.81	4.22	S_1	82 -> 83 (0.68982)	2.2872	542.07	0.9480	5.02
**diSO$_2$-PA**	Hex	−7.38	−2.10	0.00	S_1	98 -> 99 (0.69958)	2.5379	488.53	1.2705	0.00
	DCM	−7.26	−1.98	0.00	S_1	98 -> 99 (0.69962)	2.5165	492.69	1.2916	0.00
	MeOH	−7.31	−2.04	0.00	S_1	98 -> 99 (0.69931)	2.5355	489.00	1.2447	0.00
**diSO$_2$-PA-c**	DCM	−7.10	−2.46	0.00	S_1	150 -> 151 (0.7027)	2.0439	606.61	1.1961	
**PON-PA**	Hex	−6.17	−1.13	7.43	S_1	90 -> 91 (0.68575)	2.0824	595.39	0.6457	9.04
	DCM	−6.12	−1.11	9.34	S_1	90 -> 91 (0.68678)	2.0308	610.53	0.6421	11.05
	MeOH	−6.14	−1.17	10.62	S_1	90 -> 91 (0.68547)	2.0100	616.84	0.5830	12.41
**SO$_2$N-PA**	Hex	−6.34	−1.35	8.75	S_1	90 -> 91 (0.68633)	2.0125	616.08	0.5820	10.07
	DCM	−6.28	−1.33	11.38	S_1	90 -> 91 (0.68785	1.9447	637.54	0.5624	12.29
	MeOH	−6.30	−1.39	13.08	S_1	90 -> 91 (0.68729)	1.9179	646.45	0.5001	13.59
**SO$_2$N-PA-c**	Hex	−6.29	−1.81	8.36	S_1	142 -> 143 (0.6900)	1.7238	719.25	0.5932	9.29
	DCM	−6.31	−1.84	11.82	S_1	142 -> 143 (0.6903)	1.6931	732.29	0.5432	11.48
	MeOH	−6.36	−1.90	13.76	S_1	142 -> 143 (0.6896)	1.6916	732.95	0.4751	12.79
**CON-PA**	Hex	−6.18	−1.49	7.98	S_1	81 -> 82 (0.69052)	1.6932	732.25	0.3906	9.50
	DCM	−6.16	−1.51	11.24	S_1	81 -> 82 (0.69245)	1.6246	763.16	0.3687	11.63
	MeOH	−6.21	−1.63	13.90	S_1	81 -> 82 (0.69337)	1.5739	787.75	0.3105	12.96

Table S8. TD-DFT-calculated absorption ($S_0 \rightarrow S_1$) and emission ($S_1 \rightarrow S_0$) parameters for pentacene analogues, based on ground-state (GS) and S_1-excited-state (ES) optimized geometries, respectively. Method: LC-ωHPBE(ω=0.2)/def2-TZVPP, with the SMD solvation model.

Compound	Solvent	Absorption (GS geometry)				Emission (ES geometry)			
		E [eV]	λ [nm]	*f*	GS[a] dipole moment [D]	*E* [eV]	λ [nm]	*f*	ES[a] dipole moment [D]
**SN-PA**	Hex	2.2886	541.8	1.0056	3.0	1.9345	640.9	0.9722	3.7
	DCM	2.2628	547.9	1.0059	3.9	1.6570	748.2	1.3263	4.5
	MeOH	2.2872	542.1	0.9480	4.2	1.5755	787.0	1.4131	4.7
**diSO$_2$-PA**	Hex	2.5379	488.5	1.2705	0.0	2.0810	595.8	1.2583	0.0
	DCM	2.5165	492.7	1.2916	0.0	1.7610	704.1	1.5452	0.0
	MeOH	2.5355	489.0	1.2447	0.0	1.6559	748.7	1.6053	0.0
**PON-PA**	Hex	2.0824	595.4	0.6457	7.4	1.5435	803.3	0.4553	9.4
	DCM	2.0308	610.5	0.6421	9.3	1.4114	878.4	0.6910	10.9
	MeOH	2.0100	616.8	0.5830	10.6	1.3556	914.6	0.7081	11.9
**SO$_2$N-PA**	Hex	2.0125	616.1	0.5820	8.7	1.4645	846.6	0.3714	10.7
	DCM	1.9447	637.5	0.5624	11.4	1.4007	885.2	0.5763	12.4
	MeOH	1.9179	646.5	0.5001	13.1	1.3762	900.9	0.6093	13.6
**SO$_2$N-PA-c**	Hex	1.7238	719.25	0.5932	8.4	1.3005	953.4	0.4130	9.9
	DCM	1.6931	732.29	0.5432	11.8	1.2563	986.9	0.5823	12.0
	MeOH	1.6916	732.95	0.4751	13.8	1.2574	986.0	0.6122	13.4
**CON-PA**	Hex	1.6932	732.3	0.3906	8.0	1.2311	1007.1	0.2494	9.7
	DCM	1.6246	763.2	0.3687	11.2	1.2073	1027.0	0.3713	11.3
	MeOH	1.5739	787.8	0.3105	13.9	1.1800	1050.7	0.3706	12.6

[a] Dipole moments of the ground- and excited-state optimized geometries (not transition dipole moments).

Table S9. Energies of vertical singlet (S_1) and triplet (T_1) excited states calculated by TD-DFT at the LC-ωHPBE(ω=0.2)/def2-TZVPP level with the SMD solvation model. The data are color-coded from lowest (red) to highest (blue) values to reflect the relative destabilization of the triplet state with increasing solvent polarity.

Compound	Solvent	$E(S_1)$ [eV]	$E(T_1)$ [eV]	$E(T_1)$ / $E(S_1)$
**SN-PA**	Hex	2.2886	0.7085	0.31
	DCM	2.2628	0.7254	0.32
	MeOH	2.2872	0.7320	0.32
**diSO$_2$-PA**	Hex	2.5379	0.6172	0.24
	DCM	2.5165	0.6285	0.25
	MeOH	2.5355	0.6309	0.25
**PON-PA**	Hex	2.0824	0.8057	0.39
	DCM	2.0308	0.8443	0.42
	MeOH	2.0100	0.8502	0.42
**SO$_2$N-PA**	Hex	2.0125	0.8443	0.42
	DCM	1.9447	0.9176	0.47
	MeOH	1.9179	0.9573	0.50
**SO$_2$N-PA-c**	Hex	1.7238	0.4728	0.27
	DCM	1.6931	0.6893	0.41
	MeOH	1.6916	0.7794	0.46
**CON-PA**	Hex	1.6932	0.6622	0.39
	DCM	1.6246	0.7732	0.48
	MeOH	1.5739	0.8274	0.53

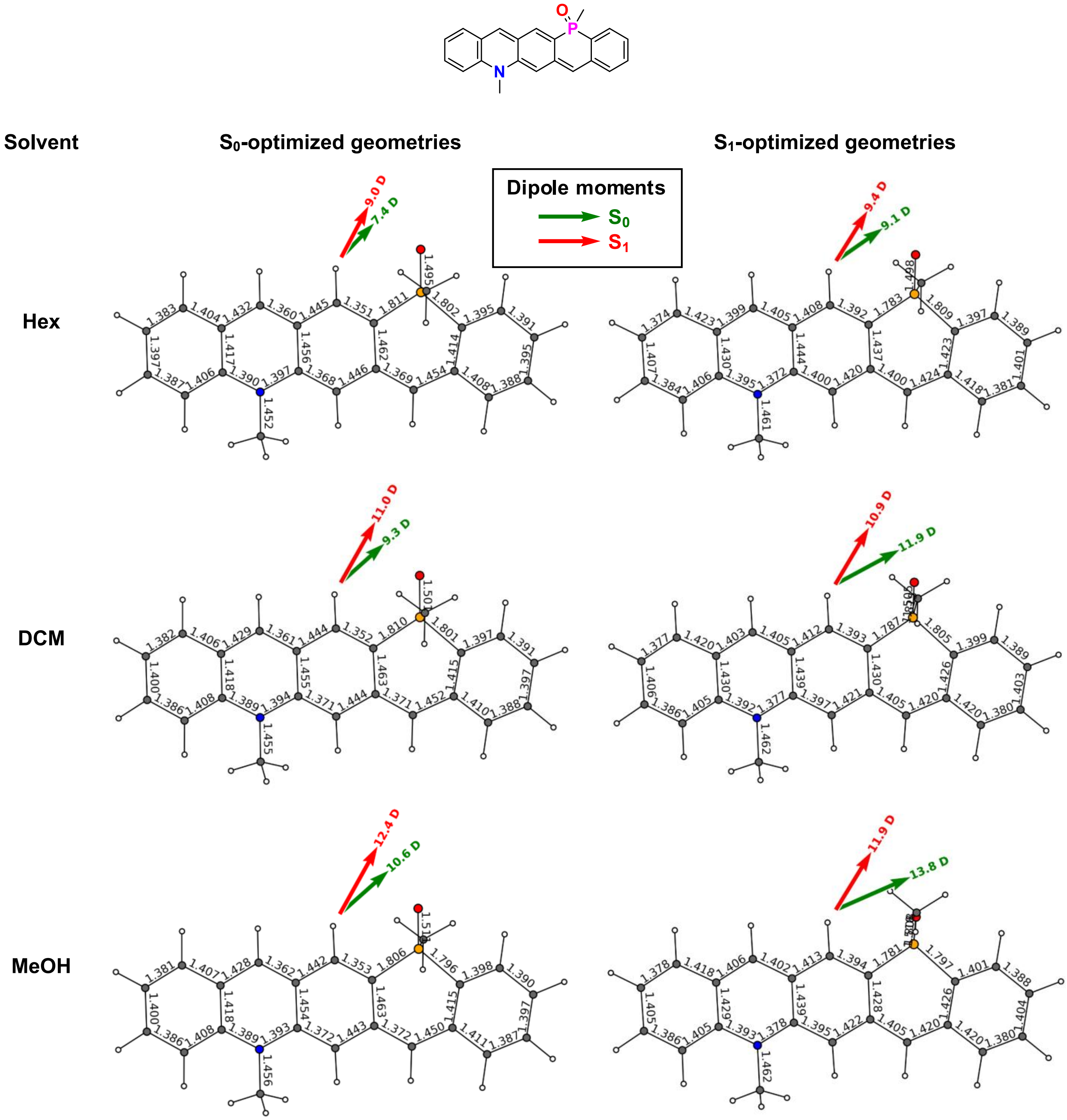

Figure S24. Geometries of simplified **PON-PA** molecules in the S$_0$ and S$_1$ states, optimized in various solvents by DFT and TD-DFT, respectively, at the LC-ωHPBE(ω=0.2)/def2-TZVPP level with the SMD solvation model. The structures are projected onto a 2D plane, with annotated bond lengths (in Å) shown for the π-system. Arrows indicate the orientation and magnitude of the dipole moments for each geometry in the ground (S$_0$, green) and vertically excited (S$_1$, red) states. The arrow lengths are scaled to reflect charge separation, using the convention 1.0 Å per 4.80 D (i.e., 1 e·Å = 4.80 D).

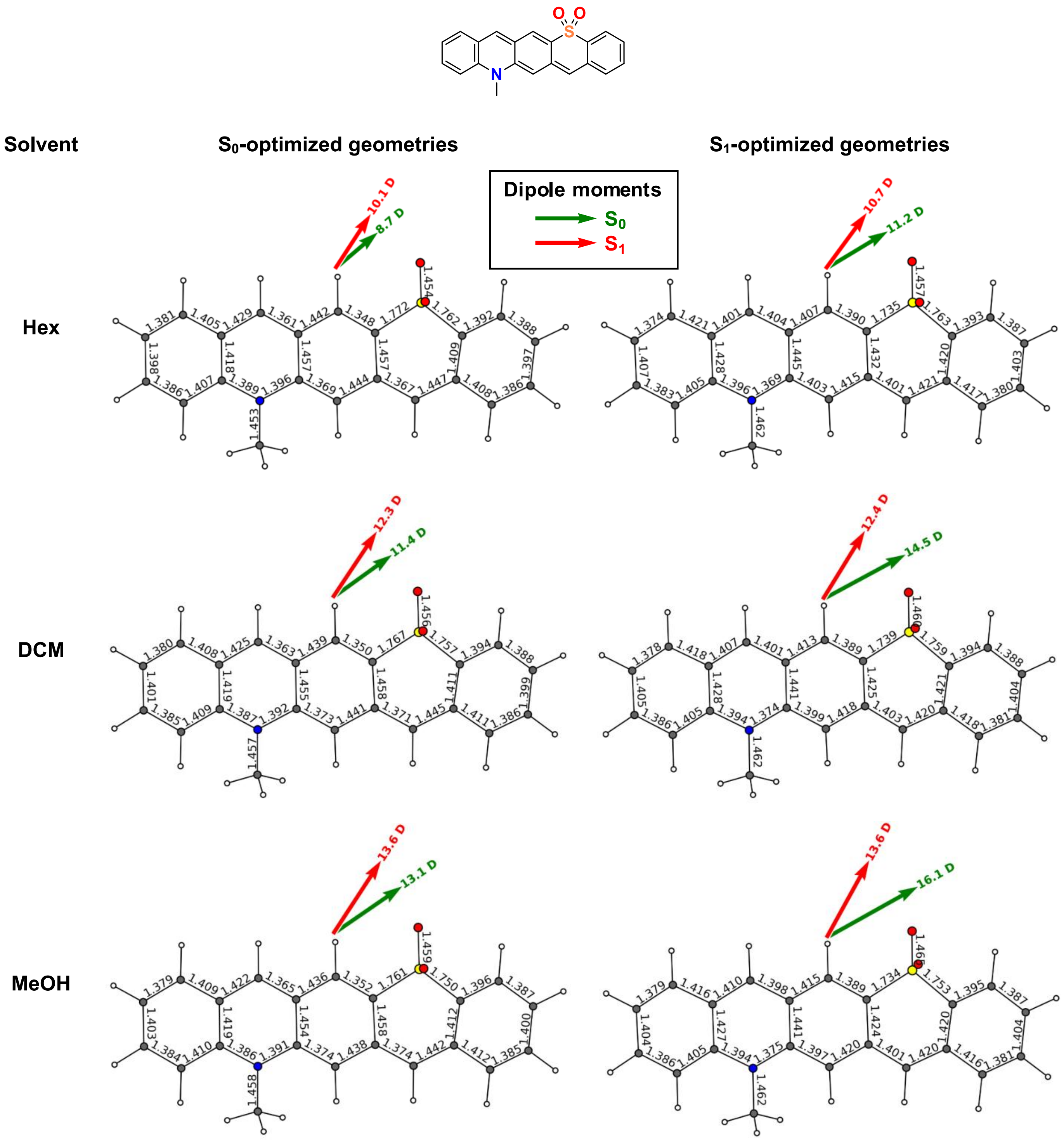

Figure S25. Geometries of simplified **SO2N-PA** molecules in the S_0 and S_1 states, optimized in various solvents by DFT and TD-DFT, respectively, at the LC-ωHPBE(ω=0.2)/def2-TZVPP level with the SMD solvation model. The structures are projected onto a 2D plane, with annotated bond lengths (in Å) shown for the π-system. Arrows indicate the orientation and magnitude of the dipole moments for each geometry in the ground (S_0, green) and vertically excited (S_1, red) states. The arrow lengths are scaled to reflect charge separation, using the convention 1.0 Å per 4.80 D (i.e., 1 e·Å = 4.80 D).

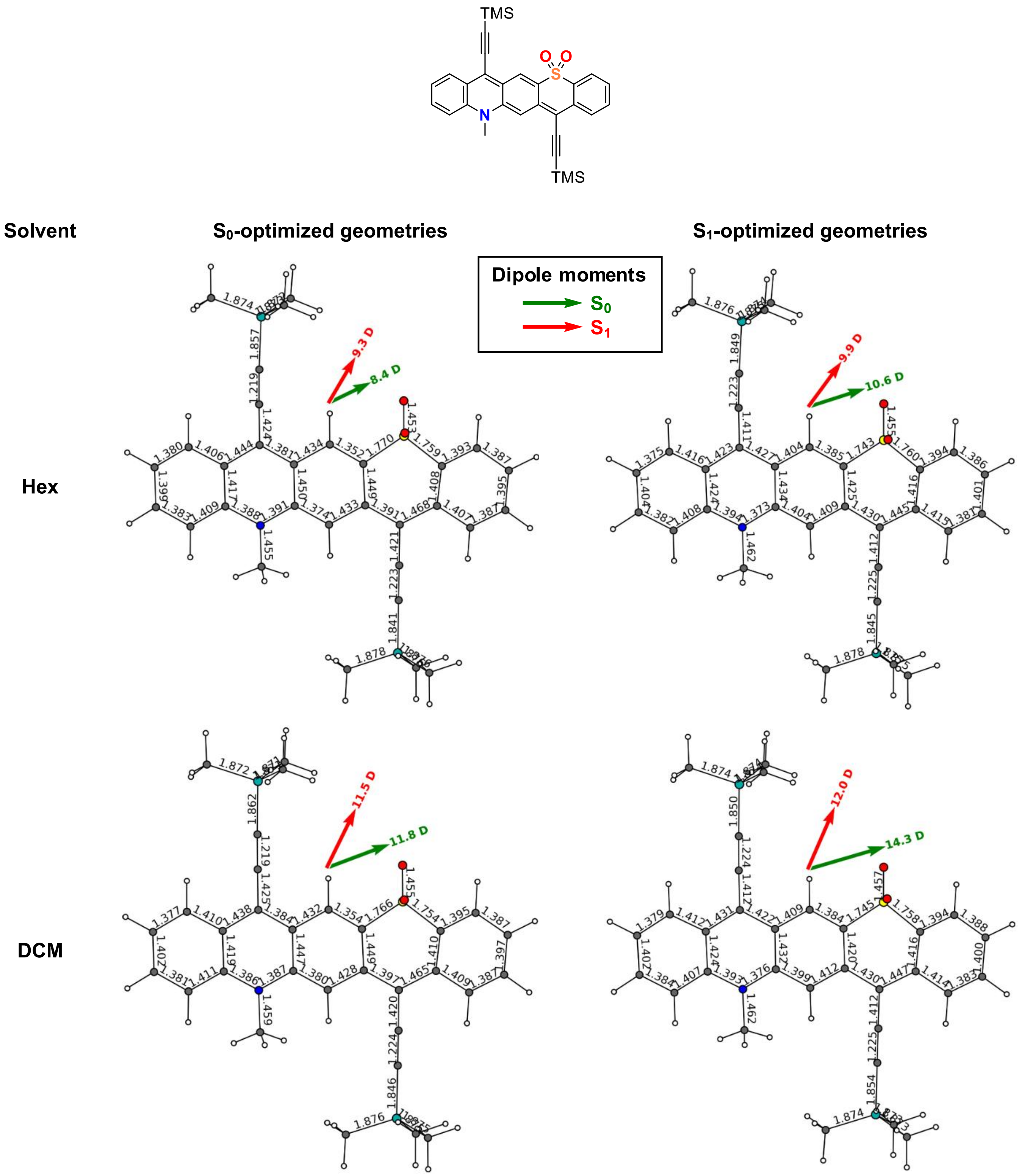

Figure S26. Geometries of simplified **SO2N-PA-c** molecules in the S_0 and S_1 states, optimized in various solvents by DFT and TD-DFT, respectively, at the LC-ωHPBE(ω=0.2)/def2-TZVPP level with the SMD solvation model. The structures are projected onto a 2D plane, with annotated bond lengths (in Å) shown for the π-system. Arrows indicate the orientation and magnitude of the dipole moments for each geometry in the ground (S_0, green) and vertically excited (S_1, red) states. The arrow lengths are scaled to reflect charge separation, using the convention 1.0 Å per 4.80 D (i.e., 1 e·Å = 4.80 D).

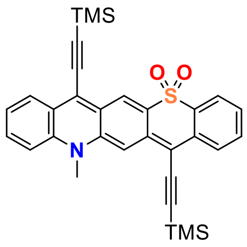

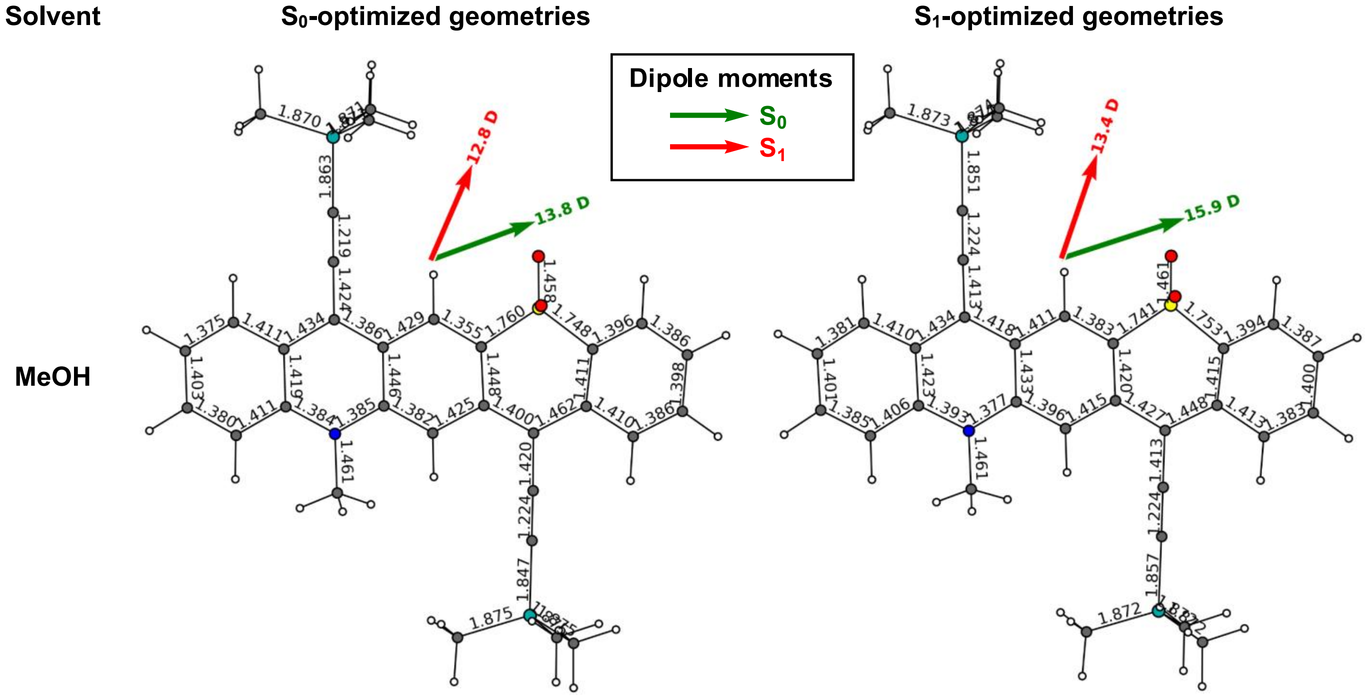

Figure S27. Geometries of simplified **SO2N-PA-c** molecules in the S_0 and S_1 states, optimized in methanol by DFT and TD-DFT, respectively, at the LC-ωHPBE(ω=0.2)/def2-TZVPP level with the SMD solvation model. The structures are projected onto a 2D plane, with annotated bond lengths (in Å) shown for the π-system. Arrows indicate the orientation and magnitude of the dipole moments for each geometry in the ground (S_0, green) and vertically excited (S_1, red) states. The arrow lengths are scaled to reflect charge separation, using the convention 1.0 Å per 4.80 D (i.e., 1 e·Å = 4.80 D).

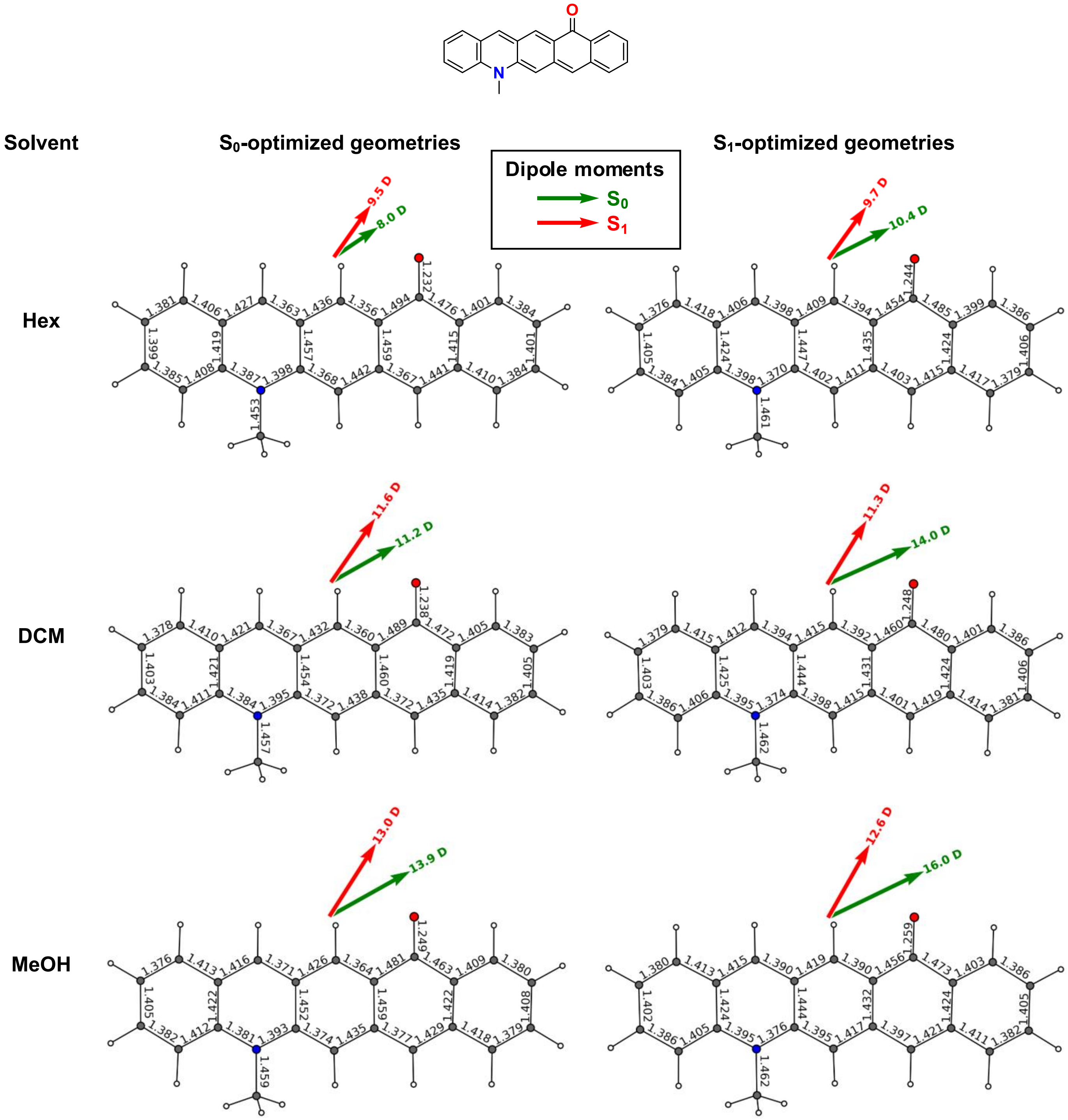

Figure S28. Geometries of simplified **CON-PA** molecules in the S_0 and S_1 states, optimized in various solvents by DFT and TD-DFT, respectively, at the LC-ωHPBE(ω=0.2)/def2-TZVPP level with the SMD solvation model. The structures are projected onto a 2D plane, with annotated bond lengths (in Å) shown for the π-system. Arrows indicate the orientation and magnitude of the dipole moments for each geometry in the ground (S_0, green) and vertically excited (S_1, red) states. The arrow lengths are scaled to reflect charge separation, using the convention 1.0 Å per 4.80 D (i.e., 1 e·Å = 4.80 D).

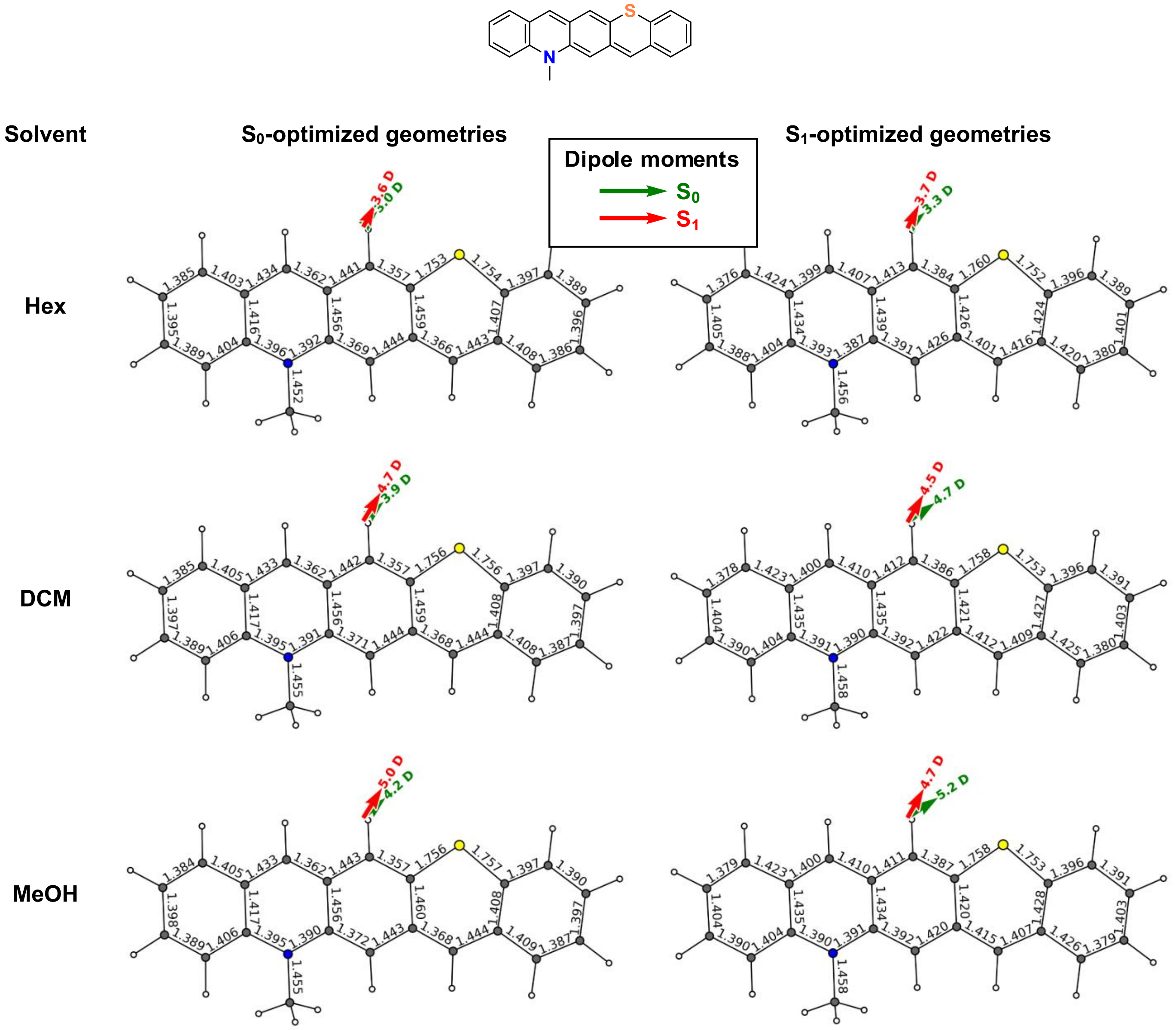

Figure S29. Geometries of simplified **SN-PA** molecules in the S_0 and S_1 states, optimized in various solvents by DFT and TD-DFT, respectively, at the LC-ωHPBE(ω=0.2)/def2-TZVPP level with the SMD solvation model. The structures are projected onto a 2D plane, with annotated bond lengths (in Å) shown for the π-system. Arrows indicate the orientation and magnitude of the dipole moments for each geometry in the ground (S_0, green) and vertically excited (S_1, red) states. The arrow lengths are scaled to reflect charge separation, using the convention 1.0 Å per 4.80 D (i.e., 1 e·Å = 4.80 D).

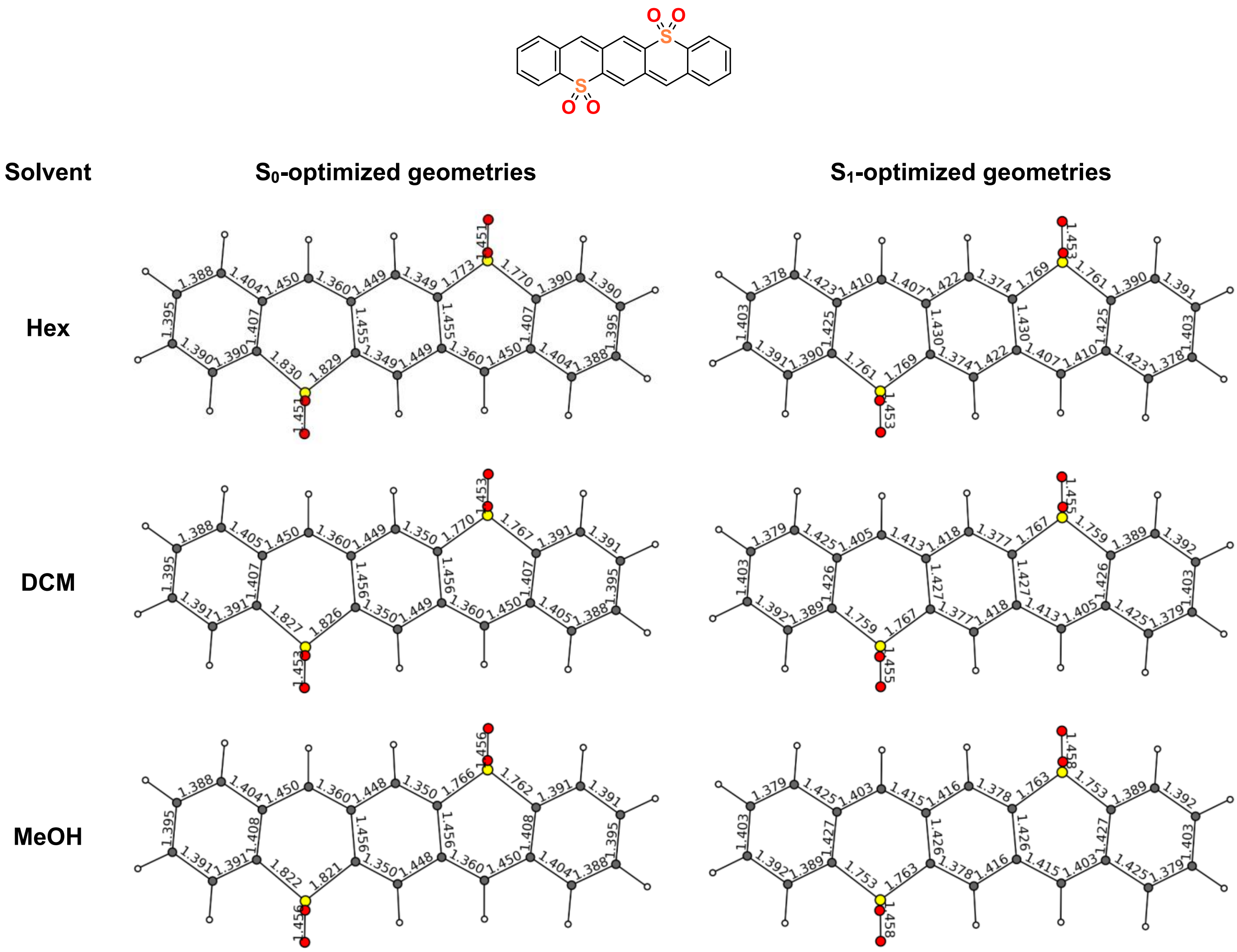

Figure S30. Geometries of simplified **diSO2-PA** molecules in the S_0 and S_1 states, optimized in various solvents by DFT and TD-DFT, respectively, at the LC-ωHPBE(ω=0.2)/def2-TZVPP level with the SMD solvation model. The structures are projected onto a 2D plane, with annotated bond lengths (in Å) shown for the π-system.

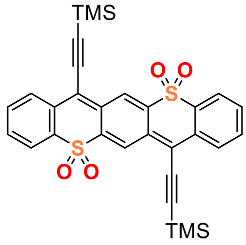

Solvent **S_0-optimized geometries** **S_1-optimized geometries**

DCM

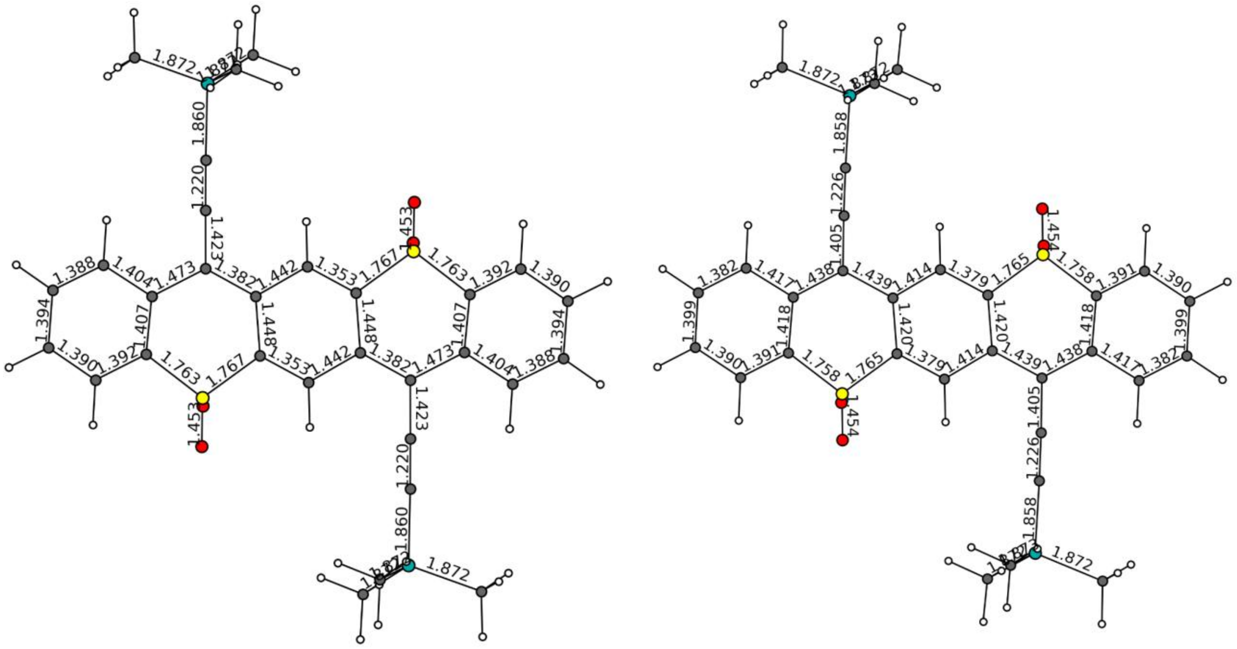

Figure S31. Geometries of simplified **diSO-PA-c** molecules in the S_0 and S_1 states, optimized in various solvents by DFT and TD-DFT, respectively, at the LC-ωHPBE(ω=0.2)/def2-TZVPP level with the SMD solvation model. The structures are projected onto a 2D plane, with annotated bond lengths (in Å) shown for the π-system.

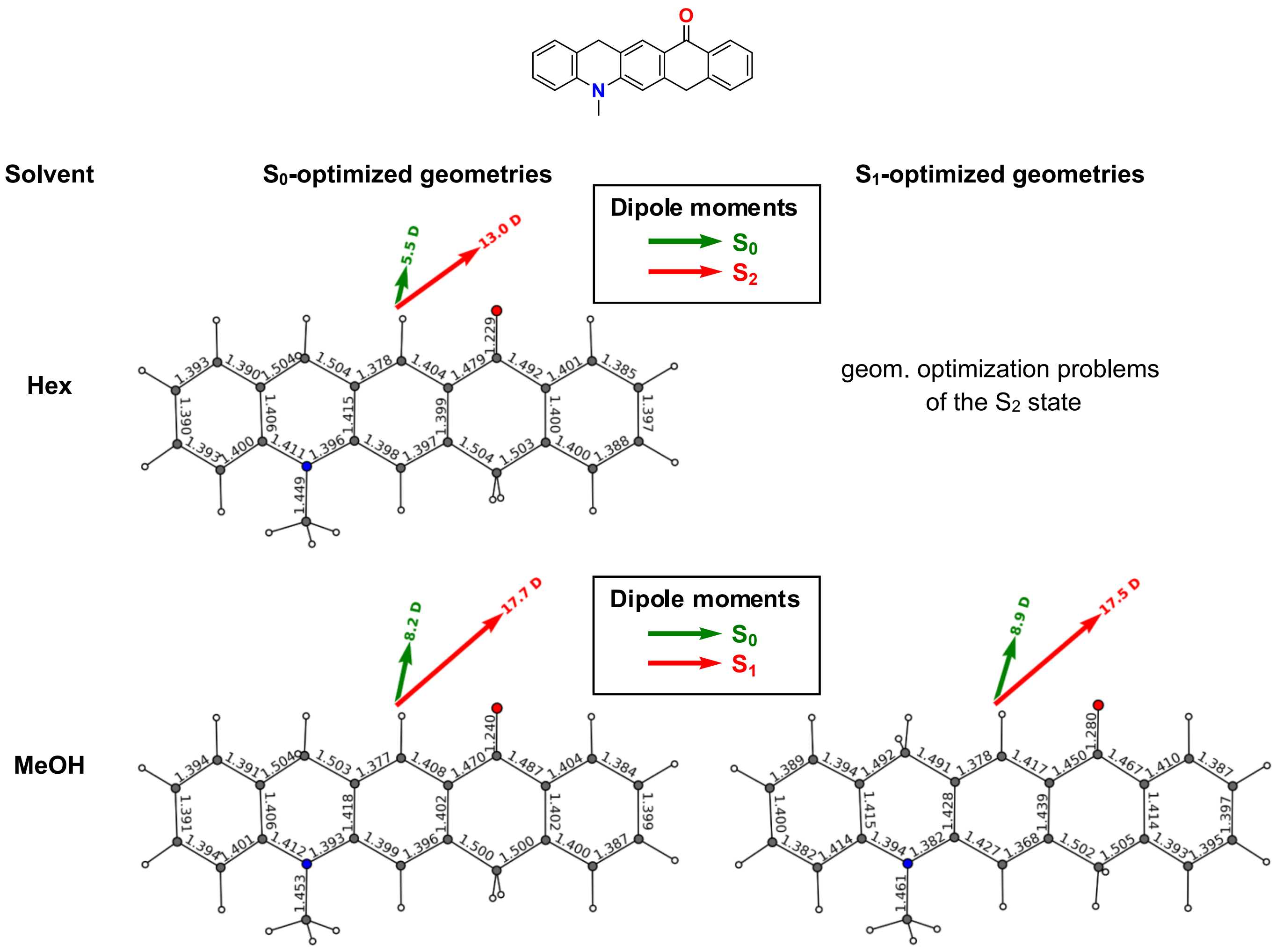

Figure S32. Geometries of simplified **CON-PA-H$_2$** molecules in the S_0 and S_1 states, optimized in various solvents by DFT and TD-DFT, respectively, at the LC-ωHPBE(ω=0.2)/def2-TZVPP level with the SMD solvation model. The structures are projected onto a 2D plane, with annotated bond lengths (in Å) shown for the π-system. Arrows indicate the orientation and magnitude of the dipole moments for each geometry in the ground (S_0, green) and vertically excited (S_2 in *n*-hexane, S_1 in methanol, red) states. In *n*-hexane S_1 state corresponds to n-π* excitation, whereas S_2 state to π-π* (HOMO-LUMO). The arrow lengths are scaled to reflect charge separation, using the convention 1.0 Å per 4.80 D (i.e., 1 e·Å = 4.80 D).

Compound **top view** **side view**

PON-PA

SO₂N-PA

SO₂N-PA-c

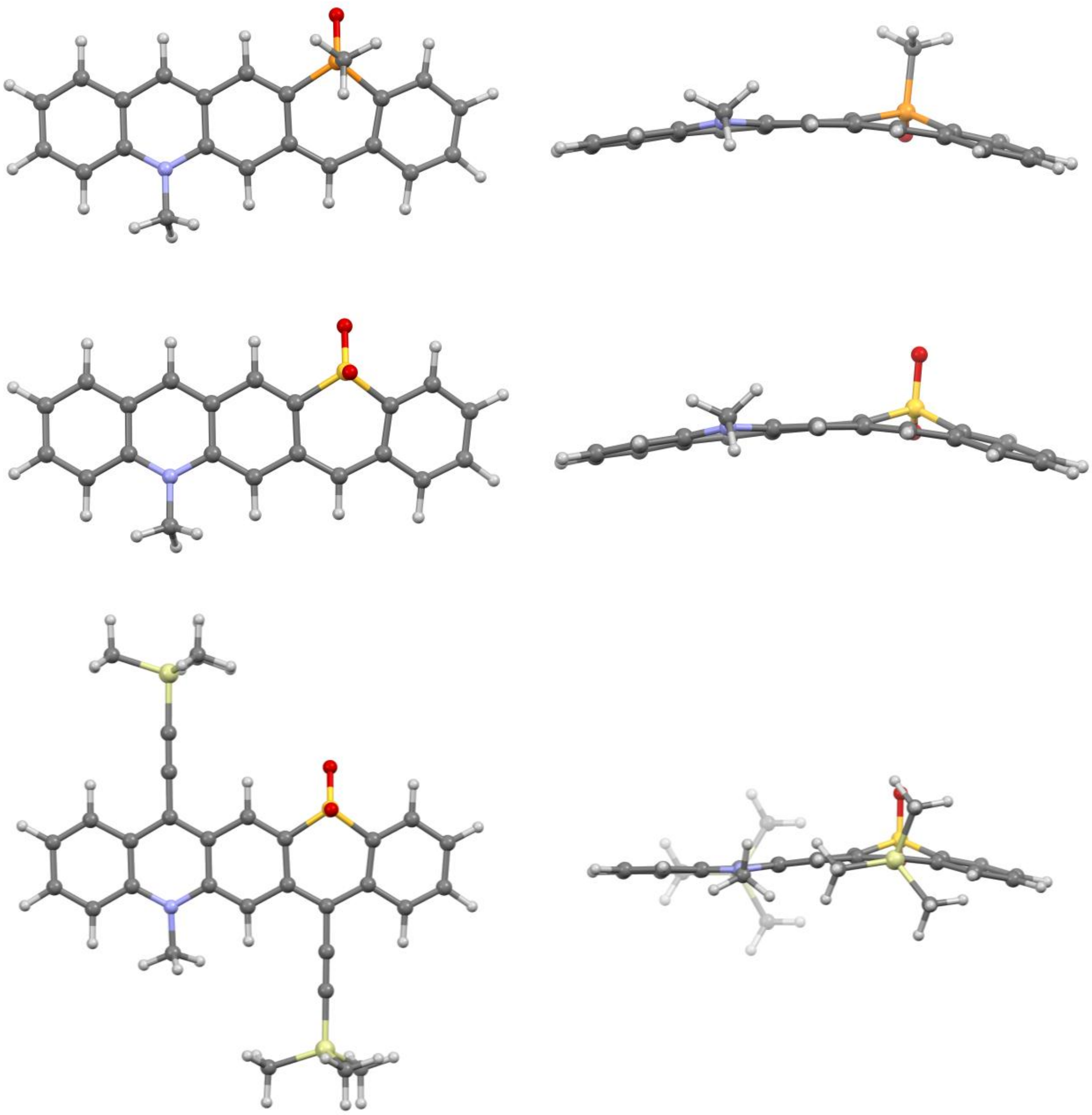

Figure S33. Top and side views of 3D models of the ground-state geometries of simplified DA-pentacene analogues **PON-PA**, **SO_2N-PA**, and **SO_2N-PA-c**, optimized in dichloromethane at the LC-ωHPBE(ω=0.2)/def2-TZVPP level with the SMD solvation model.

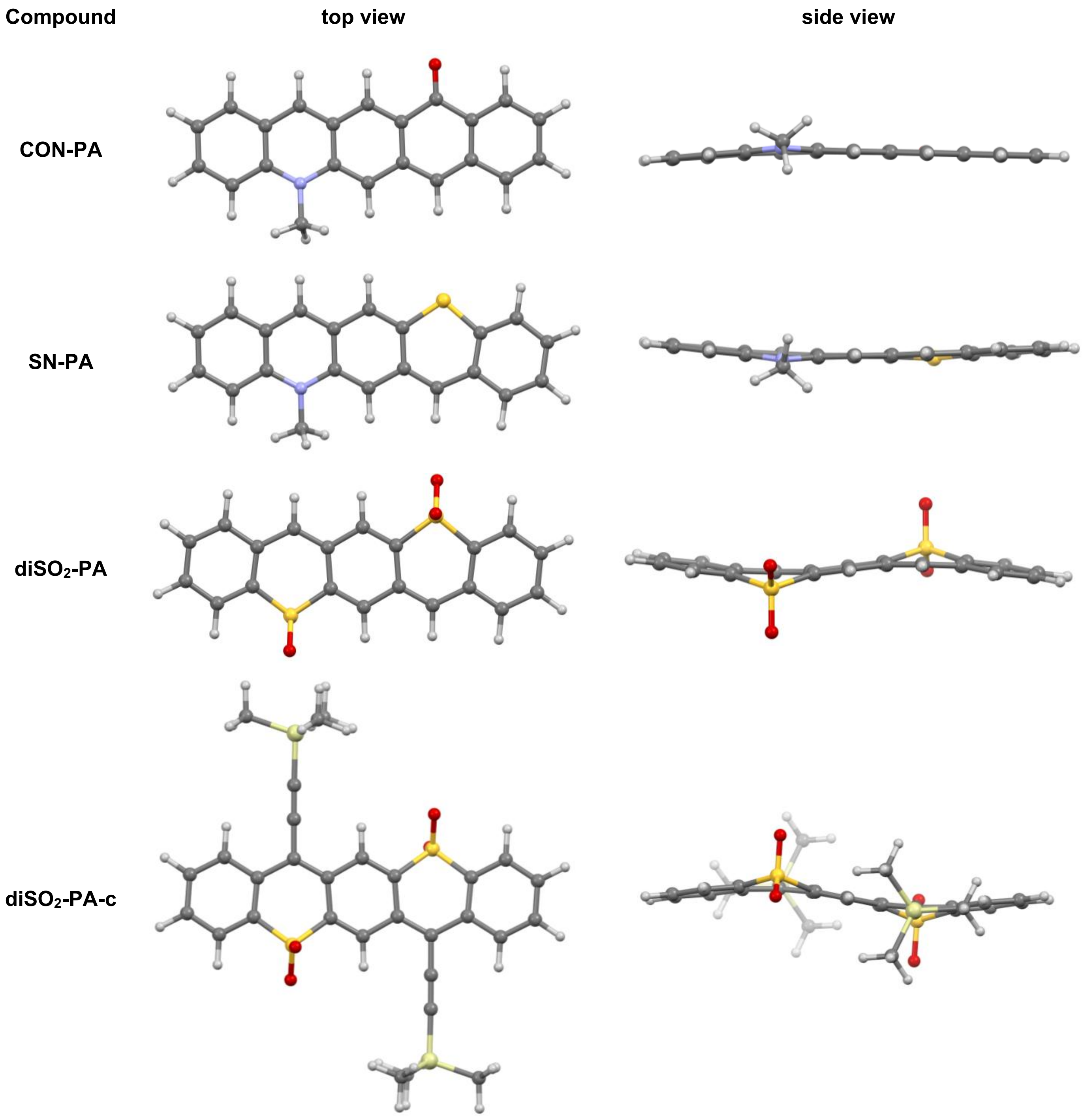

Figure S34. Top and side views of 3D models of the ground-state geometries of simplified DA-pentacene analogues **CON-PA**, **SN-PA**, **diSO$_2$-PA** and **diSO$_2$-PA-c** optimized in dichloromethane at the LC-ωHPBE(ω=0.2)/def2-TZVPP level with the SMD solvation model.

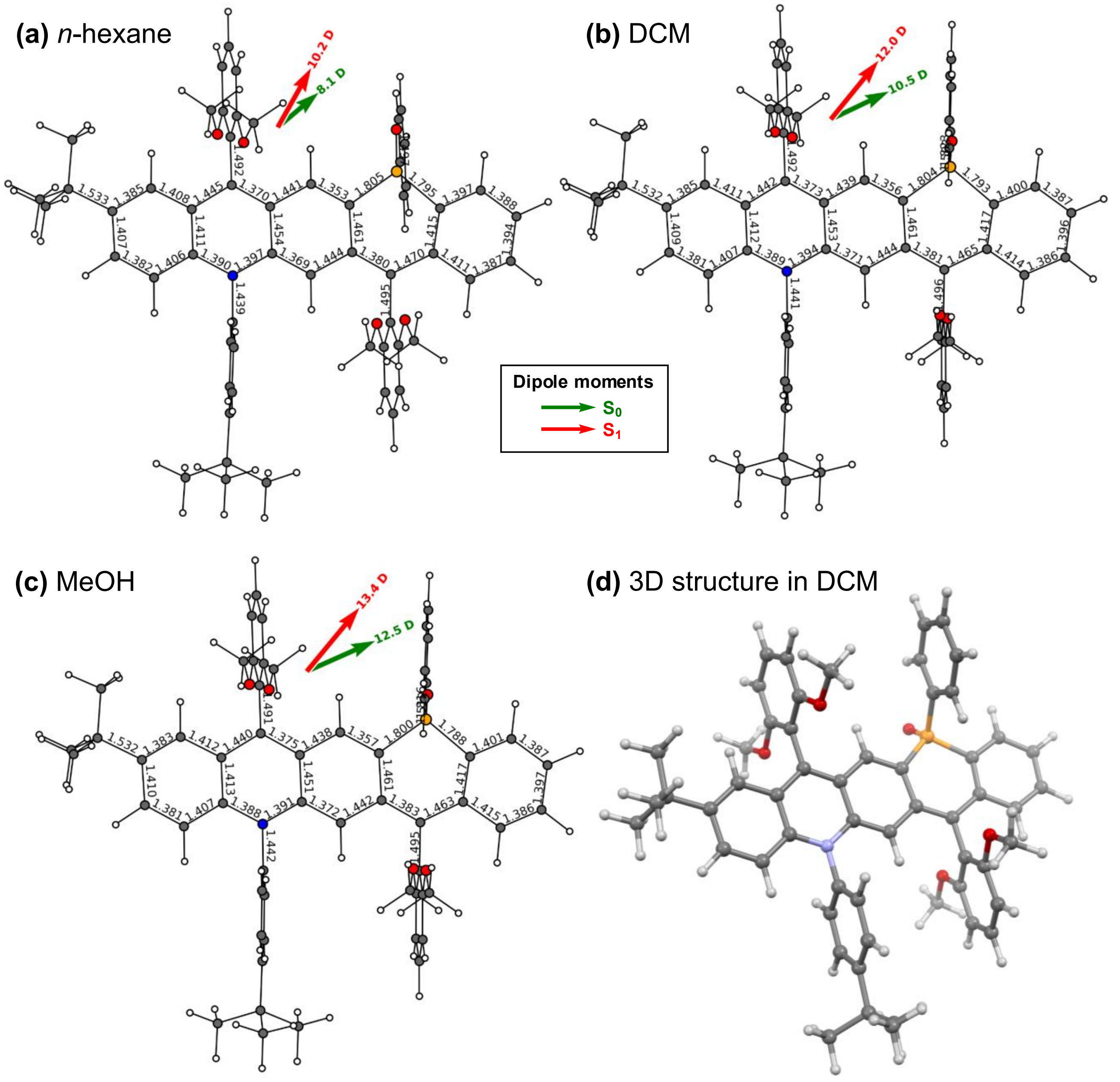

Figure S35. Ground state geometries of **PON-PA** molecules optimized by DFT at the LC-ωHPBE(ω=0.2)/def2-TZVPP level with the SMD solvation model in *n*-hexane (a), dichloromethane (b,d), and methanol (c). In panels (a-c), the structures are projected onto a 2D plane, with annotated bond lengths (in Å) shown for the π-system. Arrows indicate the orientation and magnitude of the dipole moments for each geometry in the ground (S_0, green) and vertically excited (S_1, red) states. The arrow lengths are scaled to reflect charge separation, using the convention 1.0 Å per 4.80 D (i.e., 1 e·Å = 4.80 D). Panel (d) shows a 3D model of the geometry optimized in dichloromethane.

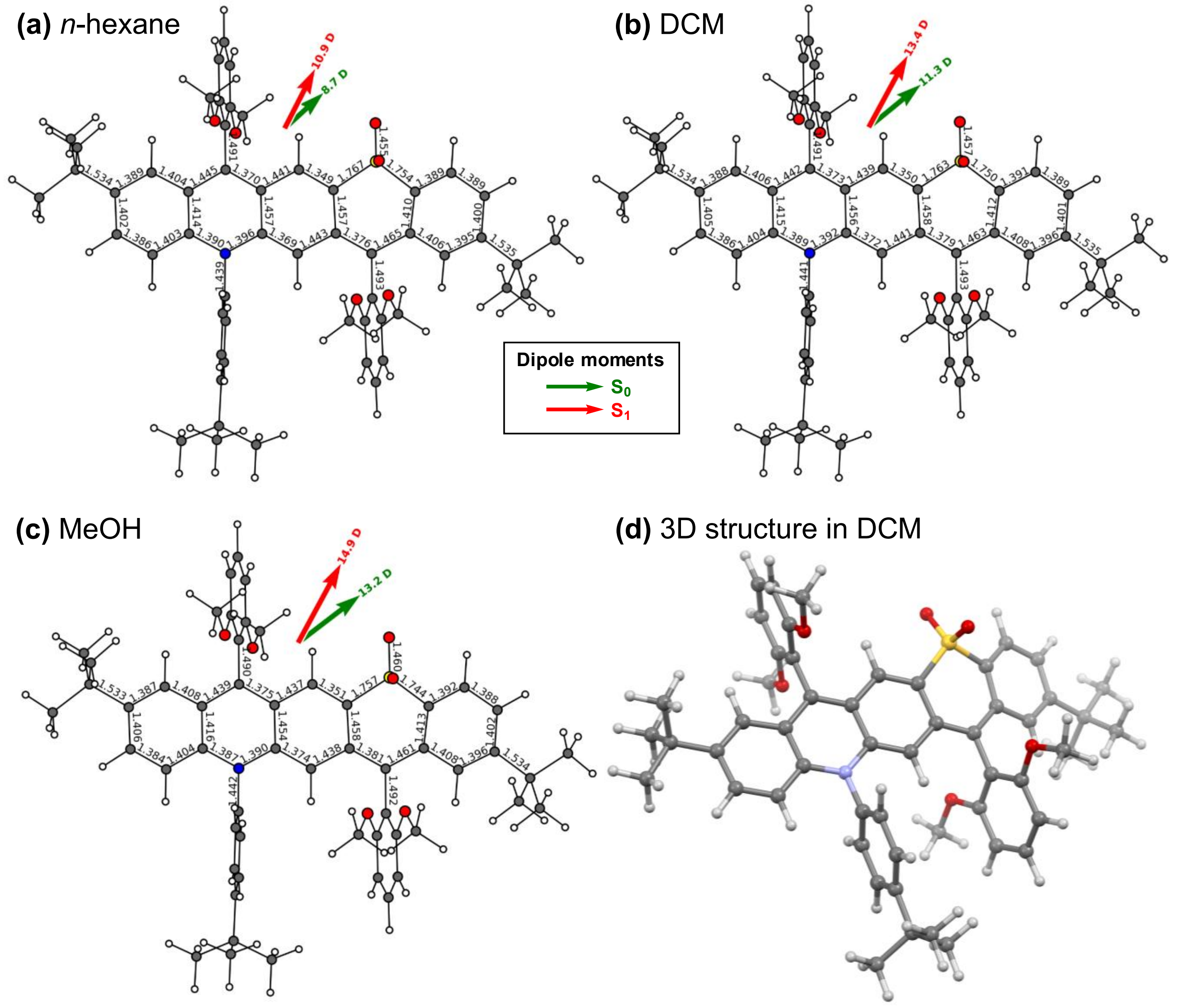

Figure S36. Ground state geometries of **SO_2N-PA-a** molecules optimized by DFT at the LC-ωHPBE(ω=0.2)/def2-TZVPP level with the SMD solvation model in *n*-hexane (a), dichloromethane (b,d), and methanol (c). In panels (a-c), the structures are projected onto a 2D plane, with annotated bond lengths (in Å) shown for the π-system. Arrows indicate the orientation and magnitude of the dipole moments for each geometry in the ground (S_0, green) and vertically excited (S_1, red) states. The arrow lengths are scaled to reflect charge separation, using the convention 1.0 Å per 4.80 D (i.e., 1 e·Å = 4.80 D). Panel (d) shows a 3D model of the geometry optimized in dichloromethane.

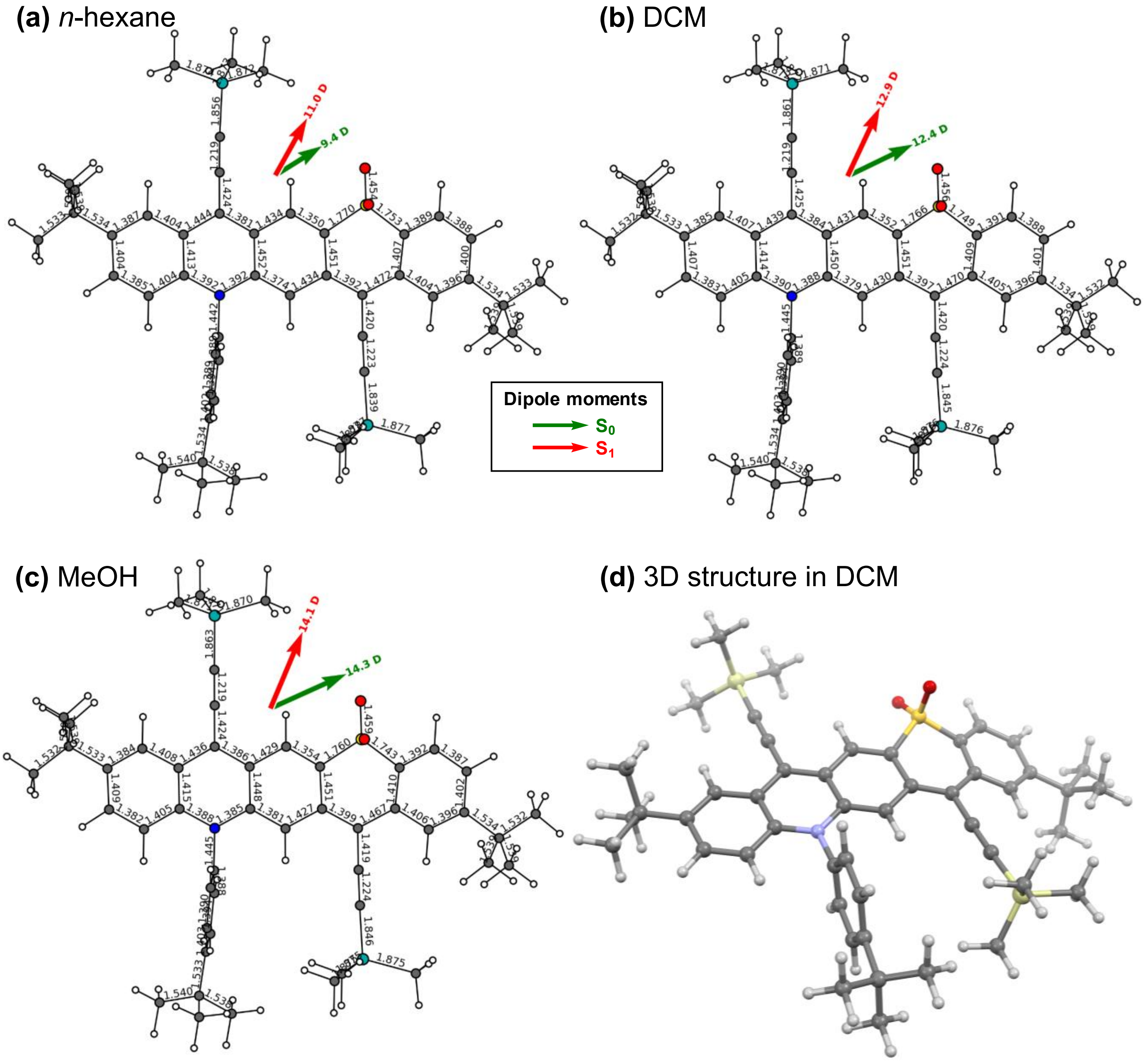

Figure S37. Ground state geometries of **SO_2N-PA-c** molecules optimized by DFT at the LC-ωHPBE(ω=0.2)/def2-TZVPP level with the SMD solvation model in *n*-hexane (a), dichloromethane (b,d), and methanol (c). In panels (a-c), the structures are projected onto a 2D plane, with annotated bond lengths (in Å) shown for the π-system. Arrows indicate the orientation and magnitude of the dipole moments for each geometry in the ground (S_0, green) and vertically excited (S_1, red) states. The arrow lengths are scaled to reflect charge separation, using the convention 1.0 Å per 4.80 D (i.e., 1 e·Å = 4.80 D). Panel (d) shows a 3D model of the geometry optimized in dichloromethane.

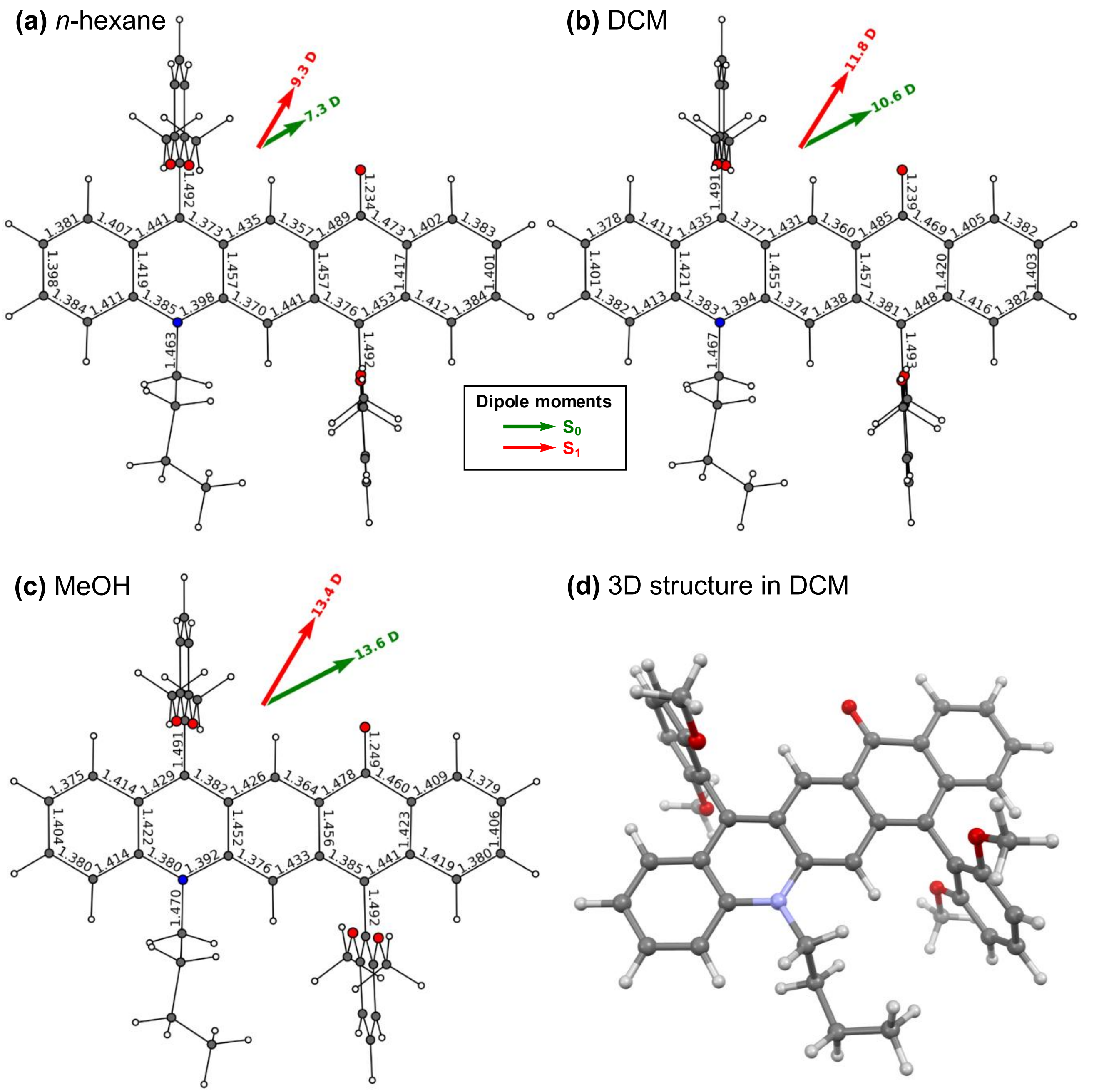

Figure S38. Ground state geometries of **CON-PA** molecules optimized by DFT at the LC-ωHPBE(ω=0.2)/def2-TZVPP level with the SMD solvation model in *n*-hexane (a), dichloromethane (b,d), and methanol (c). In panels (a-c), the structures are projected onto a 2D plane, with annotated bond lengths (in Å) shown for the π-system. Arrows indicate the orientation and magnitude of the dipole moments for each geometry in the ground (S_0, green) and vertically excited (S_1, red) states. The arrow lengths are scaled to reflect charge separation, using the convention 1.0 Å per 4.80 D (i.e., 1 e·Å = 4.80 D). Panel (d) shows a 3D model of the geometry optimized in dichloromethane.

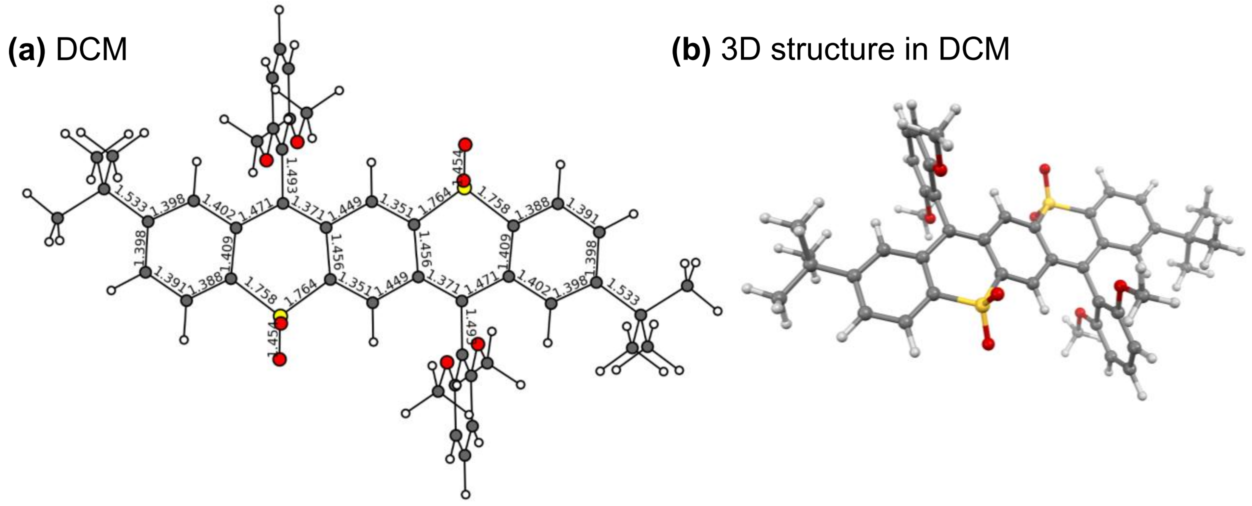

Figure S39. Ground state geometries of a **diSO$_2$-PA-a** molecule optimized by DFT at the LC-ωHPBE(ω=0.2)/def2-TZVPP level with the SMD solvation model in dichloromethane. In panel (a), the structure is projected onto a 2D plane, with annotated bond lengths (in Å) shown for the π-system. Panel (b) shows a 3D model of the geometry optimized in dichloromethane.

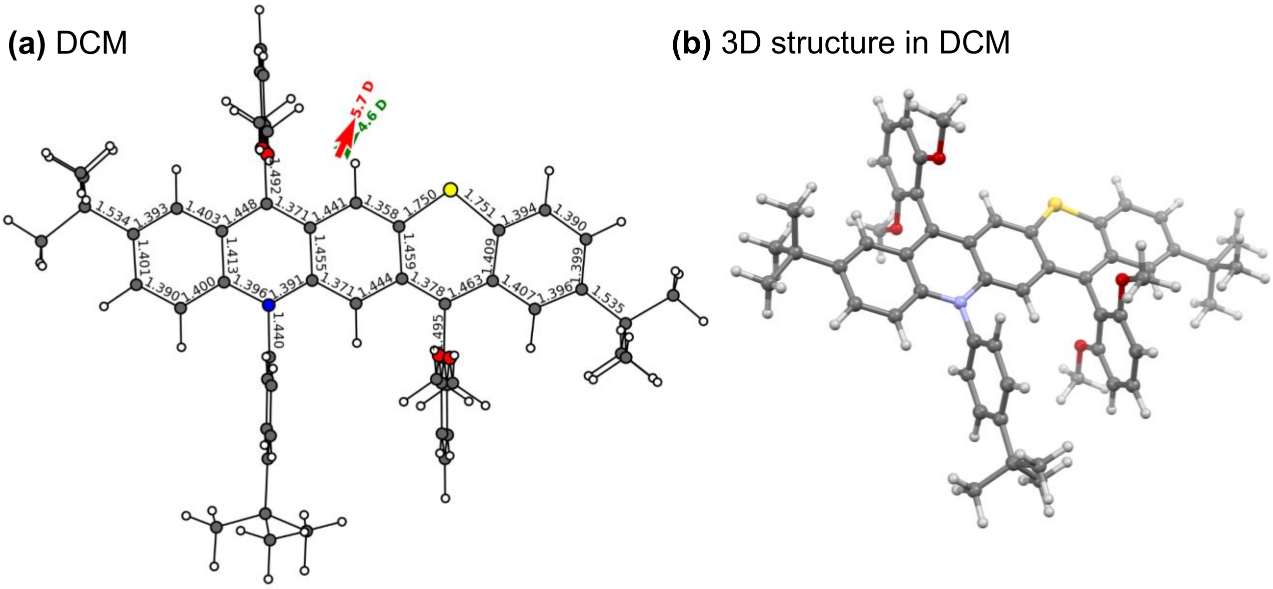

Figure S40. Ground state geometries of a **SN-PA-a** molecule optimized by DFT at the LC-ωHPBE(ω=0.2)/def2-TZVPP level with the SMD solvation model in dichloromethane. In panel (a), the structure is projected onto a 2D plane, with annotated bond lengths (in Å) shown for the π-system. Panel (b) shows a 3D model of the geometry optimized in dichloromethane.

5. Aromaticity

Nucleus-independent chemical shifts (NICS(h)$_{ZZ}$, h = 1.0 Å and 1.7 Å)[31,32] were calculated using gauge-independent atomic orbital (GIAO)[33] method at the LC-ωHPBE(ω=0.2)/def2-TZVPP level of theory for the optimized geometries of the simplified molecules (see section 4).
The route sections for the NICS map calculations in Gaussian is presented below (for DCM as solvent):

```
#p nmr=giao lc-whpbe/def2tzvpp scrf=(smd,solvent=dichloromethane) iop(3/107=0200000000) iop(3/108=0200000000) geom=connectivity
```

For the generation of 2D NICS(1)$_{ZZ}$ maps, a set of in-house Python scripts was employed to prepare Gaussian input files (*nics2d.py*) and visualize the results (*nics_map.py*, *nics_solv.py*).
The *nics2d.py* script, based on a template input file, generates Gaussian inputs for all molecular coordinate files (*.xyz) in a directory. For each geometry, it identifies the longest fused polycyclic system and aligns it with the xy plane and along the x axis. To account for non-planar π-systems (such as **PON-PA** and **SO_2N-PA**), the script determines ring centroids and vectors normal to each ring plane, which are then used to position dummy atoms (Bq) in a grid that follows the molecular curvature. Taking the height *h* and interpoint distance *d* (both in Å) as arguments, the program constructs pairs of dummy atoms arranged in two hexagonal grids, located *h* above and *h* below the polycyclic system, with average interpoint spacing of *d* (see Figure S41 for **PON-PA**, *h* = 1.7 Å and *d* = 0.2 Å).
After running the Gaussian calculations, the *nics_map.py* script processes the resulting *.log files to generate 2D NICS maps. Magnetic shielding tensors are corrected for ring tilts, and NICS(h)$_{ZZ}$ values from each pair of points (above and below) are averaged and mapped onto a 2D plane using the Matplotlib library (matplotlib.pyplot) and the RGB color scale:

```
[(0.12, 0.03, 0.39), (0.03, 0.04, 0.64), (0.05, 0.11, 0.98), (0.19, 0.68, 1), (0.73, 0.92, 1), (1, 1, 1), (1, 0.92, 0.73), (1, 0.68, 0.19), (0.98, 0.11, 0.05), (0.64, 0.04, 0.03), (0.39, 0.03, 0.12)]
```

The *nics_solv.py* script identifies pairs of calculations for the same compound performed in two different solvents and generates differential 2D NICS maps. Results are processed in the same manner as in nics_map.py, but the values are reported as:

ΔNICS(h)$_{ZZ}$ = NICS(h)$_{ZZ}^{polar}$ - NICS(h)$_{ZZ}^{non\text{-}polar}$

The above Python scripts for preparing Gaussian input files and visualizing NICS results are available at: https://github.com/mrkgrb/nics-vis
Ring-current computations were carried out at the LC-ωHPBE(ω=0.2)/def2-TZVPP level of theory on fully optimized molecular structures in the relevant solvent environments. All analyses were performed using the AIMAll suite of programs (version 19.10.12)[34] on formatted checkpoint (FCHK) files generated from GIAO-NMR single-point calculations with Gaussian.
The accuracy of the QTAIM-based magnetic response calculations was verified by two criteria: (1) ensuring that the integrated Laplacian of the electron density within each atomic basin was smaller than 10^{-4}, and (2)

maintaining the difference between the total QTAIM energy and the corresponding DFT energy below 0.1 kcal/mol.

The magnetically induced current density (MICD) associated with the ring current was evaluated as the flux of the induced current passing through the zero-flux surface shared between neighboring atoms (i.e., the interatomic surface), with the external magnetic field applied perpendicular to the ring plane. These interatomic surfaces are uniquely defined within the QTAIM framework and therefore constitute an unambiguous, non-arbitrary choice that ensures fully repeatable and reproducible MICD values. All MICD data were generated automatically by AIMAll.

Harmonic oscillator model of aromaticity (HOMA)[35,36] values were calculated using *py.Aroma* software.[37]

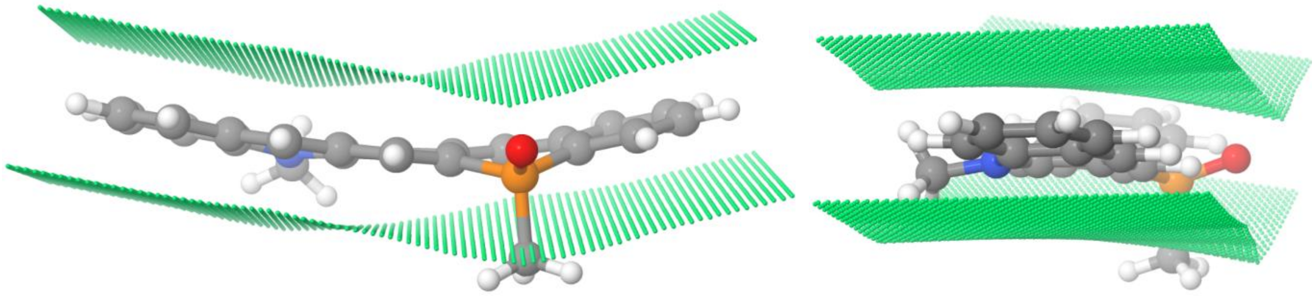

Figure S41. Two projections of the **PON-PA** molecule showing a network of dummy atoms (Bq, green) placed 1.7 Å above and 1.7 Å below the curved π-system, with interpoint distances of approximately 0.2 Å. The positions of the dummy atoms were generated using the *nics2d.py* script.

Table S10. NICS(1)$_{ZZ}$ values at ring centers calculated for simplified pentacene analogues at the LC-ωHPBE(ω=0.2)/def2-TZVPP level with the SMD solvation model in various solvents. The data are color-coded from positive (red for NICS(1)$_{ZZ}$ ≥ +12 ppm) to negative (blue for NICS(1)$_{ZZ}$ ≤ −12 ppm) values to highlight discrete changes in NICS values at intermediate ring current strengths.

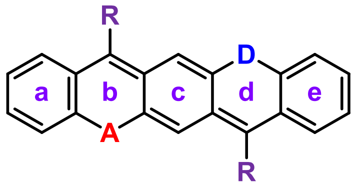

Compound	Acceptor A	Donor D	R	Solvent	Ring's NICS(1)$_{ZZ}$ [ppm] a	b	c	d	e	Sum of NICS	μ (D)
**diN-PA**	N-CH_3	N-CH_3	H	Hex	−20.1	6.8	4.9	6.8	−20.1	−21.7	0.0
				DCM	−19.9	6.9	5.1	6.9	−19.9	−20.8	0.0
				MeOH	−19.8	7.0	5.2	7.0	−19.8	−20.6	0.0
**SN-PA**	S	N-CH_3	H	Hex	−19.7	11.1	3.1	6.0	−20.6	−20.1	3.0
				DCM	−19.4	12.0	3.2	5.3	−20.7	−19.6	3.9
				MeOH	−19.3	12.2	3.2	5.1	−20.8	−19.7	4.2
**diSO_2-PA**	SO_2	SO_2	H	Hex	−24.6	6.1	1.4	6.1	−24.6	−35.5	0.0
				DCM	−24.6	6.0	1.6	6.0	−24.6	−35.7	0.0
				MeOH	−24.6	5.8	1.7	5.8	−24.6	−35.8	0.0
**PON-PA**	PO-CH_3	N-CH_3	H	Hex	−23.9	5.1	−1.3	1.5	−22.3	−41.0	7.4
				DCM	−23.5	5.1	−2.0	0.0	−22.6	−43.0	9.3
				MeOH	−23.2	5.1	−2.3	−0.5	−22.7	−43.6	10.6
**SO_2N-PA**	SO_2	N-CH_3	H	Hex	−24.2	3.4	−2.7	−0.2	−22.8	−46.4	8.7
				DCM	−23.9	2.4	−4.4	−2.5	−23.2	−51.6	11.4
				MeOH	−23.7	1.4	−5.6	−3.8	−23.4	−55.1	13.1
**SO_2N-PA-c**	SO_2	N-CH_3	–C≡C–TMS	Hex	−24.2	5.4	−5.0	−1.1	−22.9	−47.9	8.4
				DCM	−24.0	4.5	−7.4	−4.5	−23.5	−54.8	11.8
				MeOH	−23.8	3.8	−8.6	−6.1	−23.6	−58.3	13.8
**CON-PA**	C=O	N-CH_3	H	Hex	−24.5	−2.0	−5.6	−0.7	−22.6	−55.4	8.0
				DCM	−24.3	−5.0	−8.9	−4.2	−23.2	−65.7	11.2
				MeOH	−24.2	−9.0	−12.4	−7.1	−23.6	−76.3	13.9

Table S11. NICS(1.7)$_{zz}$ values at ring centers calculated for simplified pentacene analogues at the LC-ωHPBE(ω=0.2)/def2-TZVPP level with the SMD solvation model in different solvents. The data are color-coded from positive (red for NICS(1.7)$_{zz}$ ≥ +12 ppm) to negative (blue for NICS(1.7)$_{zz}$ ≤ −12 ppm) values to highlight discrete changes in NICS values at intermediate ring current strengths.

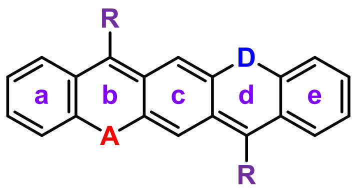

Compound	Acceptor A	Donor D	R	Solvent	Ring's NICS(1.7)$_{zz}$ [ppm] a	b	c	d	e	Sum of NICS	μ (D)
**diN-PA**	N-CH3	N-CH3	H	Hex	−16.0	−1.8	−1.3	−1.8	−16.0	−36.9	0.0
				DCM	−16.0	−1.6	−1.2	−1.6	−16.0	−36.4	0.0
				MeOH	−15.9	−1.6	−1.2	−1.6	−15.9	−36.2	0.0
**SN-PA**	S	N-CH3	H	Hex	−15.7	1.0	−2.0	−2.4	−16.4	−35.4	3.0
				DCM	−15.5	1.6	−1.9	−2.7	−16.5	−35.1	3.9
				MeOH	−15.5	1.7	−1.9	−2.9	−16.6	−35.2	4.2
**diSO2-PA**	SO2	SO2	H	Hex	−18.5	−2.1	−4.5	−2.1	−18.5	−45.7	0.0
				DCM	−18.6	−2.2	−4.4	−2.2	−18.6	−45.9	0.0
				MeOH	−18.6	−2.3	−4.3	−2.3	−18.6	−46.0	0.0
**PON-PA**	PO-CH3	N-CH3	H	Hex	−18.2	−1.8	−5.4	−5.3	−17.5	−48.1	7.4
				DCM	−18.0	−1.7	−5.8	−6.1	−17.7	−49.3	9.3
				MeOH	−17.9	−1.8	−6.0	−6.4	−17.8	−49.8	10.6
**SO2N-PA**	SO2	N-CH3	H	Hex	−18.3	−3.1	−6.2	−6.3	−17.8	−51.7	8.7
				DCM	−18.2	−3.7	−7.2	−7.6	−18.1	−54.8	11.4
				MeOH	−18.1	−4.2	−8.0	−8.4	−18.3	−56.9	13.1
**SO2N-PA-c**	SO2	N-CH3	−C≡C−TMS	Hex	−18.2	−2.2	−7.7	−6.9	−17.9	−52.7	8.4
				DCM	−18.1	−2.6	−9.1	−8.8	−18.3	−56.8	11.8
				MeOH	−18.0	−3.0	−9.8	−9.7	−18.4	−58.9	13.8
**CON-PA**	C=O	N-CH3	H	Hex	−19.0	−7.3	−8.5	−6.7	−17.7	−59.2	8.0
				DCM	−19.0	−8.9	−10.5	−8.8	−18.2	−65.4	11.2
				MeOH	−19.1	−11.1	−12.6	−10.5	−18.5	−71.8	13.9

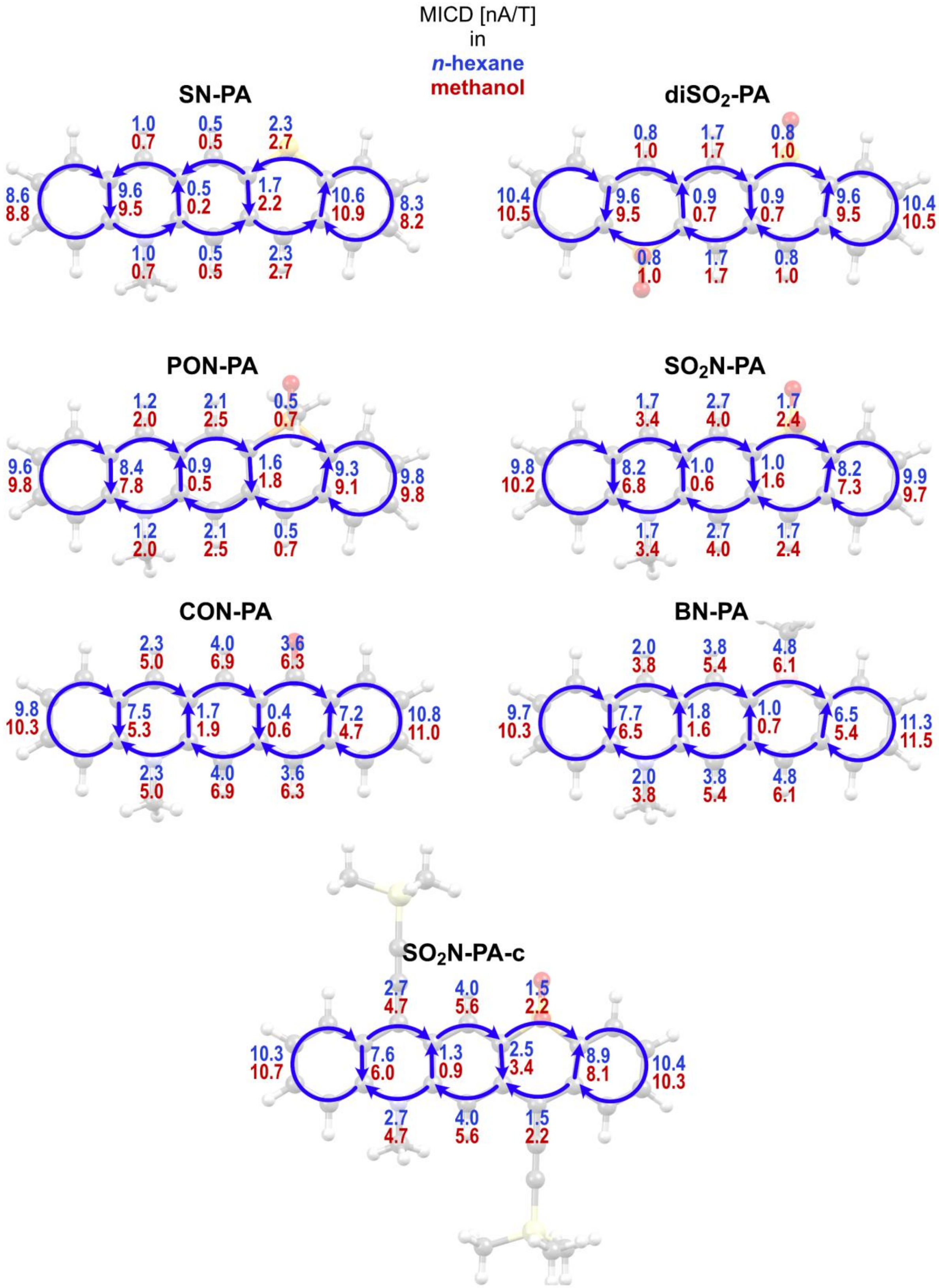

Figure S42. Magnetically induced current densities (MICD) calculated for pentacene analogues in *n*-hexane and methanol.

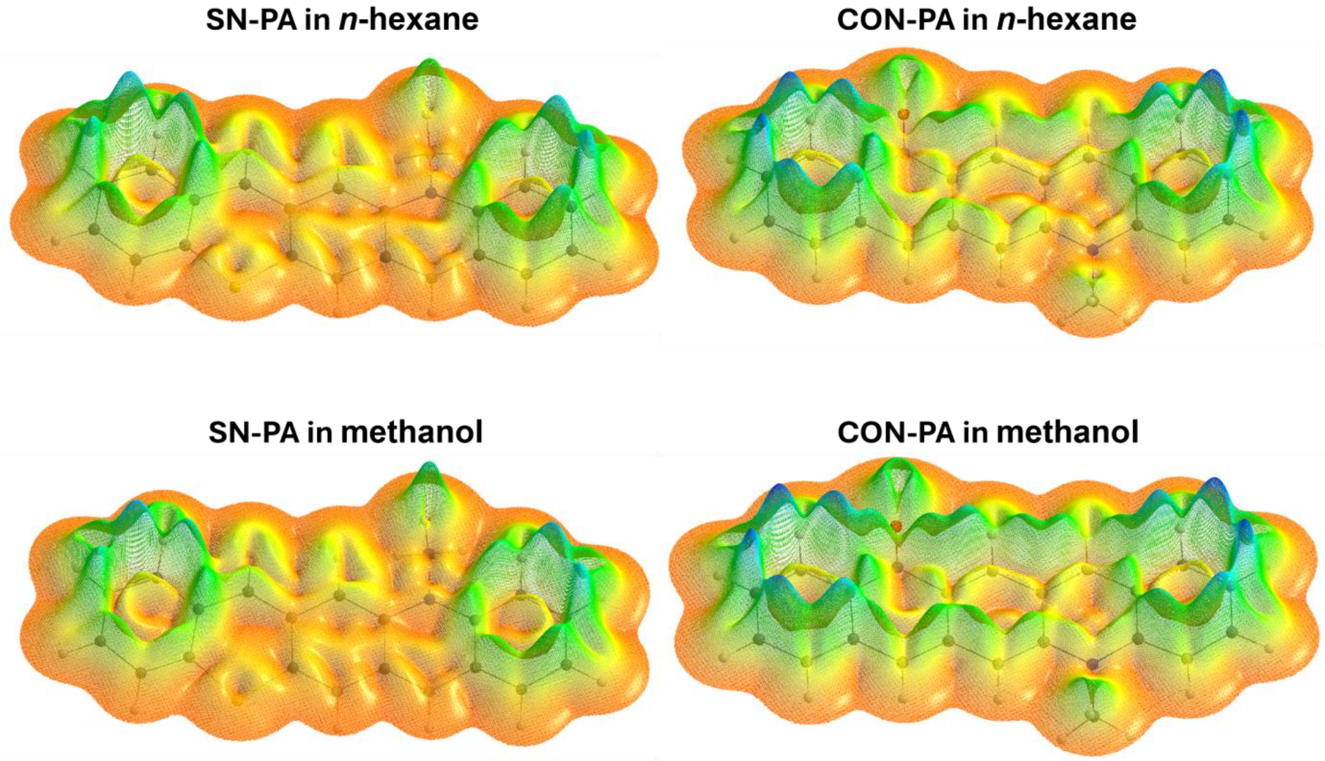

Figure S43. Relief maps present the distribution of the current density computed for the plane 1 Bohr (approximately 0.53 Å) above the molecular plane, where the π ring current is most intense. The height and colors are proportional to the current intensity at each point.

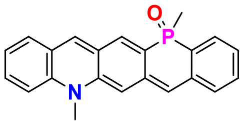

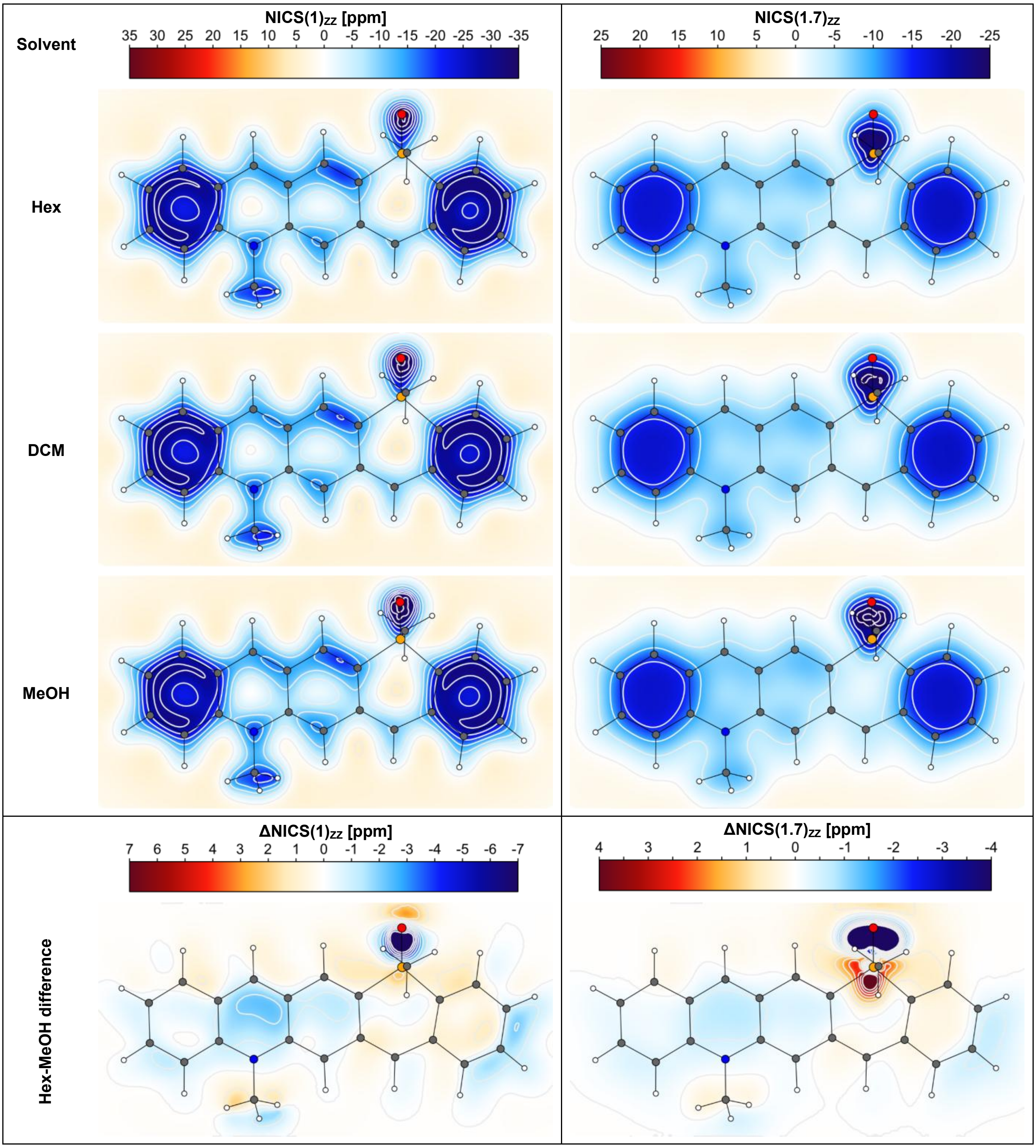

Figure S44. 2D NICS(1)$_{zz}$ (left) and NICS(1.7)$_{zz}$ (right) maps of **PON-PA** in *n*-hexane, dichloromethane, and methanol, along with a differential map (bottom) showing the *n*-hexane-methanol difference (ΔNICS(*h*)$_{zz}$ = NICS(*h*)$_{zz}^{MeOH}$ – NICS(*h*)$_{zz}^{Hex}$). Calculated at the GIAO/LC-ωHPBE(ω=0.2)/def2-TZVPP level with the SMD solvation model. Maps generated by *nics_map.py* and *nics_solv.py* scripts.

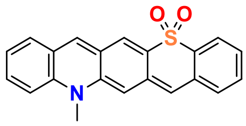

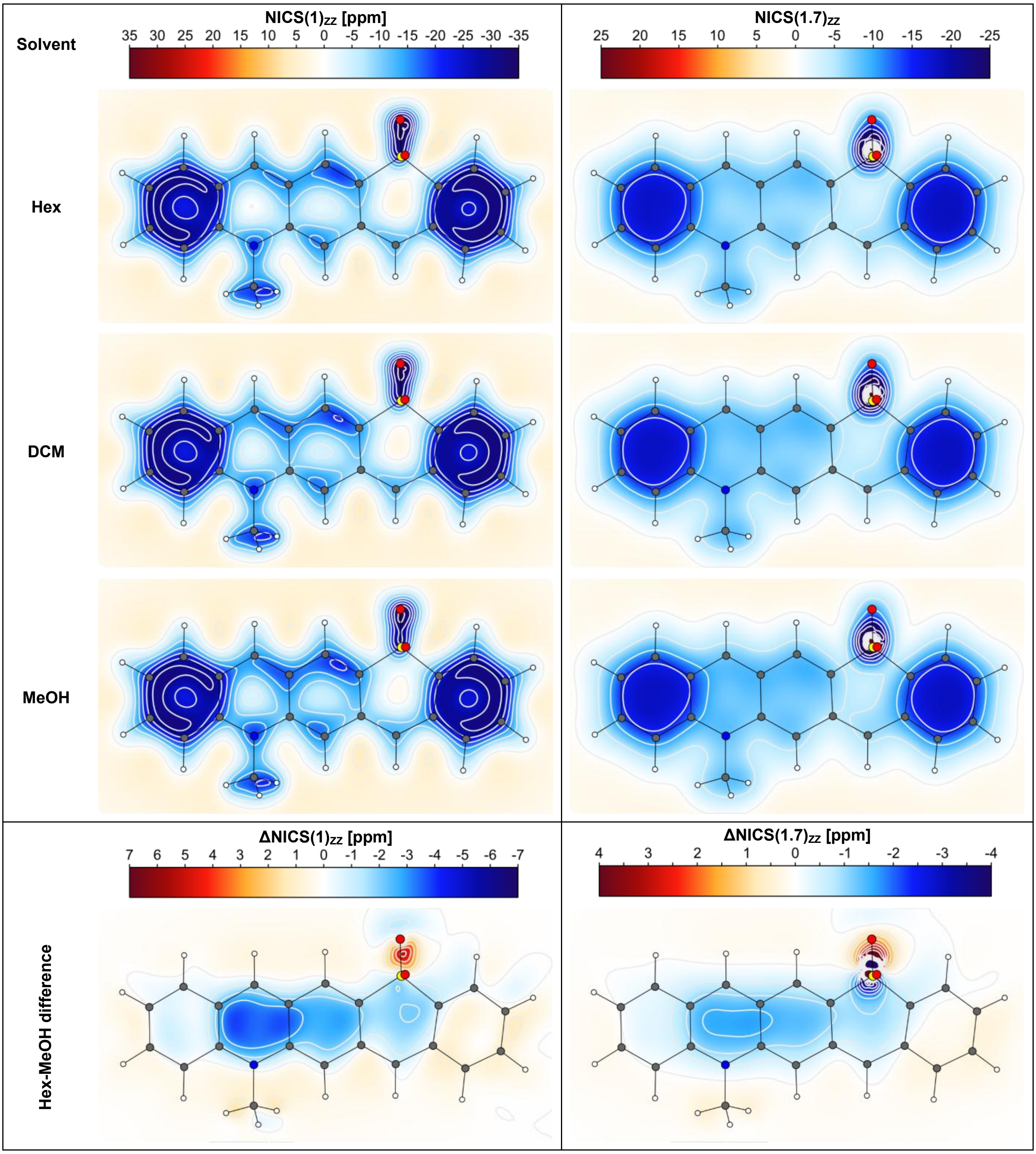

Figure S45. 2D NICS(1)$_{zz}$ (left) and NICS(1.7)$_{zz}$ (right) maps of **SO$_2$N-PA** in *n*-hexane, dichloromethane, and methanol, along with a differential map (bottom) showing the *n*-hexane-methanol difference (ΔNICS(*h*)$_{zz}$ = NICS(*h*)$_{zz}^{MeOH}$ – NICS(*h*)$_{zz}^{Hex}$). Calculated at the GIAO/LC-ωHPBE(ω=0.2)/def2-TZVPP level with the SMD solvation model. Maps generated by *nics_map.py* and *nics_solv.py* scripts.

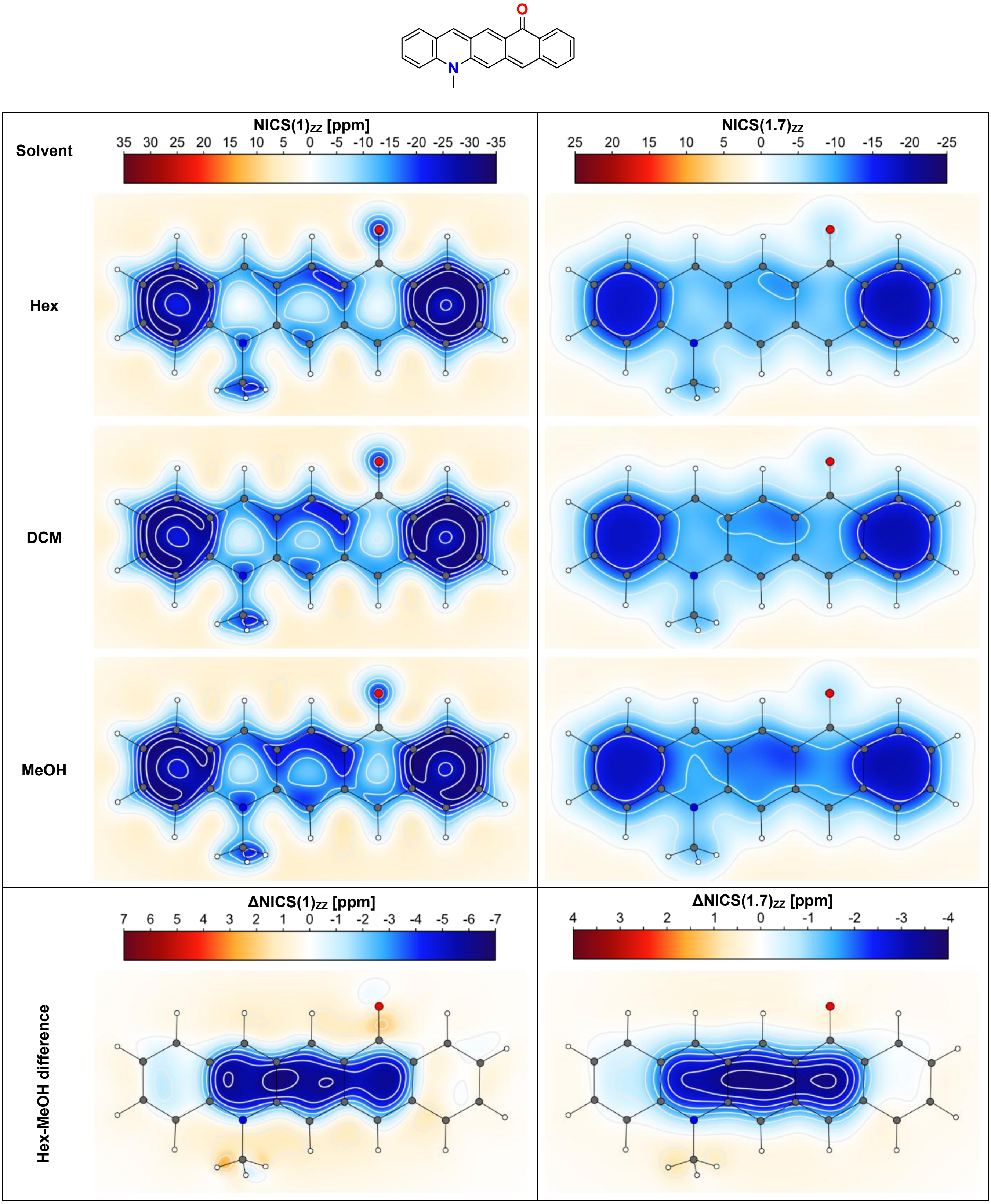

Figure S46. 2D $\text{NICS}(1)_{zz}$ (left) and $\text{NICS}(1.7)_{zz}$ (right) maps of **CON-PA** in *n*-hexane, dichloromethane, and methanol, along with a differential map (bottom) showing the *n*-hexane-methanol difference ($\Delta\text{NICS}(h)_{zz} = \text{NICS}(h)_{zz}^{\text{MeOH}} - \text{NICS}(h)_{zz}^{\text{Hex}}$). Calculated at the GIAO/LC-ωHPBE(ω=0.2)/def2-TZVPP level with the SMD solvation model. Maps generated by *nics_map.py* and *nics_solv.py* scripts.

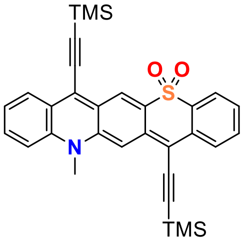

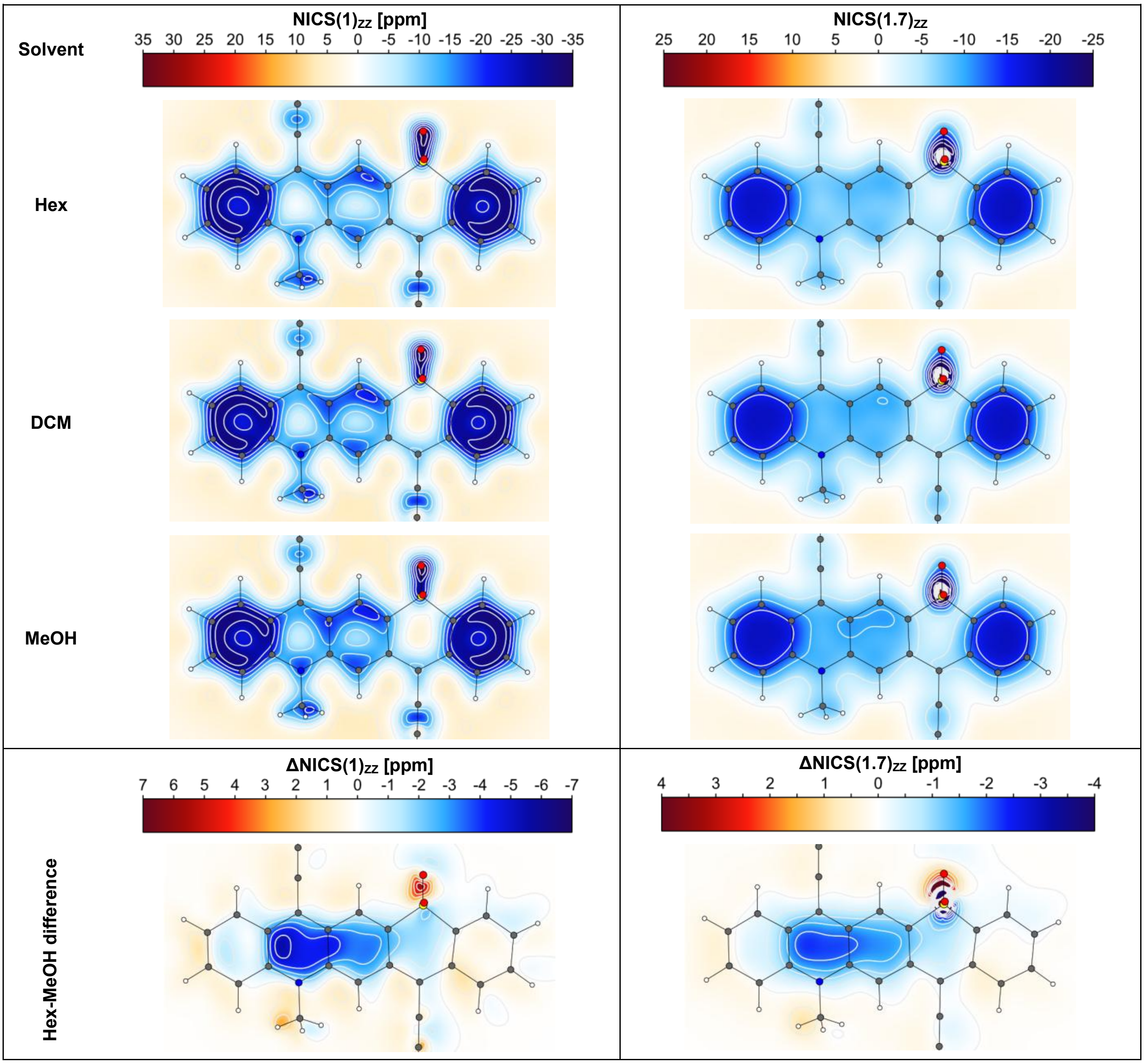

Figure S47. 2D NICS(1)$_{ZZ}$ (left) and NICS(1.7)$_{ZZ}$ (right) maps of **SO$_2$N-PA-c** in *n*-hexane, dichloromethane, and methanol, along with a differential map (bottom) showing the *n*-hexane-methanol difference (ΔNICS(*h*)$_{ZZ}$ = NICS(*h*)$_{ZZ}^{MeOH}$ − NICS(*h*)$_{ZZ}^{Hex}$). Calculated at the GIAO/LC-ωHPBE(ω=0.2)/def2-TZVPP level with the SMD solvation model. Maps generated by *nics_map.py* and *nics_solv.py* scripts.

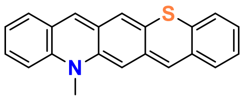

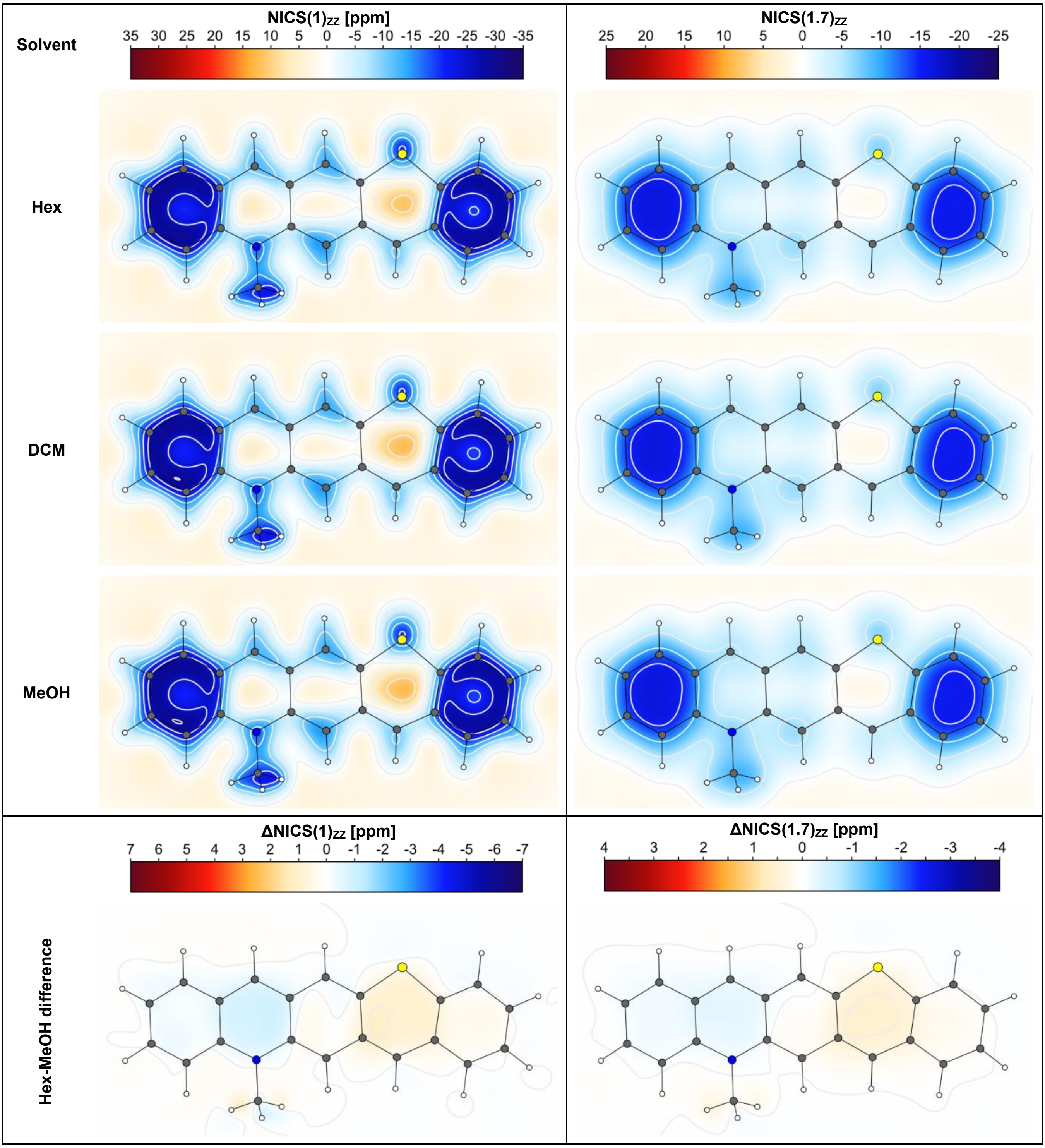

Figure S48. 2D NICS(1)$_{zz}$ (left) and NICS(1.7)$_{zz}$ (right) maps of **SN-PA** in *n*-hexane, dichloromethane, and methanol, along with a differential map (bottom) showing the *n*-hexane-methanol difference (ΔNICS(h)$_{zz}$ = NICS(h)$_{zz}^{MeOH}$ − NICS(h)$_{zz}^{Hex}$). Calculated at the GIAO/LC-ωHPBE(ω=0.2)/def2-TZVPP level with the SMD solvation model. Maps generated by *nics_map.py* and *nics_solv.py* scripts.

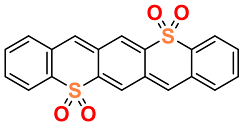

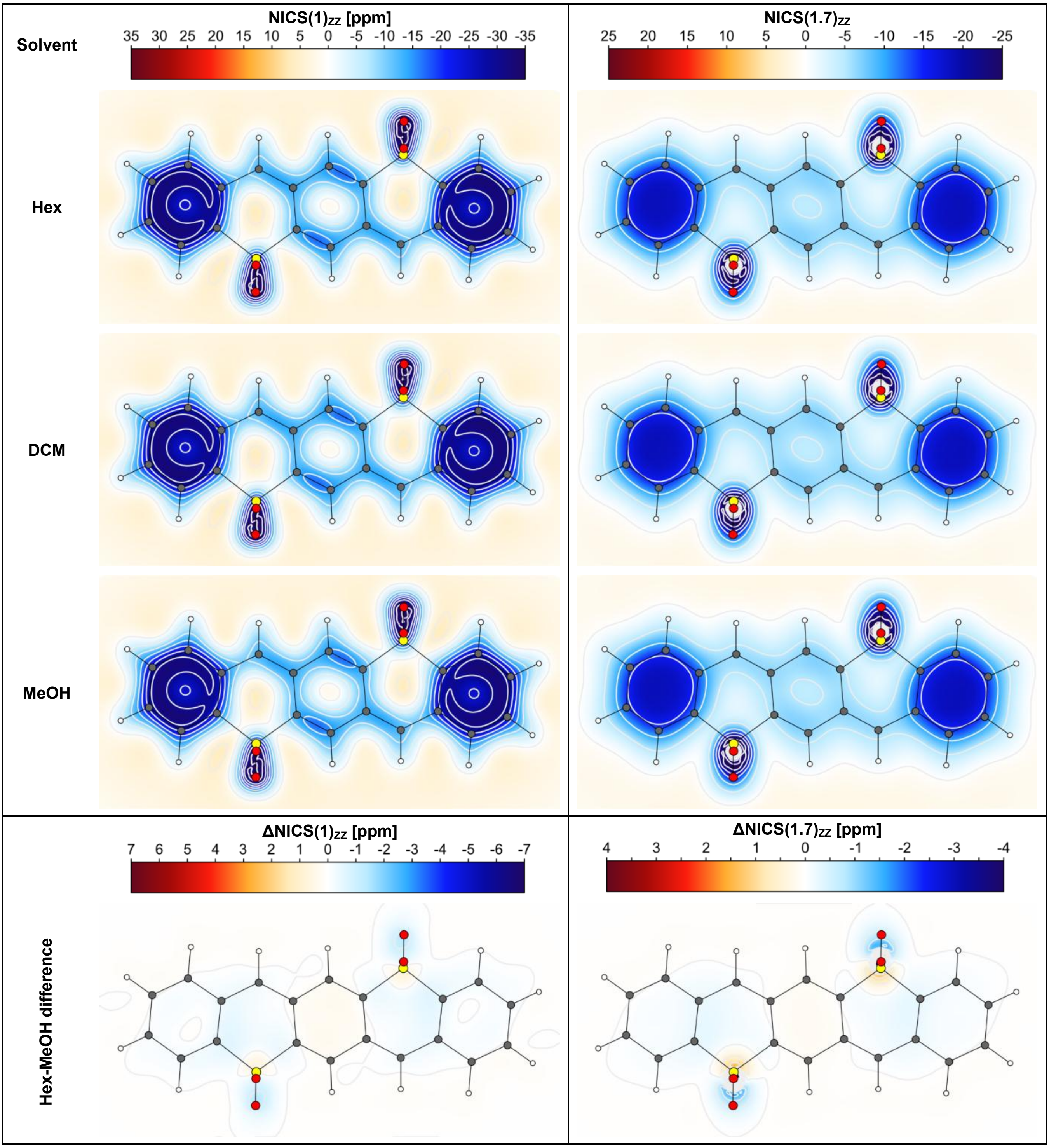

Figure S49. 2D NICS(1)zz (left) and NICS(1.7)zz (right) maps of **diSO₂-PA** in *n*-hexane, dichloromethane, and methanol, along with a differential map (bottom) showing the *n*-hexane-methanol difference ($\Delta NICS(h)_{zz} = NICS(h)_{zz}^{MeOH} - NICS(h)_{zz}^{Hex}$). Calculated at the GIAO/LC-ωHPBE(ω=0.2)/def2-TZVPP level with the SMD solvation model. Maps generated by *nics_map.py* and *nics_solv.py* scripts.

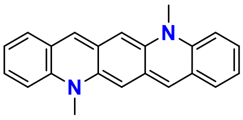

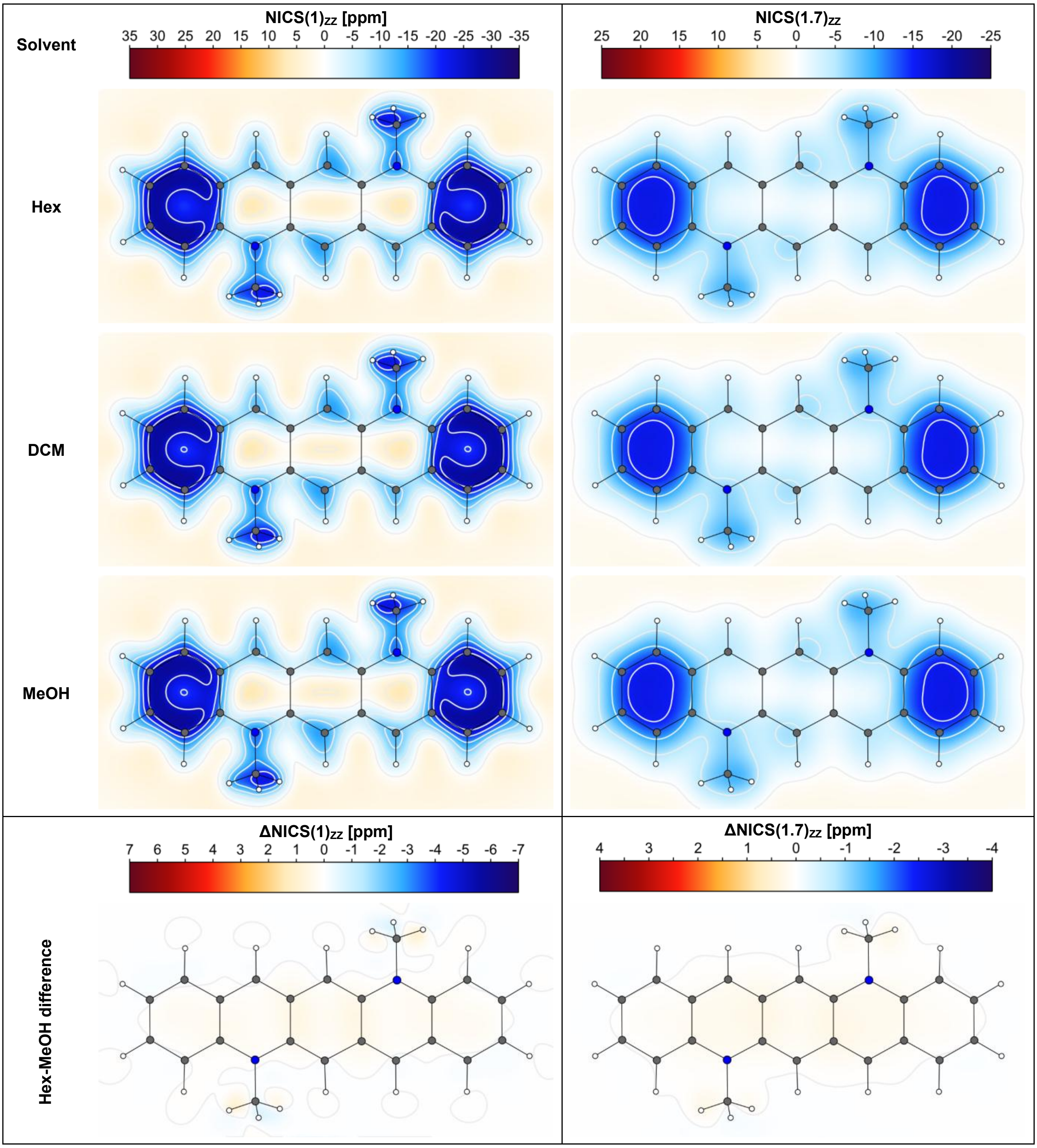

Figure S50. 2D NICS(1)zz (left) and NICS(1.7)zz (right) maps of **diN-PA** in *n*-hexane, dichloromethane, and methanol, along with a differential map (bottom) showing the *n*-hexane-methanol difference ($\Delta NICS(h)_{zz} = NICS(h)_{zz}^{MeOH} - NICS(h)_{zz}^{Hex}$). Calculated at the GIAO/LC-ωHPBE(ω=0.2)/def2-TZVPP level with the SMD solvation model. Maps generated by *nics_map.py* and *nics_solv.py* scripts.

Table S12. HOMA values calculated for the central *p*QDM ring of the DFT-optimized pentacene analogues using the *py.Aroma* software with standard parametrization. The data are color-coded from lowest (red) to highest (blue) values to reflect the discrete changes in aromaticity.

Simplified molecules

Compound	Hex	DCM	MeOH
**SN-PA**	0.27	0.27	0.27
**diSO$_2$-PA**	0.16	0.16	0.18
**PON-PA**	0.20	0.23	0.25
**SO$_2$N-PA**	0.25	0.30	0.34
**CON-PA**	0.29	0.35	0.42
**SO$_2$N-PA-c**	0.43	0.49	0.52

Complete molecules

Compound	Hex	DCM	MeOH
**SN-PA-a**		0.28	
**diSO$_2$-PA-a**		0.17	
**PON-PA**	0.25	0.28	0.32
**SO$_2$N-PA-a**	0.26	0.29	0.33
**CON-PA**	0.32	0.37	0.44
**SO$_2$N-PA-c**	0.41	0.45	0.49

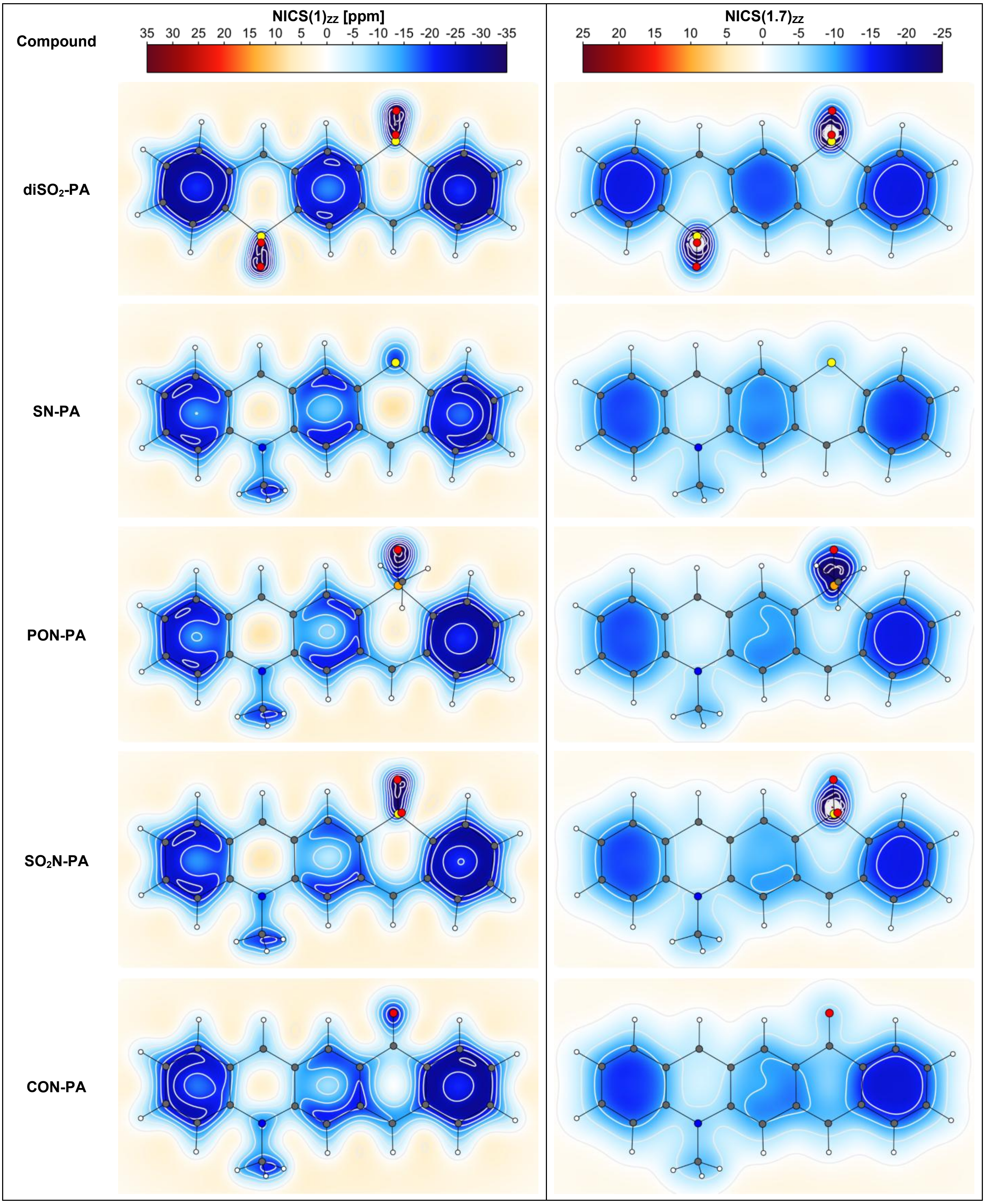

Figure S51. 2D NICS(1)zz (left) and NICS(1.7)zz (right) maps of pentacene analogues calculated for the first vertical triplet (T_1) state in dichloromethane. Calculated at the GIAO/uLC-ωHPBE(ω=0.2)/def2-TZVPP level with the SMD solvation model. Maps generated by *nics_map.py* and *nics_solv.py* scripts.

6. Cyclic voltammetry

Electrochemical characterization of the synthesized compounds was performed in a three-electrode, custom-made V-shaped glass cell using a glassy carbon working electrode (2 mm diameter), a platinum wire counter electrode, and a silver wire quasi-reference electrode. To ensure reliable potential calibration, the ferrocene/ferrocenium (Fc/Fc^+) redox couple was used as an internal standard. All reported potentials are given relative to the formal potential of the Fc/Fc^+ couple.
Prior to each experiment, the glassy carbon electrode was thoroughly polished with a 0.05 µm alumina slurry and rinsed with isopropanol and o-dichlorobenzene. Measurements were carried out in 0.1M tetrabutylammonium hexafluorophosphate ($(TBA)PF_6$) in *o*-dichlorobenzene (ODB) containing the analyte (0.9 - 2 mM). Cyclic voltammetry (CV) was performed at a scan rate of 100 $mV{\cdot}s^{-1}$, while square-wave voltammetry (SWV) was conducted using a frequency of 25 Hz, a 1 mV step, and a 10 mV pulse amplitude. Absolute HOMO and LUMO energy levels were estimated from the first oxidation and reduction potentials according to Equations (1) and (2).[38]

E_{HOMO} (eV) = −e (E_{ox} (V) + 4.71 V) (1)

E_{LUMO} (eV) = −e (E_{red} (V) + 4.71 V) (2)

Optical bandgaps were calculated from the onsets of the absorption spectra in DCM.

Table S13. Formal potentials of the first reversible anodic and cathodic redox processes of pentacene analogues measured in o-dichlorobenzene.

Compound	E_{ox} [a] [V]	E_{red} [a] [V]	E_{HOMO} [b] [eV]	E_{LUMO} [b] [eV]	$E_{g(CV)}$ [c] [eV]	$E_{g(opt)}$ [d] [eV]
**diSO$_2$-PA-a**	0.826 [e]	−1.234 [e]	−5.54	−3.48	2.06	2.33
**SN-PA-a**	−0.419, +0.281	−2.503 [e]	−4.29	−2.21	2.08	1.90
**PON-PA**	−0.388	−2.081	−4.32	−2.63	1.69	1.37
**SO$_2$N-PA-a**	0.022	−1.616	−4.73	−3.09	1.64	1.29
**CON-PA**	0.395 [e]	−1.108	−5.01	−3.60	1.40	1.00
**SN-PA-c**	−0.128, +0.524	−1.782	−4.58	−2.93	1.65	1.66
**diSO$_2$-PA-c**	0.934 [e]	−0.910, −1.416	−5.64	−3.80	1.84	2.13
**SO$_2$N-PA-c**	−0.093	−1.413	−4.62	−3.30	1.32	1.14

[a] vs. Fc/Fc^+.
[b] Calculated using the energy of an electron in vacuum (−4.71 eV).
[c] Electrochemical bandgap.
[d] Optical bandgap calculated from the onset of the absorption spectrum in DCM.
[e] Irreversible process; onset potential is reported.

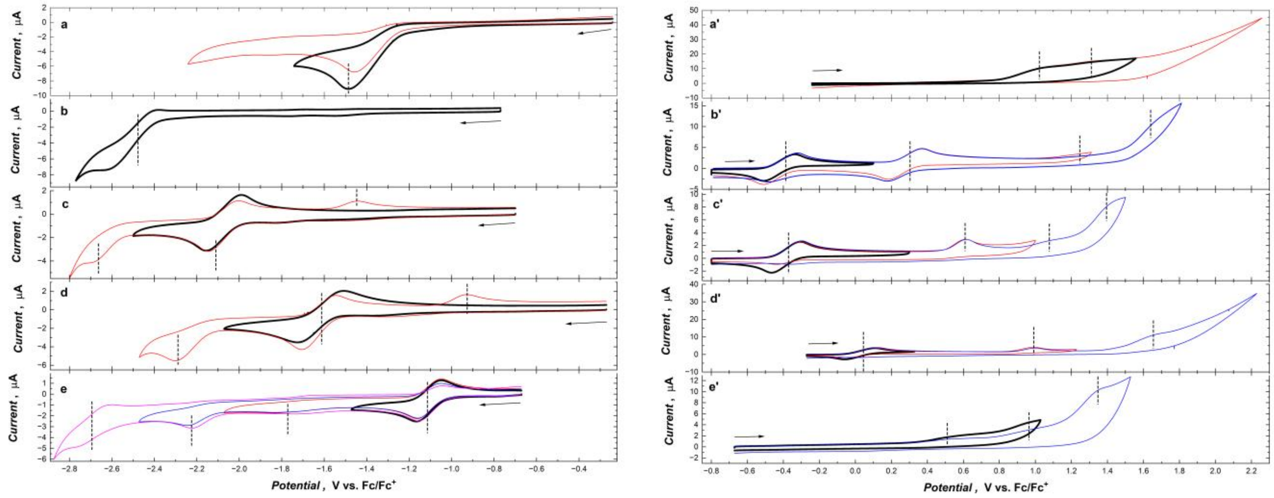

Figure S52. Cyclic voltammograms of (a, a') **diSO$_2$-PA-a**, (b, b') **SN-PA-a**, (c, c') **PON-PA**, (d, d') **SO$_2$N-PA-a**, and (e, e') **CON-PA**, recorded over extended potential ranges: negative (a–e) and positive (a'–e'). Measurements were performed in 0.1M (TBA)PF$_6$ solution in *o*-dichlorobenzene using a glassy carbon working electrode. Scan rate:100 mV s^{-1}.

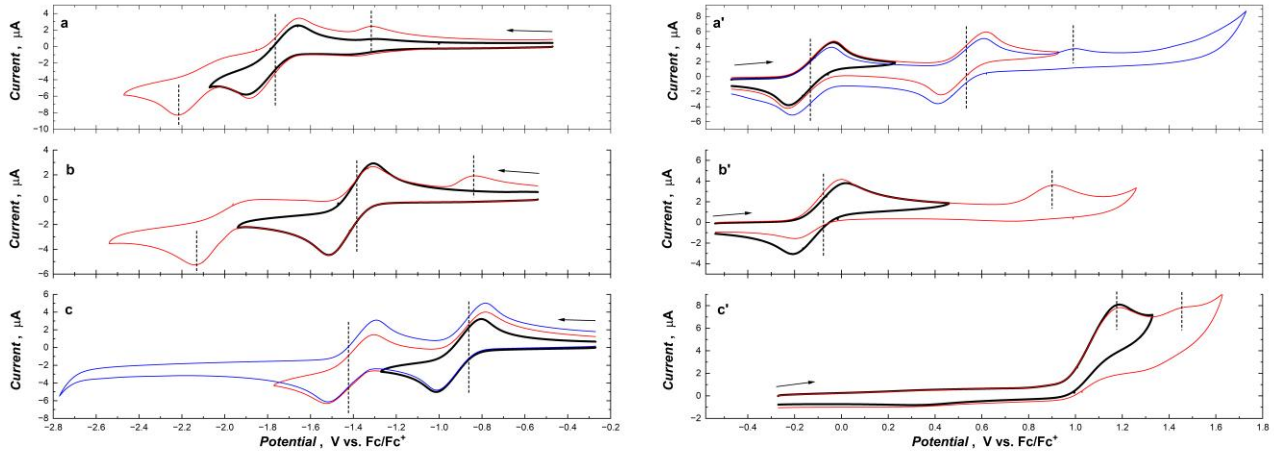

Figure S53. Cyclic voltammograms of (a, a') **SN-PA-c**, (b, b') **SO$_2$N-PA-c**, and (c, c') **diSO$_2$-PA-c**, recorded over extended potential ranges: negative (a–c) and positive (a'–c'). Measurements were performed in 0.1M (TBA)PF$_6$ solution in *o*-dichlorobenzene using a glassy carbon working electrode. Scan rate:100 mV s^{-1}.

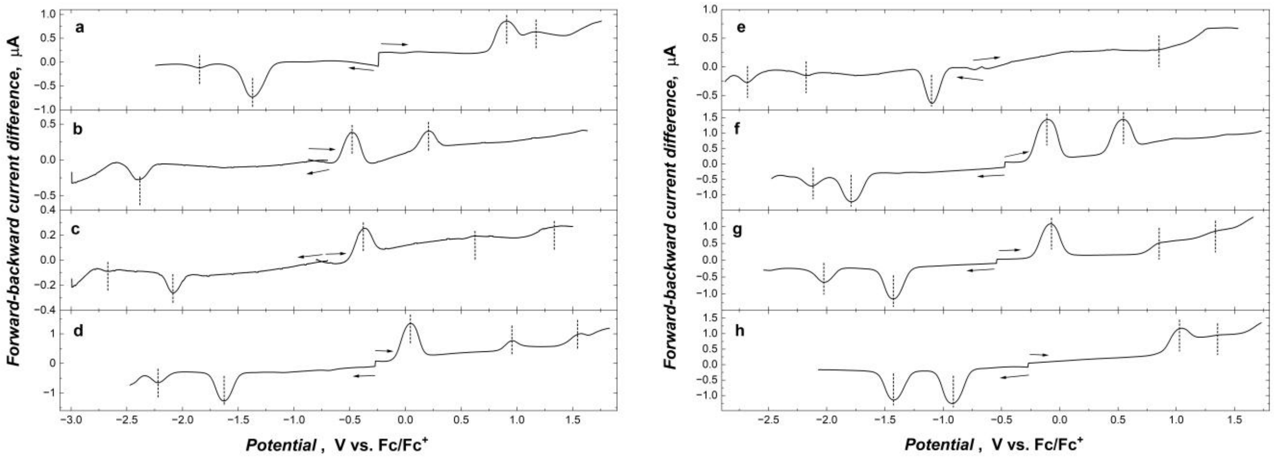

Figure S54. Square-wave voltammograms of (a) **diSO$_2$-PA-a**, (b) **SN-PA-a**, (c) **PON-PA**, (d) **SO$_2$N-PA-a**, (e) **CON-PA**, (f) **SN-PA-c**, (g) **SO$_2$N-PA-c**, and (h) **diSO$_2$-PA-c**. Measurements were performed in 0.1M (TBA)PF$_6$ solution in *o*-dichlorobenzene using a glassy carbon working electrode. Frequency: 25 Hz; pulse height: 10 mV; potential step: 1 mV.

7. Bibliography

[1] O. V. Dolomanov, L. J. Bourhis, R. J. Gildea, J. A. K. Howard, H. Puschmann, “OLEX2: A complete structure solution, refinement and analysis program” *J. Appl. Crystallogr.* **2009**, *42*, 339–341.

[2] G. M. Sheldrick, “A short history of SHELX” *Acta Crystallogr. A* **2008**, *64*, 112–122.

[3] L. J. Bourhis, O. V Dolomanov, R. J. Gildea, J. A. K. Howard, H. Puschmann, “The anatomy of a comprehensive constrained, restrained refinement program for the modern computing environment – Olex2 dissected” *Acta Crystallogr., Sect. A:Found. Adv.* **2015**, *71*, 59–75.

[4] G. M. Sheldrick, “Crystal structure refinement with SHELXL” *Acta Crystallogr., Sect. C:Struct. Chem.* **2015**, *71*, 3–8.

[5] C. F. Macrae, I. Sovago, S. J. Cottrell, P. T. A. Galek, P. McCabe, E. Pidcock, M. Platings, G. P. Shields, J. S. Stevens, M. Towler, P. A. Wood, “Mercury 4.0 : from visualization to analysis, design and prediction” *J. Appl. Crystallogr.* **2020**, *53*, 226–235.

[6] H. H. Nguyen, X. Li, N. Wang, Z. Y. Wang, M. Jianjun, W. J. Bock, M. Dongge, “Fiber-optic detection of explosives using readily available fluorescent polymers” *Macromolecules* **2009**, *42*, 921–926.

[7] B. Meindl, K. Pfennigbauer, B. Stöger, M. Heeney, F. Glöcklhofer, “Double Ring-Closing Approach for the Synthesis of 2,3,6,7-Substituted Anthracene Derivatives” *J. Org. Chem.* **2020**, *85*, 8240–8244.

[8] Q. Ye, J. Chang, X. Shi, G. Dai, W. Zhang, K.-W. Huang, C. Chi, “Stable 7,14-Disubstituted-5,12-Dithiapentacenes with Quinoidal Conjugation” *Org. Lett.* **2014**, *16*, 3966–3969.

[9] B. He, L. S. Zheng, P. Phansavath, V. Ratovelomanana-Vidal, “RhIII-Catalyzed Asymmetric Transfer Hydrogenation of α-Methoxy β-Ketoesters through DKR in Water: Toward a Greener Procedure” *ChemSusChem* **2019**, *12*, 3032–3036.

[10] C. Reichardt, “Empirical Parameters of Solvent Polarity as Linear Free-Energy Relationships” *Angew. Chem. Int. Ed.* **1979**, 18, 98–110.

[11] X. Li, Y. Yang, R. Zhang, W. Huang, “Construction and optimization of organic fluorophores in NIR-II fluorescence imaging” *Chem. Soc. Rev.* **2025**, 54, 11184–11225.

[12] H. J. Zhou, T. B. Ren, “Recent Progress of Cyanine Fluorophores for NIR-II Sensing and Imaging” *Chem. Asian J.* **2022**, *17*, e202200147.

[13] A. L. Antaris, H. Chen, S. Diao, Z. Ma, Z. Zhang, S. Zhu, J. Wang, A. X. Lozano, Q. Fan, L. Chew, M. Zhu, K. Cheng, X. Hong, H. Dai, Z. Cheng, “A high quantum yield molecule-protein complex fluorophore for near-infrared II imaging” *Nat. Commun.* **2017**, 8, 15269.

[14] E. D. Cosco, B. A. Arús, A. L. Spearman, T. L. Atallah, I. Lim, O. S. Leland, J. R. Caram, T. S. Bischof, O. T. Bruns, E. M. Sletten, “Bright Chromenylium Polymethine Dyes Enable Fast, Four-Color in Vivo Imaging with Shortwave Infrared Detection” *J. Am. Chem. Soc.* **2021**, *143*, 6836–6846.

[15] B. Li, L. Lu, M. Zhao, Z. Lei, F. Zhang, “An Efficient 1064 nm NIR-II Excitation Fluorescent Molecular Dye for Deep-Tissue High-Resolution Dynamic Bioimaging” *Angew. Chem. Int. Ed.* **2018**, *57*, 7483–7487.

[16] B. Li, M. Zhao, L. Feng, C. Dou, S. Ding, G. Zhou, L. Lu, H. Zhang, F. Chen, X. Li, G. Li, S. Zhao, C. Jiang, Y. Wang, D. Zhao, Y. Cheng, F. Zhang, “Organic NIR-II molecule with long blood half-life for in vivo dynamic vascular imaging” *Nat. Commun.* **2020**, *11*, 3102.

[17] A. C. Benniston, A. Harriman, P. Li, J. P. Rostron, J. W. Verhoeven, “Illumination of the 9-mesityl-10-methylacridinium ion does not give a long-lived photoredox state” *Chem. Commun.* **2005**, 2701–2703.

[18] Gaussian 16, Revision C.01, M. J. Frisch, G. W. Trucks, H. B. Schlegel, G. E. Scuseria, M. A. Robb, J. R. Cheeseman, G. Scalmani, V. Barone, G. A. Petersson, H. Nakatsuji, X. Li, M. Caricato, A. V Marenich, J. Bloino, B. G. Janesko, R. Gomperts, B. Mennucci, H. P. Hratchian, J. V Ortiz, A. F. Izmaylov, J. L. Sonnenberg, D. Williams-Young, F. Ding, F. Lipparini, F. Egidi, J. Goings, B. Peng, A. Petrone, T. Henderson, D. Ranasinghe, V. G. Zakrzewski, J. Gao, N. Rega, G. Zheng, W. Liang, M. Hada, M. Ehara, K. Toyota, R. Fukuda, J. Hasegawa, M. Ishida, T. Nakajima, Y. Honda, O. Kitao, H. Nakai, T. Vreven, K. Throssell, J. A. Montgomery Jr., J. E. Peralta, F. Ogliaro, M. J. Bearpark, J. J. Heyd, E. N. Brothers, K. N. Kudin, V. N. Staroverov, T. A. Keith, R. Kobayashi, J. Normand, K. Raghavachari, A. P. Rendell, J. C. Burant, S. S. Iyengar, J. Tomasi, M. Cossi, J. M. Millam, M. Klene, C. Adamo, R. Cammi, J. W. Ochterski, R. L. Martin, K. Morokuma, O. Farkas, J. B. Foresman, D. J. Fox, Gaussian Inc., Wallingford CT, **2016**.

[19] N. J. Hestand, H. Yamagata, B. Xu, D. Sun, Y. Zhong, A. R. Harutyunyan, G. Chen, H. L. Dai, Y. Rao, F. C. Spano, “Polarized absorption in crystalline pentacene: Theory vs experiment” *J. Phys. Chem. C* **2015**, *119*, 22137–22147.

[20] D. T. Chase, A. G. Fix, S. J. Kang, B. D. Rose, C. D. Weber, Y. Zhong, L. N. Zakharov, M. C. Lonergan, C. Nuckolls, M. M. Haley, “6,12-Diarylindeno[1,2- b]fluorenes: Syntheses, photophysics, and ambipolar OFETs” *J. Am. Chem. Soc.* **2012**, *134*, 10349–10352.

[21] Y. Zhao, D. G. Truhlar, “The M06 suite of density functionals for main group thermochemistry, thermochemical kinetics, noncovalent interactions, excited states, and transition elements: Two new functionals and systematic testing of four M06-class functionals and 12 other function” *Theor. Chem. Acc.* **2008**, *120*, 215–241.

[22] F. Weigend, R. Ahlrichs, “Balanced basis sets of split valence, triple zeta valence and quadruple zeta valence quality for H to Rn: Design and assessment of accuracy” *Phys. Chem. Chem. Phys.* **2005**, *7*, 3297–3305.

[23] A. V. Marenich, C. J. Cramer, D. G. Truhlar, “Universal solvation model based on solute electron density and on a continuum model of the solvent defined by the bulk dielectric constant and atomic surface tensions” *J. Phys. Chem. B* **2009**, *113*, 6378–6396.

[24] J. Liang, X. Feng, D. Hait, M. Head-Gordon, “Revisiting the Performance of Time-Dependent Density Functional Theory for Electronic Excitations: Assessment of 43 Popular and Recently Developed Functionals from Rungs One to Four” *J. Chem. Theory. Comput.* **2022**, *18*, 6, 3460–3473.

[25] R. Sarkar, M. Boggio-Pasqua, P. F. Loos, D. Jacquemin, “Benchmarking TD-DFT and Wave Function Methods for Oscillator Strengths and Excited-State Dipole Moments” *J. Chem. Theory. Comput.* **2021**, *17*, 1117–1132.

[26] I. Knysh, F. Lipparini, A. Blondel, I. Duchemin, X. Blase, P. F. Loos, D. Jacquemin, “Reference CC3 Excitation Energies for Organic Chromophores: Benchmarking TD-DFT, BSE/GW, and Wave Function Methods” *J. Chem. Theory. Comput.* **2024**, 20, *18*, 8152–8174.

[27] O. A. Vydrov, G. E. Scuseria, “Assessment of a long-range corrected hybrid functional” *J. Chem. Phys.* **2006**, *125*, 234109.

[28] T. M. Henderson, A. F. Izmaylov, G. Scalmani, G. E. Scuseria, “Can short-range hybrids describe long-range-dependent properties?” *J. Chem. Phys.* **2009**, *131*, 044108.

[29] Jmol: an open-source Java viewer for chemical structures in 3D. http://www.jmol.org/

[30] J. D. Hunter, “Matplotlib: A 2D graphics environment” *Comput. Sci. Eng.* **2007**, *9*, 90–95.

[31] Z. Chen, C. S. Wannere, C. Corminboeuf, R. Puchta, P. von Ragué Schleyer, "Nucleus-independent chemical shifts (NICS) as an aromaticity criterion" *Chem. Rev.* **2005**, *105*, 3842–3888.

[32] R. Gershoni-Poranne, A. Stanger, "The NICS-XY-Scan: Identification of Local and Global Ring Currents in Multi-Ring Systems" *Chem. Eur. J.* **2014**, *20*, 5673–5688.

[33] K. Wolinski, J. F. Hinton, P. Pulay, "Efficient implementation of the gauge-independent atomic orbital method for NMR chemical shift calculations" *J. Am. Chem. Soc.* **1990**, *112*, 8251–8260.

[34] AIMAll (Version 19.10.12), Todd A. Keith, TK Gristmill Software, Overland Park KS, USA, 2019 (aim.tkgristmill.com).

[35] J. Kruszewski, T. M. Krygowski, "Definition of aromaticity basing on the harmonic oscillator model" *Tetrahedron Lett.* **1972**, *13*, 3839–3842.

[36] T. M. Krygowski, H. Szatylowicz, O. A. Stasyuk, J. Dominikowska, M. Palusiak, "Aromaticity from the viewpoint of molecular geometry: Application to planar systems" *Chem. Rev.* **2014**, *114*, 6383–6422.

[37] Z. Wang, "py.Aroma: An Intuitive Graphical User Interface for Diverse Aromaticity Analyses" *Chemistry* **2024**, *6*, 1692–1703.

[38] J. L. Wang, X. Li, C. D. Shreiner, X. Lu, C. N. Moorefield, S. R. Tummalapalli, D. A. Medvetz, M. J. Panzner, F. R. Fronczek, C. Wesdemiotis, G. R. Newkome, "Shape-persistent, ruthenium(II)- and iron(II)-bisterpyridine metallodendrimers: synthesis, traveling-wave ion-mobility mass spectrometry, and photophysical properties" *New J. Chem.* **2012**, *36*, 484–491.

8. NMR spectra

NMR spectra of the reported compounds are given on the following pages.

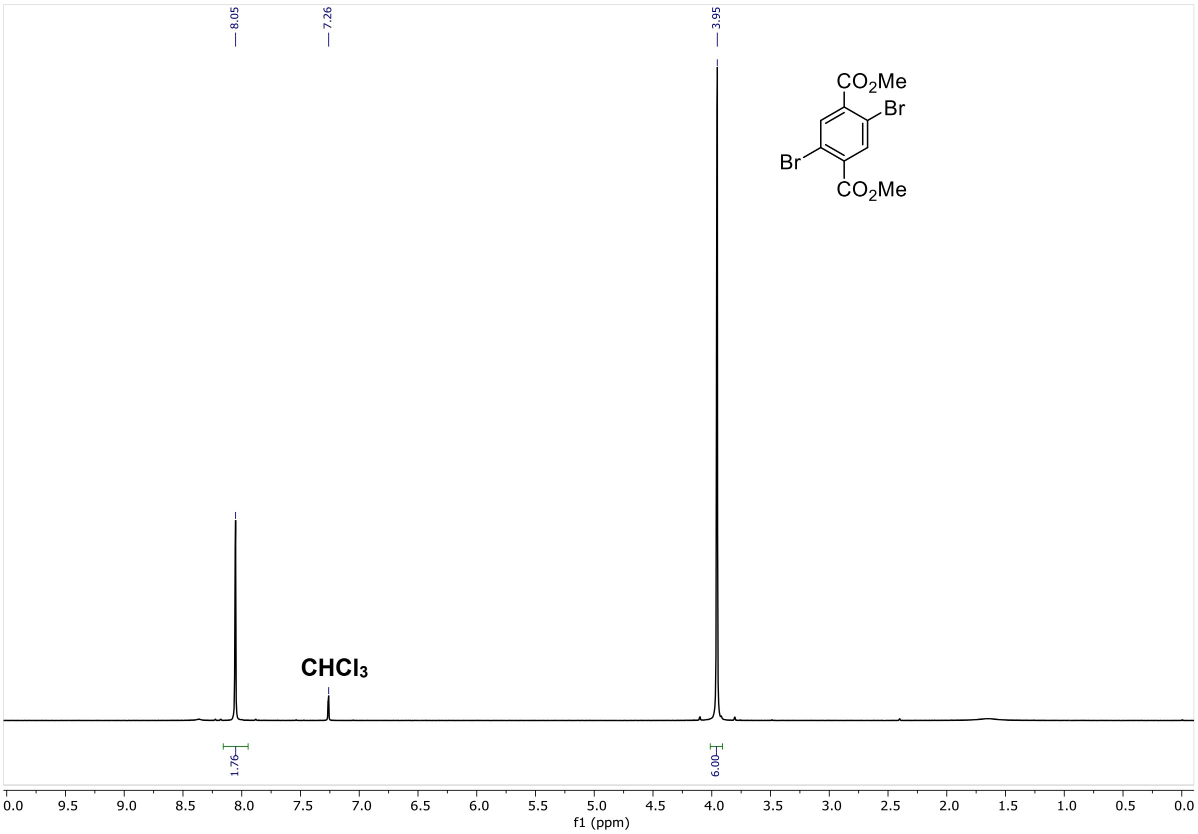

Figure S55. ^{1}H NMR spectrum of **3** in $CDCl_3$.

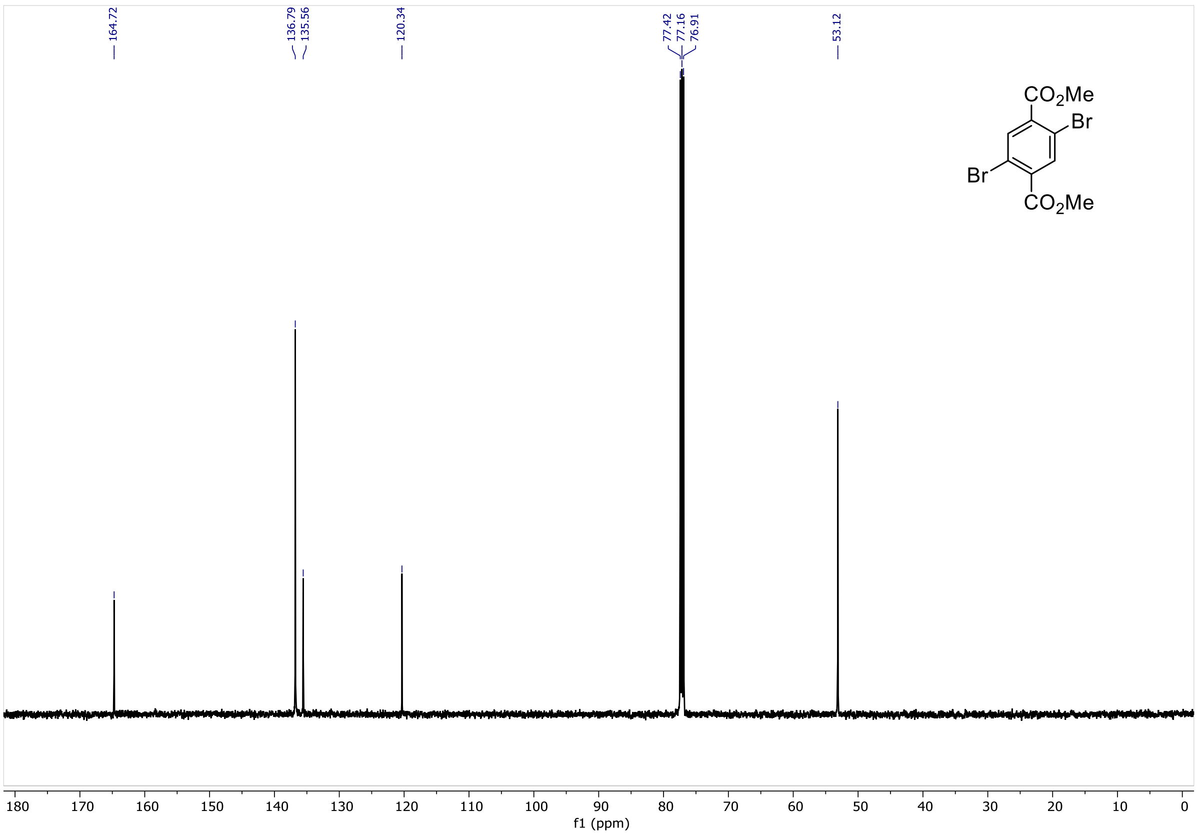

Figure S56. ^{13}C NMR spectrum of **3** in $CDCl_3$.

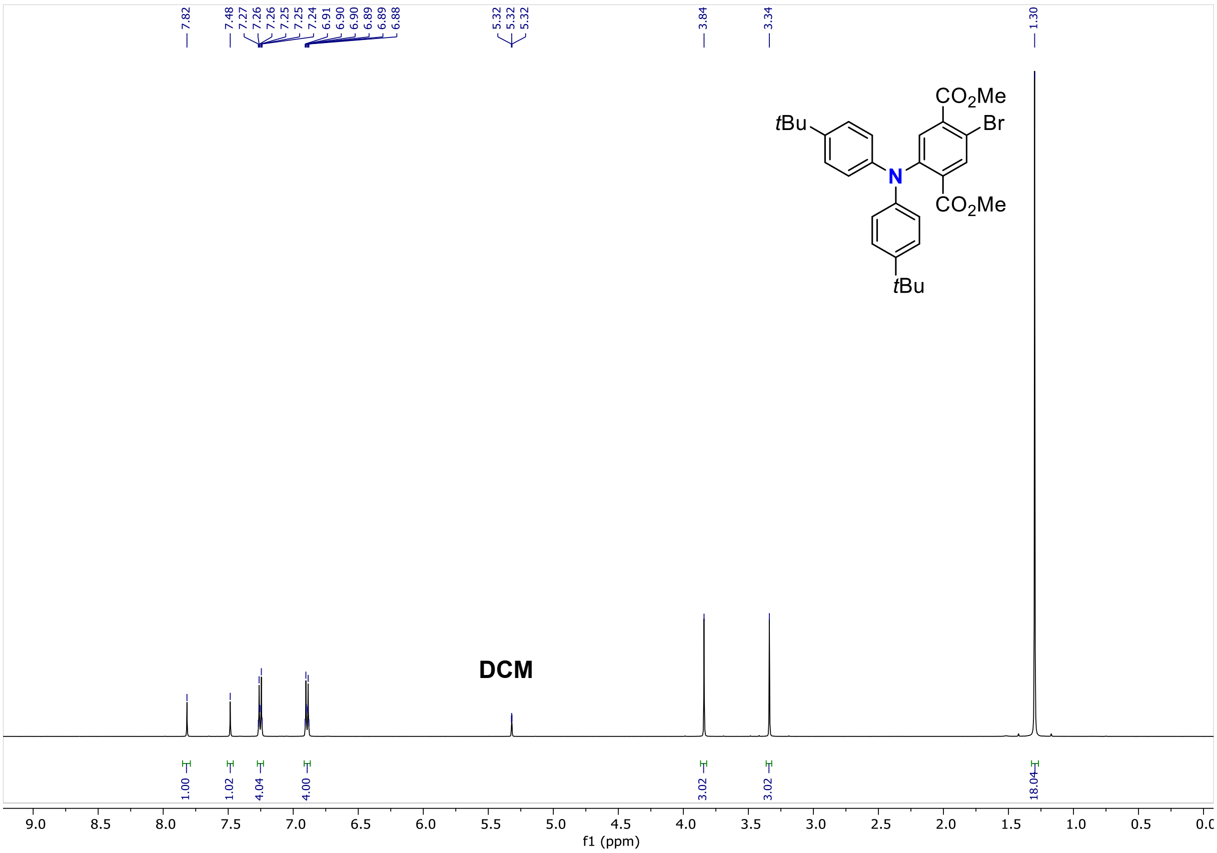

Figure S57. ^{1}H NMR spectrum of **4** in CD_2Cl_2.

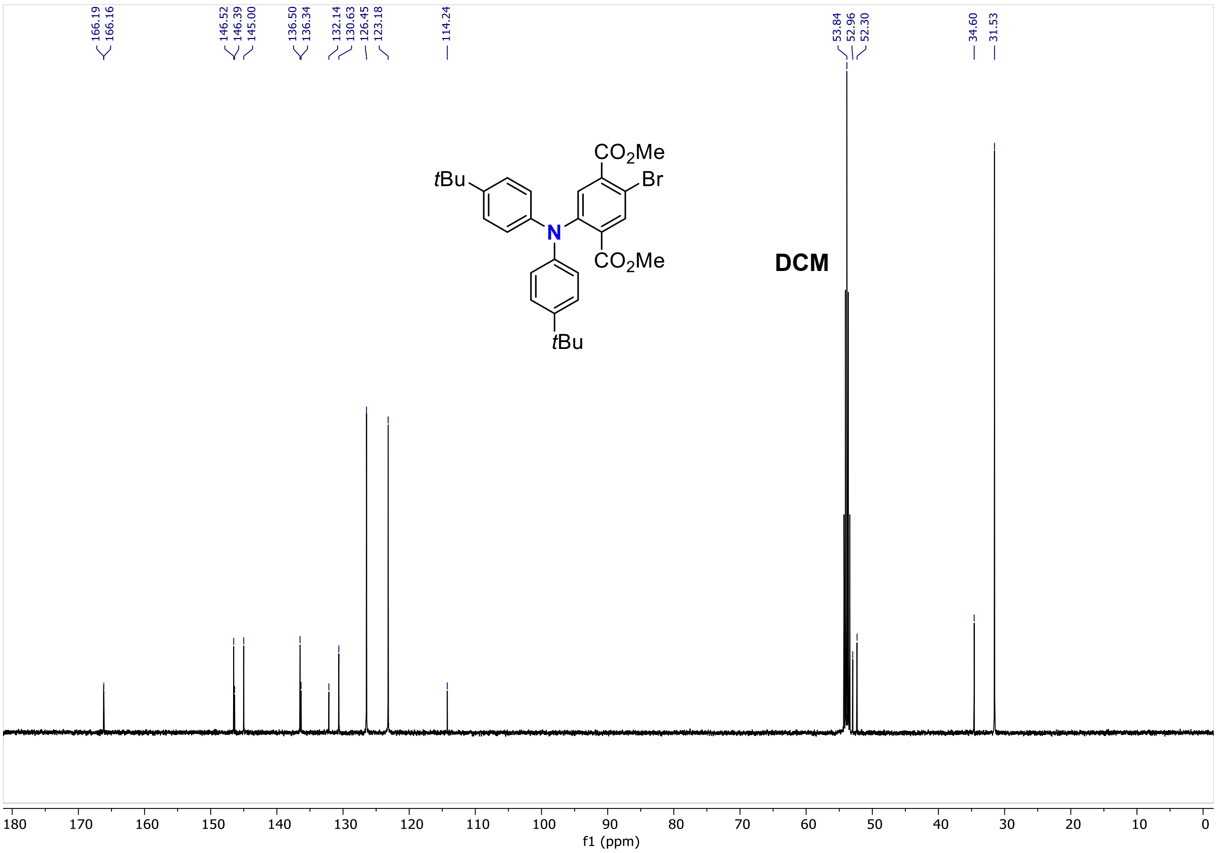

Figure S58. ^{13}C NMR spectrum of **4** in CD_2Cl_2.

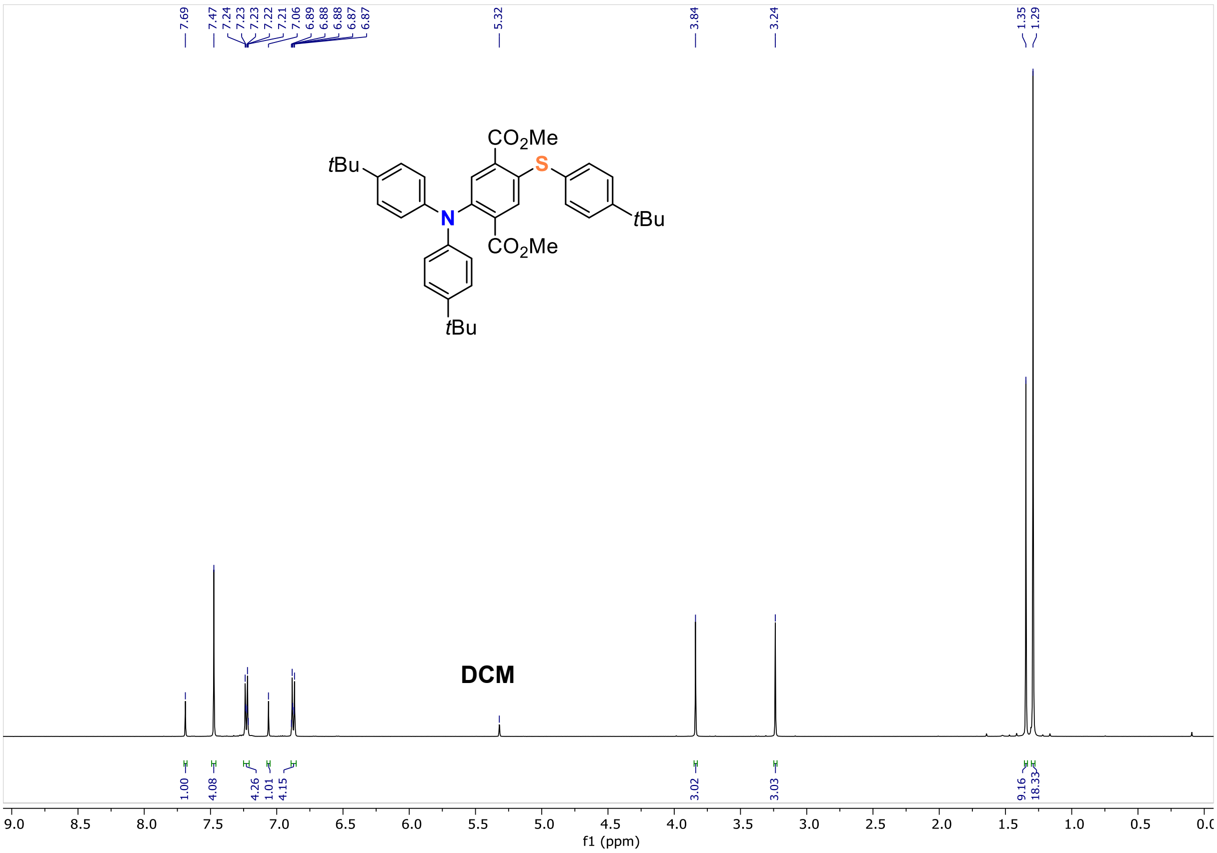

Figure S59. ^{1}H NMR spectrum of **S1** in CD_2Cl_2.

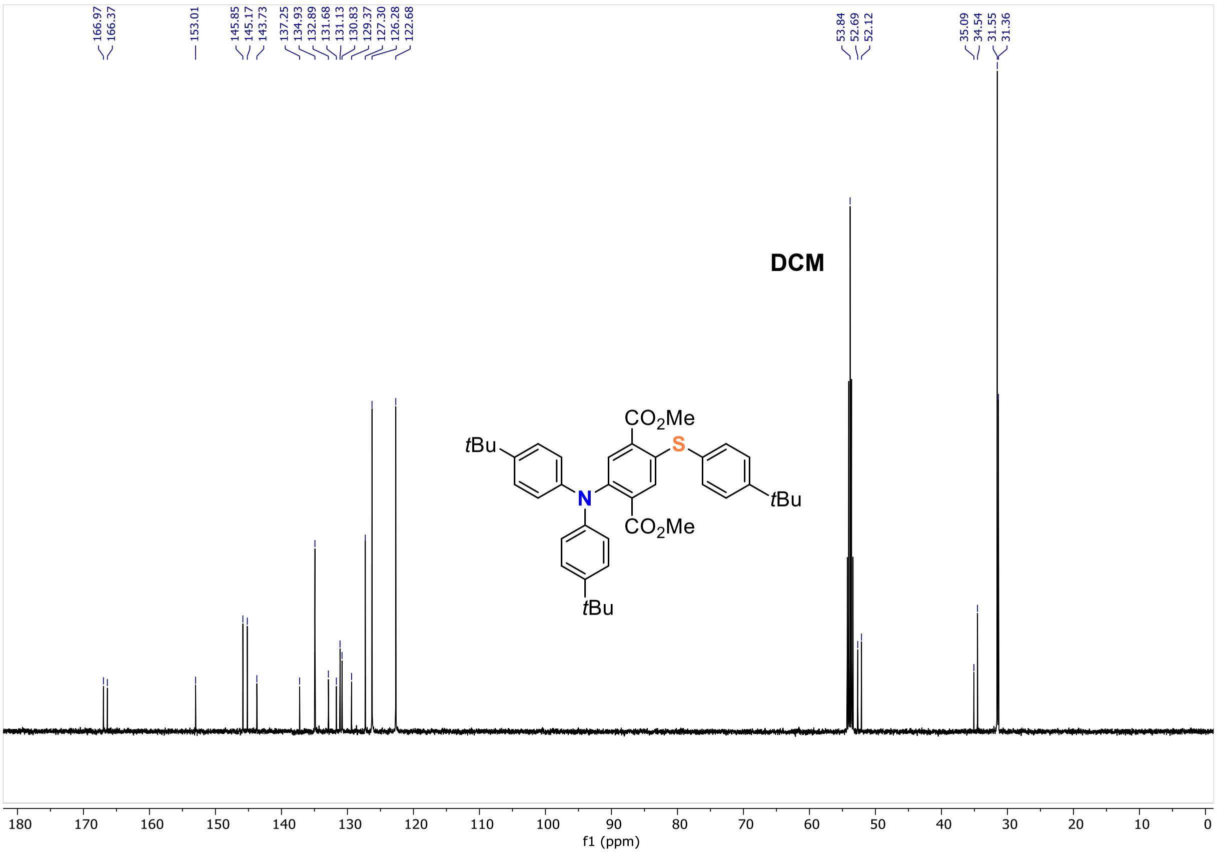

Figure S60. ^{13}C NMR spectrum of **S1** in CD_2Cl_2.

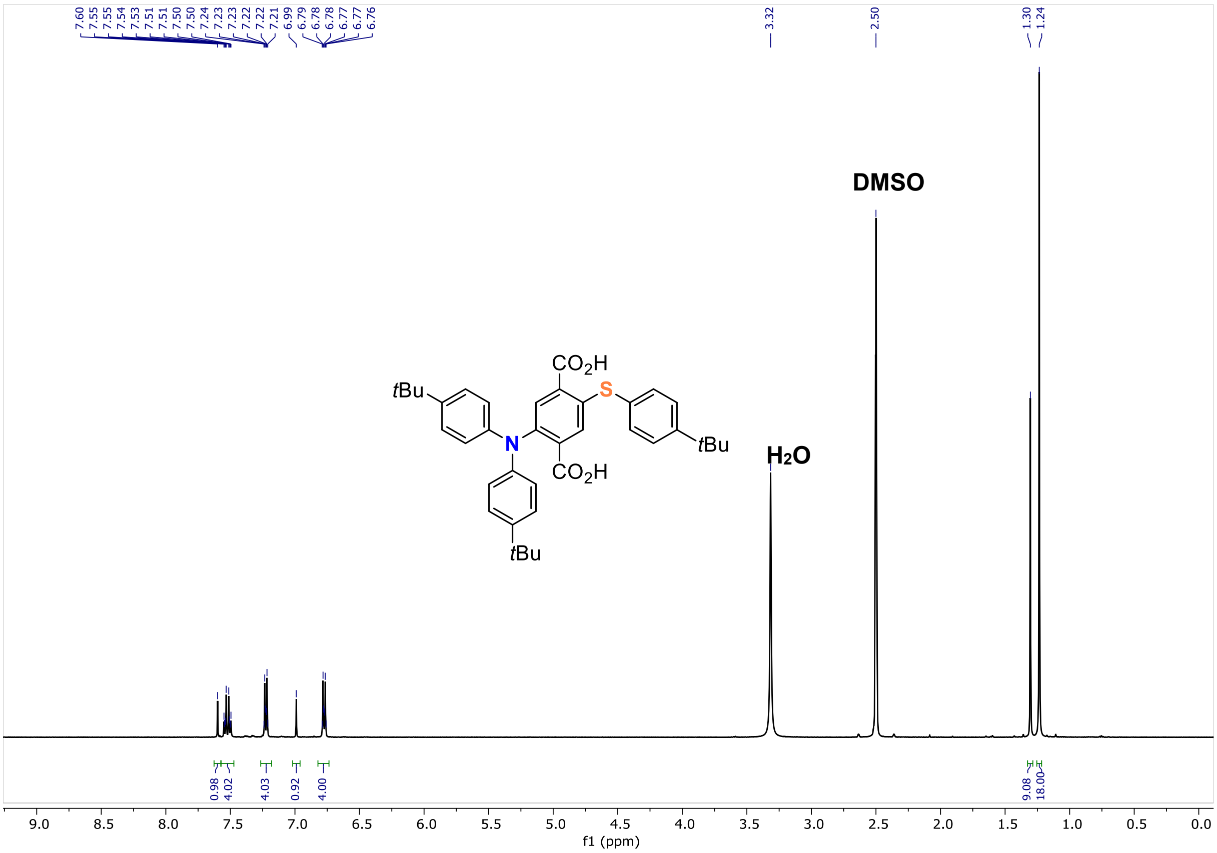

Figure S61. ^{1}H NMR spectrum of crude **5** in DMSO-d$_6$.

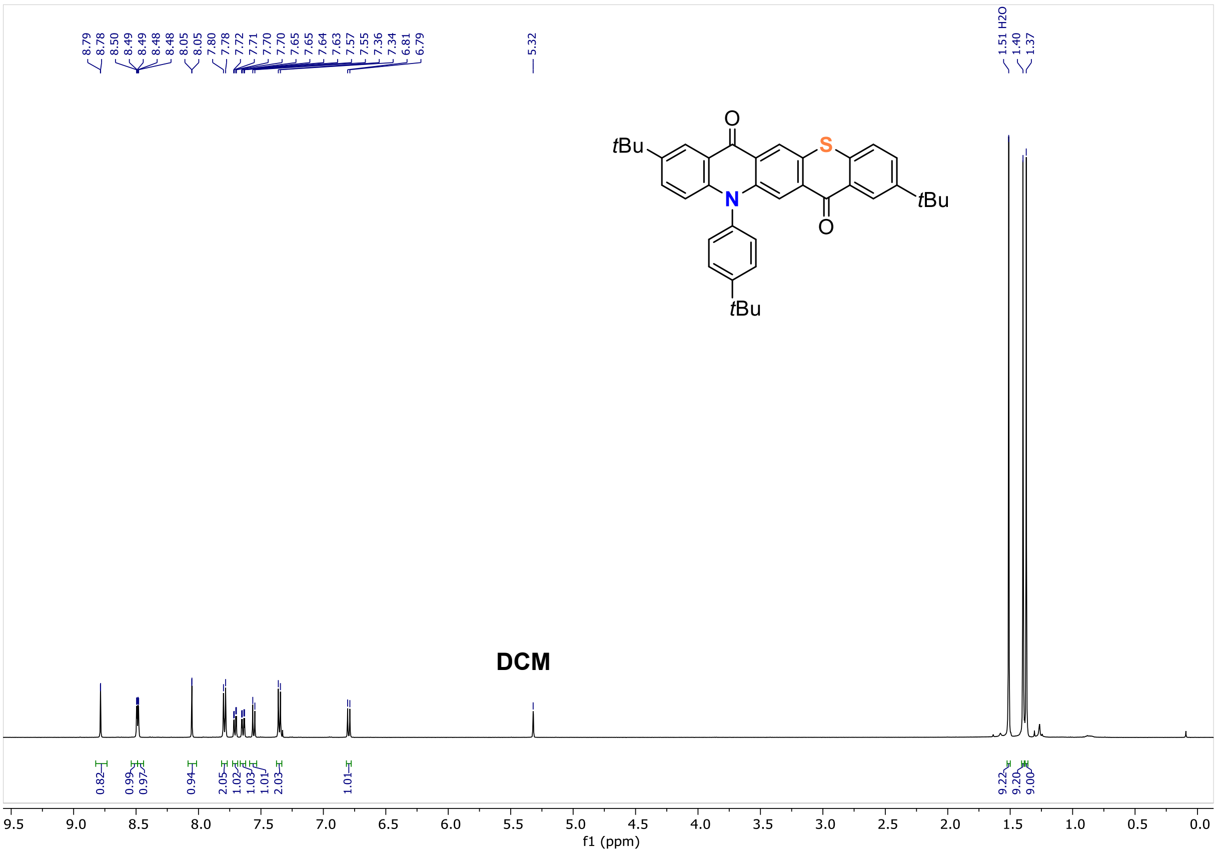

Figure S62. ^{1}H NMR spectrum of **6** in CD_2Cl_2.

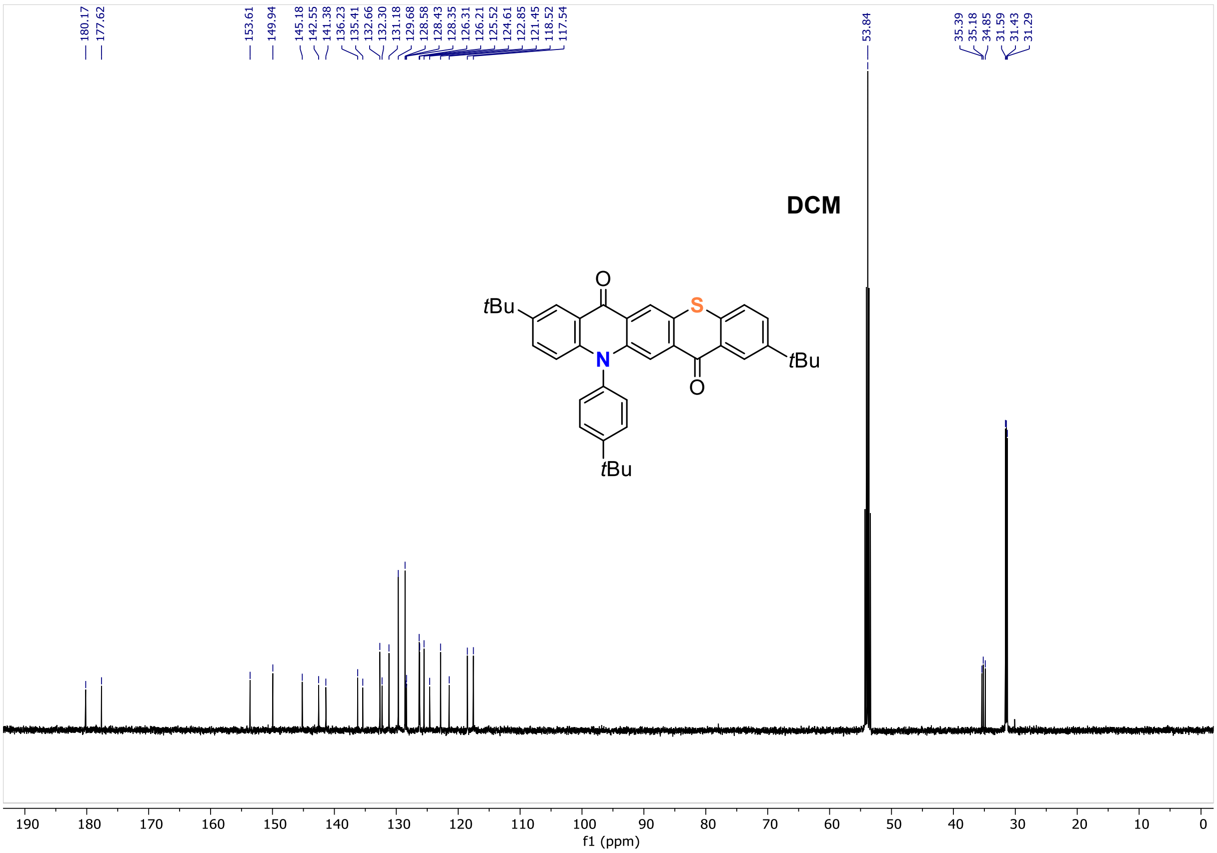

Figure S63. ^{13}C NMR spectrum of **6** in CD_2Cl_2.

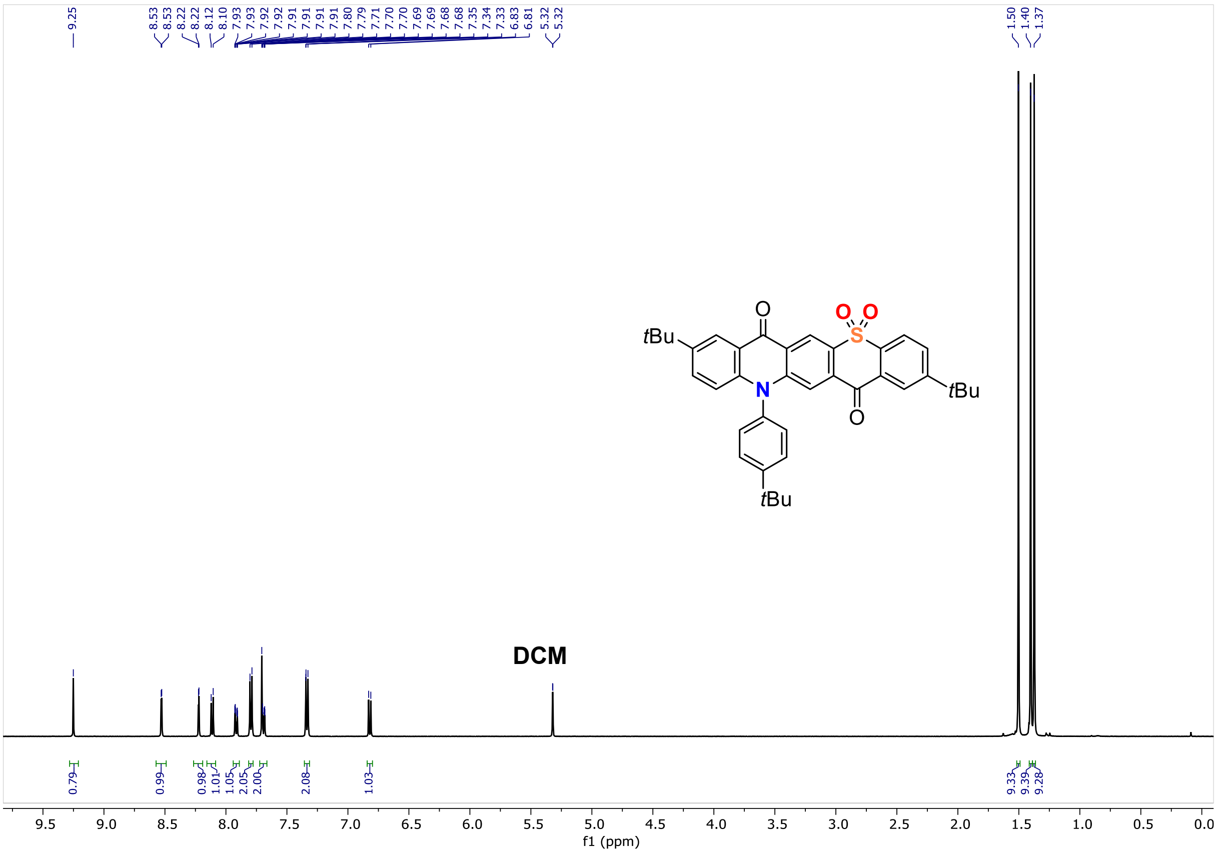

Figure S64. ^{1}H NMR spectrum of **7** in CD_2Cl_2.

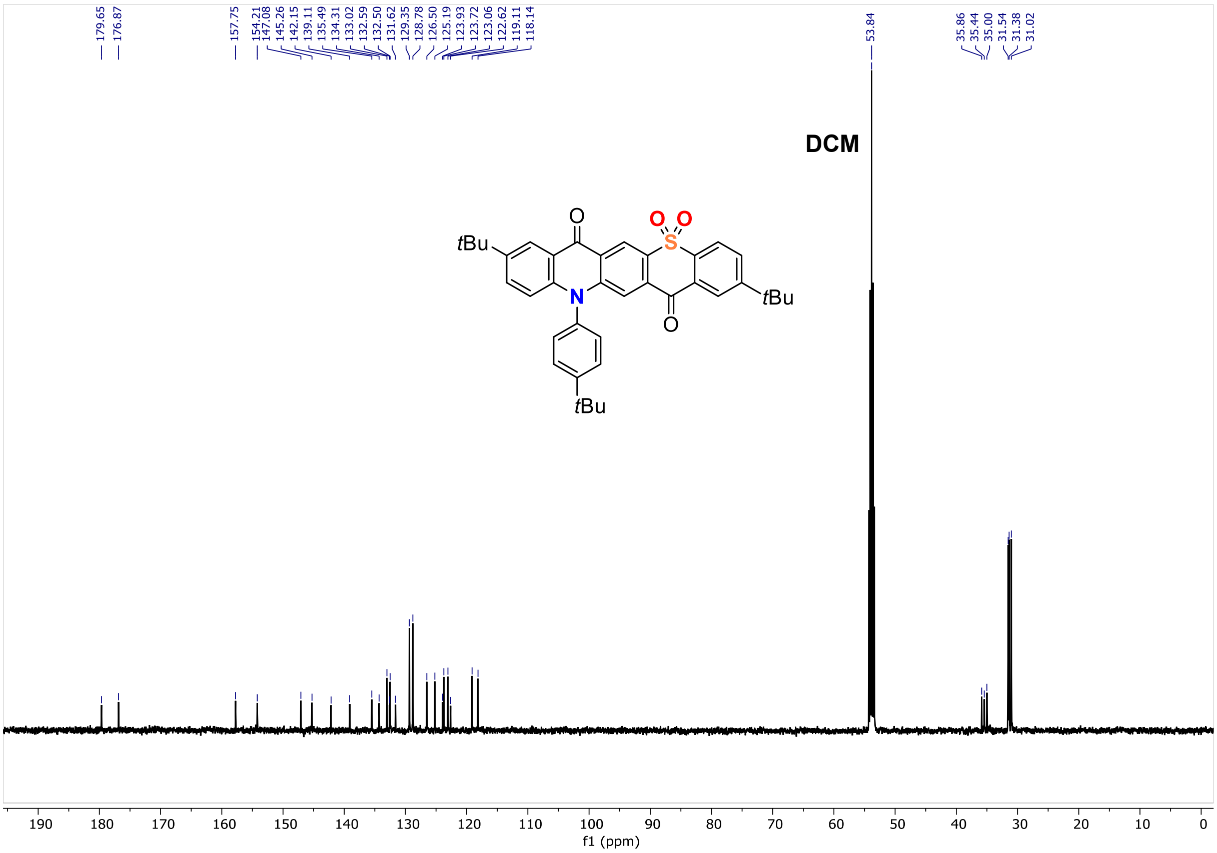

Figure S65. ^{13}C NMR spectrum of **7** in CD_2Cl_2.

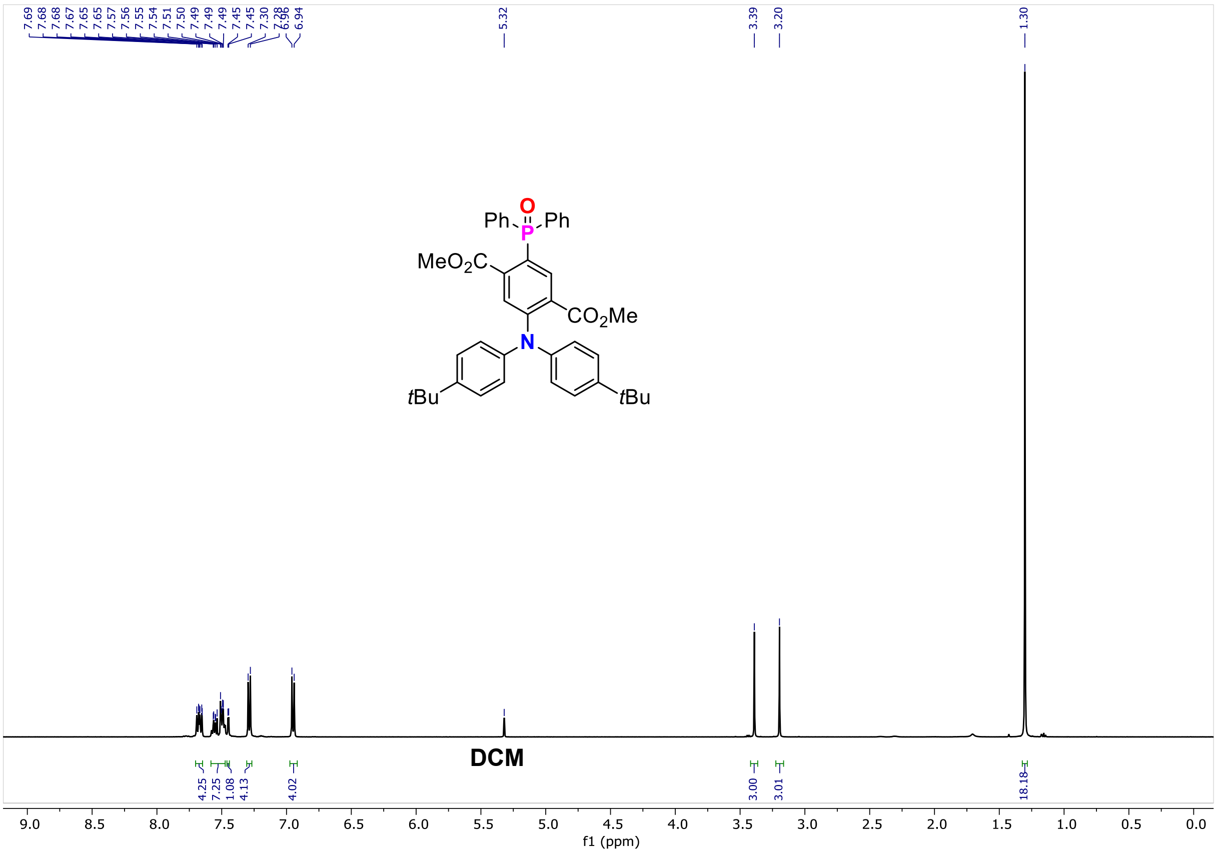

Figure S66. ^{1}H NMR spectrum of **S2** in CD_2Cl_2.

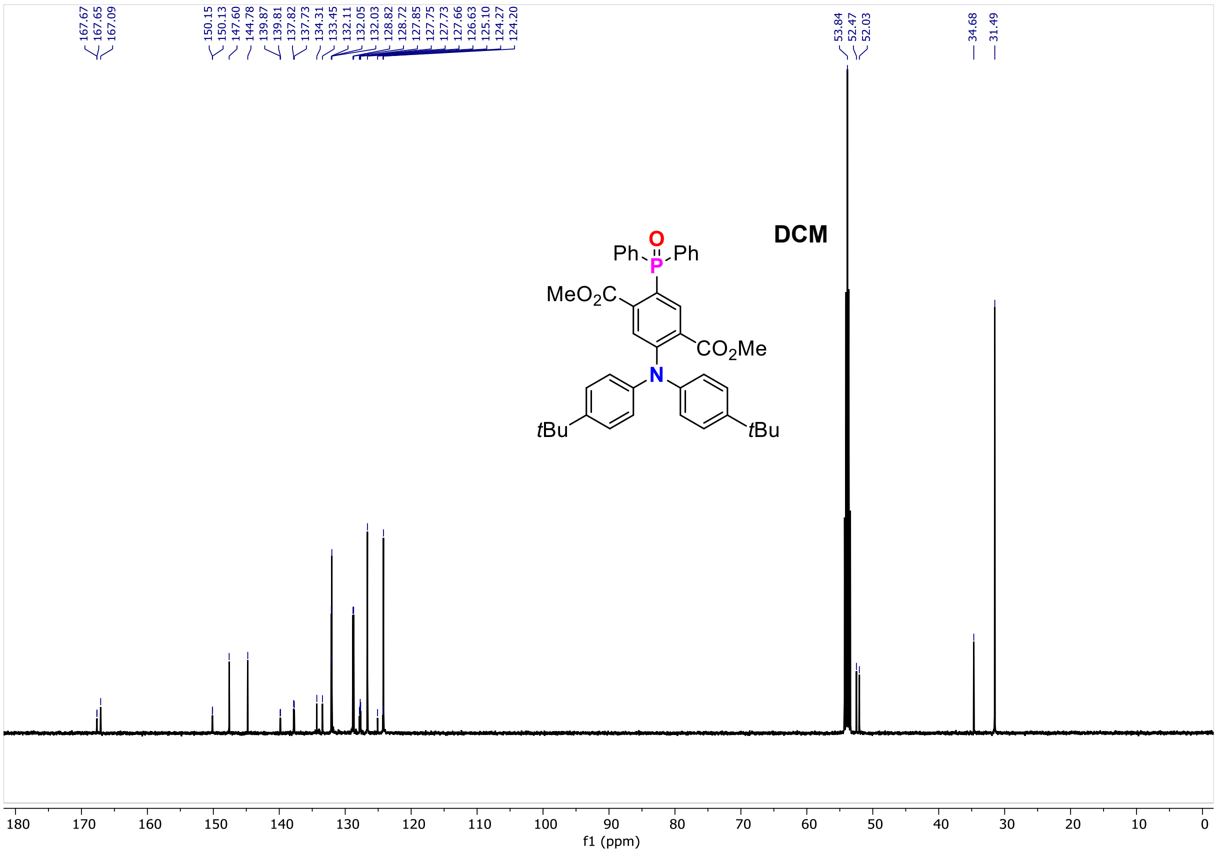

Figure S67. ^{13}C NMR spectrum of **S2** in CD_2Cl_2.

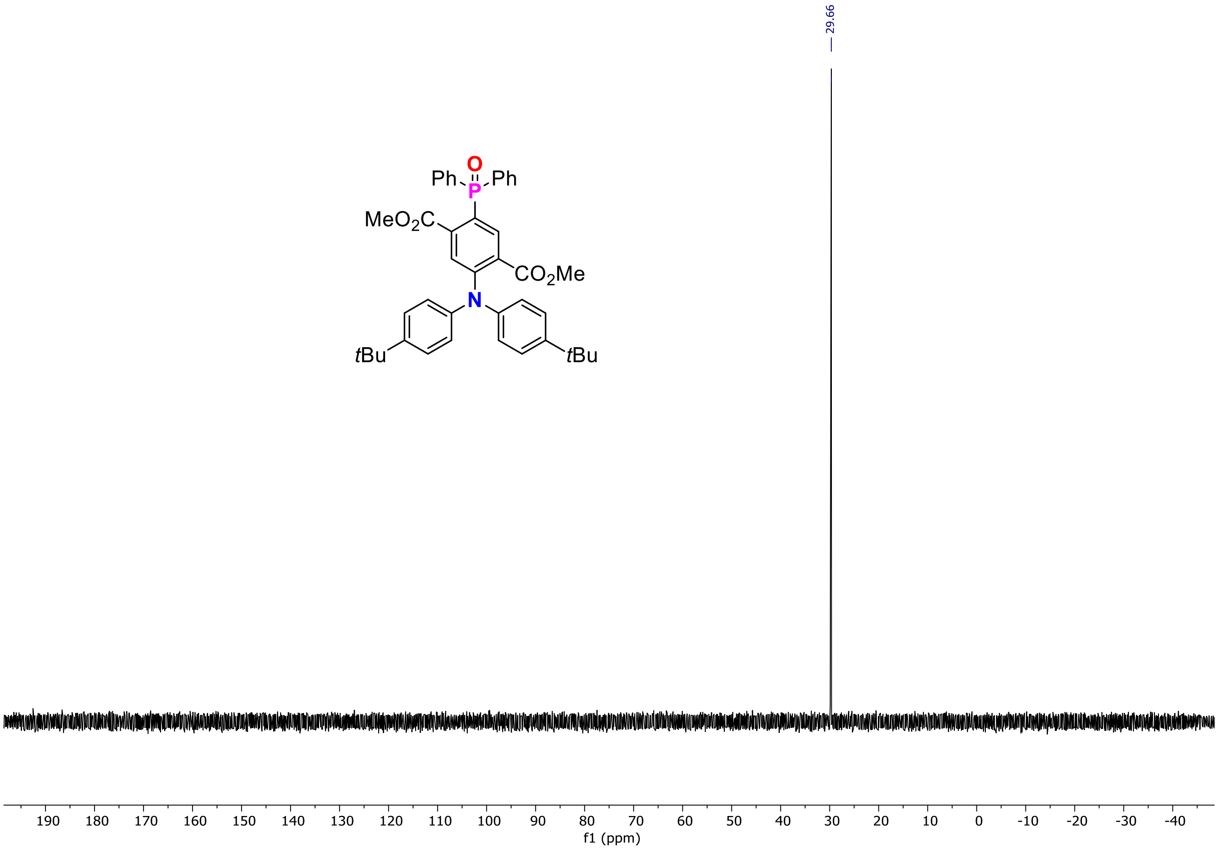

Figure S68. ^{31}P NMR spectrum of **S2** in CD_2Cl_2.

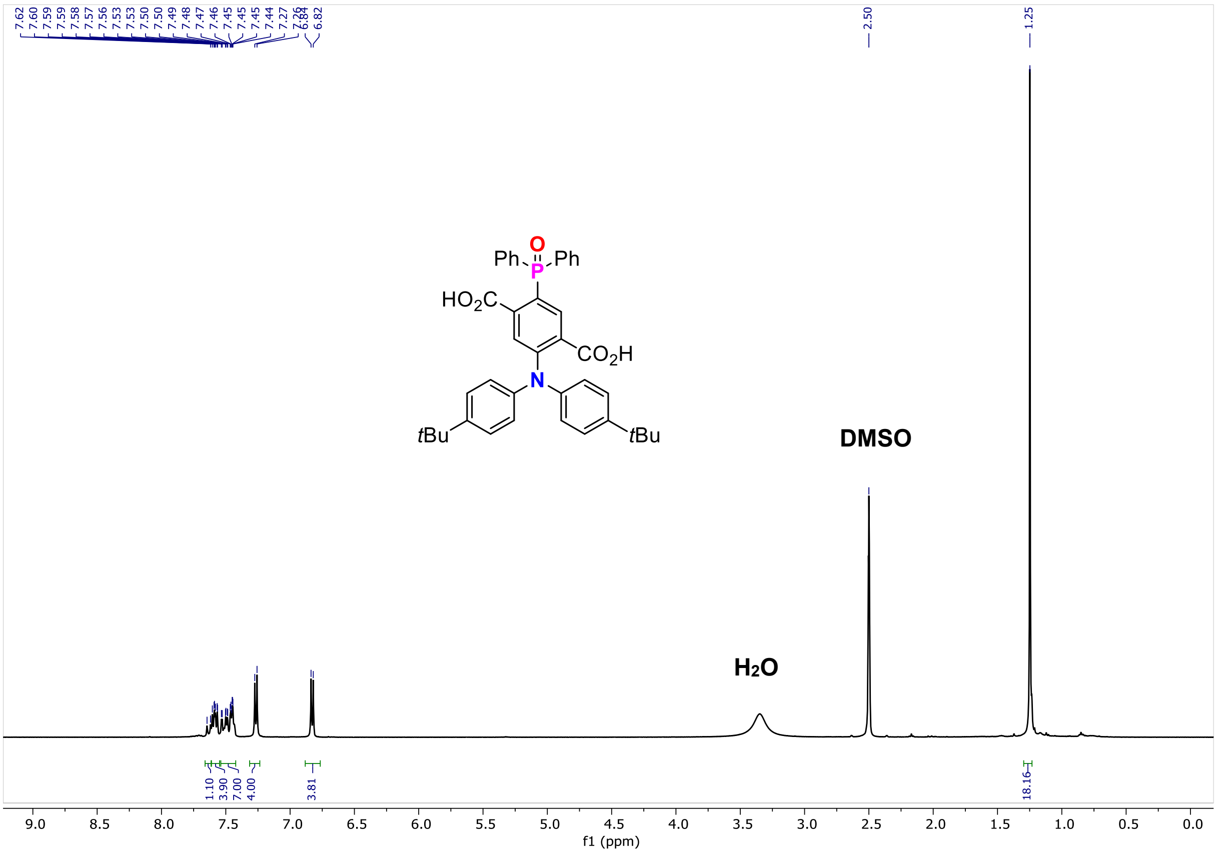

Figure S69. ^{1}H NMR spectrum of crude **8** in DMSO-d$_6$.

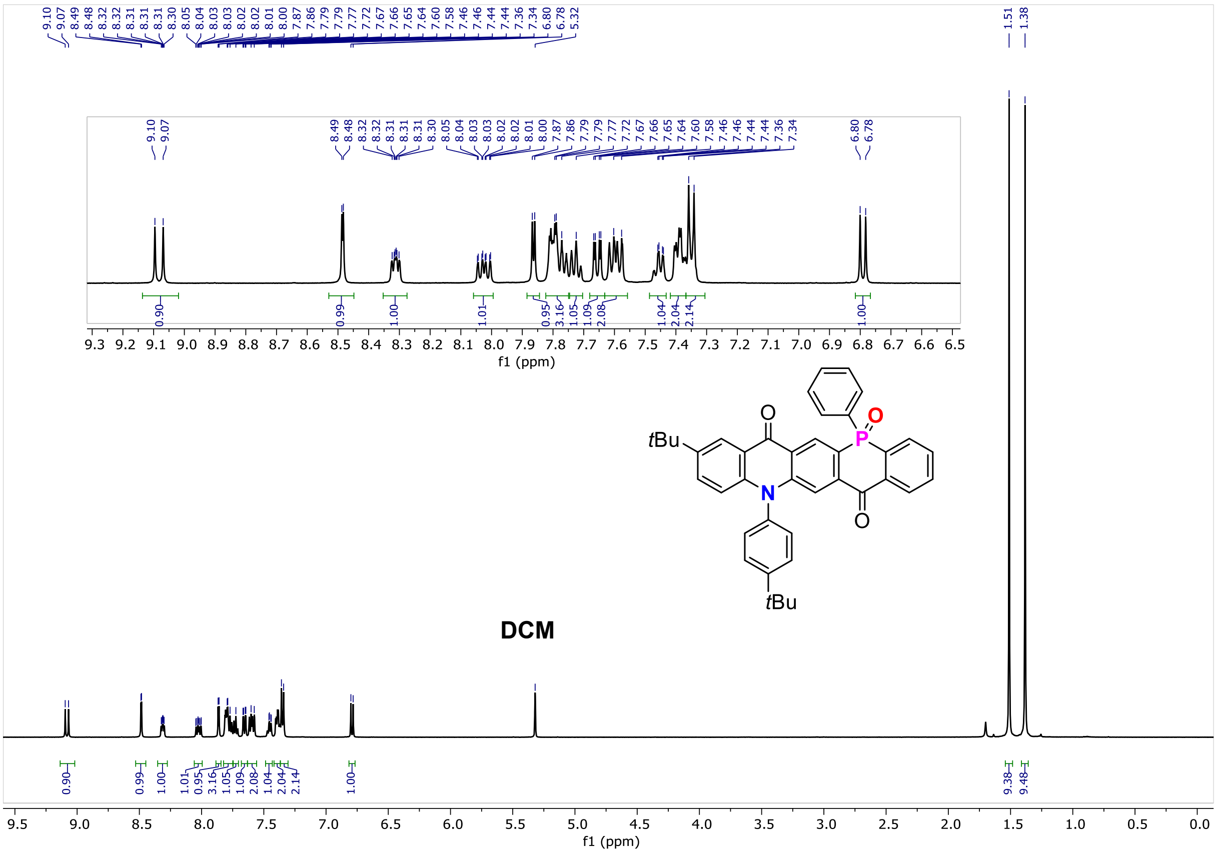

Figure S70. ^{1}H NMR spectrum of **10** in CD_2Cl_2.

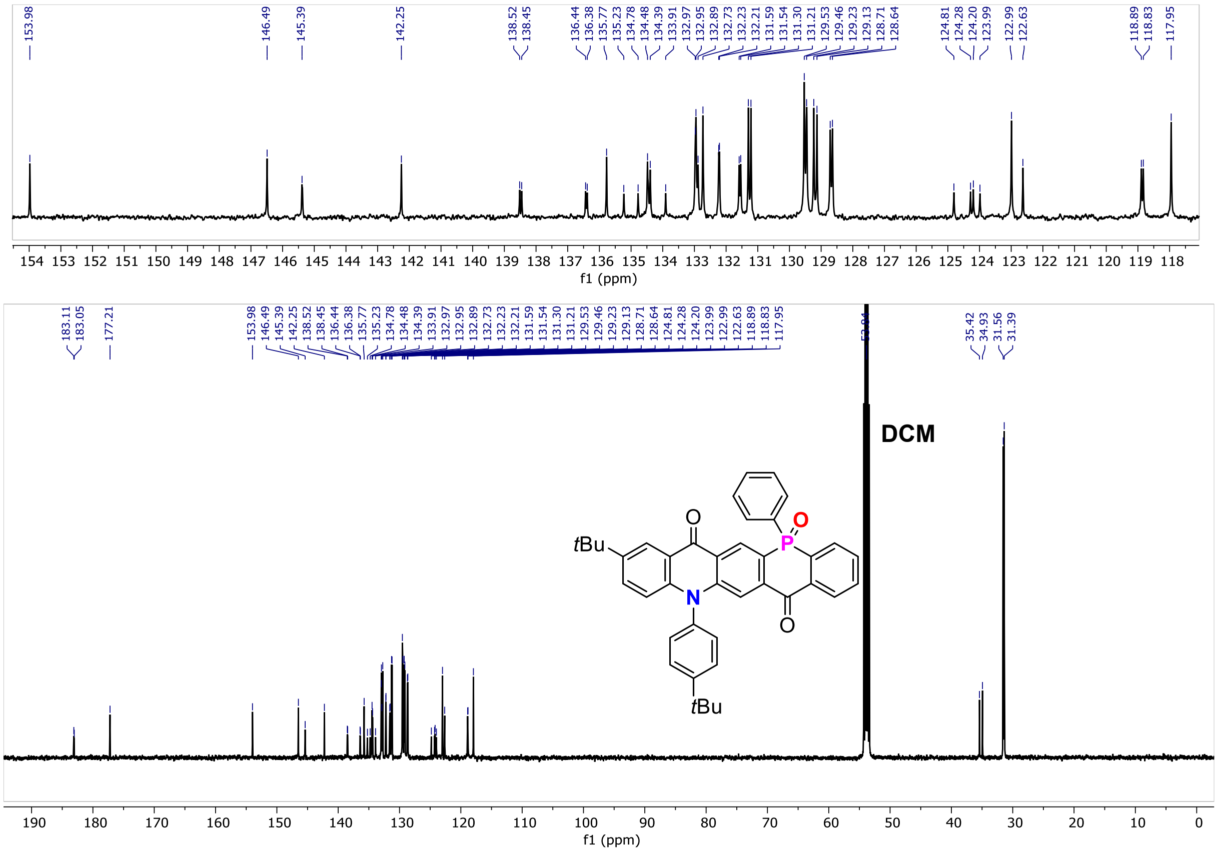

Figure S71. ^{13}C NMR spectrum of **10** in CD_2Cl_2.

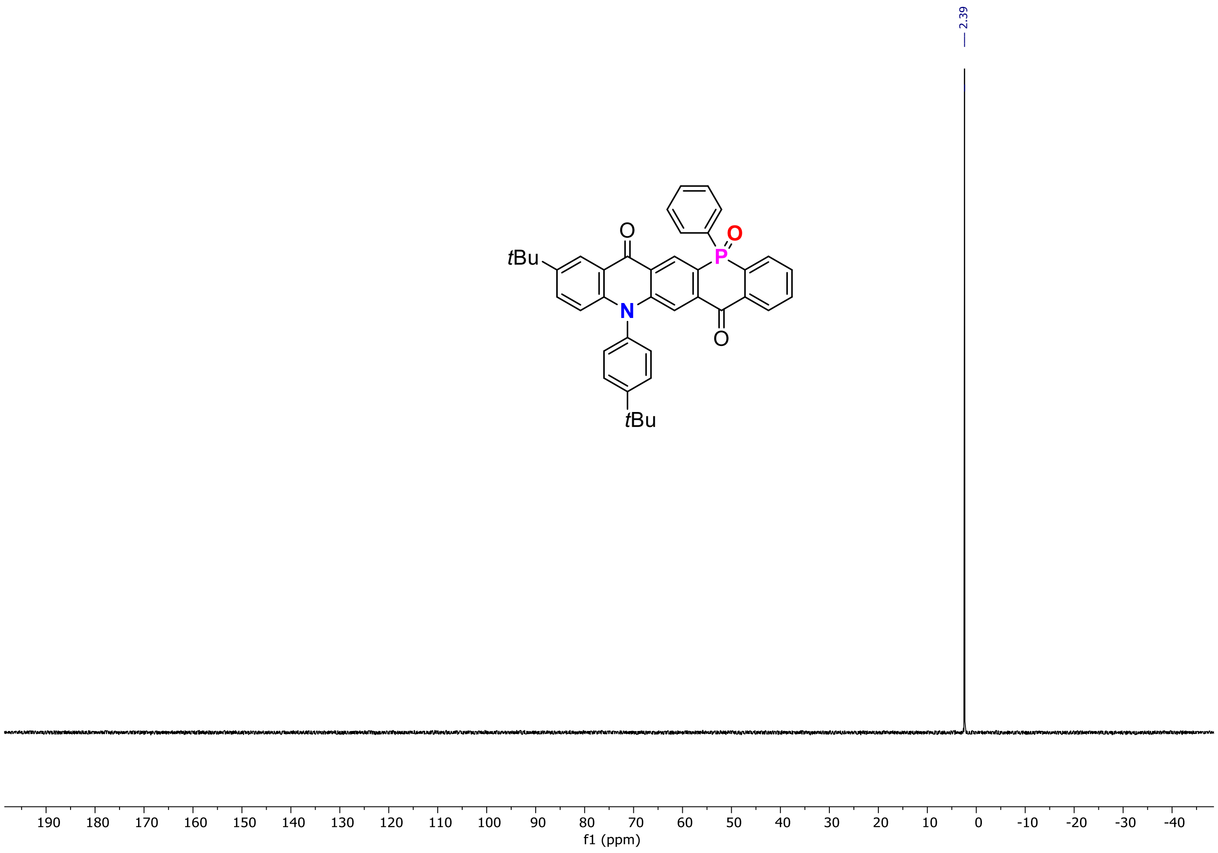

Figure S72. ^{31}P NMR spectrum of **10** in CD_2Cl_2.

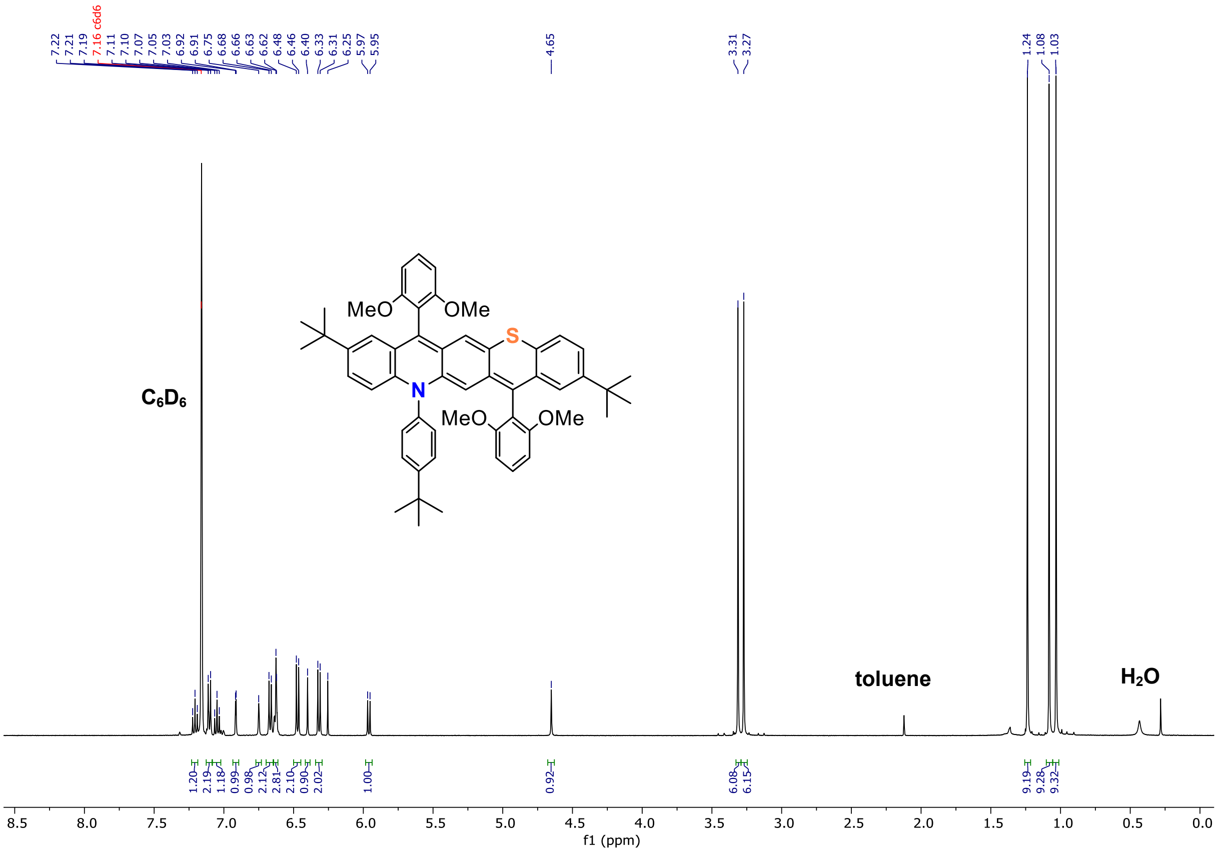

Figure S73. ^{1}H NMR spectrum of **SN-PA-a** in C_6D_6 at 323 K.

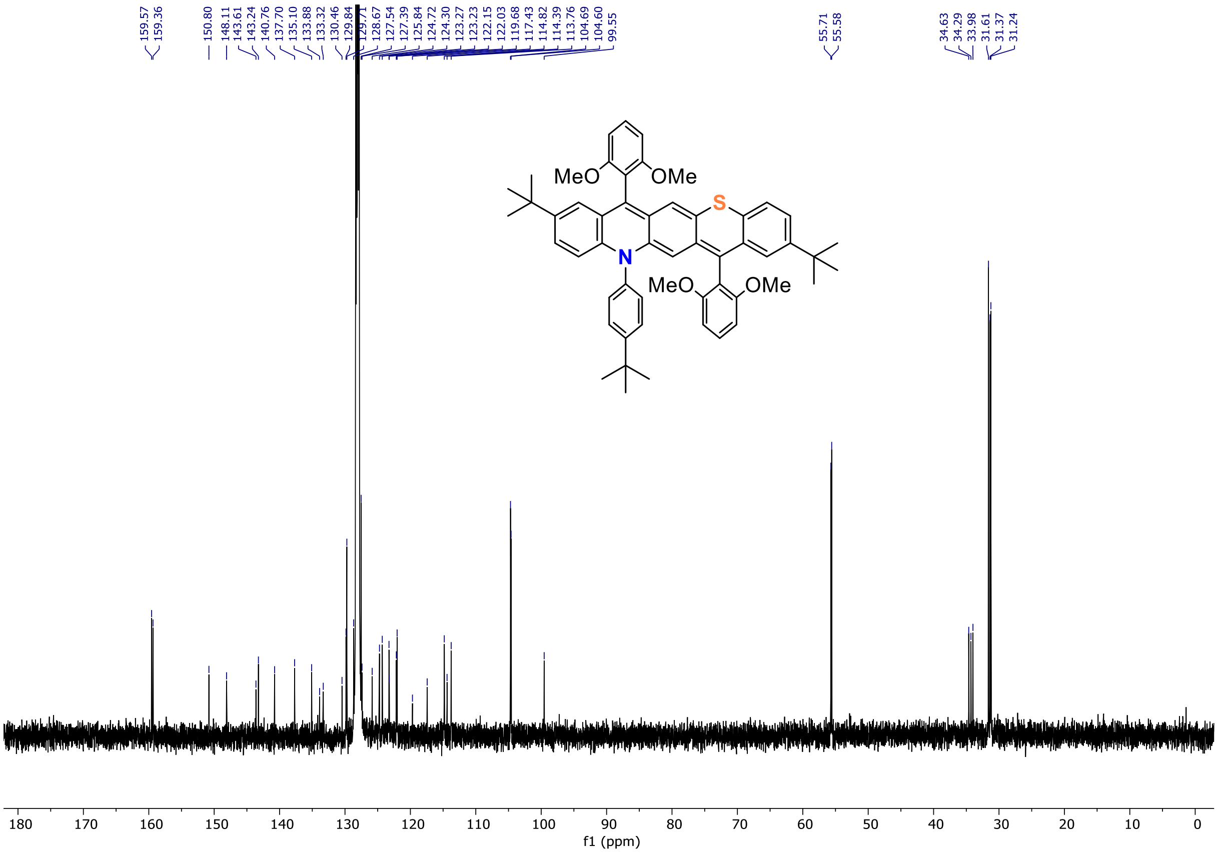

Figure S74. ^{13}C NMR spectrum of **SN-PA-a** in C_6D_6 at 323 K.

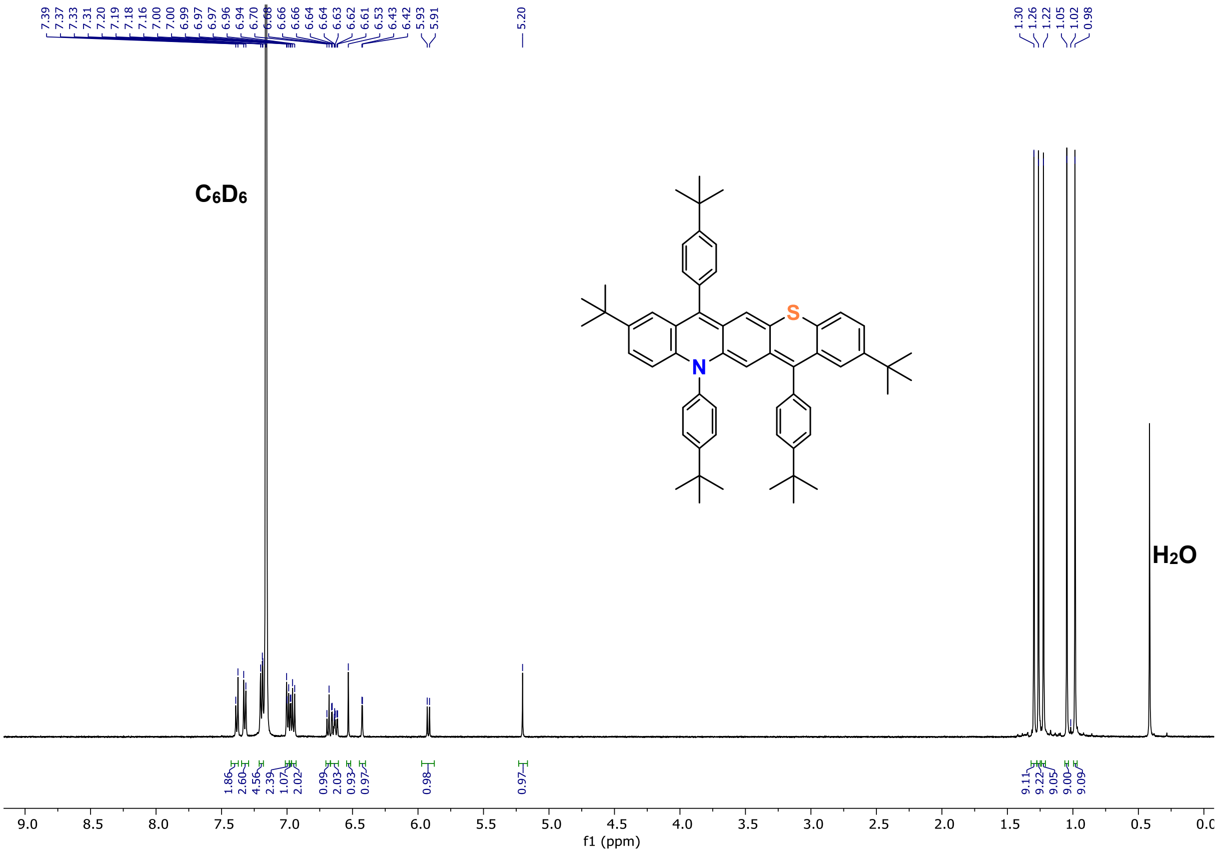

Figure S75. ^{1}H NMR spectrum of **SN-PA-b** in C_6D_6 at 323 K.

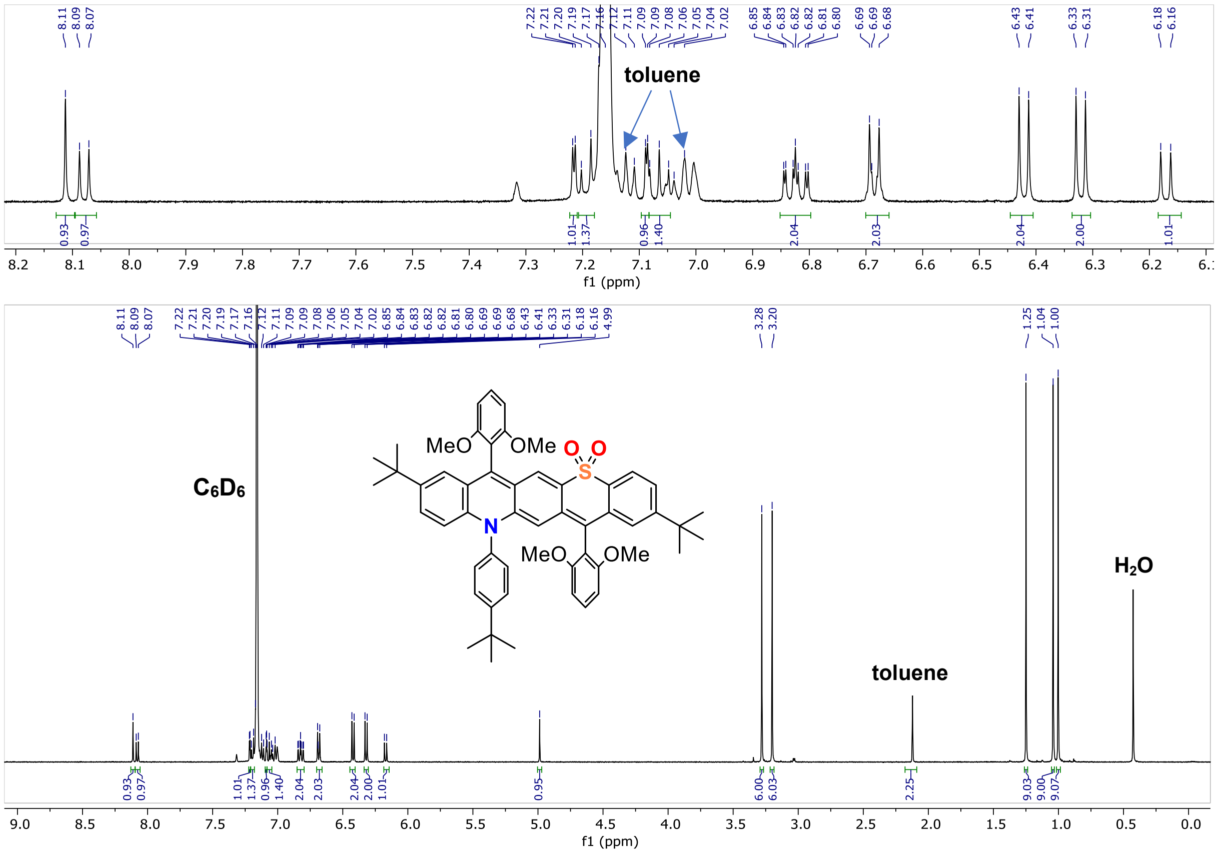

Figure S76. ^{1}H NMR spectrum of **SO$_2$N-PA-a** in C_6D_6 at 323 K.

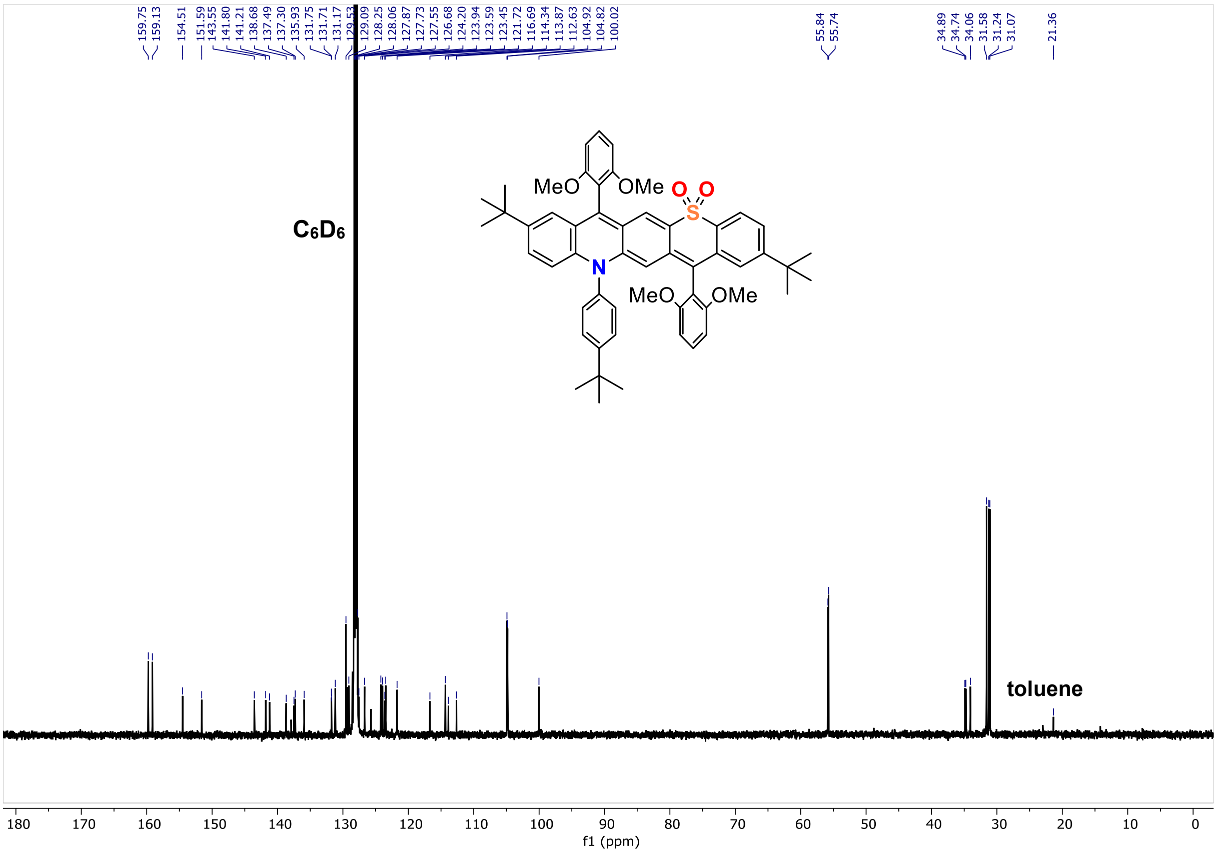

Figure S77. ^{13}C NMR spectrum of **SO_2N-PA-a** in C_6D_6 at 323 K.

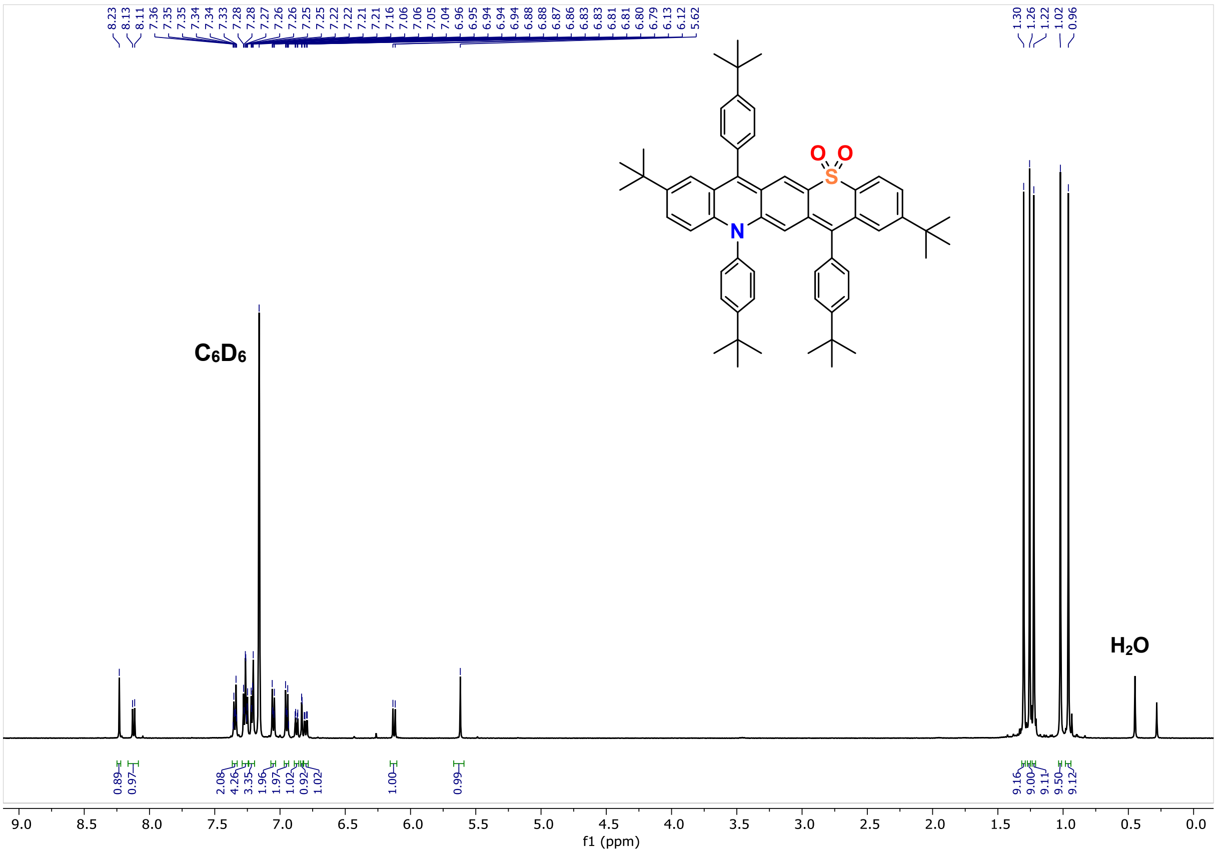

Figure S78. ^{1}H NMR spectrum of **SO_2N-PA-b** in C_6D_6 at 323 K.

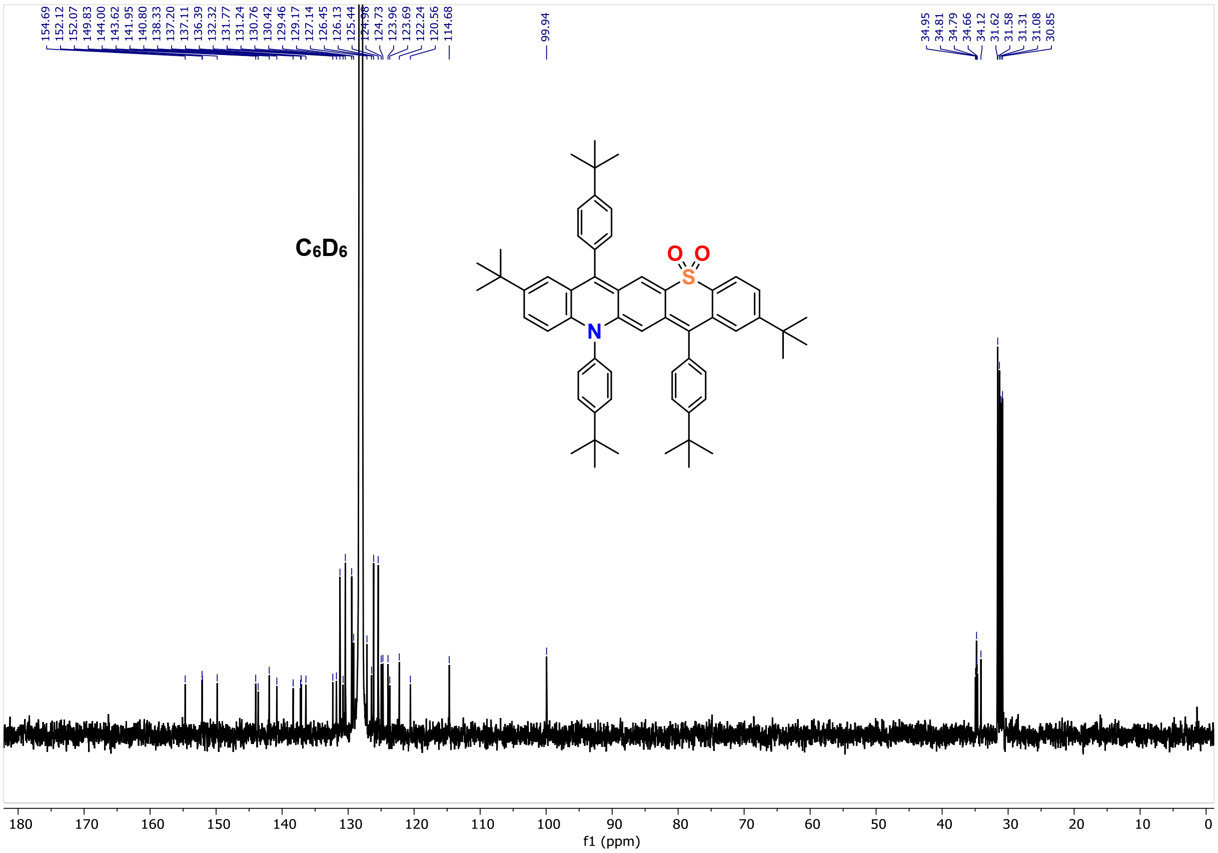

Figure S79. ^{13}C NMR spectrum of **SO_2N-PA-b** in C_6D_6 at 323 K.

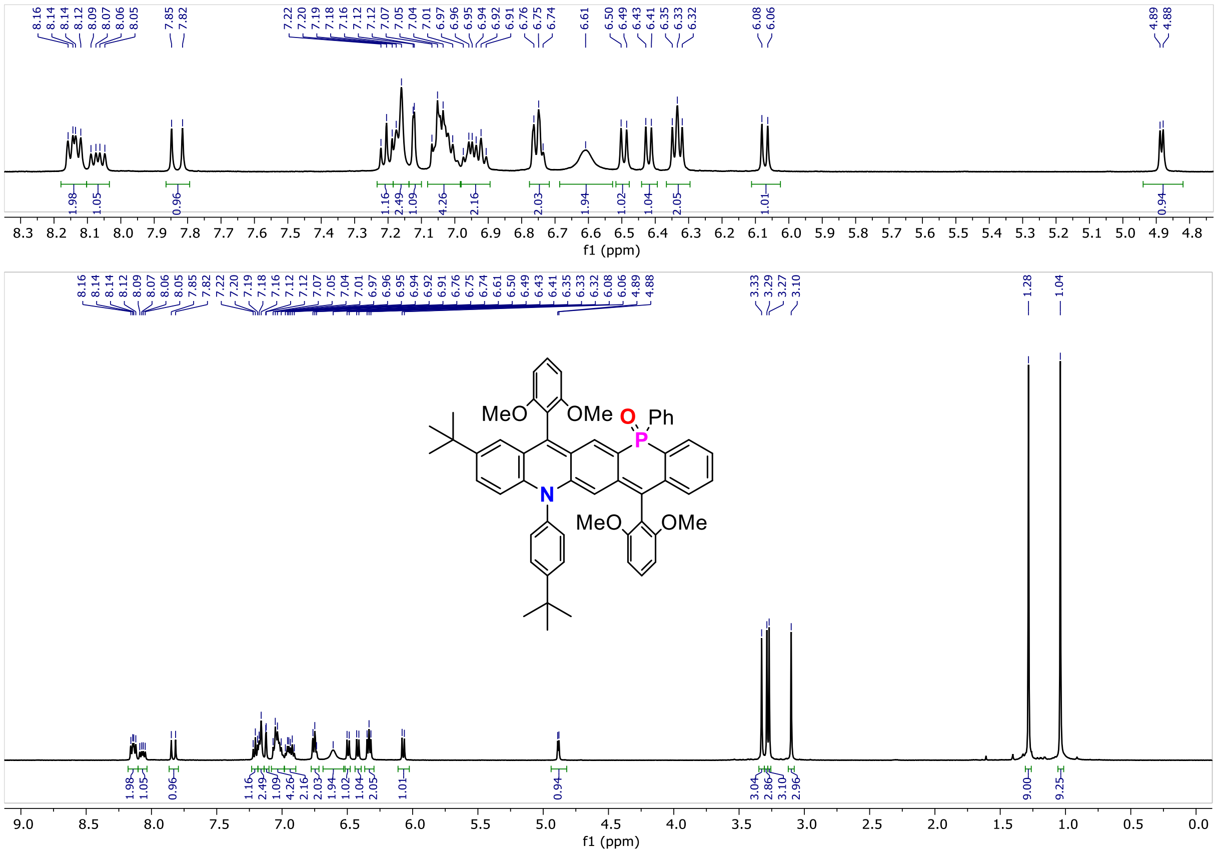

Figure S80. ^{1}H NMR spectrum of **PON-PA** in C_6D_6 at 323 K.

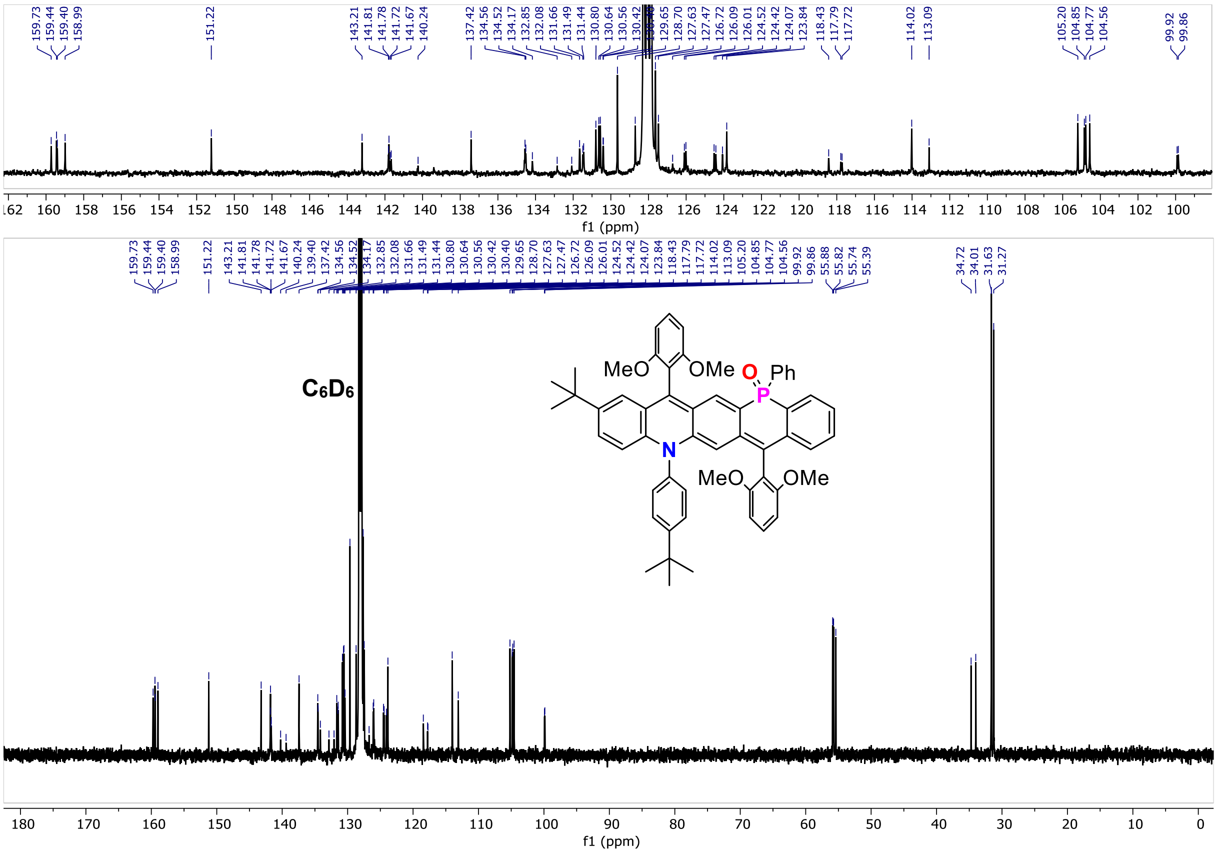

Figure S81. ^{13}C NMR spectrum of **PON-PA** in C_6D_6 at 323 K.

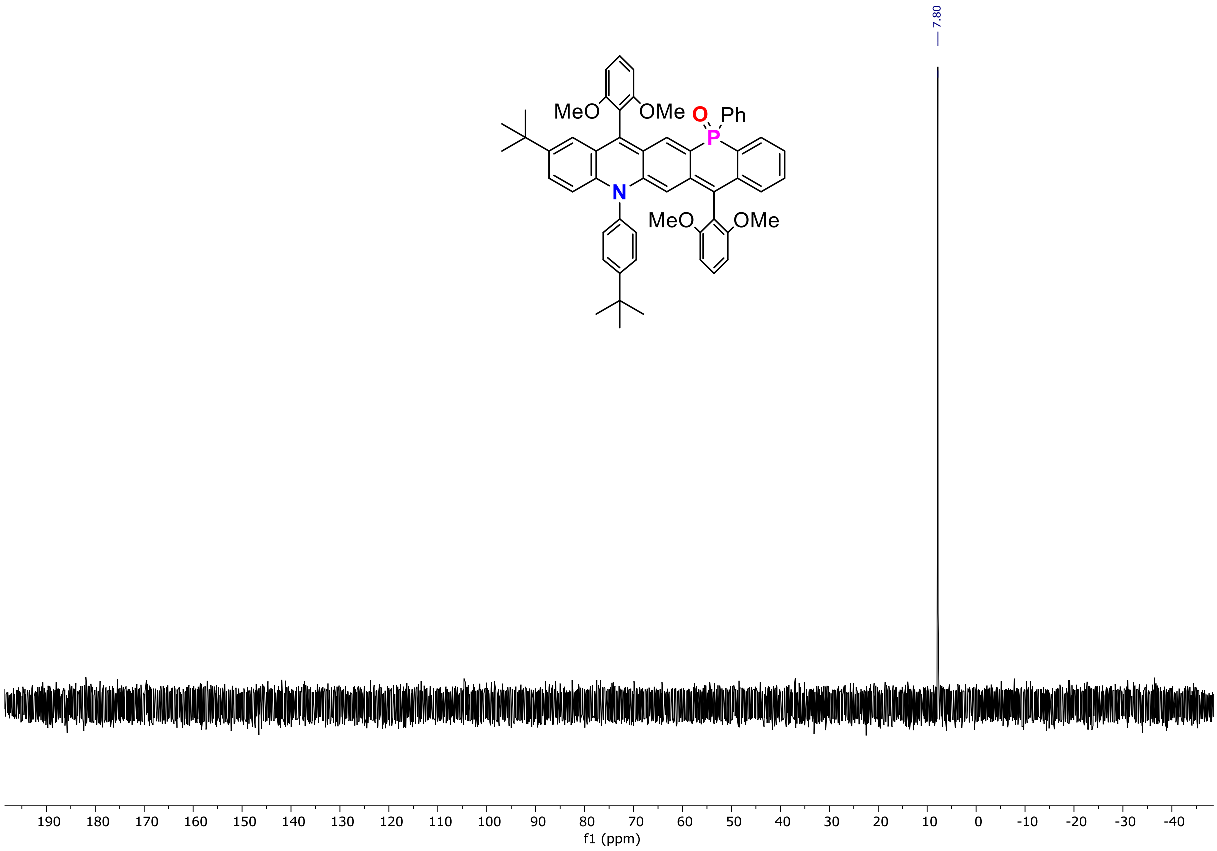

Figure S82. ^{31}P NMR spectrum of **PON-PA** in C_6D_6.

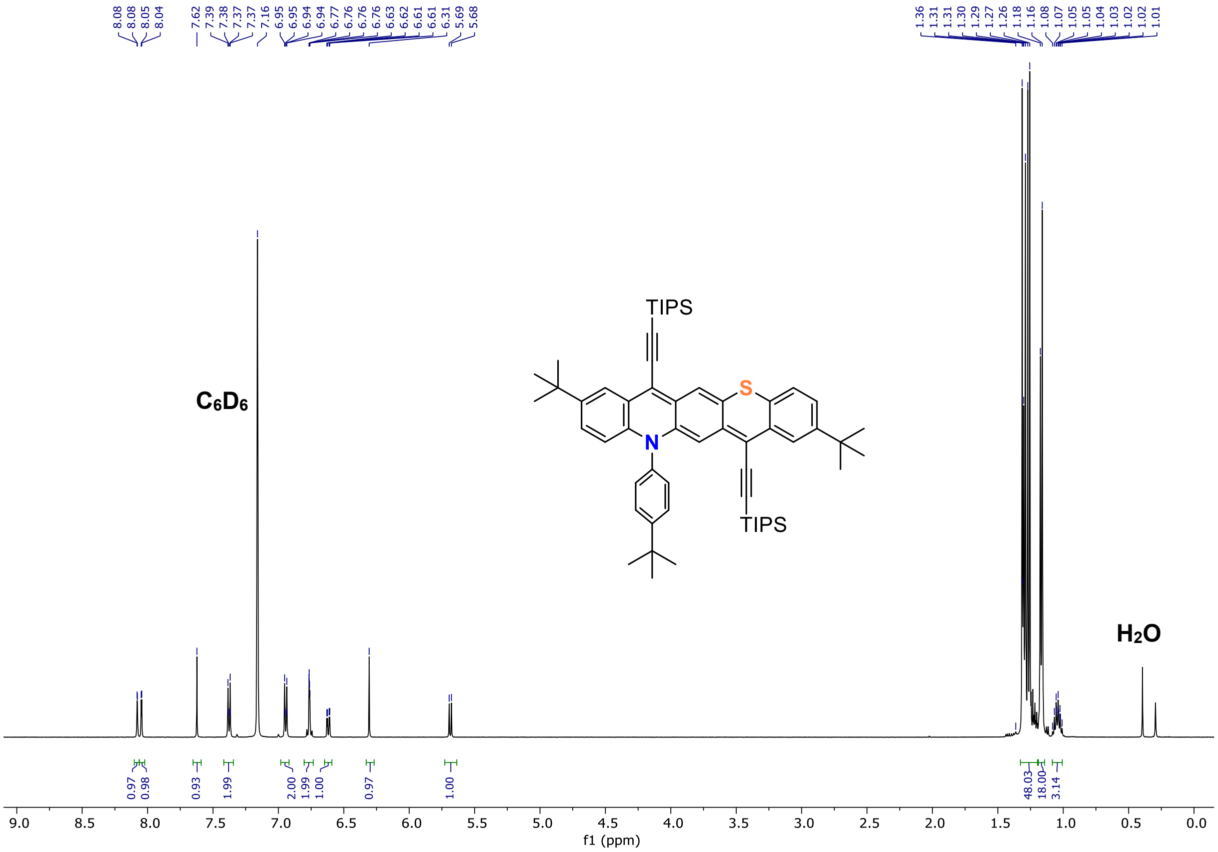

Figure S83. ^{1}H NMR spectrum of **SN-PA-c** in C_6D_6 at 323 K.

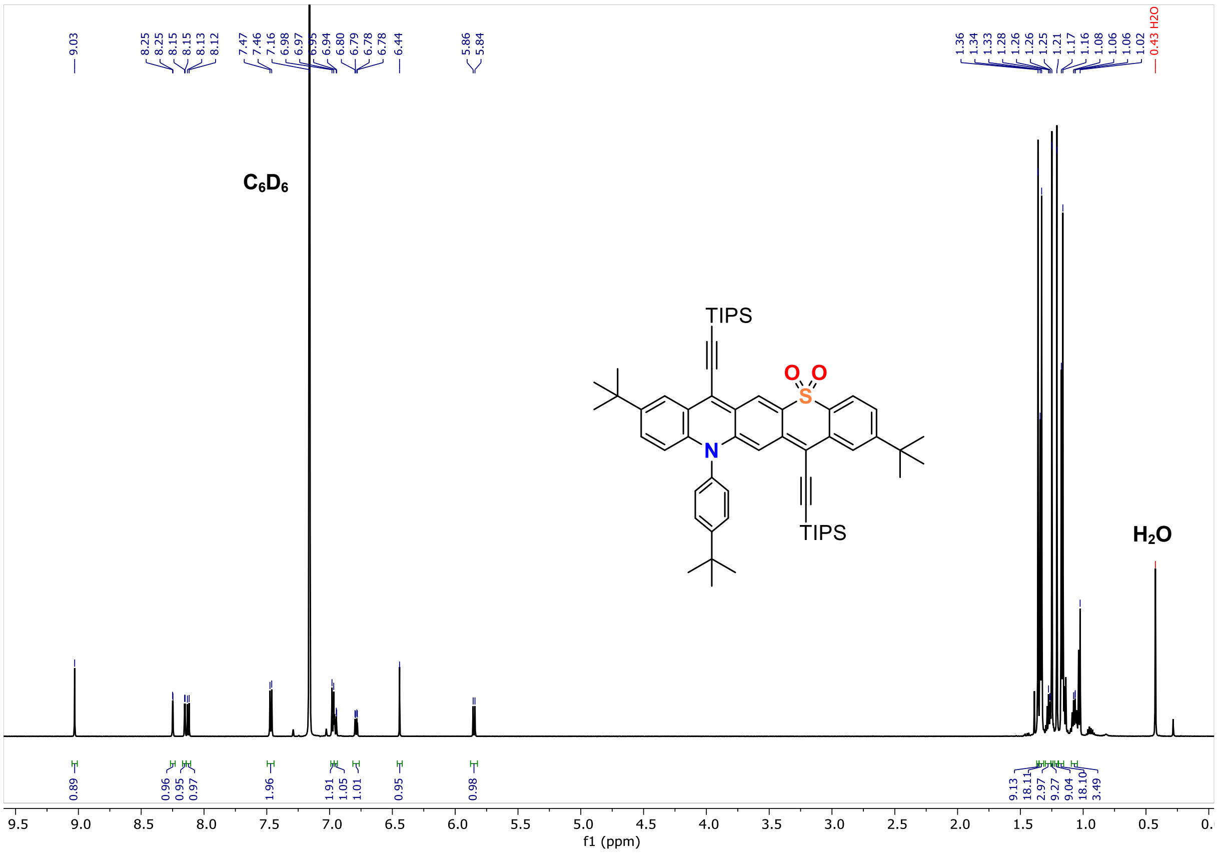

Figure S84. ^{1}H NMR spectrum of **SO_2N-PA-c** in C_6D_6 at 323 K.

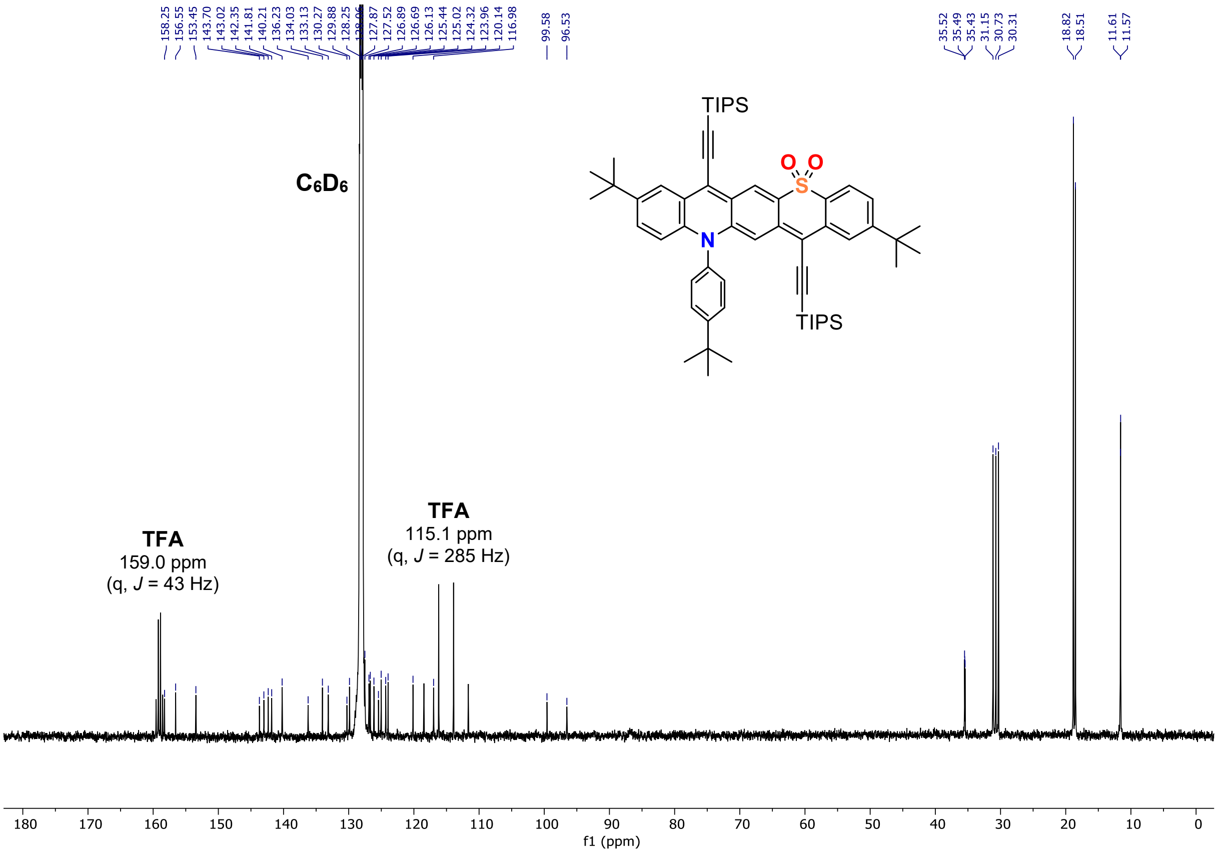

Figure S85. ^{13}C NMR spectrum of **SO$_2$N-PA-c** in C_6D_6 with TFA.

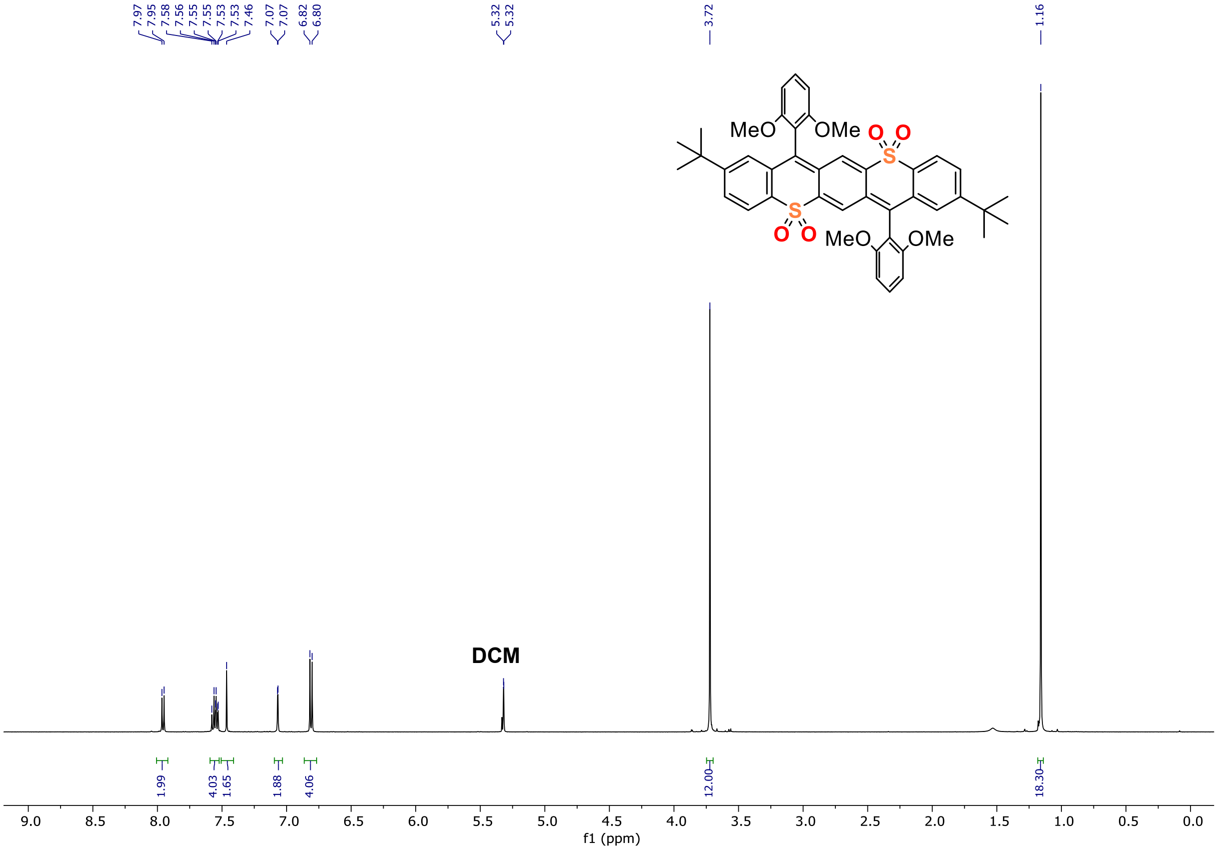

Figure S86. ^{1}H NMR spectrum of **diSO$_2$-PA-a** in CD_2Cl_2.

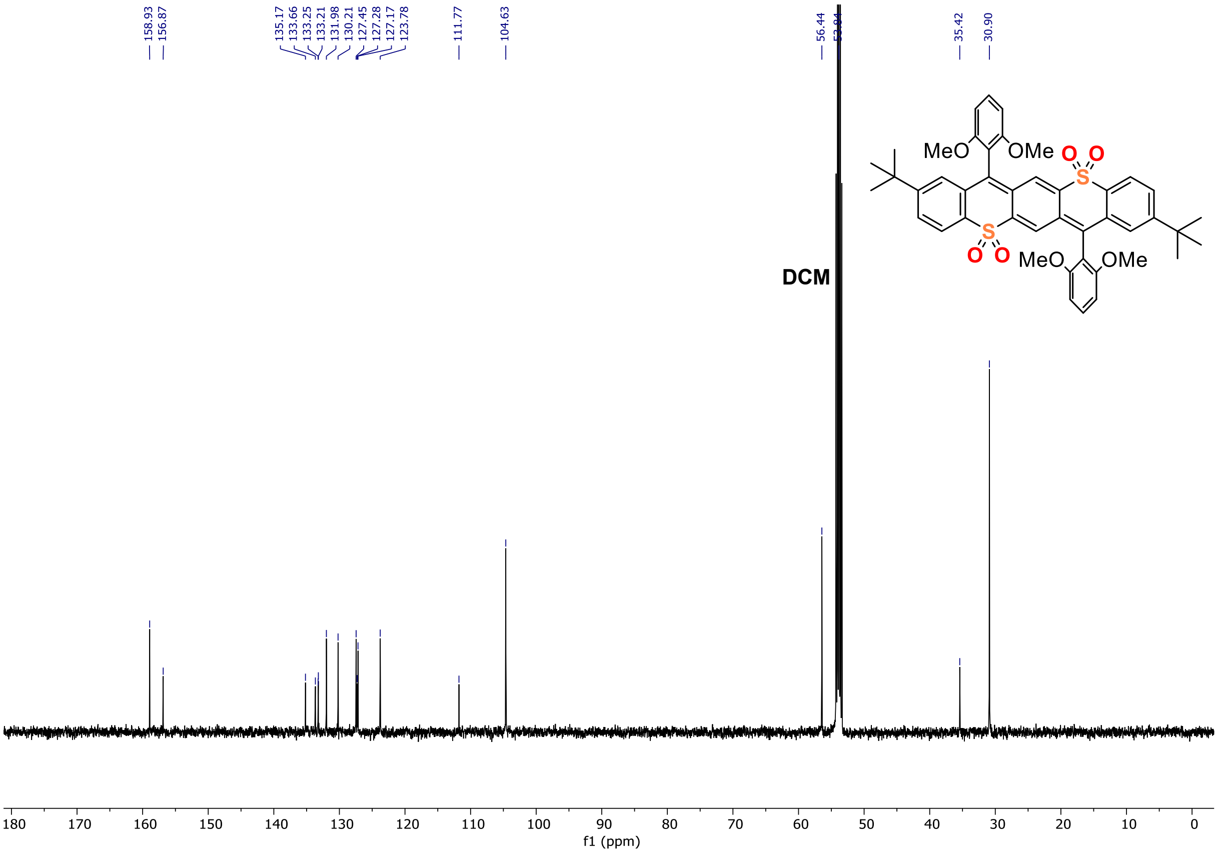

Figure S87. ^{13}C NMR spectrum of **diSO$_2$-PA-a** in CD_2Cl_2.

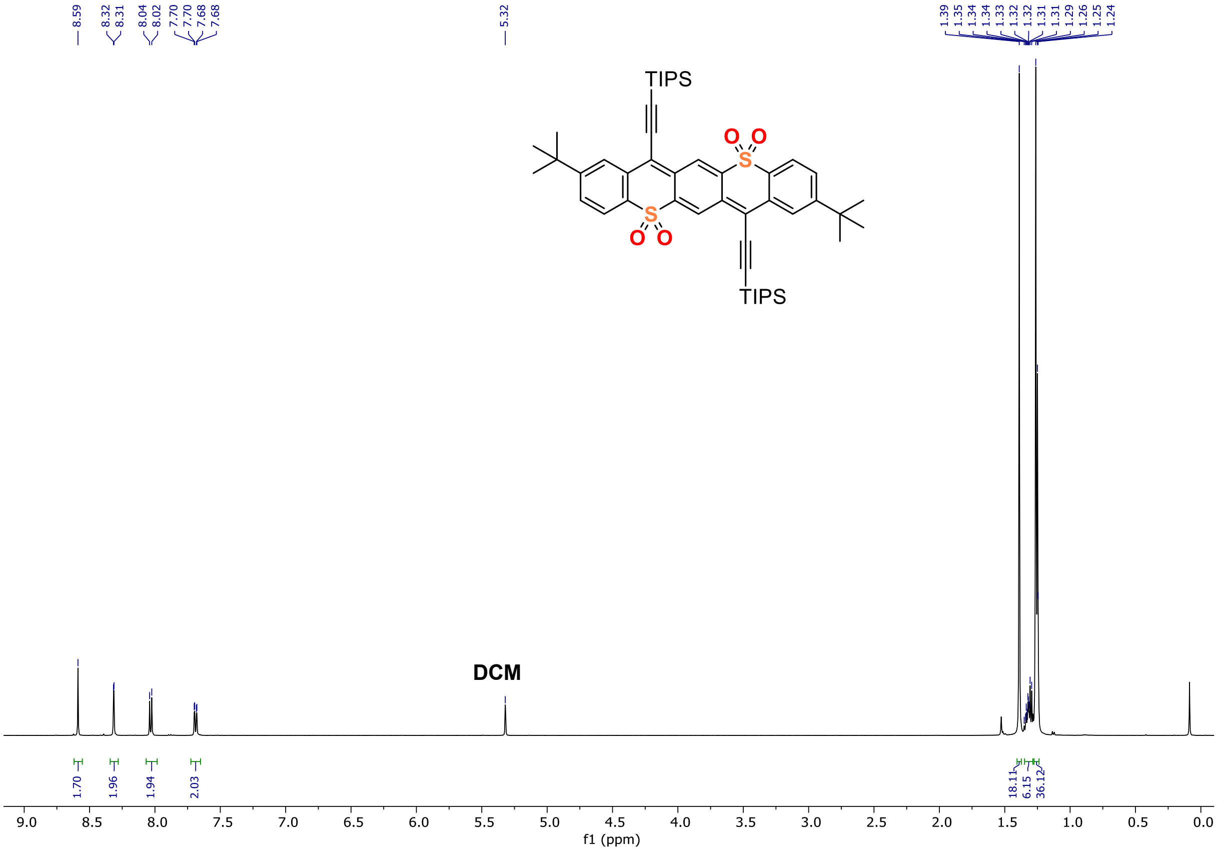

Figure S88. ^{1}H NMR spectrum of **diSO$_2$-PA-c** in CD_2Cl_2.

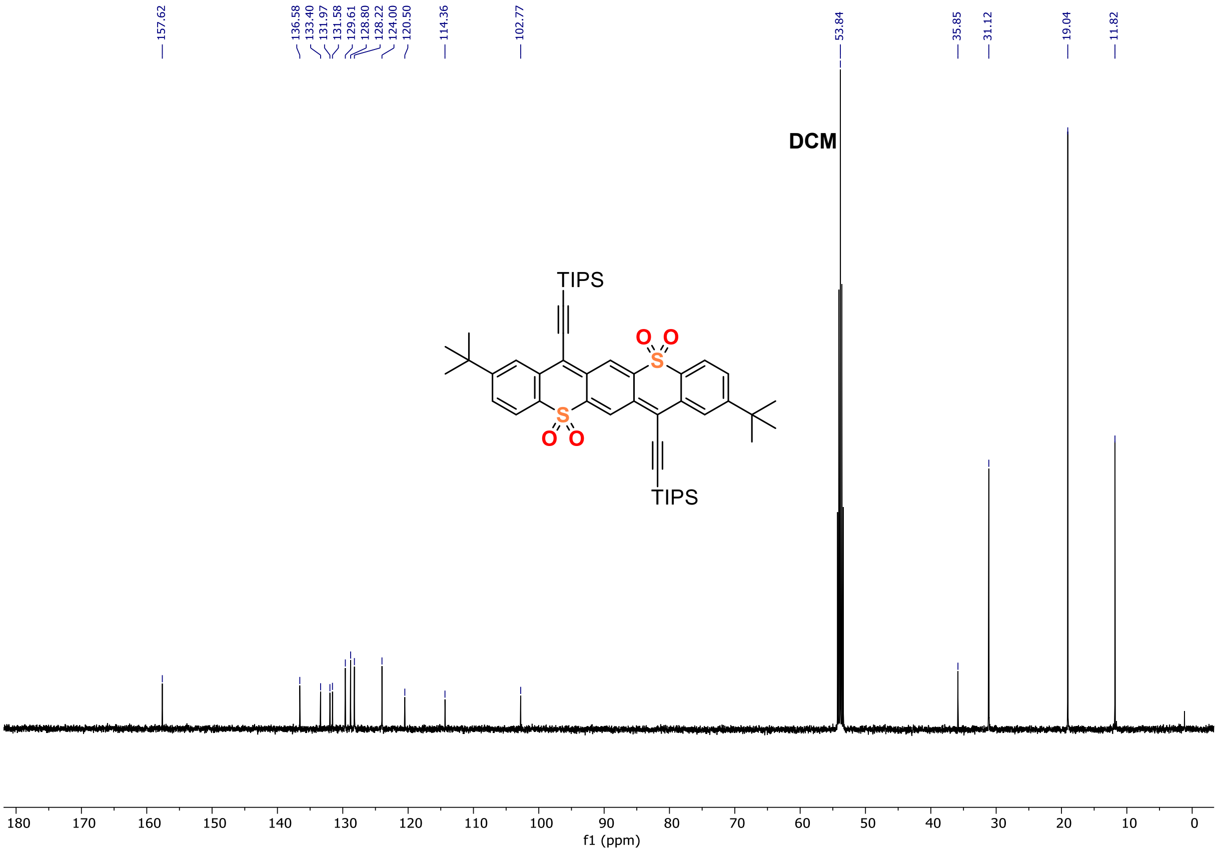

Figure S89. ^{13}C NMR spectrum of **diSO$_2$-PA-c** in CD_2Cl_2.

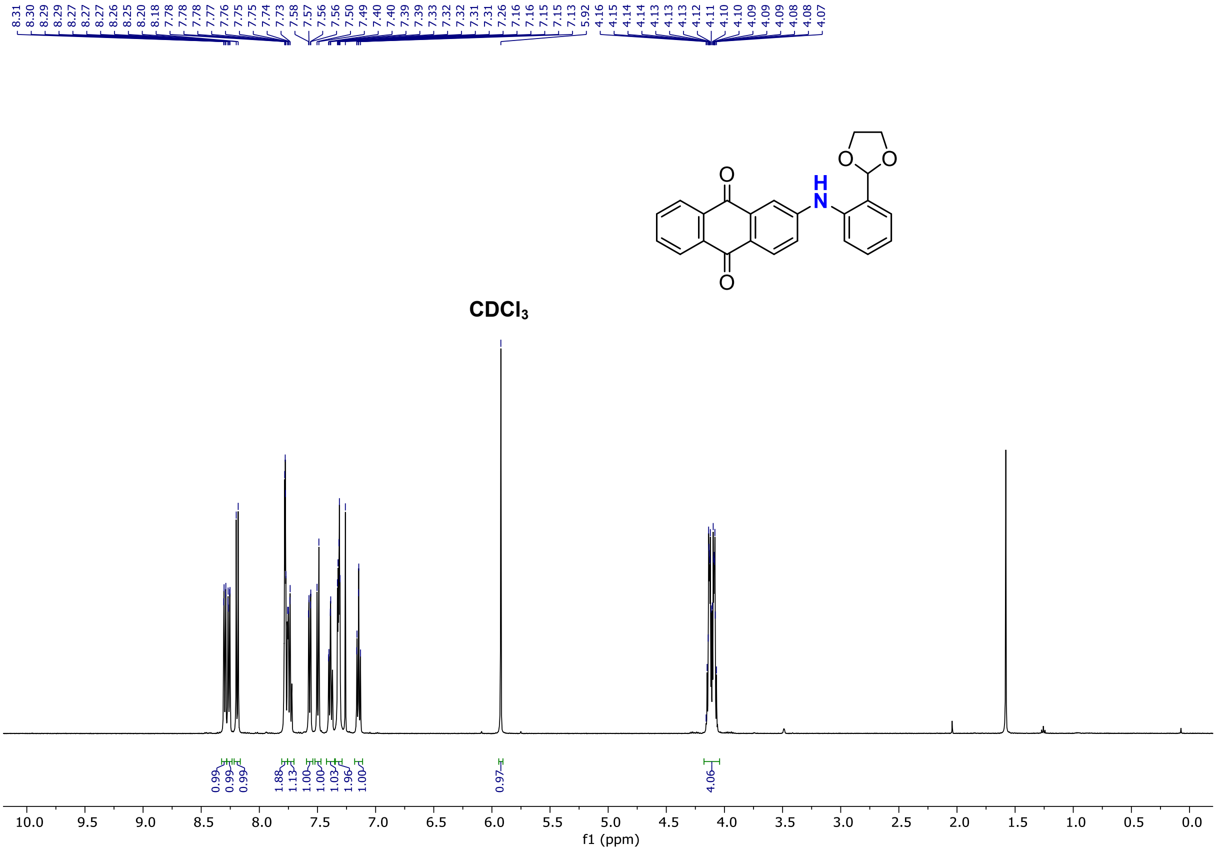

Figure S90. ^{1}H NMR spectrum of **S9** in $CDCl_3$.

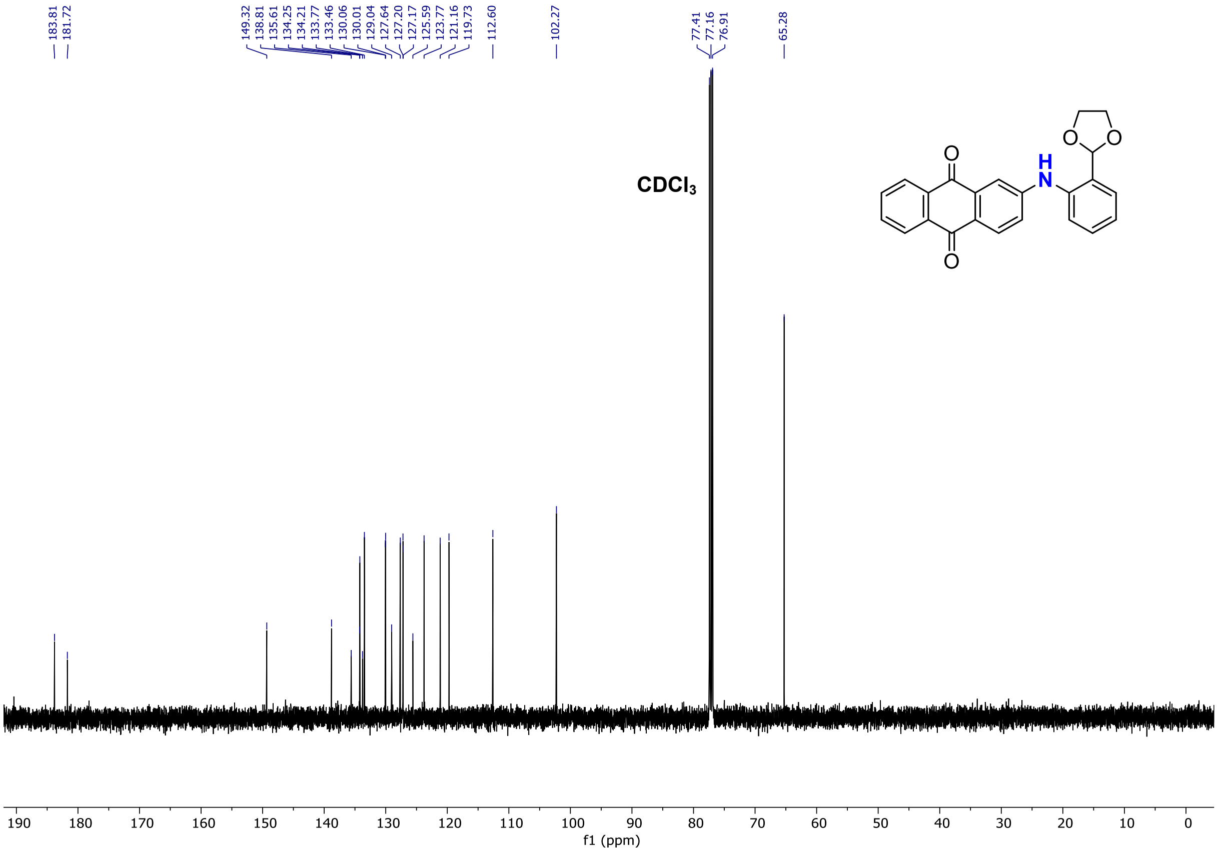

Figure S91. ^{13}C NMR spectrum of **S9** in $CDCl_3$.

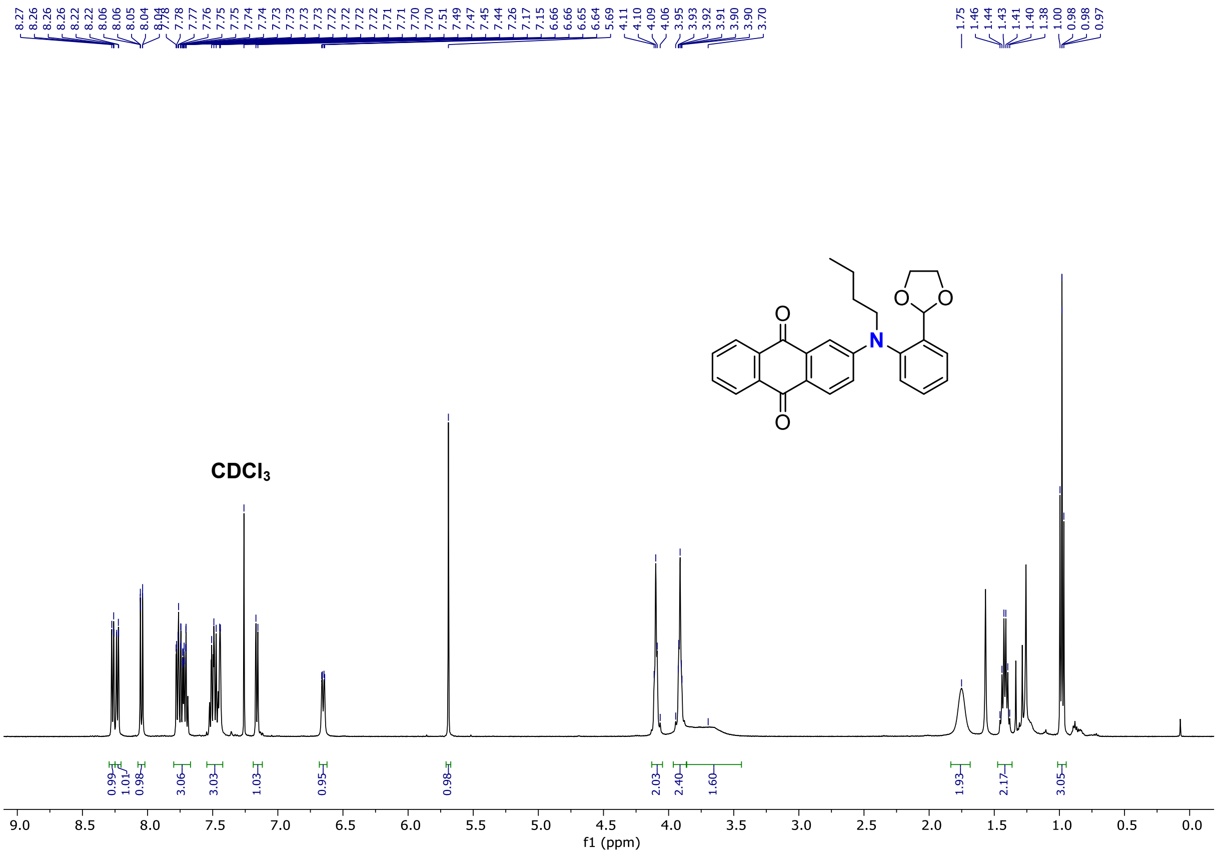

Figure S92. ^{1}H NMR spectrum of **S10** in $CDCl_3$.

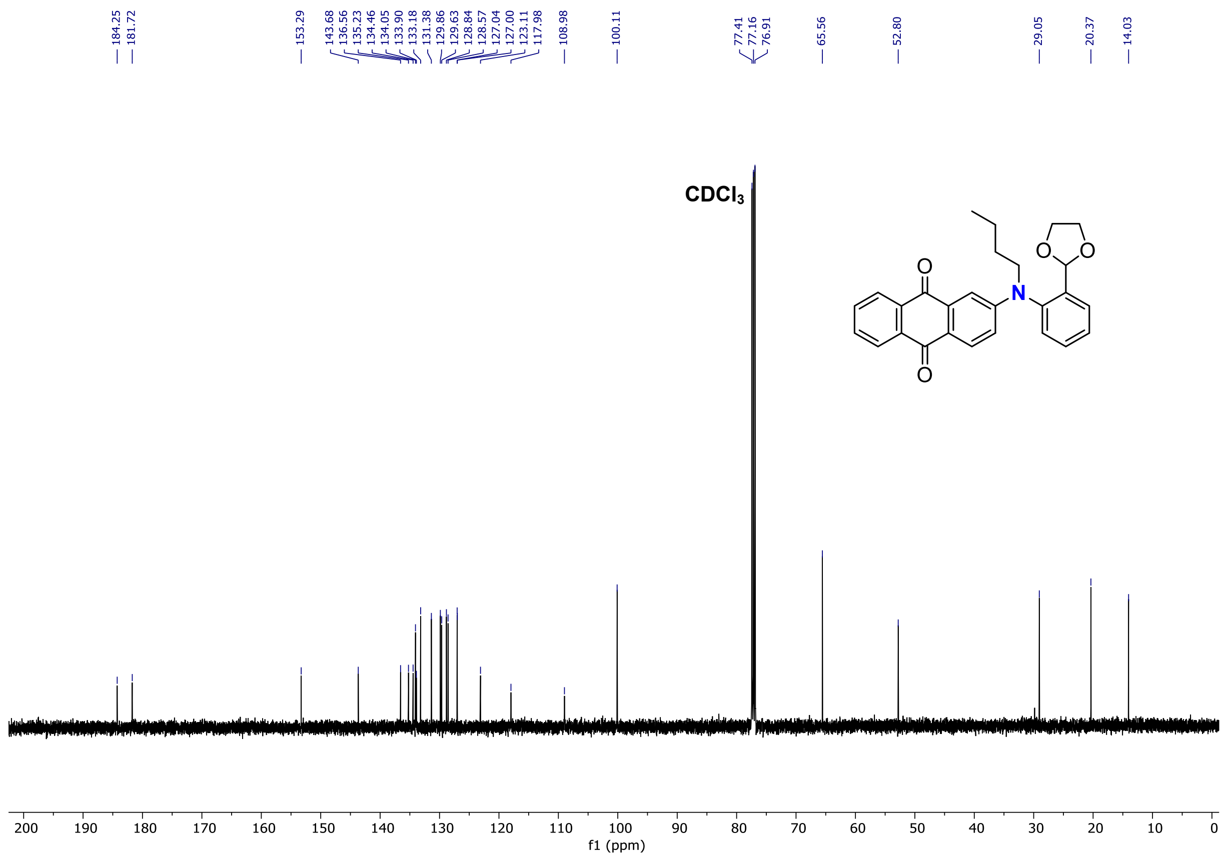

Figure S93. ^{13}C NMR spectrum of **S10** in $CDCl_3$.

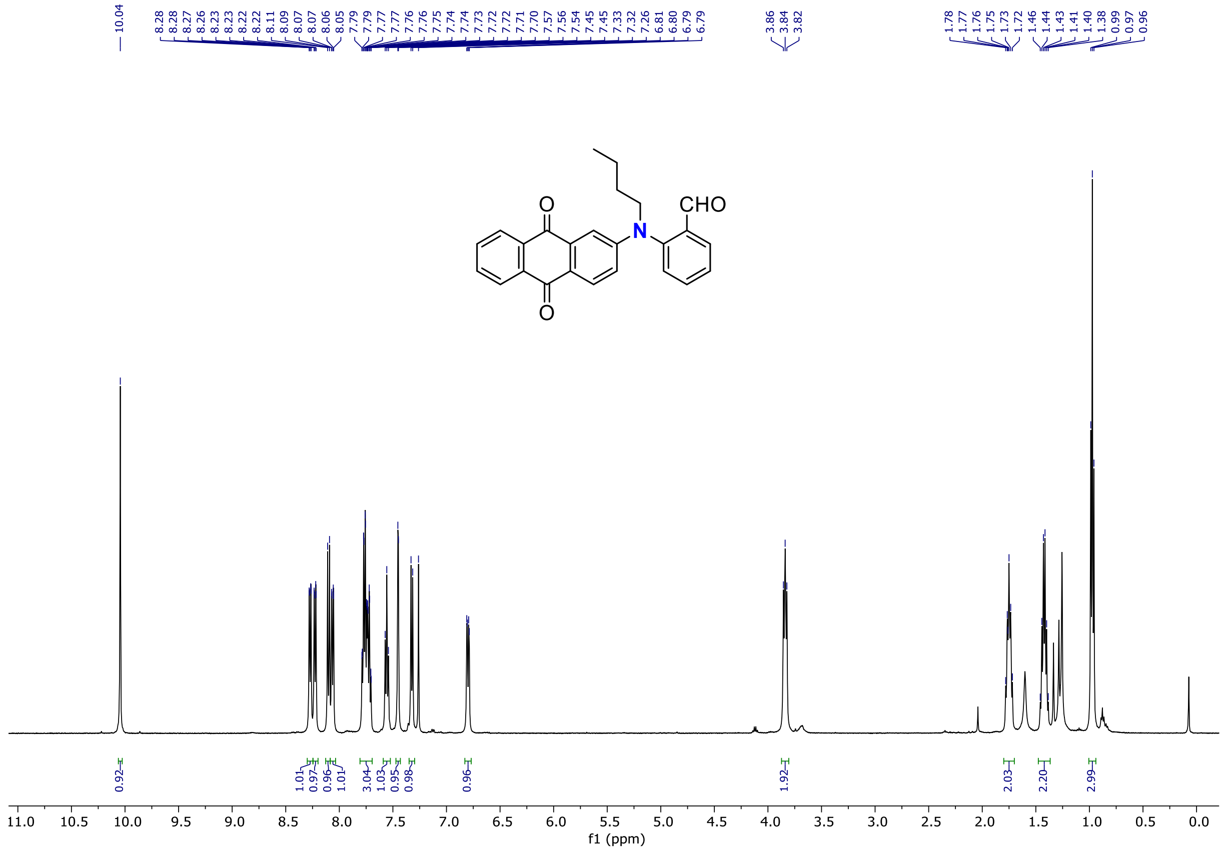

Figure S94. ^{1}H NMR spectrum of **13** in $CDCl_3$.

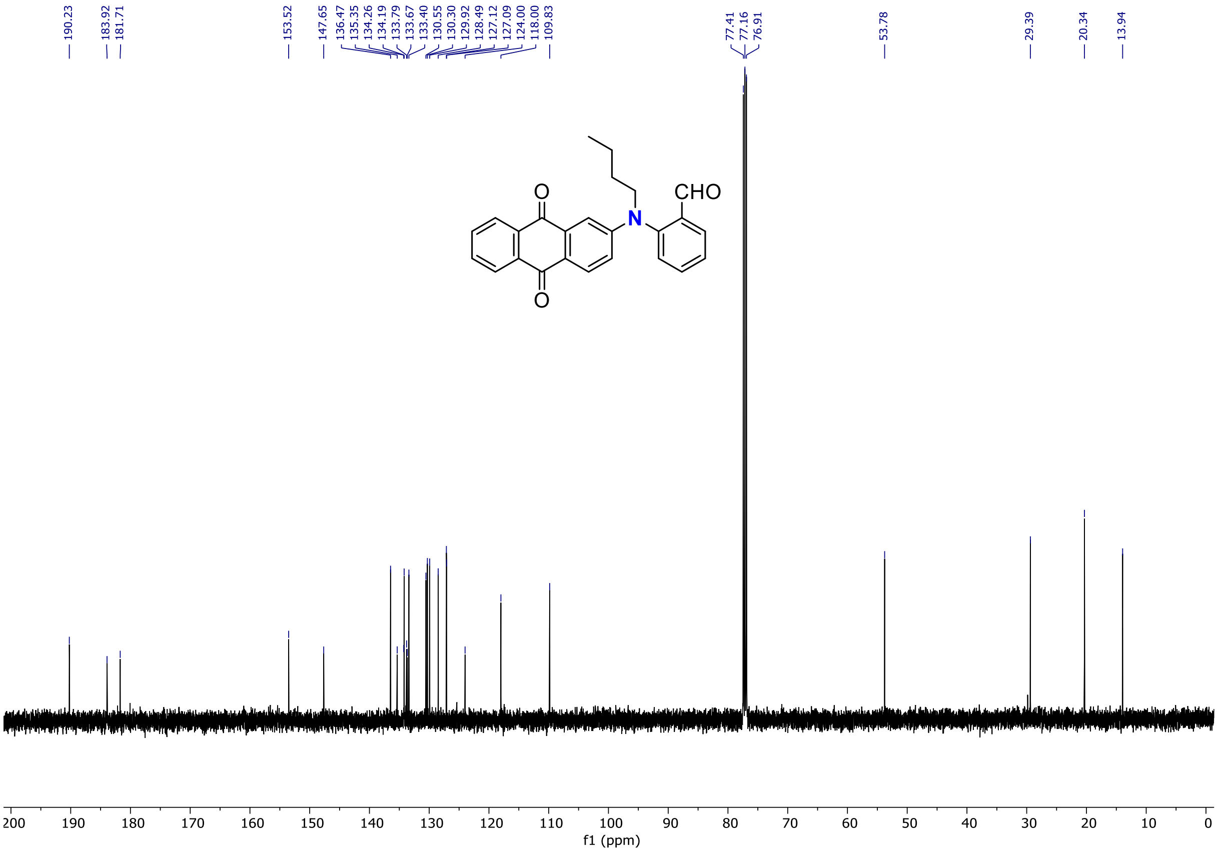

Figure S95. ^{13}C NMR spectrum of **13** in $CDCl_3$.

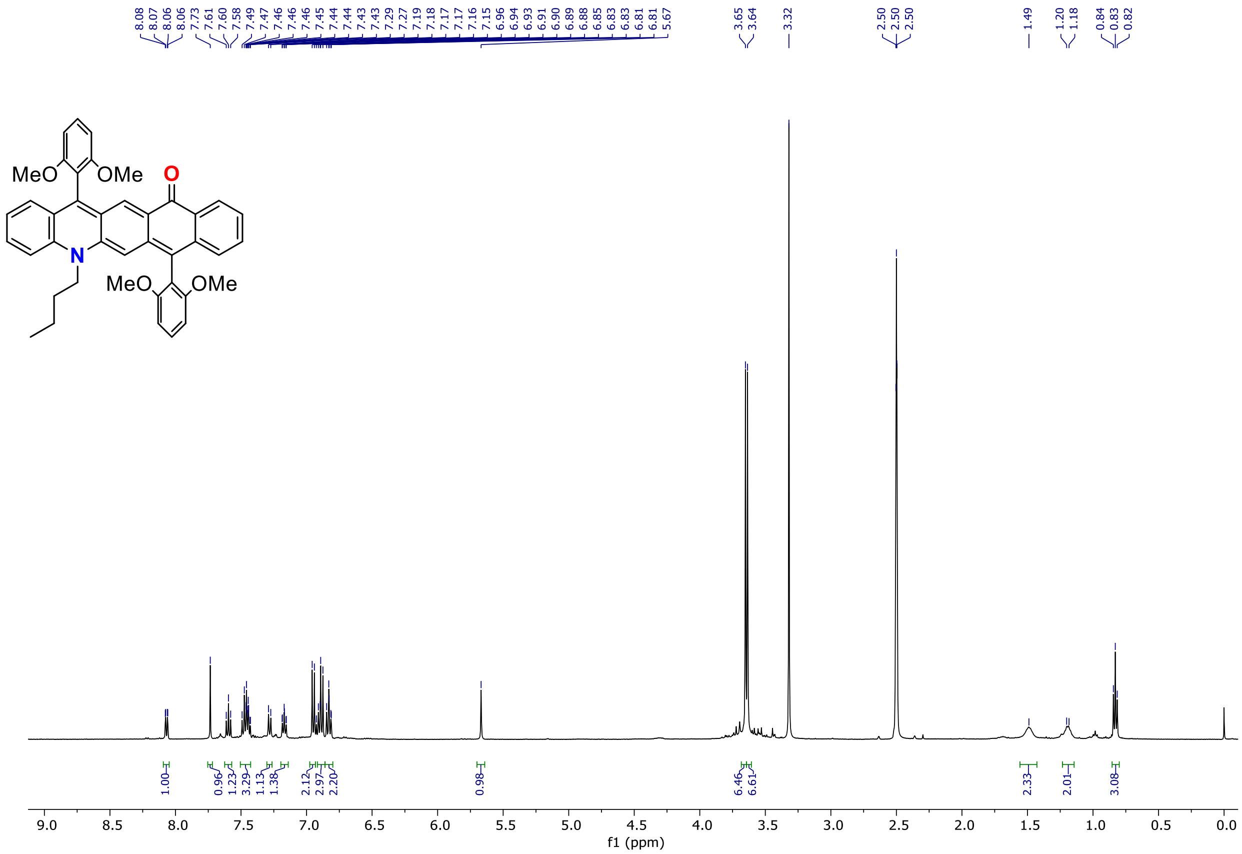

Figure S96. ^{1}H NMR spectrum of **CON-PA** in DMSO-d_6.

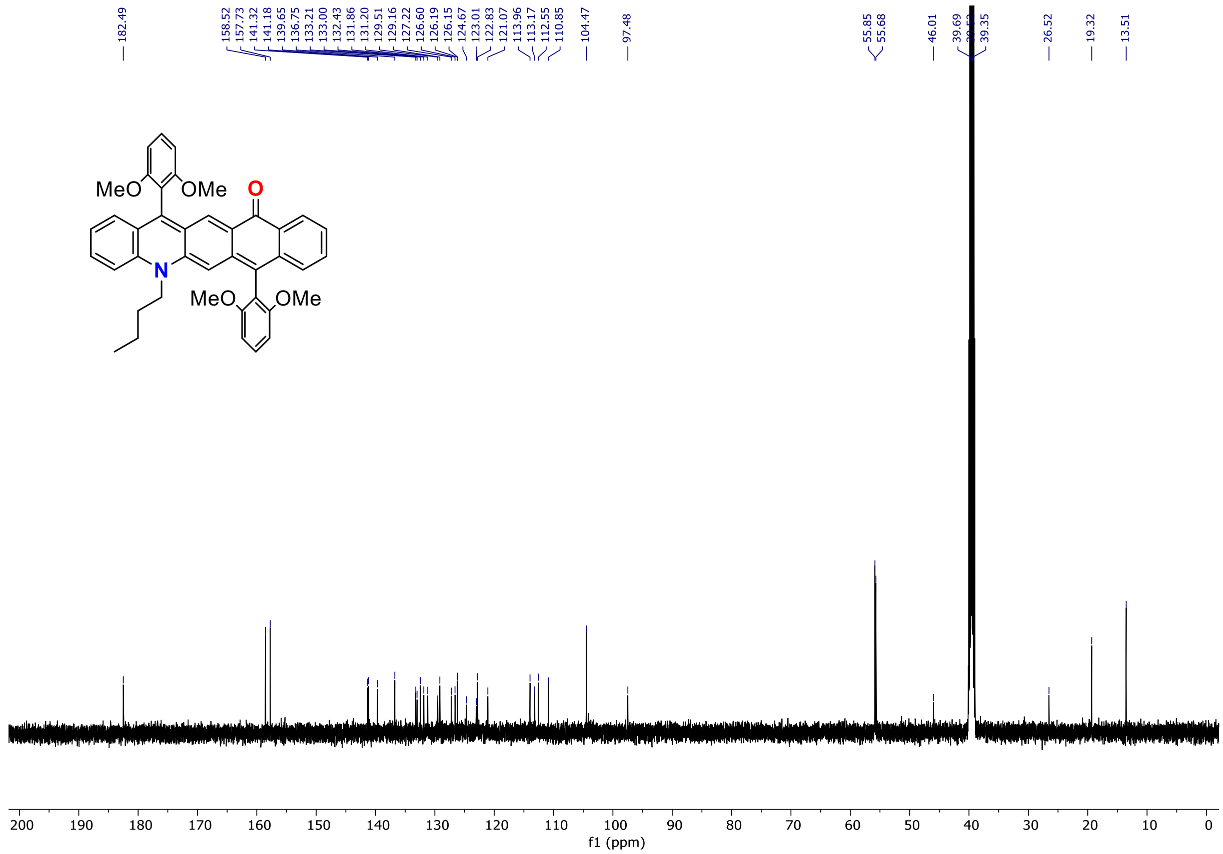

Figure S97. ^{13}C NMR spectrum of **CON-PA** in DMSO-d_6.

9. Mass spectra

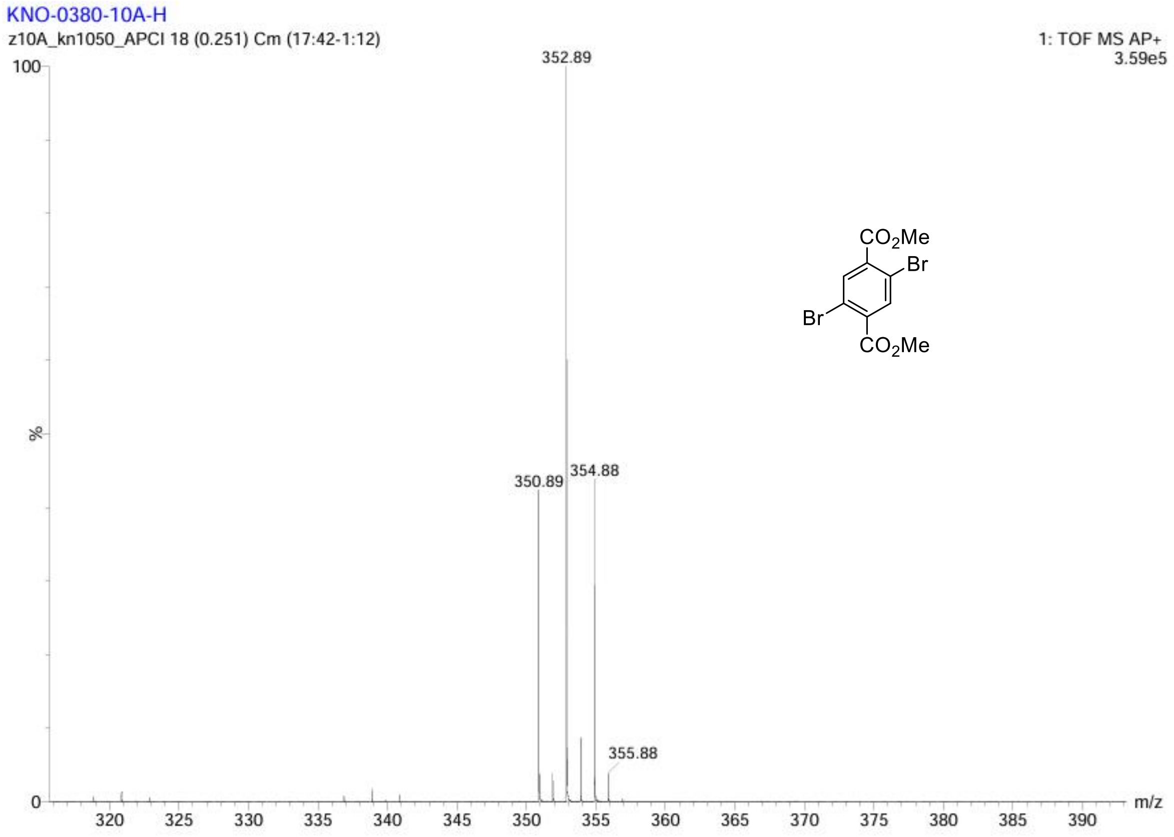

Figure S98. Mass spectrum of **3** $(M+H)^+$.

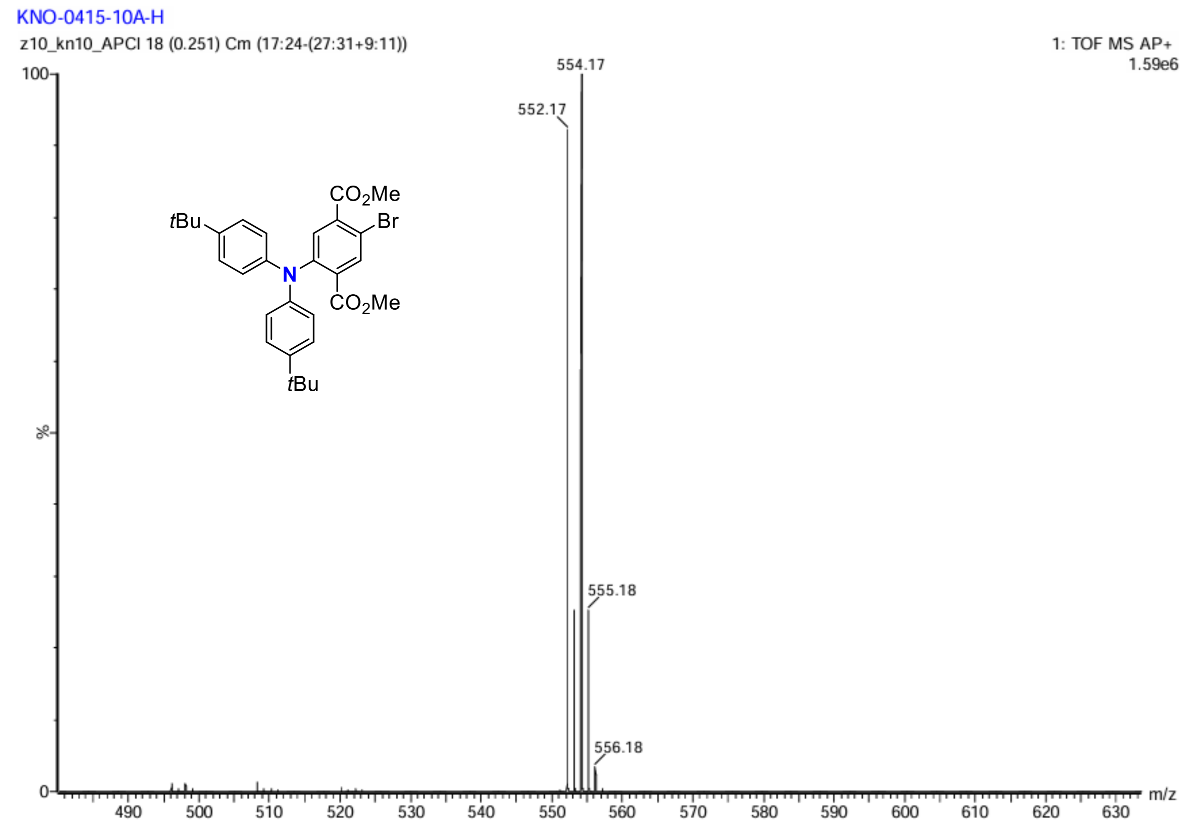

Figure S99. Mass spectrum of **4** (M+H)$^+$.

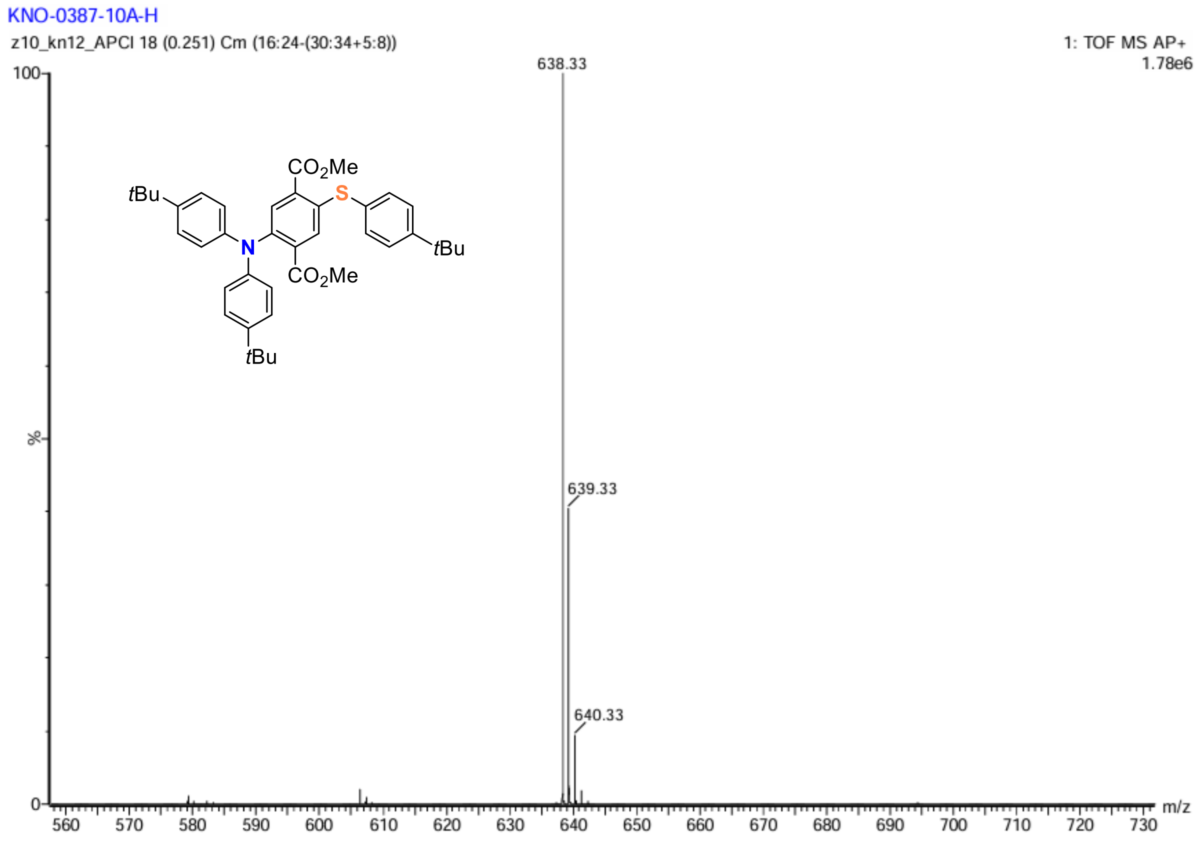

Figure S100. Mass spectrum of **S1** (M+H)$^+$.

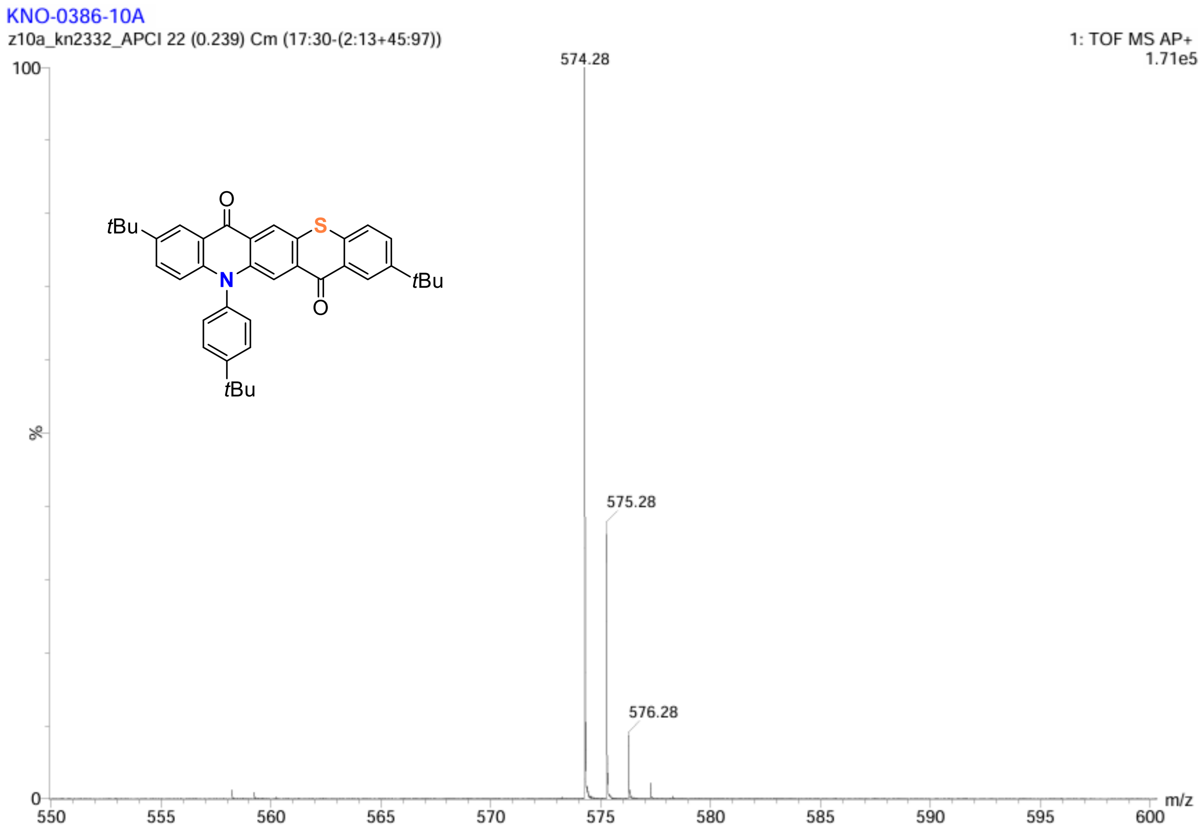

Figure S101. Mass spectrum of **6** (M+H)+.

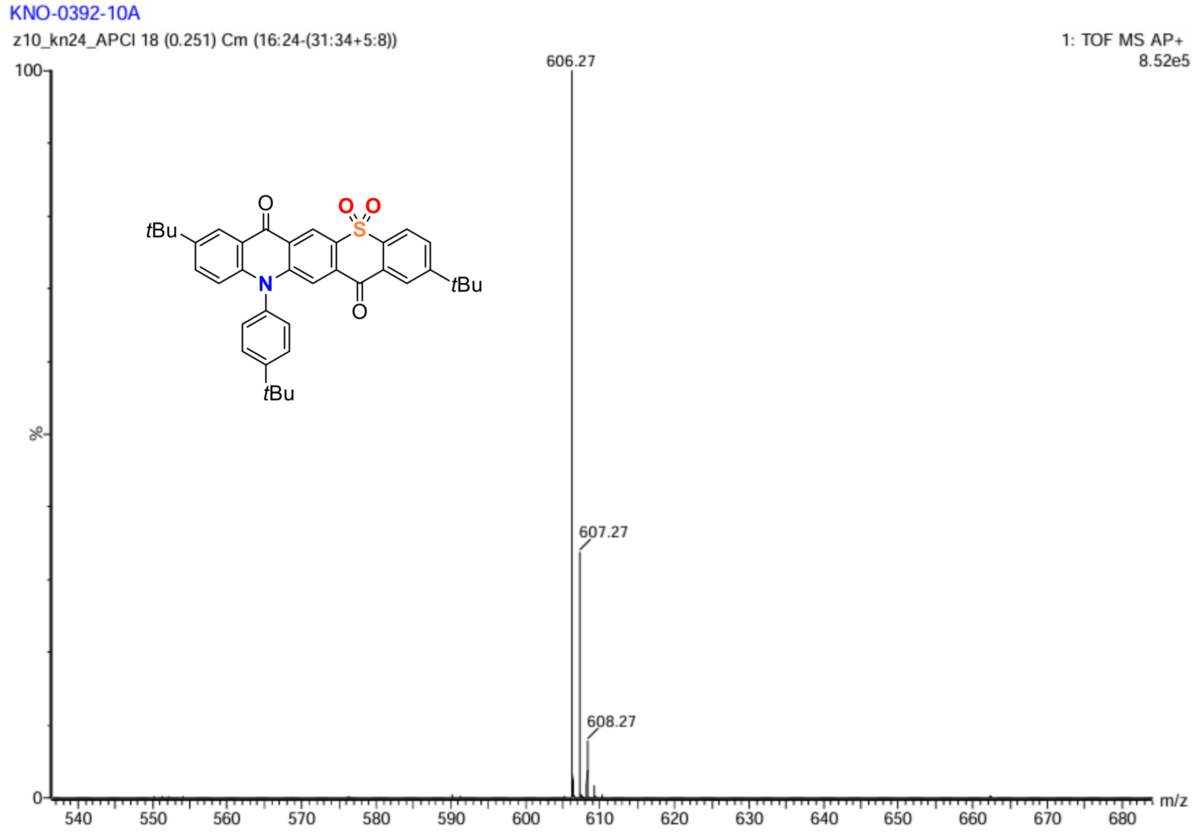

Figure S102. Mass spectrum of **7** (M+H)+.

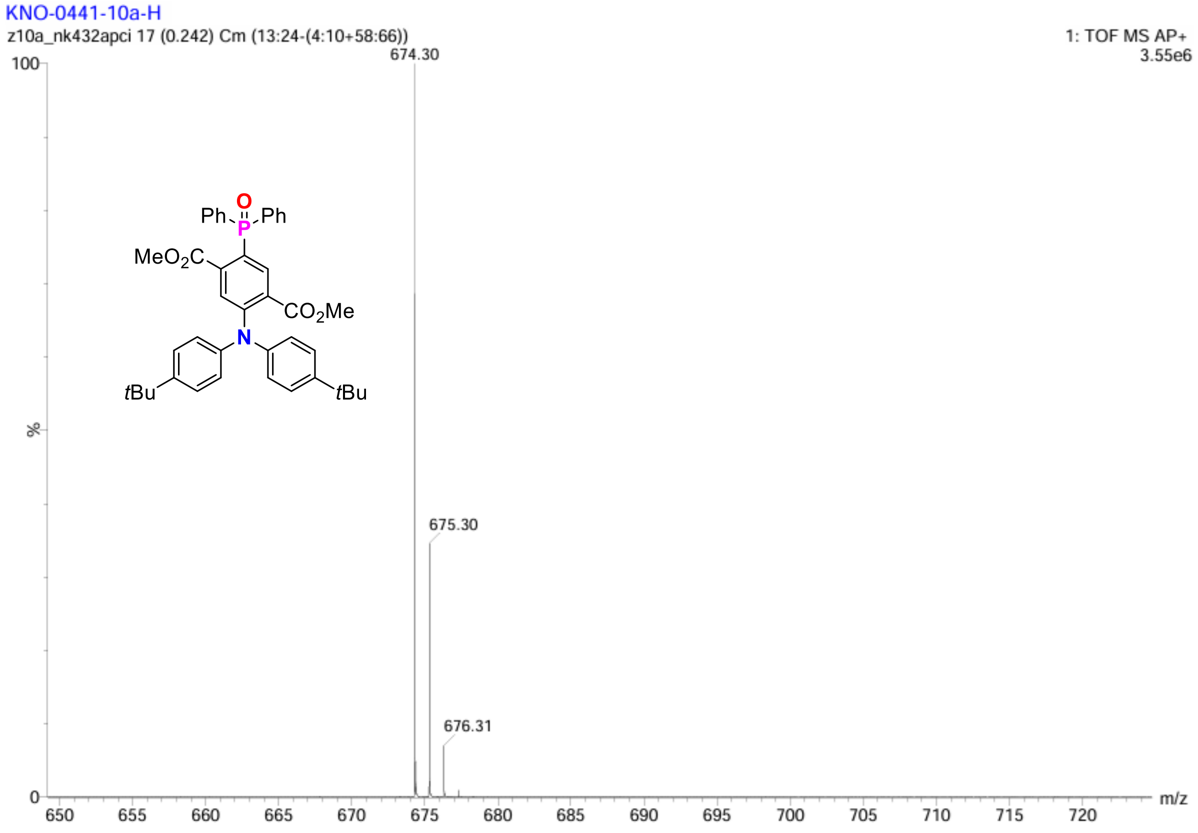

Figure S103. Mass spectrum of **S2** $(M+H)^+$.

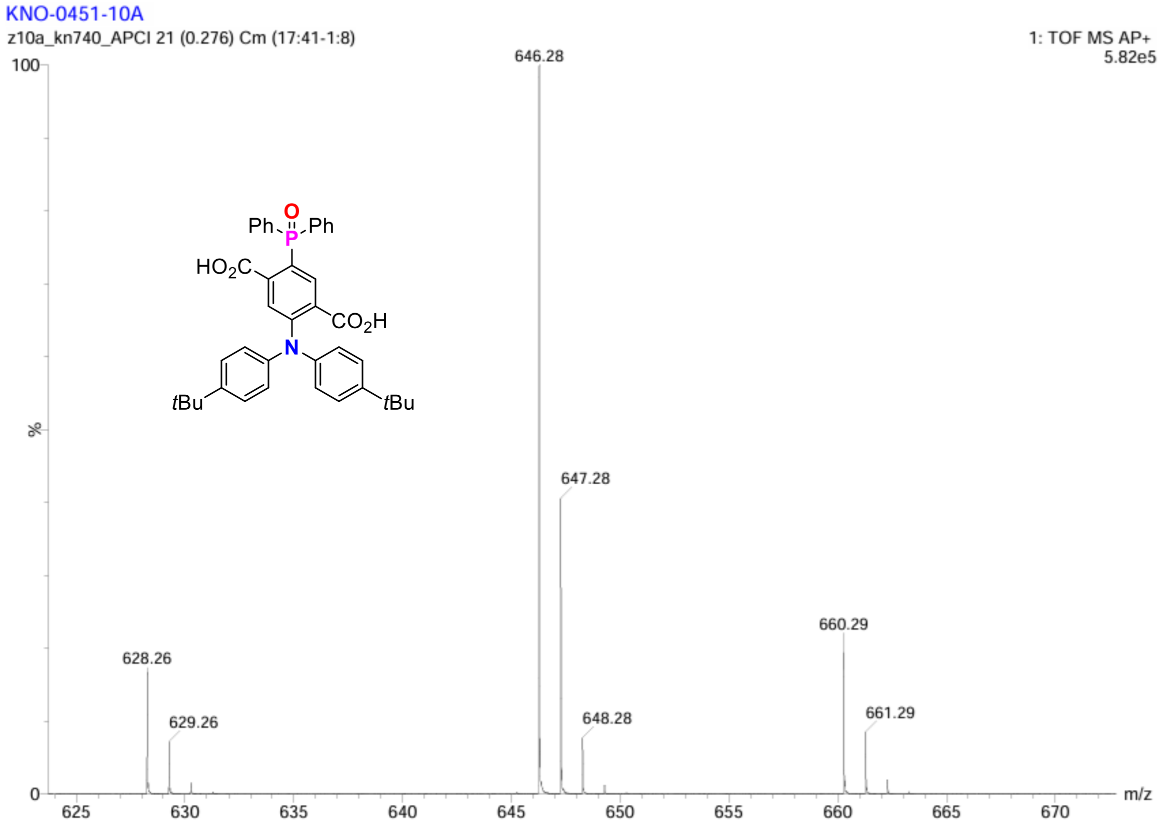

Figure S104. Mass spectrum of **8** $(M+H)^+$.

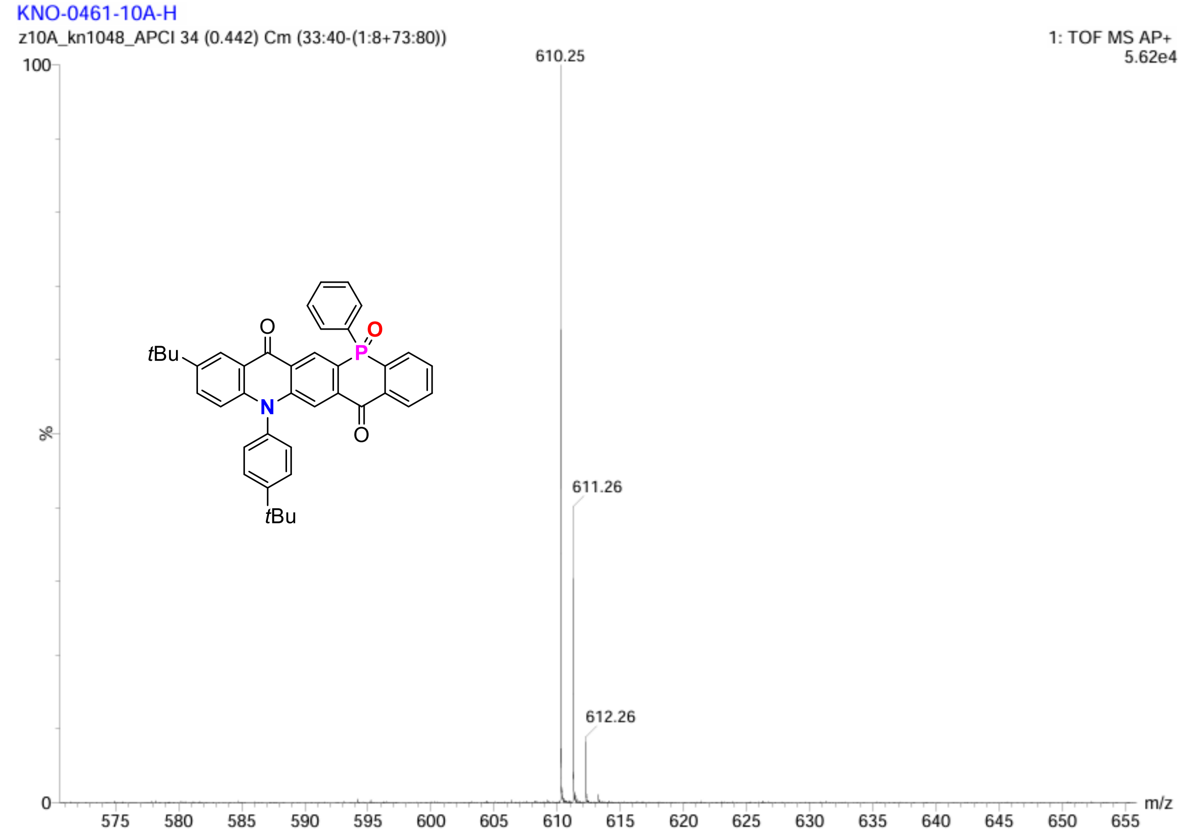

Figure S105. Mass spectrum of **10** $(M+H)^+$.

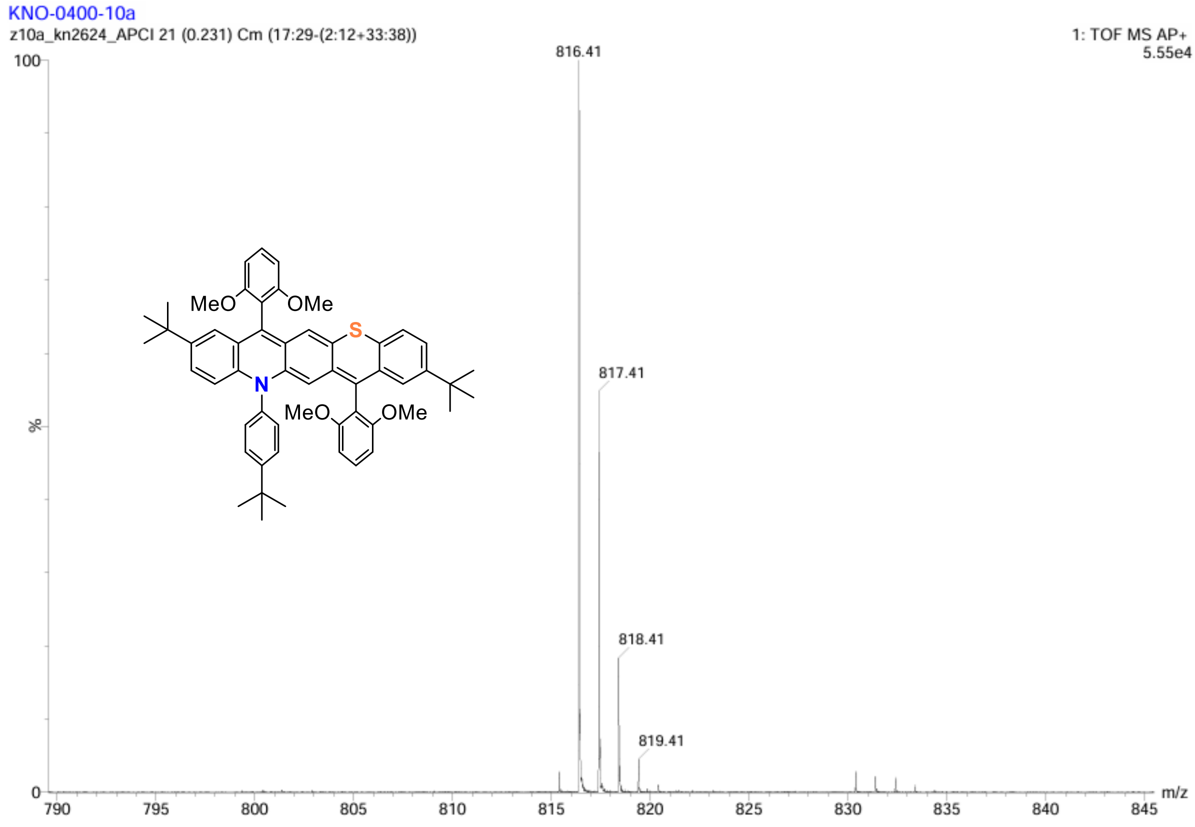

Figure S106. Mass spectrum of **SN-PA-a** $(M+H)^+$.

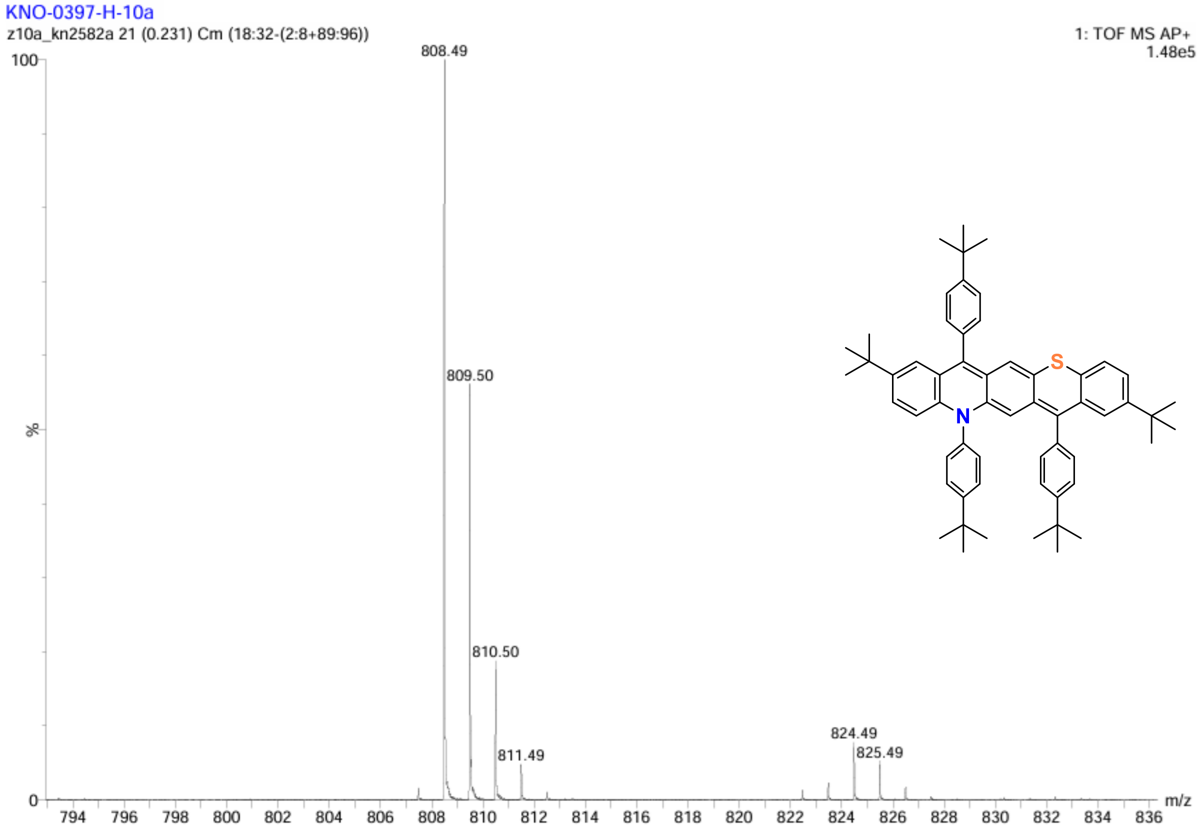

Figure S107. Mass spectrum of **SN-PA-b** (M+H)+.

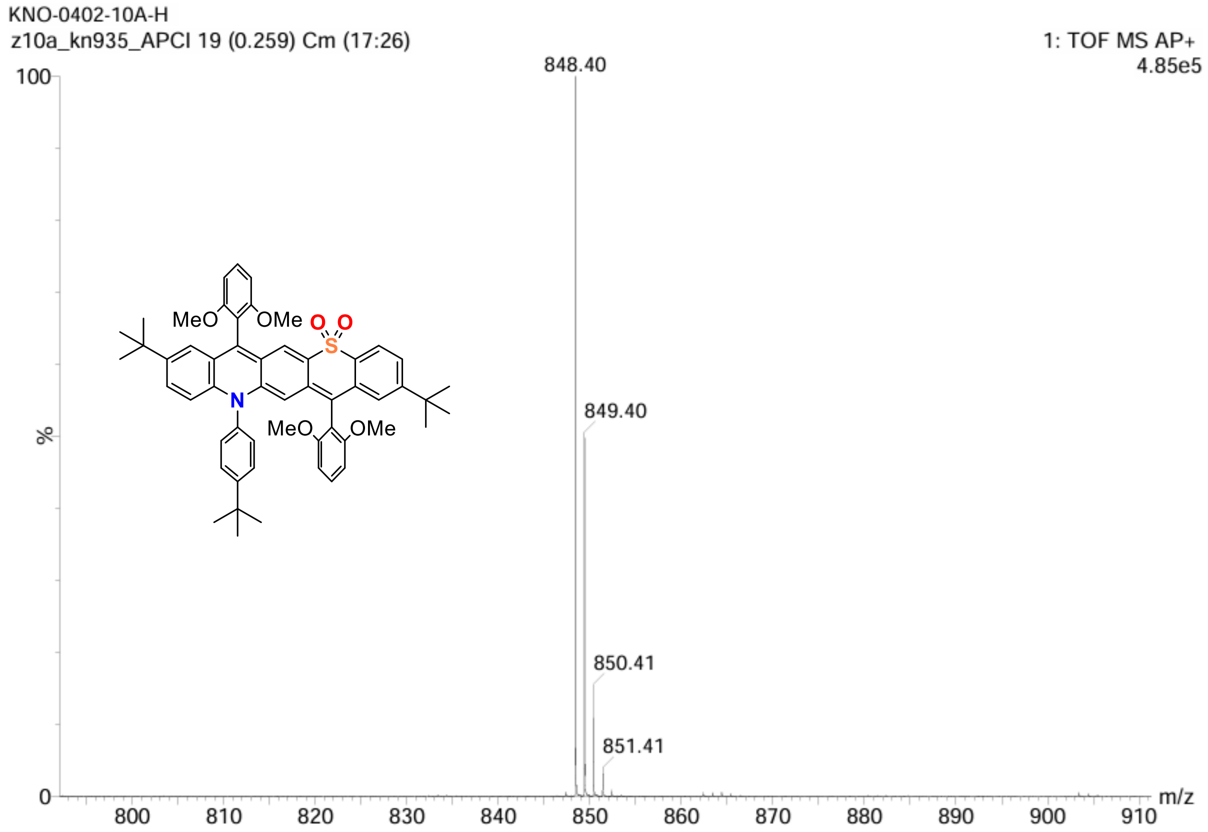

Figure S108. Mass spectrum of **SO2N-PA-a** (M+H)+.

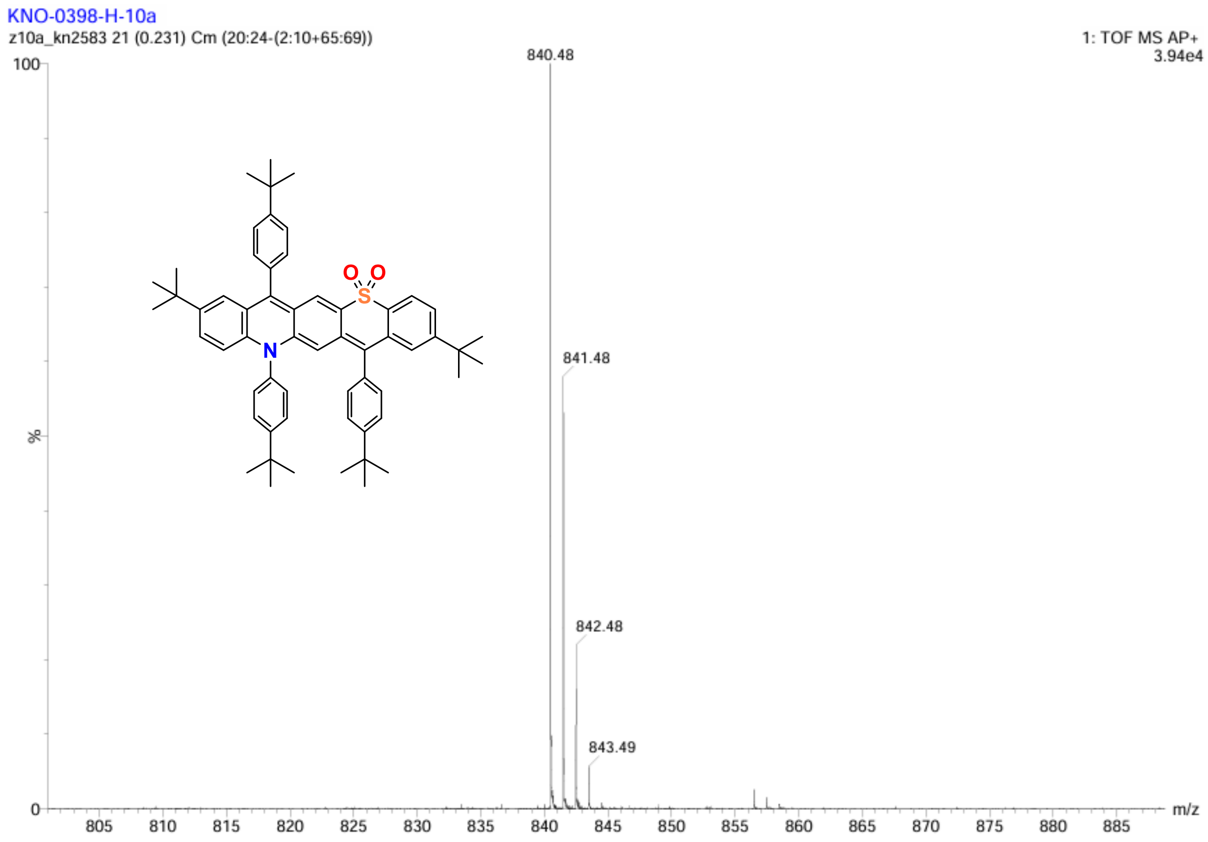

Figure S109. Mass spectrum of **SO_2N-PA-b** $(M+H)^+$.

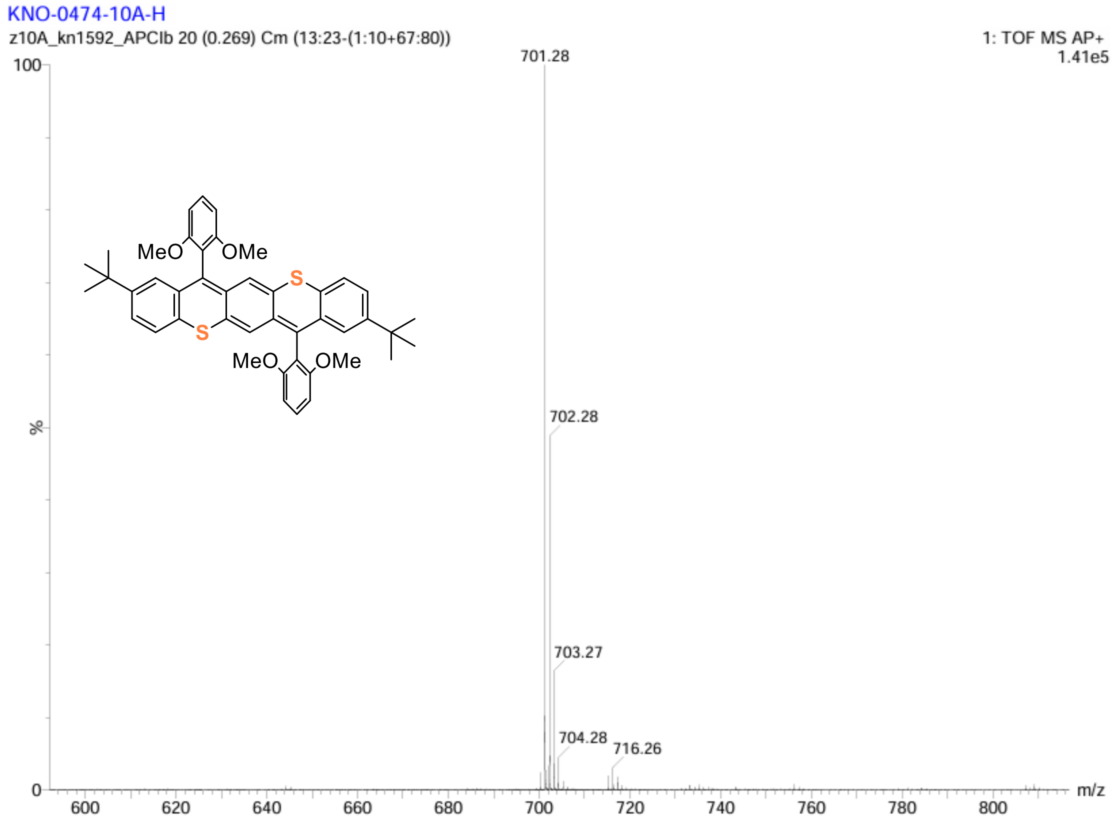

Figure S110. Mass spectrum of **17a** $(M+H)^+$.

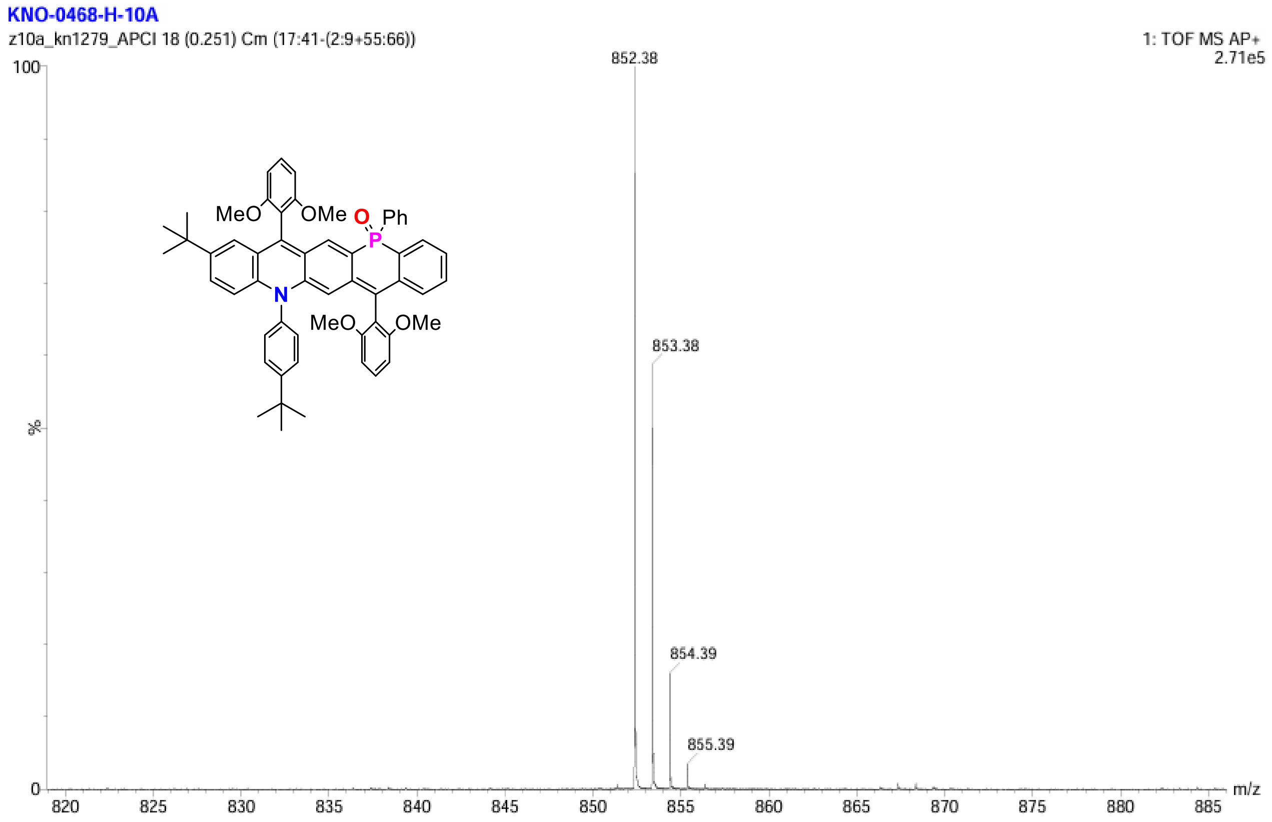

Figure S111. Mass spectrum of **PON-PA** (M+H)+.

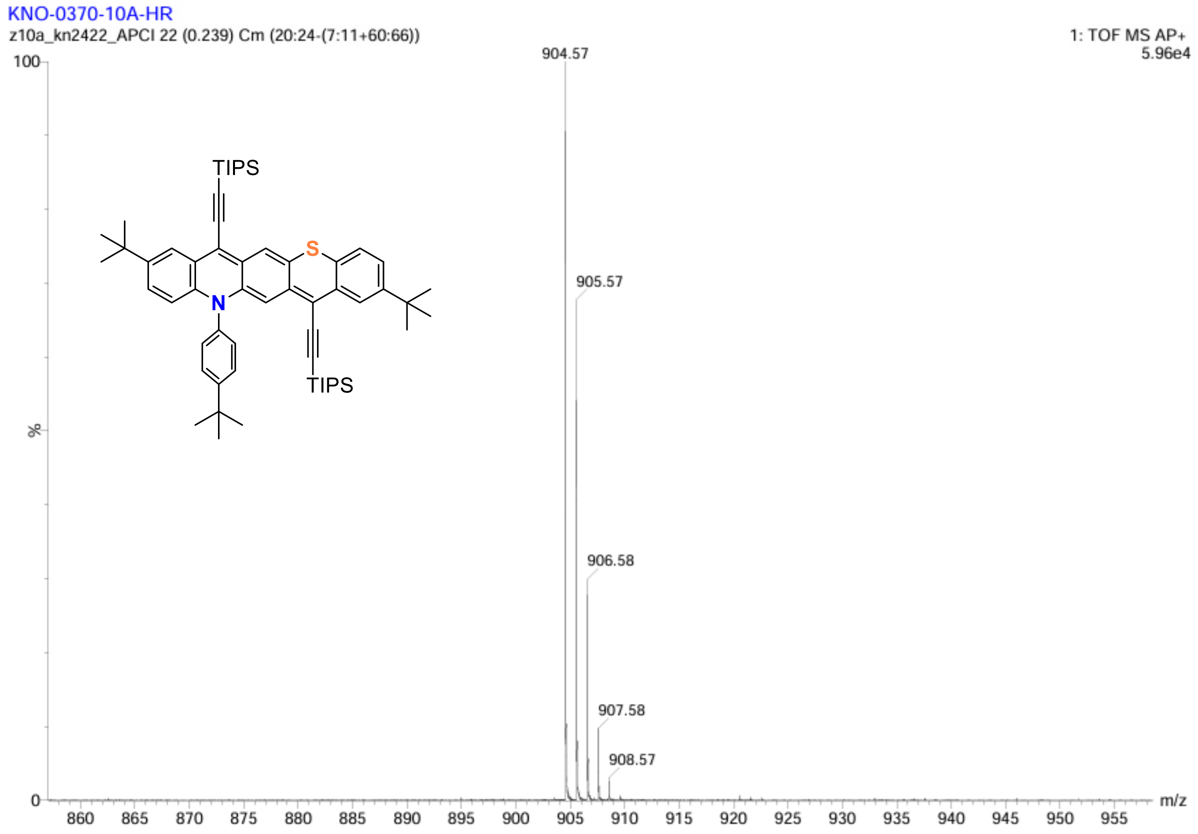

Figure S112. Mass spectrum of **SN-PA-c** (M+H)+.

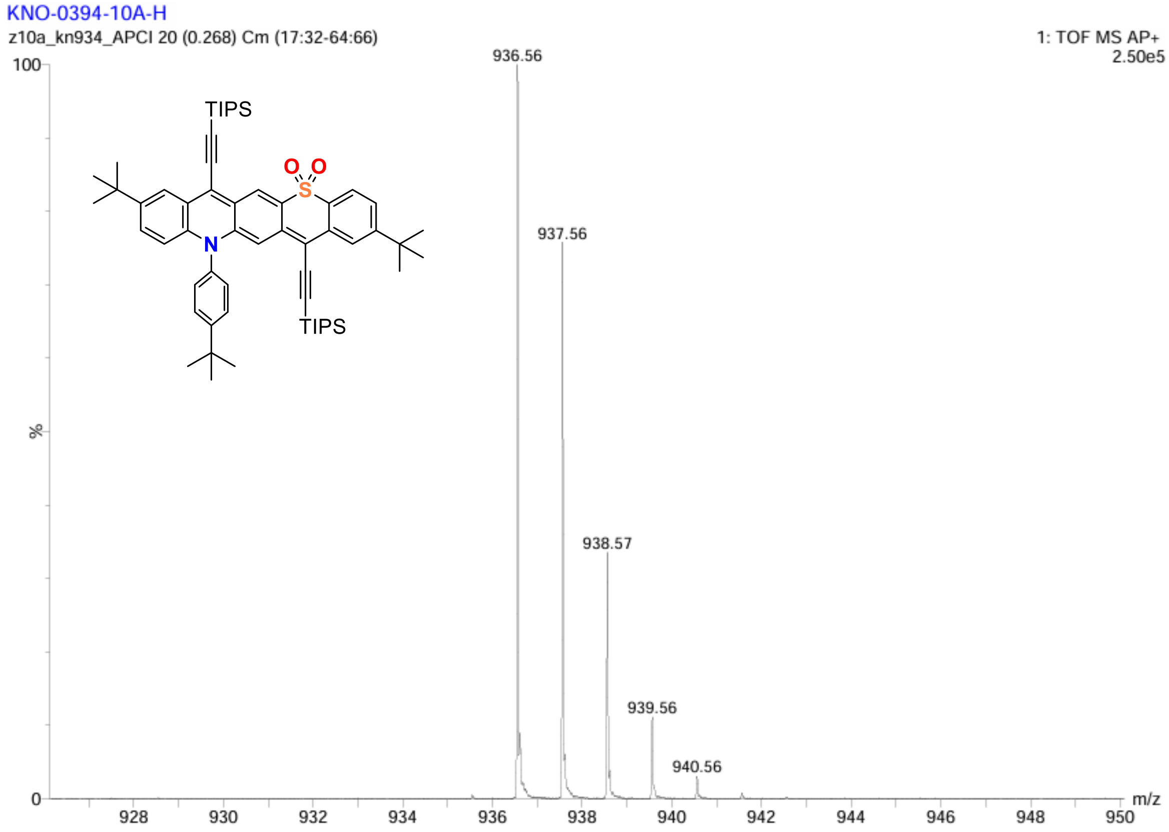

Figure S113. Mass spectrum of **SO_2N-PA-c** $(M+H)^+$.

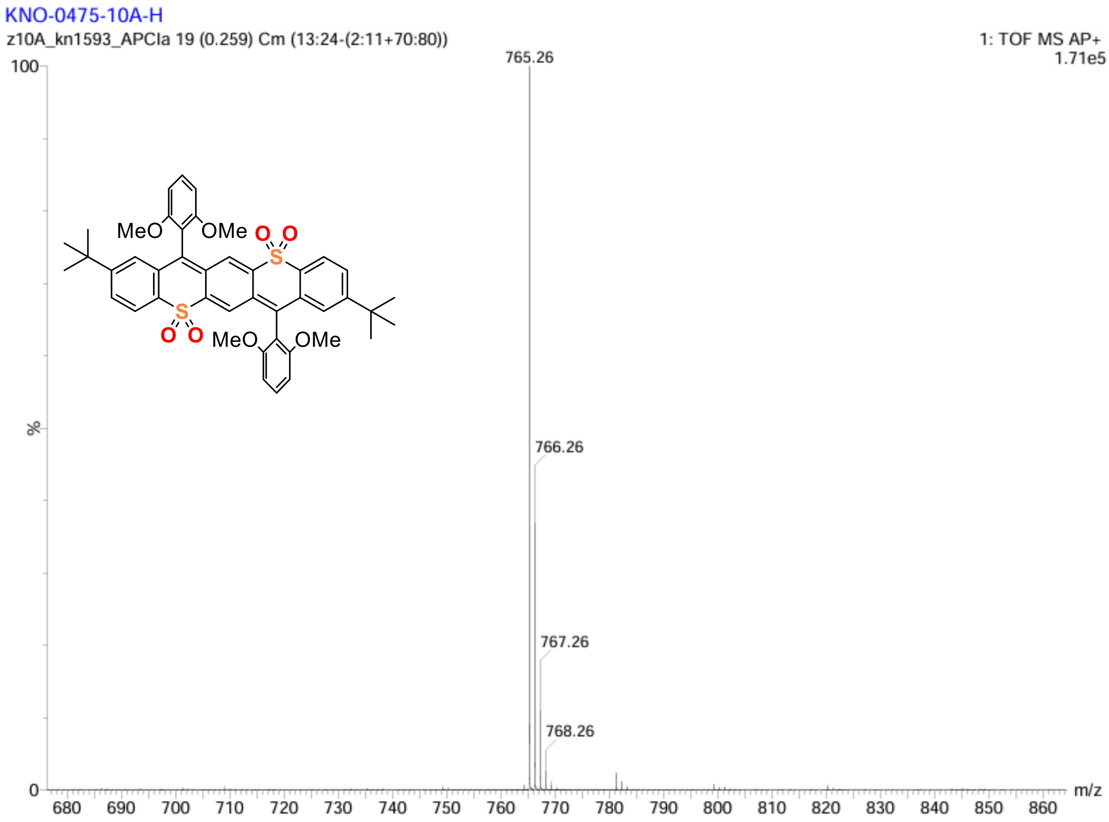

Figure S114. Mass spectrum of **diSO_2-PA-a** $(M+H)^+$.

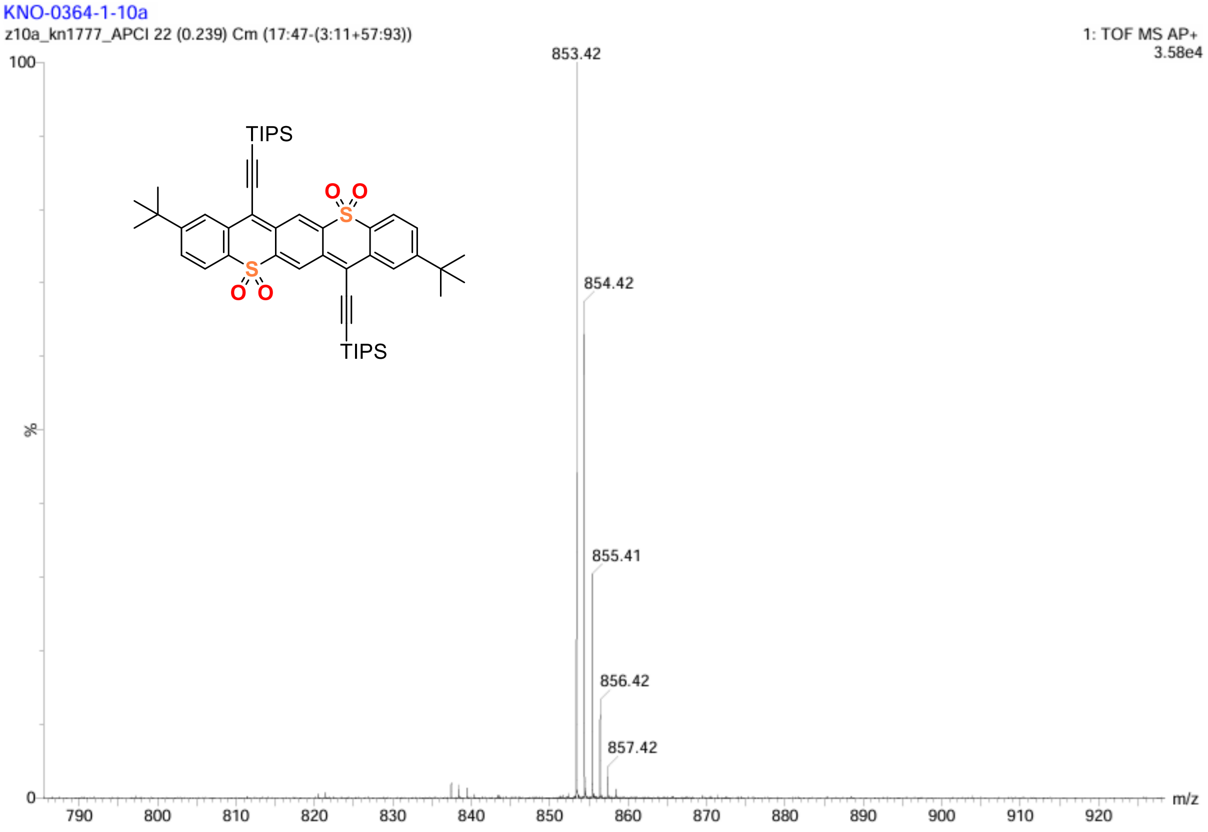

Figure S115. Mass spectrum of **diSO$_2$-PA-c** (M+H)$^+$.

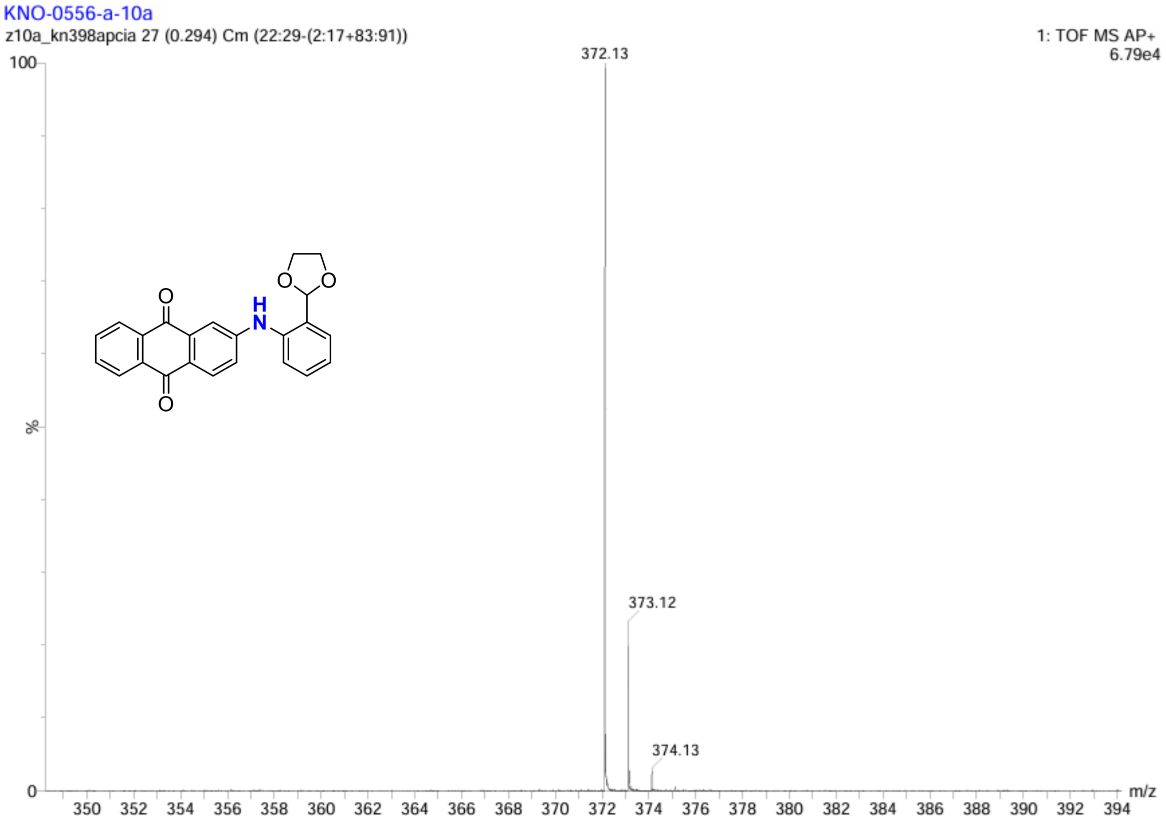

Figure S116. Mass spectrum of **S9** (M+H)$^+$.

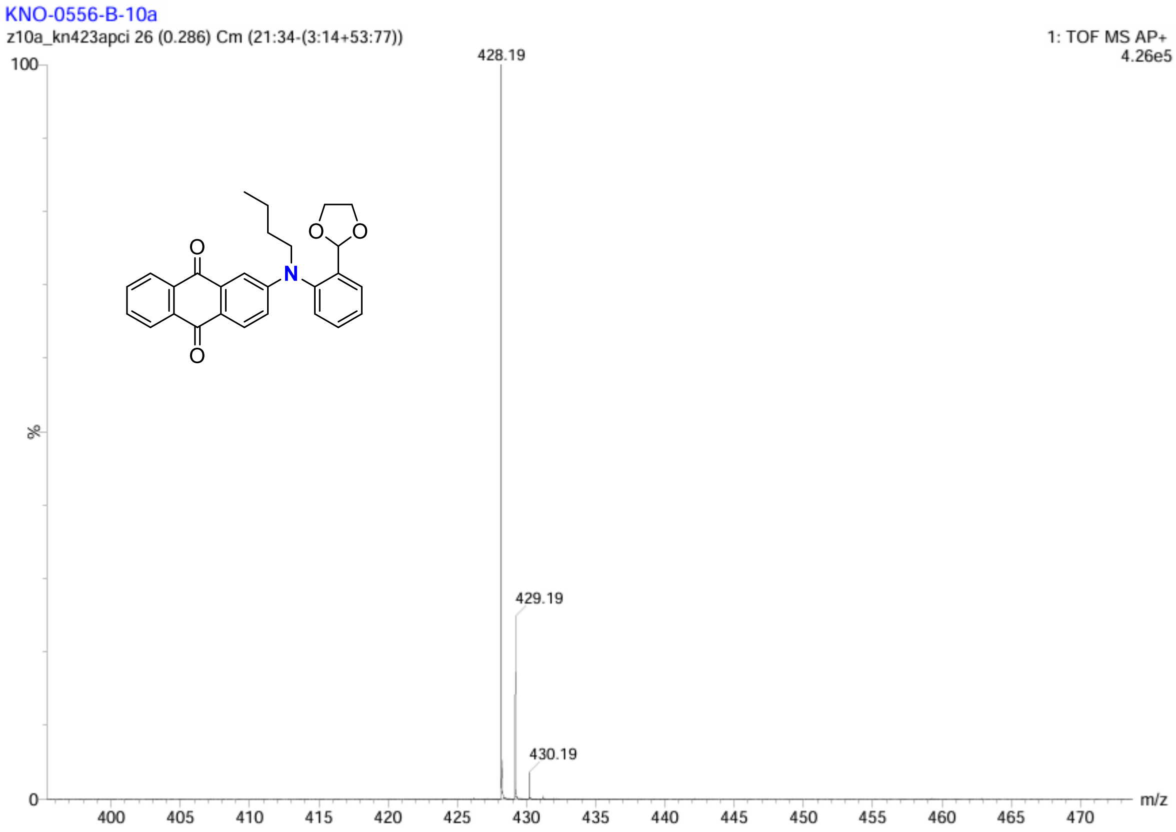

Figure S117. Mass spectrum of **S10** (M+H)$^+$.

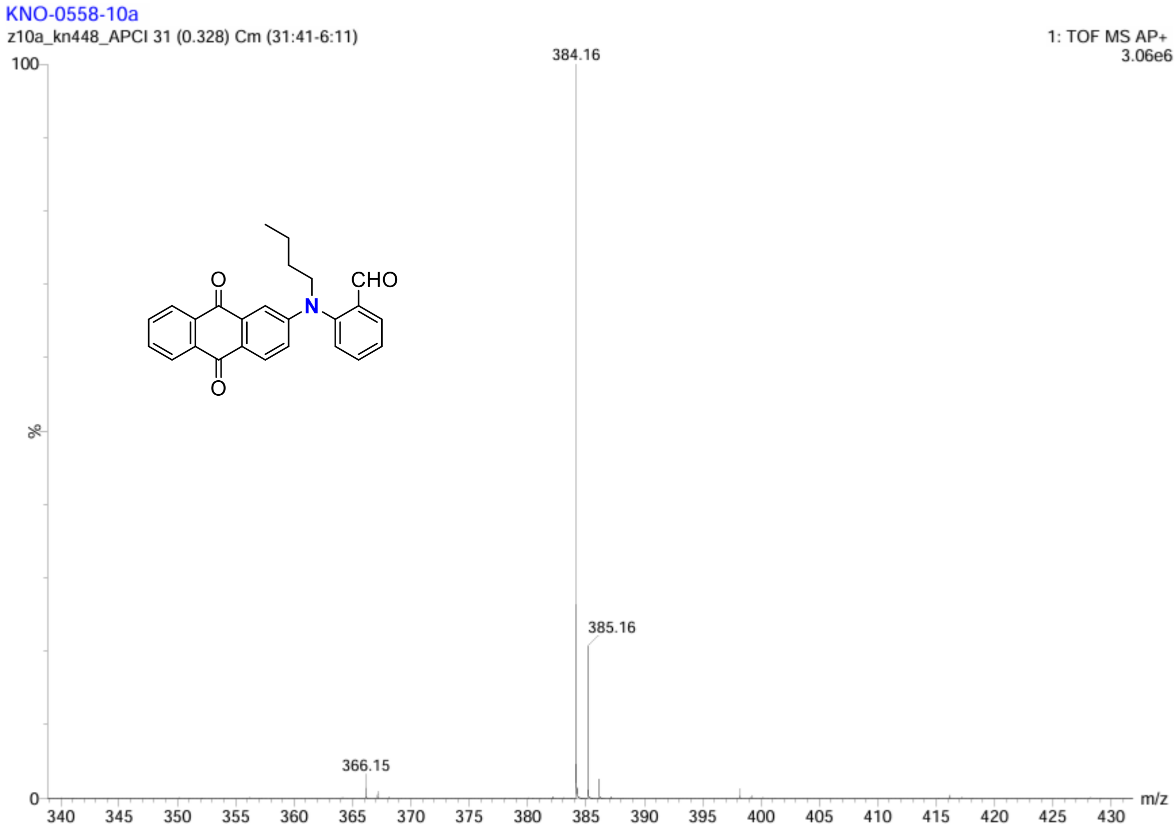

Figure S118. Mass spectrum of **13** (M+H)$^+$.

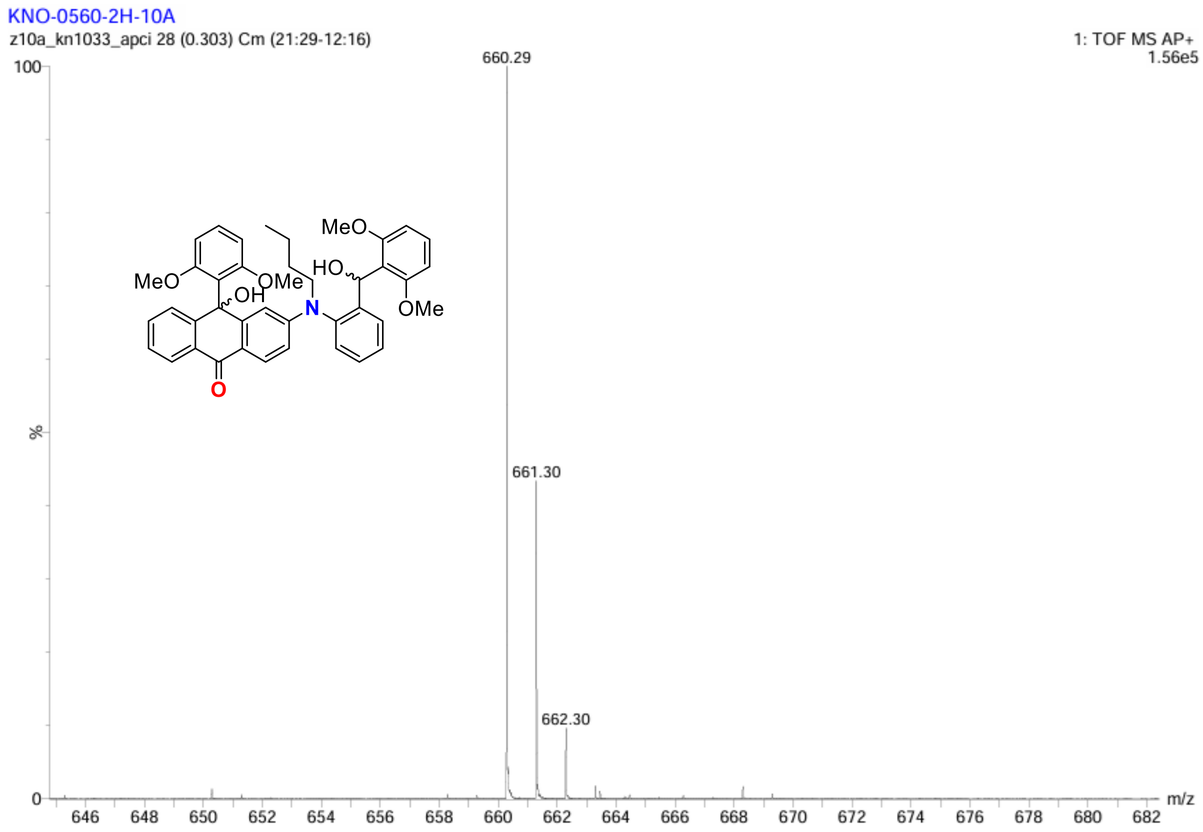

Figure S119. Mass spectrum of **14** $(M+H)^+$.

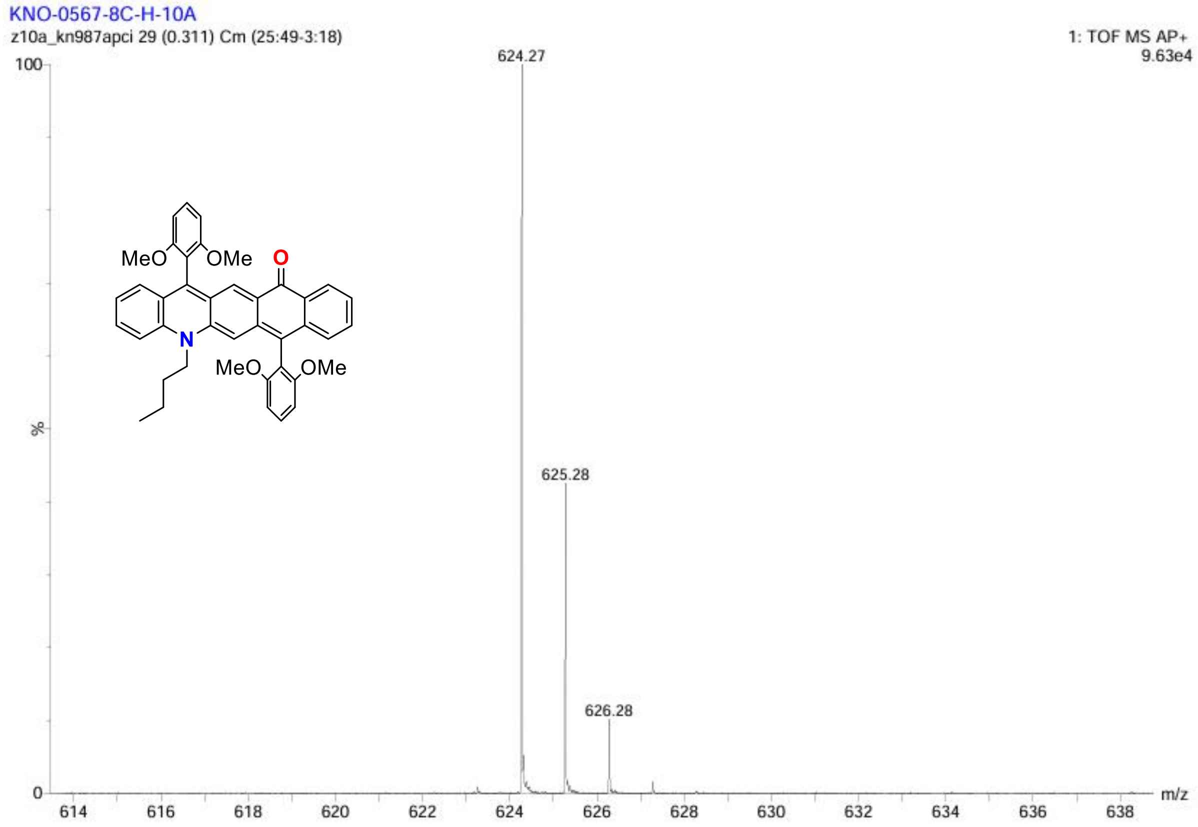

Figure S120. Mass spectrum of **CON-PA** $(M+H)^+$.
